# Establishment of Protocols for Global Metabolomics by LC-MS for Biomarker Discovery

**DOI:** 10.1371/journal.pone.0160555

**Published:** 2016-08-31

**Authors:** Daisuke Saigusa, Yasunobu Okamura, Ikuko N. Motoike, Yasutake Katoh, Yasuhiro Kurosawa, Reina Saijyo, Seizo Koshiba, Jun Yasuda, Hozumi Motohashi, Junichi Sugawara, Osamu Tanabe, Kengo Kinoshita, Masayuki Yamamoto

**Affiliations:** 1 Department of Integrative Genomics, Tohoku Medical Megabank Organization, Tohoku University, Sendai, Miyagi, Japan; 2 Medical Biochemistry, Tohoku University School of Medicine, Sendai, Miyagi, Japan; 3 CREST, Japan Agency for Medical Research and Development (AMED), Chiyoda, Tokyo, Japan; 4 Department of Systems Bioinformatics, Graduate School of Information Sciences, Tohoku University, Sendai, Miyagi, Japan; 5 Department of Biochemistry, Tohoku University Graduate School of Medicine, Sendai, Miyagi, Japan; 6 Department of Gynecology and Obstetrics, Tohoku University Graduate School of Medicine, Sendai, Miyagi, Japan; 7 Department of Gene Expression Regulation, Institute of Development, Aging and Cancer, Tohoku University, Sendai, Miyagi, Japan; Instituto de Investigacion Sanitaria INCLIVA, SPAIN

## Abstract

Metabolomics is a promising avenue for biomarker discovery. Although the quality of metabolomic analyses, especially global metabolomics (G-Met) using mass spectrometry (MS), largely depends on the instrumentation, potential bottlenecks still exist at several basic levels in the metabolomics workflow. Therefore, we established a precise protocol initially for the G-Met analyses of human blood plasma to overcome some these difficulties. In our protocol, samples are deproteinized in a 96-well plate using an automated liquid-handling system, and conducted either using a UHPLC-QTOF/MS system equipped with a reverse phase column or a LC-FTMS system equipped with a normal phase column. A normalization protocol of G-Met data was also developed to compensate for intra- and inter-batch differences, and the variations were significantly reduced along with our normalization, especially for the UHPLC-QTOF/MS data with a C18 reverse-phase column for positive ions. Secondly, we examined the changes in metabolomic profiles caused by the storage of EDTA-blood specimens to identify quality markers for the evaluation of the specimens’ pre-analytical conditions. Forty quality markers, including lysophospholipids, dipeptides, fatty acids, succinic acid, amino acids, glucose, and uric acid were identified by G-Met for the evaluation of plasma sample quality and established the equation of calculating the quality score. We applied our quality markers to a small-scale study to evaluate the quality of clinical samples. The G-Met protocols and quality markers established here should prove useful for the discovery and development of biomarkers for a wider range of diseases.

## Introduction

Reliable disease biomarkers are essential for establishing an advanced healthcare system where preventive measures, early diagnosis, and treatment are provided based on personalized risk assessments. Metabolomics is a promising approach in the search for disease biomarkers because the metabolite concentrations of body fluids are considered as quantitative traits that can describe and define phenotypic characteristics of each individual, which are generated through interactions between genes and environmental influences [1–4]. Metabolite profiling by nuclear magnetic resonance (NMR) has been described as a precise and reproducible method for biomarker discovery [5–7]. Recently, metabolomics technologies based on mass spectrometry (MS) have made remarkable advances. The MS-based analysis of targeted metabolomics (T-Met) of primary and secondary metabolites has significantly contributed to biomarker discovery and the elucidation of disease patho-physiologies; thus, this method has become widely employed as a versatile analytical technology [8–11]. The methods for metabolite profiling have matured from those developed in small-scale studies with tens to hundreds of human subjects, toward developing more comprehensive analyses to gain understandings of disease states from large-scale studies with thousands of participants, such as population-based prospective cohort studies [12,13].

Tohoku Medical Megabank Organization (ToMMo) is conducting a prospective community-based cohort study in the regions affected by the 2011 Great East Japan Earthquake and tsunami. In this cohort study, to establish methods for personalized disease-risk assessments according to the genomic information and biomarkers and genomic analyses of the study participants, along with proteomic and metabolomic analyses of blood plasma, we used both MS and NMR approaches [14].

Recently, translational studies with comprehensive omics analyses have been performed for clinical application [15–17]. Specifically, the MS-based analysis of global metabolomics (G-Met), which quantifies whole sets of metabolites in biological specimens without targeting specific molecules, has proven useful in the search for novel biomarkers [18–21]. However, one of the challenges in conducting metabolomic analyses with samples from cohort studies or clinical settings is the significant fluctuations of analytical results due to variations in pre-analytical conditions during sample collection and storage [22–25]. Several other bottlenecks and difficulties exist in the workflow of MS-based G-Met, such as the throughput of sample processing, the reproducibility of MS analyses, the reliability of metabolite identification, and the technical intricacy of data analysis. Therefore, to adopt MS-based G-Met in clinical practice, methods and protocols to overcome these problems must be established.

Several methods of sample preparation have been developed for the extraction and analysis of plasma metabolites [26–28]. Because we focus on the development of a high-throughput method for large-scale clinical and cohort studies in our G-Met protocols, we initially established a sample processing workflow that is fully automated with a STARlet liquid-handling system for the deproteinization, filtration, and dilution of samples in 96-well plates. We also established G-Met protocols using ultra-high performance liquid chromatography quadrupole time-of-flight mass spectrometry (UHPLC-QTOF/MS) and high-performance liquid chromatography Fourier transform mass spectrometry (LC-FTMS) based on high-throughput and precise analytical protocols for biological samples, as previously reported [29–32]. Then, all of the data obtained were imported into small-molecule discovery software for chromatogram alignment and feature picking. A normalization protocol was also developed to adjust for intra- and inter-batch differences based on previous reports [31,33,34].

In this study, we first evaluated our normalization protocol for the inter-batch reproducibility by analysing three plates (72 samples) using the G-Met method. Second, we evaluated the variations of the plasma metabolite concentrations caused by the storage of EDTA blood at an ambient temperature (25°C) for up to 48 hours after blood collection and identified a set of metabolites that can serve as quality markers for the evaluation of the pre-analytical storage conditions of the blood samples. Finally, we applied those quality markers to a small-scale case-control study of pregnancy-induced hypertension (PIH) plasma samples. These samples were of potentially reduced quality due to the power failure of the freezers in which they were stored for a few days following the Great East Japan Earthquake in 2011. Our present study revealed that the protocols and quality markers developed are quite useful for the discovery and development of predictive or prognostic biomarkers in large-scale clinical and cohort studies.

## Materials and Methods

### Materials

Methanol, acetonitrile and ammonium hydroxide for LC-MS were purchased from Kanto Chemical (Tokyo, Japan). Ammonium bicarbonate (1 mol L^−1^) was purchased from Cell Science & Technology Inst., Inc. (Miyagi, Japan), and formic acid for LC-MS was purchased from Wako Pure Chemical Industries (Osaka, Japan). Sterile Plasma, Human (D519-04-0050), as a reference quality control (RQC), was perched from Rockland Immunochemicals Inc. (Limerick, Pennsylvania, USA).

### Automated sample processing

Sample preparation was automated using a Microlab® STARlet robot system (Hamilton, Reno, NV), a Rack Runner (Hamilton) cooling water circulation apparatus, a Cool mini (GL Science, Tokyo, Japan), an ultrasonic bath (Kyowa Irika, Kanagawa, Japan), and an auto-controlled centrifuge (Hettich, Tuttlingen, Germany). Plasma samples were stored in Matrix 2D barcode storage tubes (Thermo Fisher Scientific, NH, USA) and were thawed in a 96-well formatted holder before transfer to the STARlet. The individual automated sample preparation steps were as follows: Study samples (1–88) and a RQC were set at positions corresponding to a 96-well format. A total of 50-μL of each plasma sample was transferred to a single well in a 96-well sample collection plate (700-μL round well; Waters Corp., Milford, MA), and 150 μL of methanol containing 0.1% formic acid was added to each plasma sample. After mixing for 5 min, the plate was transferred to the ultrasonic bath and further homogenized for 5 min. Then, the plate was transferred to the auto-controlled centrifuge. After centrifugation at 6,440 *g* for 20 min at 4°C, the plate was placed on the STARlet, and 100 μL of the supernatant was transferred to Sirocco® Protein Precipitation plates (Waters Corp.), which were then washed 3 times with 100-μL methanol containing 0.1% formic acid drawn from another 96-well plate. Then, the plates were transferred and centrifuged at 3,000 *g* for 5 min at 4°C. Finally, the plates were transferred to the STARlet, and 100 μL of water containing 0.1% formic acid was added to each study sample.

### Preparation of the quality control samples

Thirty microliters of each study sample was collected from the 96-well plate after the automated sample processing, and the samples were mixed together in a 15-mL tube. Then, the mixture was transferred into a well for the study quality control (SQC). From the SQC well, a series of dilution quality controls (dQC) were prepared by dilution with 50% methanol (water/methanol = 50/50, v/v %) containing 0.1% formic acid as follows: dilution by 2-fold (d2QC), 4-fold (d4QC), 8-fold (d8QC), and 16-fold (d16QC). Finally, the dilution plate was replicated; the original plate was used for UHPLC-QTOF/MS analysis, and the replicate plate was used for LC-FTMS analysis.

### Quality control sequences

The required frequency of quality control (QC) injections was investigated [31]. The overall run order is shown in S1 Fig. The study samples were injected in randomized run orders. Higher rates of QC injections in a sequence resulted in better data correction but at the expense of a longer total analysis time, and the best signal correction results were achieved with at least one QC injection every 2 h of sample injections. Therefore, the SQC was injected after every eight study samples (2 h). Additionally, 10 consecutive injections of SQC were made at the start of the chromatographic run to initialize the column. Finally, dQCs were injected three times at each concentration in the following order: d16QC, d8QC, d4QC, d2QC and SQC at the end of the sequence. Sample volumes of 4 and 3 μL were used for UHPLC-QTOF/MS and LC-FTMS, respectively.

### UHPLC-QTOF/MS and LC-FTMS methods

The UHPLC-QTOF/MS analysis was performed on an Acquity™ Ultra Performance LC I-class system equipped with a binary solvent manager, a sample manager, and a column heater (Waters Corp.) interfaced with a Waters Synapt G2-Si QTOF MS with electrospray ionization (ESI) operated in both positive (pos) and negative (neg) ion modes. The capillary voltage was 1.5 kV for both the pos and neg ion modes, and the cone voltage was 10 V. The source temperature was set at 120°C with a cone gas flow rate of 50 L h^−1^, a desolvation gas temperature of 500°C, and a nebulization gas flow of 1200 L h^−1^. Both the cone gas and the nebulization gas were nitrogen. The instrument was operated in high-resolution (50,000) mode and was set to acquire data over the *m/z* range of 50−1200 with a scan time of 0.1 s. The mass tolerance was 1 ppm. All mass spectral data were collected in profile mode using the MS^e^ data acquisition function to simultaneously obtain fragmentation data. In function one, a low collision energy (6 eV) was used, and in the second function, a high collision energy (ramp 10−35 eV) was used for fragmentation. Leucine-enkephalin (*m/z* 555.2771 for pos, *m/z* 554.2615 for neg) was used as a lock mass at a concentration of 2 μg mL^−1^ in 50% acetonitrile containing 0.1% formic acid infused at a flow rate of 10 μL min^−1^ via a lock spray interface. Lock-mass scans were collected every 10 s and required over 3 scans on average to perform mass correction. The instrument was calibrated before analysis with 0.5 mmol L^-1^ sodium formate solution. The data were collected using MassLynx v4.1 software (Waters Corp., Manchester, UK). LC separation was performed using a reverse-phase (C18) column (Acquity HSS T3; 150 mm × 2.1 mm i.d., 1.8 μm particle size; Waters) with a gradient elution of solvent A (water containing 0.01% formic acid) and solvent B (acetonitrile containing 0.01% formic acid) at 400 μL min^−1^. The initial condition was set at 1.0% B. The following solvent gradient was applied: 1.0% B for 1 min followed by a linear gradient to 99% B from 1 to 8 min, and then 85% B for 5 min. Subsequently, the mobile phase was immediately returned to the initial conditions and maintained for 2 min until the end of the run. The oven temperature was 40°C.

The LC-FTMS system consisted of a NANOSPACE SI-II HPLC, equipped with a dual pump system, an auto sampler, and a column oven (Shiseido, Tokyo, Japan), and a Q Exactive Orbitrap MS (Thermo Fisher Scientific, San Jose, CA) equipped with a heated-ESI-II (HESI-II) source. The pos and neg HESI-II spray voltages were 3.5 and 2.5 kV, respectively, the heated capillary temperature was 250°C, the sheath gas pressure was 50 psi, the auxiliary gas setting was 10 psi, and the heated vaporizer temperature was 300°C. Both the sheath gas and the auxiliary gas were nitrogen. The collision gas was argon at a pressure of 1.5 mTorr. The FTMS scan type was full MS/data dependent (dd)-MS^2^. The parameters of the full mass scan were as follows: a resolution of 70,000, an auto gain control target under 1 × 10^6^, a maximum isolation time of 100 ms, and an *m/z* range 70–1050. The calibration was customized for the analysis of QExactive to keep the mass tolerance of 5 ppm. The calibrated *m/z*s were 74.09643, 83.06037, 195.08465, 262.63612, 524.26496 and 1022.00341 for positive mode, and 91.00368, 96.96010, 112.98559, 265.14790, 514.28440 and 1080.00999 for negative mode. The parameters of the dd-MS^2^ scan were as follows: a resolution of 17,500, an auto gain control target under 1 × 10^5^, a maximum isolation time of 50 ms, a loop count of top 5 peaks, an isolation window of *m/z* 1.5, a normalized collision energy of 30, an under fill ratio of 5.00%, and an intensity threshold under 1 × 10^5^. The LC-FTMS system was controlled using Xcalibur 2.2 SP1.48 software (Thermo Fisher Scientific), and data were collected with the same software. The LC conditions were as reported previously [35]. LC separation was performed using a normal-phase (hydrophilic interaction chromatography, HILIC) column (ZIC®-pHILIC; 100 mm × 2.1 mm i.d., 5 μm particle size; Sequant, Darmstadt, Germany) with a gradient elution of solvent A (10 mmol L^-1^ ammonium bicarbonate in water, pH 9.2) and solvent B (acetonitrile) at 300 μL min^−1^. Solvent A was prepared by mixing 99 mL of Milli-Q (Millipore) water and 1 mL of 1 mol L^−1^ ammonium bicarbonate and was adjusted to a pH of 9.2 with 25% ammonium hydroxide (approximately 0.1 mL). The initial condition was set at 95% B, linearly decreasing to 40% B from 0 min to 4 min, and 5% B from 4 min to 6 min, and was then maintained at 85% B for 2 min. Subsequently, the mobile phase was immediately returned to the initial conditions and maintained for 7 min until the end of the run. The oven temperature was 40°C.

### Data processing

All data obtained from the four assays in the two systems in both pos and neg ion modes were processed using Progenesis QI data analysis software (Nonlinear Dynamics, Newcastle, UK) for peak picking, alignment, and normalization to produce peak intensities for retention time (*t*_R_) and *m/z* data pairs. The ranges of automatic peak picking for the C18 and HILIC assays were between 0.5 and 13 min and between 0.5 and 9 min, respectively; the ‘more 5’ mode was selected in setting the threshold for the picking sensitivity. Then, the adduct ions of each “feature” (*m/z*, *t*_R_) were deconvoluted, and these features were identified in the human metabolome database (HMDB) and Lipidmaps. The features were selected based on their coefficients of variation (CVs) with SQC samples, which were injected after every 8 study samples; features with CVs over 30% were eliminated. Features were also positively selected by the inverse correlation between the dilution fold and the peak intensity with dQC samples, along with their CVs with 3 injections of the same dQC samples. Additionally, we considered neither features above *m/z* 950 in the C18 mode nor those above *m/z* 700 in the HILIC mode because those peaks most likely represent either noise or column contaminants.

### Evaluation of the G-Met protocol

Reference human plasma, which is a mixture of plasma samples obtained from healthy volunteers, along with 24 test samples consisting of plasma samples derived from 3 individuals with 8 replicates, were placed on triplicate 96-well plates. Then, these plates were automatically processed for analysis using the STARlet robot system, as described above. SQC and dQC samples were prepared on the same plate with the test samples, and a reference QC (RQC) sample was prepared by mixing three SQC samples. The plate was replicated for the UHPLC-QTOF/MS and LC-FTMS analyses. The data normalization processes were performed as described above.

### The effects of EDTA blood storage conditions on metabolite abundance

Blood was drawn from 6 healthy volunteers by venipuncture of superficial forearm veins into 11 tubes (Venoject®II Glass Tube, EDTA-2Na) (Terumo, Tokyo, Japan). As a control specimen (0 h), one of the 11 tubes from each volunteer was immediately centrifuged at 2,000 × *g* for 20 min at 4°C, and the blood plasma was taken and frozen at -80°C in aliquots. The other tubes were centrifuged similarly for plasma separation and freezing after being kept as whole EDTA blood at either 4°C or 25°C for 3, 6, 12, 24 or 48 h. In total, 66 plasma samples were automatically processed by the STARlet robot system as described above. Then, we prepared SQC and dQCs on the same plate, and the plate was replicated. The UHPLC-QTOF/MS and LC-FTMS analysis and the data normalization processes were performed as described above. Compounds of interest were automatically identified by Progenesis QI, which was based on the isotope ratio, specific MS/MS fragmentations, and a database search. Then, we applied those quality markers to a small-scale case-control study of pregnancy-induced hypertension (PIH) plasma samples to evaluate the sample quality. This study complied with the ethical guidelines of the 1975 Declaration of Helsinki and was conducted with the approval of the Medical Ethics Committee of Tohoku University. Patients with either PIH or uncomplicated pregnancies were recruited at Tohoku University Hospital from 2008 to 2011, and written informed consent was obtained from each participant for the use of their blood samples and clinical information. EDTA blood specimens were drawn both during pregnancy and after delivery. Plasma was separated by centrifugation and was stored in aliquots at −80°C until analysis. PIH was defined as having a blood pressure >140/90 mmHg on at least 2 consecutive measurements and maternal proteinuria of at least 300 mg 24 h^−1^. Test subjects consisted of 17 patients with normal pregnancy and 26 patients with PIH. Many of the samples were of potentially reduced quality due to the power failure of the freezers in which they were stored for a few days following the Great East Japan Earthquake in 2011. The samples were automatically processed by the STARlet robot system as described above. The SQC and dQC samples were prepared on the same plate, and the plate was replicated. The UHPLC-QTOF/MS and LC-FTMS analyses and the data normalization processes were performed as described above.

### Statistical analysis

The intensities of the identified features were imported into EZinfo software (Waters) for multivariate analysis, and their relative quantities were evaluated by principal component analysis (PCA) and orthogonal partial least square-discriminant analysis (OPLS-DA). P-values were calculated using the Wilcoxon rank sum test with the Shapiro-Wilk test, and the ANOVA value (p) was calculated using Progenesis QI.

## Results and Discussion

### Evaluation of the G-Met protocol

**Fig 1:**
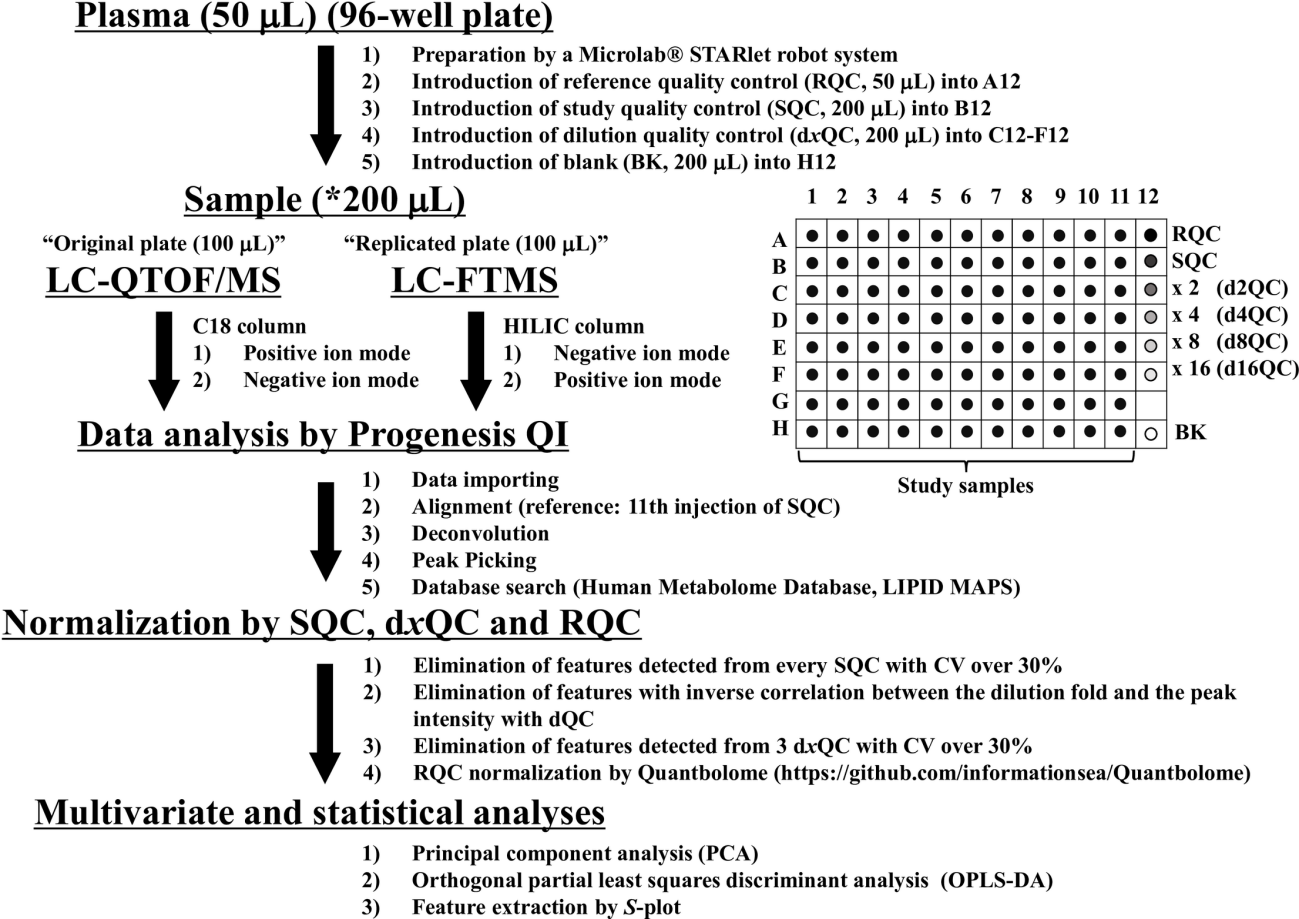
Global metabolomics protocol. Study samples (1–88) were set at well positions from A1 to H11, and an RQC was set at A12 from sample tubes using a robotic system. The SQC (study quality control) is a mixture of 30 μL of each study sample collected from the 96-well plate after automated sample processing and is introduced into B12, whereas d*x*QC is a *x*-fold dilution of SQC (*x* = 2, 4, 8, or 16) with 50% methanol (water/methanol = 50/50, v/v %) containing 0.1% formic acid and is introduced into C12-F12. BK indicates a blank sample (50% methanol containing 0.1% formic acid) introduced into H12. *All study samples were diluted 8-fold; a 150 μL of methanol containing 0.1% formic acid was added to the 50 μL volume of plasma sample. After mixing, homogenization, and centrifugation, a 100 μL volume of the supernatant was transferred, and 100 μL of water containing 0.1% formic acid was added to the sample.

**Fig 2:**
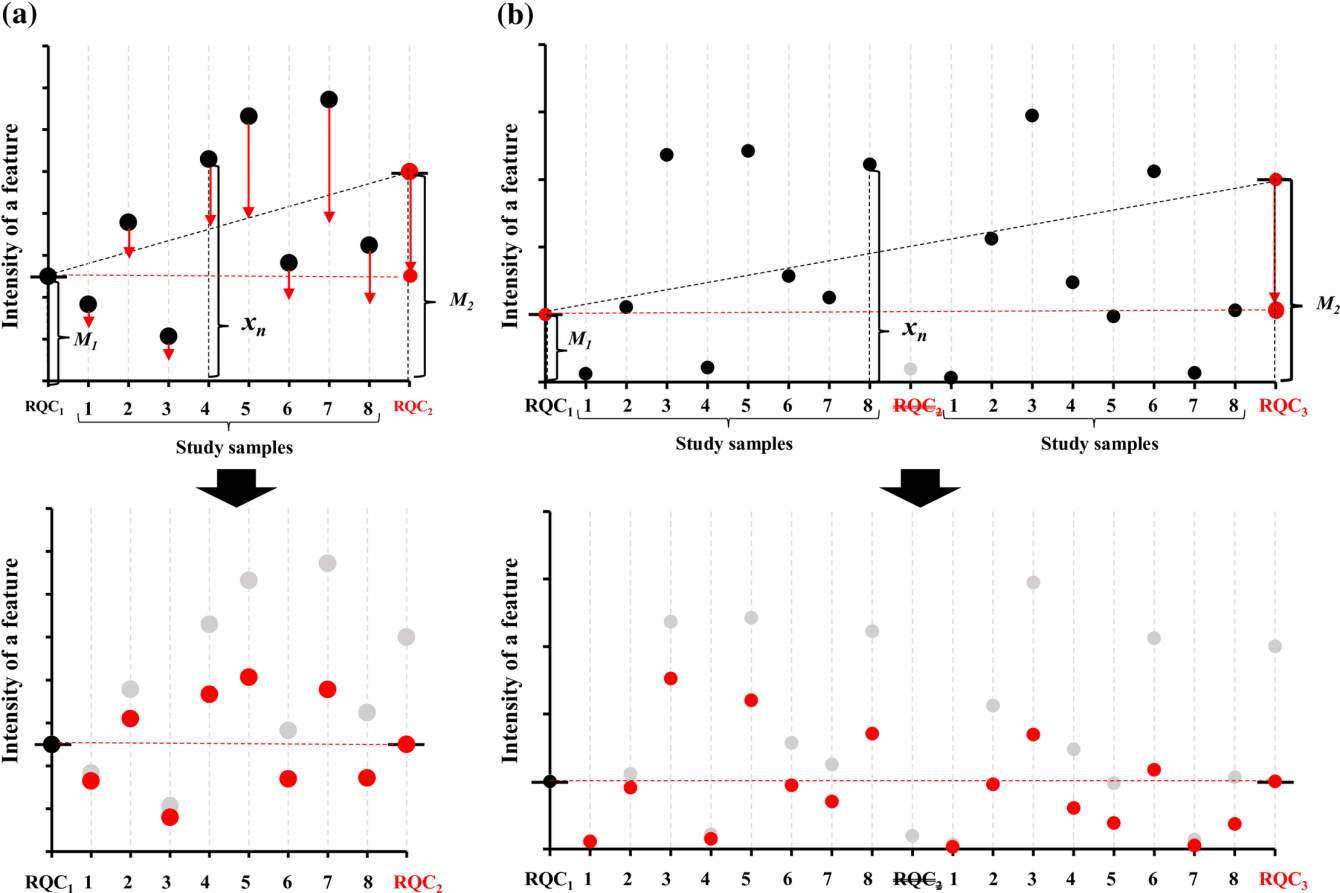
**Visualized images of RQC normalization**: two neighbouring RQC (RQC_1_ and RQC_2_) injections were deemed reliable (a) and unreliable (b). The intensities of a feature are described with black and red dots before and after normalization, respectively. ***x***_***n*:**_ Intensity of a feature (***n*** = Injection number) in a “Study sample: 1~8” between RQCs; M_1_ and M_2_: Intensity of a feature in RQC_1_ and RQC_2_ between injections in 8 study samples.

We developed a protocol for automated sample processing with a Microlab® STARlet robot system. The G-Met protocol for EDTA blood analysis is summarized in Fig 1 (see *Materials and Methods* for details). Additionally, we developed novel normalization software to correct for intra- and inter-plate variations with RQC in the G-Met analysis using an automated sample processing protocol. The software is available at (https://github.com/informationsea/Quantbolome). The visualized images of RQC normalization are shown in Fig 2A and 2B. We only considered RQC injections with less than 1,000 undetectable compounds that were selected via the feature-picking process in Progenesis QI as reliable and used those injections for the normalization processes as described below. If two neighbouring RQC injections were both deemed reliable, we then assumed that the sensitivity for each feature drifted linearly between the two neighbouring RQC injections (Fig 2A). Between two neighbouring RQC injections, we implemented a linear intensity corrector by envisioning a linear gradient of the intensity of each feature that was successfully quantified in both of the two neighbouring RQC injections. An intensity value deduced from the linear corrector between two flanking RQCs was presumed to represent the sensitivity for each feature in each study sample flanked by the two RQC injections. We then devised the following equation to calculate the normalized intensity of each feature.

$$y=x_{n}M_{1}{\left\{n(M_{2}-M_{1})/9+M_{1}\right\}}^{-1}$$

***x***_***n***_ = Intensity of a feature in a “Study sample: 1~8” between RQCs

***n*** = Injection number of a “Study sample: 1~8” between RQCs

**M**_**1**_ = Intensity of a feature in RQC_1_: before injection in 8 study samples

**M**_**2**_ = Intensity of a feature in RQC_2_: after injection in 8 study samples

If either of two neighbouring RQC injections with 1,000 or more undetectable compounds was deemed unreliable (possibly due to injection failure), or if a certain feature could not be detected or quantified in either of two neighbouring RQC injections, we excluded the RQC from the analysis or used another approach to correct for inter- and intra-plate variations due to the drift in the sensitivity of the assay (Fig 2B).

**Fig 3:**
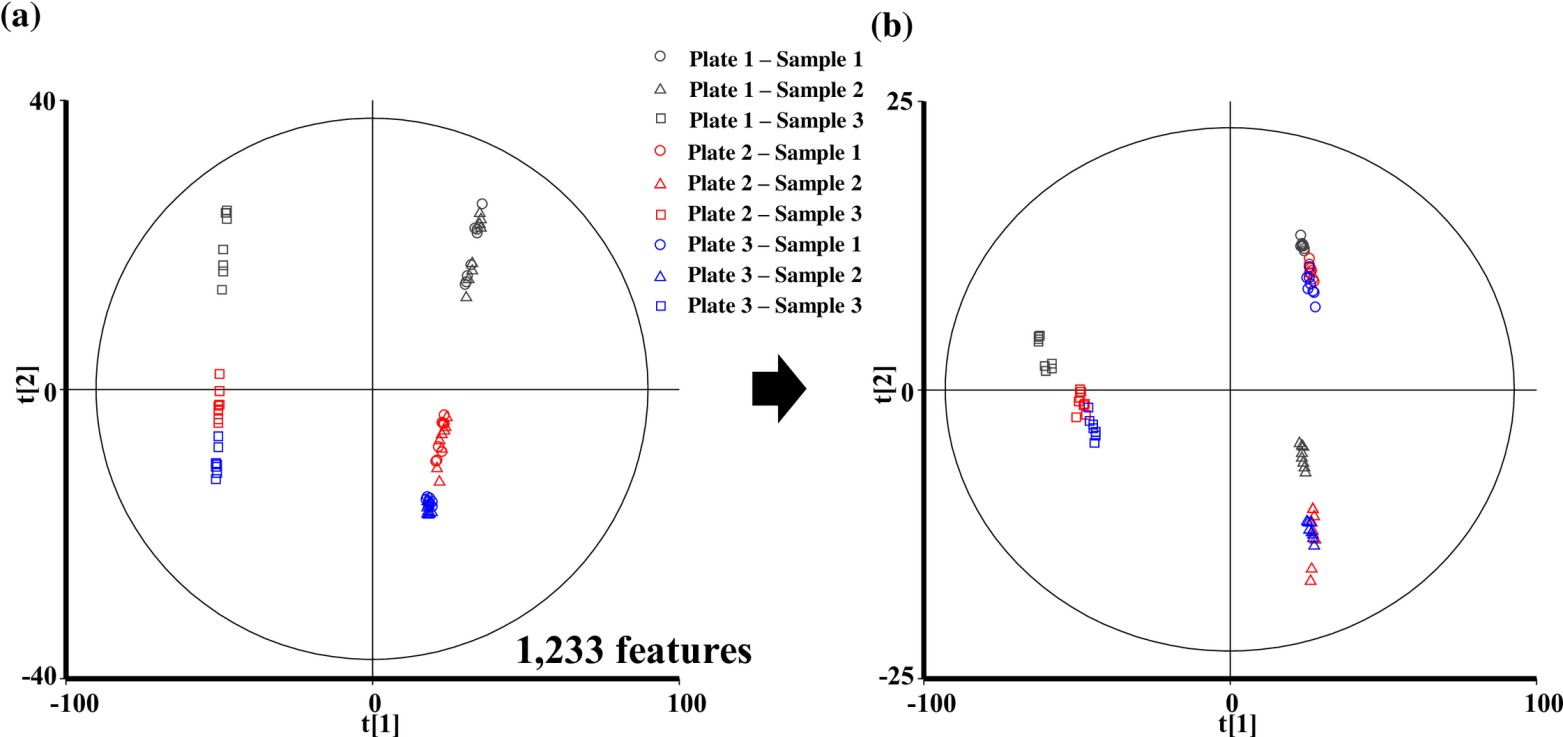
**Intra- and inter-plate variations were visualized by PCA (score plots) for C18pos assays before (a) and after normalization (b).** Samples are represented by symbols colour-coded black, red, and blue for plates 1, 2, and 3; dots, triangles and squares represent donors 1, 2, and 3, respectively. The number of features identified by each assay is indicated.

To assess the normalization process, 24 samples, consisting of plasma samples derived from 3 individuals with 8 replicates, were processed on triplicate 96-well plates. Then, the results of the relative quantification of plasma metabolites by UHPLC-QTOF/MS and LC-FTMS were examined by PCA for the visualization of sample-to-sample variations (Fig 3A, S2A–S2C Fig). The results clearly indicated larger inter-plate variations than intra-plate variations, particularly among the data evaluated by UHPLC-QTOF/MS with a C18 reverse-phase column for positive and negative ions. In contrast, there were weak variations in the data evaluated by LC-FTMS with a HILIC normal-phase column for both positive and negative ions. Therefore, we introduced the normalization processes described above to correct for inter- and intra-plate variations due to the drift in the sensitivity of the assay. The inter-plate variations in the global metabolomics analysis were significantly reduced when these normalization processes were applied, especially for the UHPLC-QTOF/MS data with a C18 reverse-phase column for positive ions (Fig 3B, S2D–S2F Fig). However, the inter-plate variations in the UHPLC-QTOF/MS data with a C18 reverse-phase column for negative ions did not work with our normalization process because of the inferior reproducibility of feature intensities at the moment. A greater QC intensity (e.g., after every three or four samples), depending on the study scale, should overcome the changes in the time of acquisition. Therefore, additional normalization might be required, or we have to improve the method for the use of a C18 reverse-phase column for negative ions. The present normalization procedure using the intermittent RQC injections was efficient for the feature-based correction of the sample-to-sample drift in the sensitivity analysis of each feature. In addition, the process of data importing was taken much time because all data were acquired in profile mode in this study. The profile data is bulkier than its centroid counterpart and its configuration difficult automated data processing of generated raw files. Therefore, the mode of data acquisition should be taken into account when a large-scale sample is analyzed by G-Met.

At the beginning of each sequence of injections, we routinely applied 10 consecutive SQC injections, which were critical for initializing the column to obtain reproducible chromatograms. The correlation of feature intensities between two consecutive SQC injections significantly improved during serial SQC injections (S3 Fig). These results indicate that the normalization procedures, along with the initial SQC injections, significantly enhanced the reproducibility and reduced sample-to-sample variations in plasma metabolite quantification.

### The effects of the EDTA blood storage conditions on plasma metabolite abundance

**Fig 4:**
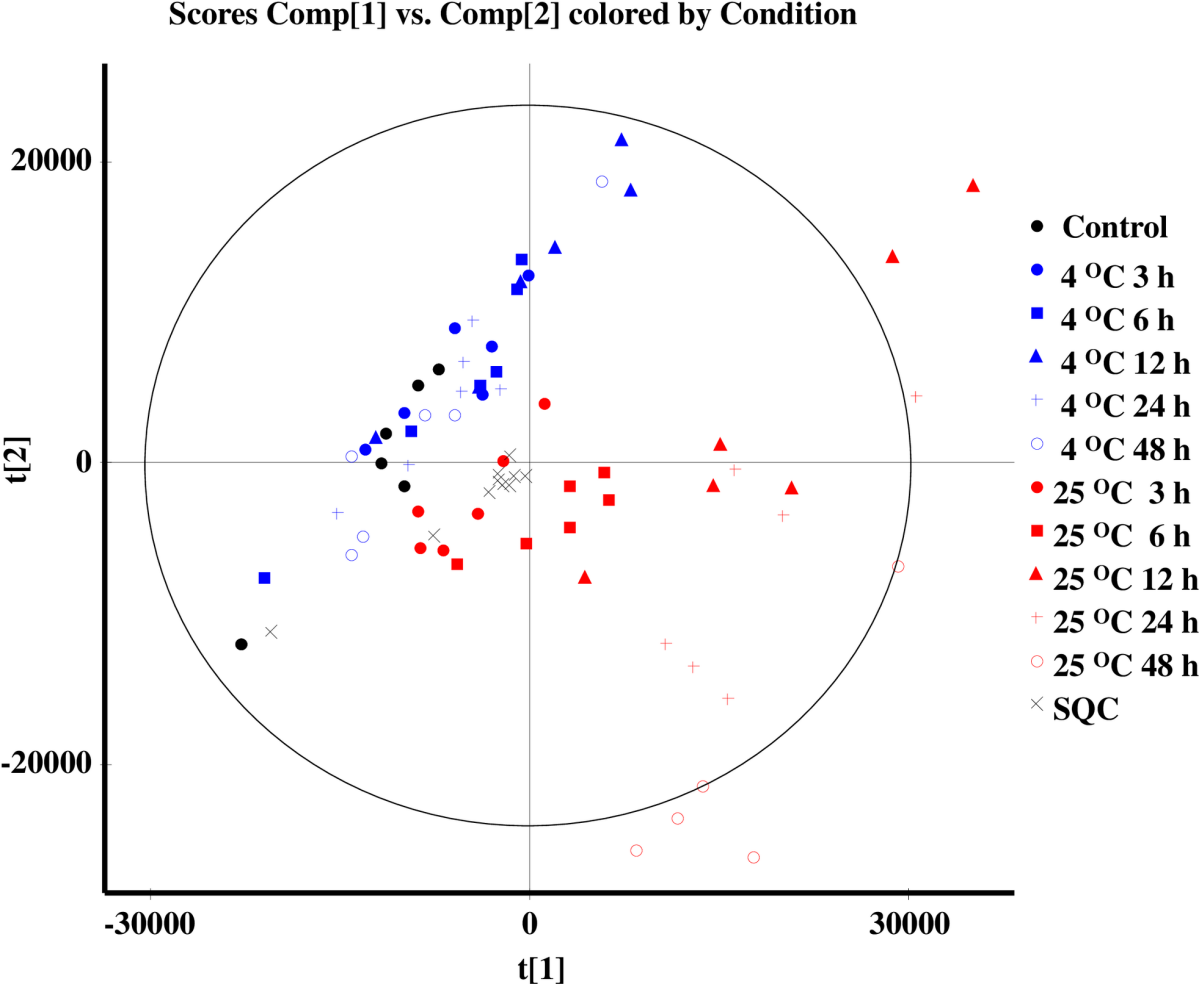
Changes in the metabolomic profiles caused by the storage of EDTA blood are visualized by PCA (score plot) based on the chemical features in the plasma samples as detected by the HILICpos assay. Sample storage conditions are represented by symbols colour-coded blue and red for 4°C and 25°C, respectively; dots, squares, triangles, crosses, and circles represent 3, 6, 12, 24, and 48 h, respectively. Control and SQC samples are represented by black dots and diagonal crosses, respectively.

We applied our normalization procedure to our study on the effects of EDTA blood storage conditions on the abundance of plasma metabolites. We used the whole sets of features that passed the selection criteria (prior to the database search), presented in S4 Fig, for multivariate analyses of plasma samples separated from EDTA blood either immediately after withdrawal from donors (control plasma) or after storage at 4°C or 25°C for 3, 6, 12, 24 or 48 h. After the alignment of *t*_R_ and the deconvolution by Progenesis QI, 8,363 features were detected by the HILICpos assay, 6,403 features by HILICneg, 9,726 features by C18pos and 5,910 features by C18neg. Approximately 20% to 50% of the features were excluded because of either high CV values (≥ 30%) with SQC injections or with three dQC injections of the same dilution fold or the disproportionality between the dQC dilution and feature intensities (e.g., the higher intensities of d8QC or d16QC injections over others, and vice versa). Then, 1,551 features detected by the HILICpos assay, 1,504 by HILICneg, 3,481 by C18pos, and 2,809 by C18neg passed all of these selection criteria and remained after the exclusion of features with *m*/*z* values of 900 or higher in the HILIC analyses. Finally, chemical identities were determined by a database search for the 795, 681, 1,038 and 342 features detected by HILICpos, HILICneg, C18pos and C18neg, respectively. The typical PCA score plot of the C18pos analysis demonstrated large variations among plasma from EDTA blood stored at 25°C (Fig 4). The plasma samples from EDTA blood stored at 25°C for 48 h are furthest in the plot from either the control plasma or the plasma samples from blood stored at 4°C. We see similar distributions of plasma from blood stored at 25°C in the PCA score plots and by other assays (S5A–S5C Fig). Then, by OPLS-DA, we selected features of plasma separated after storage at 25°C for 24 h or 48 h with larger changes in the abundance compared to the control plasma. These features were selected based on correlation values (p(corr)[1]P) greater than 0.7 (up to 1.0) and less than -0.7 (as low as -1.0) derived from the *S*-plot analysis of the OPLS-DA results of any of the four assays (S6 Fig and S7 Fig). In general, restriction in dimension p[1], not only in p(corr)[1], is required to select features in OPLS-DA for the removal of false positives in biomarker studies. In our protocol, we selected features using the SQC and dQC values to first remove the false positives, allowing us to pick up the features as much as possible, including those with low fold changes. In total, the levels of 30 endogenous metabolites, such as lysophospholipids (LPLs), dipeptides, fatty acids, succinic acid, and some amino acids, were increased in plasma by the storage of EDTA blood at 25°C, whereas the levels of 10 metabolites, including glucose, uric acid, and other amino acids, were reduced (top 40 compounds in four assays, selected in order the lowest p-values calculated by student’s *t*-test between control plasma, which was immediately centrifuged and test plasma, which was stored at 25°C for 24h and 48h, and highest fold-changes). The p-value is influenced by a number of features; including several thousands of detected features may change the significant features that are identified, which may be statistically, but not biologically, significant. Therefore, forty compounds were selected with accurate identification, including endogenous identification.

We surmised that these metabolites can be used as quality markers for the estimation of the length of EDTA blood storage at 25°C (Table 1). The data are consistent with previous reports indicating that blood LPLs increase depending on the time during which blood is exposed to ambient temperatures [23,36]. We then attempted to develop a metabolomics-based assay for the evaluation of pre-analytical conditions and the quality of plasma samples, based on the relative abundance levels of these metabolites as quality markers. The normalized abundance (NLA) of the metabolites, determined using our normalization procedure, in the control plasma samples and those from EDTA blood stored at 25°C for 3, 6, 12, 24, or 48 h, were fit to appropriate equations for one of the following trendlines by regression analysis: power *y* = a*x*^b^, linear *y* = a*x* + b, and logarithmic *y* = a*lnx* + b, in which x represents the number of hours of storage at 25°C as an independent variable and y represents the NLA of each metabolite as an explanatory variable (Table 1). The typical fitting curve of the compounds is shown in S8 Fig. Correlation coefficients (*R*^*2*^) of these trendlines were within the range of 0.717–0.999. We then devised the following equation to calculate the quality score of the plasma samples.

$$Score=CP\sum_{k=1}^{10}P_{k}+CN\sum_{k=1}^{5}P_{k}+HP\sum_{k=1}^{10}P_{k}+CP\sum_{k=1}^{15}P_{k}$$

$$P_{k}\;(point\;for\;metabolite\;k)=\frac{100}{40}\times \frac{\left\{{NLA}_{k}(48h)-{NLA}_{k}(Sample)\right\}}{{NLA}_{k}(48h)}$$

CP, C18pos; CN, C18neg; HP, HILICpos; HN, HILICneg

NLA_*k*_ (48 h): the mean NLA of metabolite *k* in plasma from standard blood specimens kept at 25°C for 48 h

NLA_*k*_ (sample): the NLA of metabolite *k* in the plasma for the quality assay

**Table 1:** Quality markers identified in this study for the evaluation of pre-analytical conditions.

	Compound Name	Room Temp. for 48 h	Trendline	Equations	Coefficient correlation
HILIC Positive ion mode	L-Histidine	Decreasing	Logarithmic	y = -1E+05ln(x) + 1E+06	R² = 0.8433
	Glycerophosphocholine	Increasing	Linear	y = 188303x - 110912	R² = 0.9979
	Oleoylcarnitine	Increasing	Power	y = 277587x0.4468	R² = 0.9854
	L-Palmitoylcarnitine	Increasing	Power	y = 170310x0.3882	R² = 0.9778
	PA(20:1/0:0)	Increasing	Power	y = 650494x0.2267	R² = 0.9875
	PC(16:0/0:0)	Increasing	Power	y = 7E+07x0.3404	R² = 0.9927
	PC(O-16:1/0:0)	Increasing	Power	y = 155226x0.4488	R² = 0.9974
	PC(O-18:0/0:0)	Increasing	Power	y = 45255x0.5283	R² = 0.9852
	PC(20:1/0:0)	Increasing	Power	y = 162933x0.4378	R² = 0.992
	PC(20:3/0:0)	Increasing	Power	y = 793974x0.2119	R² = 0.9851
HILIC Negative ion mode	L-Lactic acid	Increasing	Power	y = 1E+07x0.4748	R² = 0.9551
	Succinic acid	Increasing	Power	y = 20436x0.4232	R² = 0.9687
	Threonic acid	Increasing	Power	y = 28250x0.3007	R² = 0.9223
	L-Glyceric acid	Increasing	Power	y = 7518.8x0.1083	R² = 0.9673
	Ethylphosphate	Increasing	Power	y = 44786x0.4037	R² = 0.9601
	Beta-Citryl-L-glutamic acid	Increasing	Power	y = 36468x0.3966	R² = 0.9608
	PA(16:0/0:0)	Increasing	Power	y = 97140x0.1932	R² = 0.9824
	D-Glucose	Decreasing	Logarithmic	y = -7E+05ln(x) + 3E+06	R² = 0.9118
	D-Ribose	Decreasing	Logarithmic	y = -26418ln(x) + 118800	R² = 0.9101
	Methylsuccinic acid	Decreasing	Logarithmic	y = -26854ln(x) + 115962	R² = 0.9139
	L-Erythrulose	Decreasing	Logarithmic	y = -44871ln(x) + 203422	R² = 0.9134
	Acrylic acid	Decreasing	Logarithmic	y = -4460ln(x) + 18760	R² = 0.9108
	PE(20:4/0:0)	Decreasing	Logarithmic	y = -21310ln(x) + 93068	R² = 0.9872
	Hydroxyprolyl-Tyrosine	Increasing	Linear	y = 64.432x + 1275.6	R² = 0.9765
	Tetradecanedioic acid	Decreasing	Linear	y = -8354.1x + 61556	R² = 0.9955
C18 Positive ion mode	PC(O-18:1/0:0)	Increasing	Power	y = 24044x0.5118	R² = 0.9985
	PC(14:0/0:0)	Increasing	Power	y = 40662x0.318	R² = 0.9868
	PC(18:0/0:0)	Increasing	Power	y = 99236x0.42	R² = 0.9949
	PC(O-16:0/0:0)	Increasing	Power	y = 34731x0.3774	R² = 0.9961
	PC(16:1/0:0)	Increasing	Power	y = 119274x0.2217	R² = 0.9326
	PC(22:6/0:0)	Increasing	Power	y = 148097x0.1866	R² = 0.9653
	Allantoic acid	Increasing	Power	y = 516.24x0.1803	R² = 0.7172
	PC(20:4/0:0)	Increasing	Power	y = 118726x0.192	R² = 0.9901
	PC(0:0/20:4)	Increasing	Power	y = 1944.8x0.1386	R² = 0.9666
	PA(20:4/0:0)	Increasing	Power	y = 2000.7x0.0771	R² = 0.8869
C18 Negative ion mode	L-Glutamic acid	Increasing	Linear	y = 33.032x + 200.84	R² = 0.9867
	Taurine	Increasing	Linear	y = 103.39x + 1543.5	R² = 0.9317
	Uric acid	Decreasing	Logarithmic	y = 256.13x + 1575.8	R² = 0.9919
	Ribonic acid	Increasing	Linear	y = -22623ln(x) + 98337	R² = 0.9283
	Aspartyl-Cysteine	Decreasing	Logarithmic	y = -18480ln(x) + 249512	R² = 0.7661

Normalized abundance levels (NLAs) of metabolites in the log scale in the control plasma samples and those from EDTA blood stored at 25°C for 3, 6, 12, 24, or 48 h were fitted to appropriate equations for one of the following trendlines by regression analysis: power *y* = a*x*^b^, linear *y* = a*x* + b, and logarithmic *y* = a*lnx* + b, in which x represents hours of storage at 25°C as an independent variable and y represents the NLA of each metabolite in the log scale as an explanatory variable. The coefficient correlations were calculated for each metabolite and are indicated as the *R*^*2*^.

### Validation of sample selection with quality markers

Previously, the influences of common pre-analytical variations on the human plasma metabolome were examined by both LC-MS and GC-MS assays, and analytical methods to evaluate plasma sample quality using the abundance levels of specific marker compounds (quality markers) were established [22]. Based on these results, a metabolomics-based profiling assay using GC-MS to measure validated quality markers for the evaluation of pre-analytical conditions and the plasma sample quality was developed and is available commercially as “MxP® Quality Control Plasma” by Metanomics Health GmbH. We employed this assay to evaluate the quality of our plasma samples derived from EDTA blood stored at 25°C for various lengths of time and compared the results with our LC-MS-based assay using our own quality markers. The quality scores of the plasma samples by our LC-MS-based assay and by the GC-MS-based MxP® assay are shown in S1 Table. The quality scores obtained using our LC-MS-based assay were well correlated with those obtained using the MxP^®^ assay (*R* = 0.93) (S9 Fig).

The quality of plasma samples can directly affect metabolomic analysis results, including those for clinically relevant biomarkers. However, in most clinical settings, blood specimens drawn from patients are not immediately processed for metabolomics analyses, or only undergo plasma separation and freezing. Therefore, methods to identify deviations from a standard protocol for blood processing are essential for assuring reproducible and credible results. In a recent NMR study, glucose, lactate, and pyruvate concentration changes were most pronounced in plasma metabolites after the exposure of EDTA blood to 25°C for up to 4 h [37]. In another study using LC-MS-based G-Met and subsequent T-Met analyses, hypoxanthine, sphingosine 1-phosphate, and linolenyl carnitine were identified as the three metabolites most affected by exposure to 22°C for up to 24 h [23]. In our study applying G-Met analysis to four different LC-MS assays, we examined temporal changes of nearly 3,000 chemicals during the storage of EDTA blood at either 4°C or 25°C for up to 48 h and identified 40 metabolites as possible quality markers that were significantly affected by storage at 25°C. Our findings should contribute to the development of protocols to identify pre-analytical variations of plasma samples and thereby select optimal samples for metabolite analyses, which will be most useful for large-scale clinical or cohort studies that potentially contain inherent variations in procedures for sample collection, processing, or storage. Finally, we applied the quality markers to a small-scale clinical study. A total of 36 plasma samples were obtained from 17 patients with PIH, and 52 samples were obtained from 26 women with normal pregnancies (S2 Table). We detected 3,405 features with the C18pos assay, 2,109 features with the C18neg assay, 2,524 features with the HILICpos assay, and 2,189 features with the HILICneg assay after data normalization with SQC and dQC. Then, we applied our quality score to these samples to select high-quality samples (S3 Table). Thirty-three samples (37.5%) were excluded from this study by our quality score (< 85 points). Some of the samples showed significantly reduced quality scores, possibly due to freezer power failures in the days following the Great East Japan Earthquake in 2011. Thus, we only used samples with high scores (> 85 points) for the extraction of features that were higher or lower in abundance in the PIH samples compared with those from normal pregnancies by OPLS-DA The metabolites that were higher or lower in PIH samples compared to normal pregnancy (NP) samples are shown in S4 Table. In our study, although some potential biomarker candidates, along with known biomarkers, were extracted as metabolites that exhibited differences between PIH and NP, the significance between the 2 groups was relatively small, and the sample size was also small for biomarker detection. Therefore, the validity of the biomarker candidates that we identified is currently limited. We are planning to conduct a large-scale case-control study of PIH to evaluate these metabolites in the near future. In addition, many of the features identified in our G-Met analysis could not be assigned to any chemical identity in the available databases; more than half of the features that we obtained as biomarker candidates were ‘unknown’ despite the application of the seven golden rules [38] that automatically exclude incorrect molecular formulas, such as those containing unlikely high or low numbers of elements. Additionally, the *m/z* range (70–1050) to operate the LC-FTMS with the HILIC assays was too wide to preserve mass accuracy. Generally, the operational *m/z* range of the QExactive is commonly divided in 50–750 and 750–2,000 to keep a mass tolerance of 5 ppm. Therefore, the improvements of software and assays, which focus on the low mass range for chemical identification, are essential to realize the full potential of the G-Met analysis.

## Conclusions

The present study demonstrates that our G-Met protocol with automated high-throughput sample processing, combined with data processing by normalization with SQC, dQC, and novel software, corrected by RQC, can detect numerous features from four different assays by either UHPLC-QTOF/MS or LC-FTMS. These protocols are applicable to large-scale cohorts and clinical metabolomic profiling studies. Forty quality markers were identified by G-Met for the evaluation of plasma sample quality and were validated using another analytical platform. However, more robust validation is necessary to confirm the utility of the G-Met protocol. We plan to apply the present assay protocols, including sample evaluation using quality markers, to our large-scale cohort studies in the future.

## Supporting Information

S1 Fig Overall run order for the global metabolomics analysis. BK indicates a blank sample (50% methanol (water/methanol = 50/50, v/v %) containing 0.1% formic acid). The SQC (study quality control) is a mixture of all study samples on a plate. d*x*QC is a *x*-fold dilution of SQC (*x* = 2, 4, 8, or 16). An RQC (reference quality control) was introduced when multiple plates were processed for a single analysis.
(TIF)

S2 Fig **Intra- and inter-plate variations are visualized by PCA (score plots) for three different assays before normalization**: HILICpos (a), HILICneg (b), and C18neg (c); and after normalization: HILICpos (d), HILICneg (e), and C18neg (f). The samples are represented by symbols colour-coded black, red and blue for plates 1, 2, and 3, respectively; dots, triangles and squares represent donors 1, 2, and 3, respectively. The number of features identified by each assay is indicated.
(TIFF)

S3 Fig **The correlations of the 5,910 feature intensities detected with a C18 column for positive ions between two consecutive SQC injections over the initial 10 SQC injections (SQC1–10) and a single additional injection (SQC11)**: SQC01 *vs*. SQC02 (a), SQC05 *vs*. SQC06 (b), and SQC10 *vs*. SQC11 (c).
(TIF)

S4 Fig Results of the normalization processes for the study of the effects of EDTA blood storage conditions on the abundance of plasma metabolites. (TIF)

S5 Fig **Changes in the metabolomic profiles caused by the storage of EDTA blood are visualized by PCA (score plot) based on the chemical features of the plasma samples detected using three different assays**: HILICneg (a), C18pos (b), and C18neg (c). Sample storage conditions are represented by symbols colour-coded blue and red for 4°C and 25°C, respectively; by dots, squares, triangles, crosses, and circles represent 3, 6, 12, 24, and 48 h, respectively. Control and SQC samples are represented by black dots and diagonal crosses, respectively.
(TIF)

S6 Fig ***S*-plot analysis of OPLS-DA for extracting features in the study on the effects of EDTA blood storage conditions in four assays**: HILICpos (a). HILICneg (b), C18pos (c), and C18neg (d). Correlation values (p(corr)[1]P) greater than 0.7 (up to 1.0) and less than -0.7 (as low as -1.0) for the selected features are shown as red lines.
(TIF)

S7 Fig Features that were separated in plasma after storage at 25°C for 24 or 48 h had larger abundance changes compared to the control plasma. These features were selected based on correlation values (p(corr)[1]P) greater than 0.7 (up to 1.0) and less than -0.7 (as low as -1.0) derived from the *S*-plot analysis of OPLS-DA in four assays: C18pos, C18neg, HILICpos and HILICneg.
(PDF)

S8 Fig Changes in the normalized abundance (NLA) of representative plasma metabolites due to the storage of EDTA blood at 25°C. NLAs of plasma metabolites in the log scale after storage at 25°C for 0 (control), 3, 6, 12, 24, and 48 h were fitted to appropriate equations for one of the following trendlines: power *y* = a*x*^b^, linear *y* = a*x* + b, or logarithmic *y* = a*lnx* + b (x represents hours of storage, and y represents the NLA of each metabolite in the log scale).
(TIF)

S9 Fig Correlations between the quality scores obtained with the EDTA blood samples using the GC-MS assay and the LC-MS assay. (TIF)

S1 Table Comparison of quality scores for stability samples by our LC-MS-based assay and those by GC-MS-based MxP^®^ assay. LC-MS score was summarized with four assays.
(TIF)

S2 Table Numbers of the study participants with pregnancy induced hypertension or normal pregnancy, and numbers of blood specimens used for this study, collected prior to, at the time of, or after the delivery. (TIF)

S3 Table Quality scores for NP and PIH samples obtained by our LC-MS-based assay. **Sample quality was evaluated by the mean quality**; more than 85 points indicated high quality. *B2, before birth > 1w; B1, before birth in 1 w; B0, birth; A1, after birth in 1 w; A2, after birth < 1 w; UK, unknown.
(TIF)

S4 Table List of metabolites higher or lower in pregnancy induced hypertension than in normal pregnancy. The samples were used prior to or at the time of delivery to identify predictive biomarkers for PIH. Features were selected with a value of p(corr)[1]P (correlation) greater than 0.55, or lower than -0.55 (below to -1.0) on an S-plot, and a value of “Anova (p) <0.05” calculated by Progenesis QI between NP and PIH.
(TIFF)
