# Supplementary figures and images for: Establishment of Protocols for Global Metabolomics by LC-MS for Biomarker Discovery
Source: PLoS One. 2016 Aug 31;11(8):e0160555. doi: 10.1371/journal.pone.0160555 (PMC5006994; doi:10.1371/journal.pone.0160555)

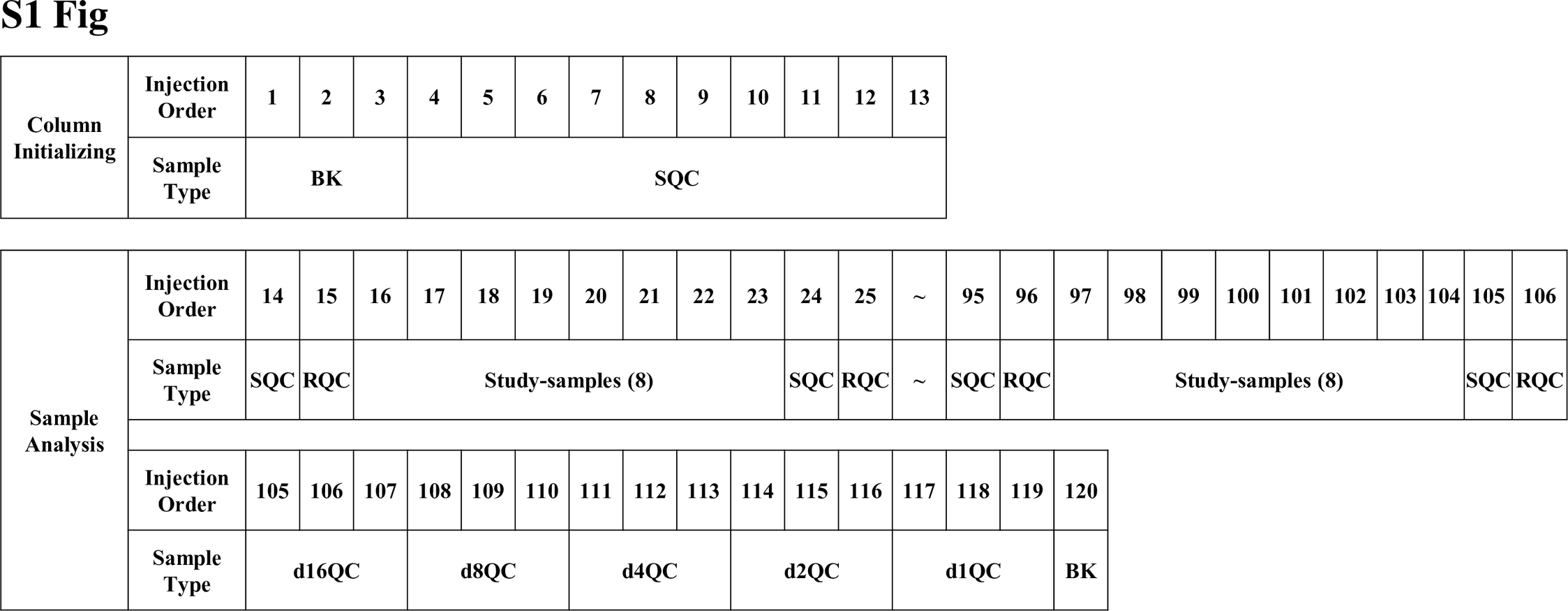

Supplement: S1 Fig — BK indicates a blank sample (50% methanol (water/methanol = 50/50, v/v %) containing 0.1% formic acid). The SQC (study quality control) is a mixture of all study samples on a plate. dxQC is a x-fold dilution of SQC (x = 2, 4, 8, or 16). An RQC (reference quality control) was introduced when multiple plates were processed for a single analysis. (TIF) [file pone.0160555.s001.tif]

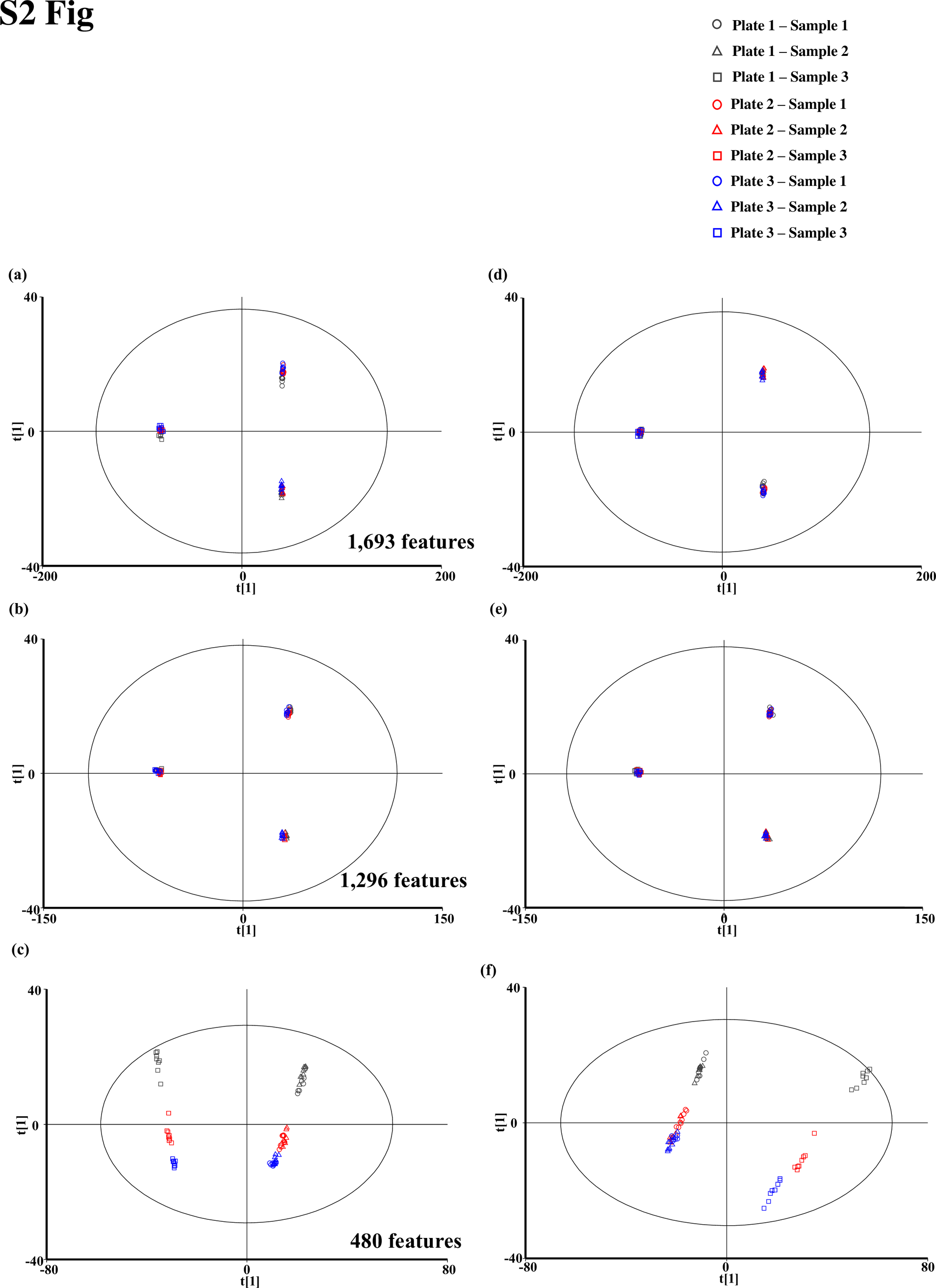

Supplement: S2 Fig — Intra- and inter-plate variations are visualized by PCA (score plots) for three different assays before normalization: HILICpos (a), HILICneg (b), and C18neg (c); and after normalization: HILICpos (d), HILICneg (e), and C18neg (f). The samples are represented by symbols colour-coded black, red and blue for plates 1, 2, and 3, respectively; dots, triangles and squares represent donors 1, 2, and 3, respectively. The number of features identified by each assay is indicated. (TIFF) [file pone.0160555.s002.tiff]

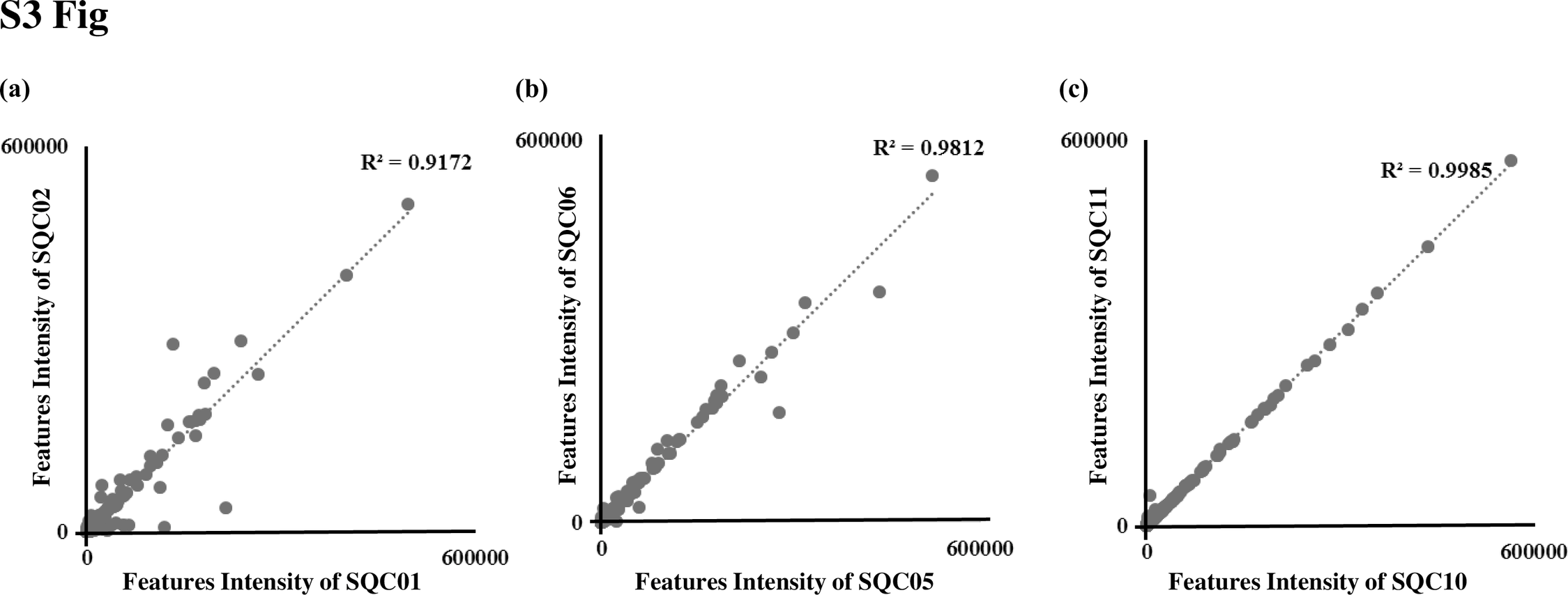

Supplement: S3 Fig — The correlations of the 5,910 feature intensities detected with a C18 column for positive ions between two consecutive SQC injections over the initial 10 SQC injections (SQC1–10) and a single additional injection (SQC11): SQC01 vs. SQC02 (a), SQC05 vs. SQC06 (b), and SQC10 vs. SQC11 (c). (TIF) [file pone.0160555.s003.tif]

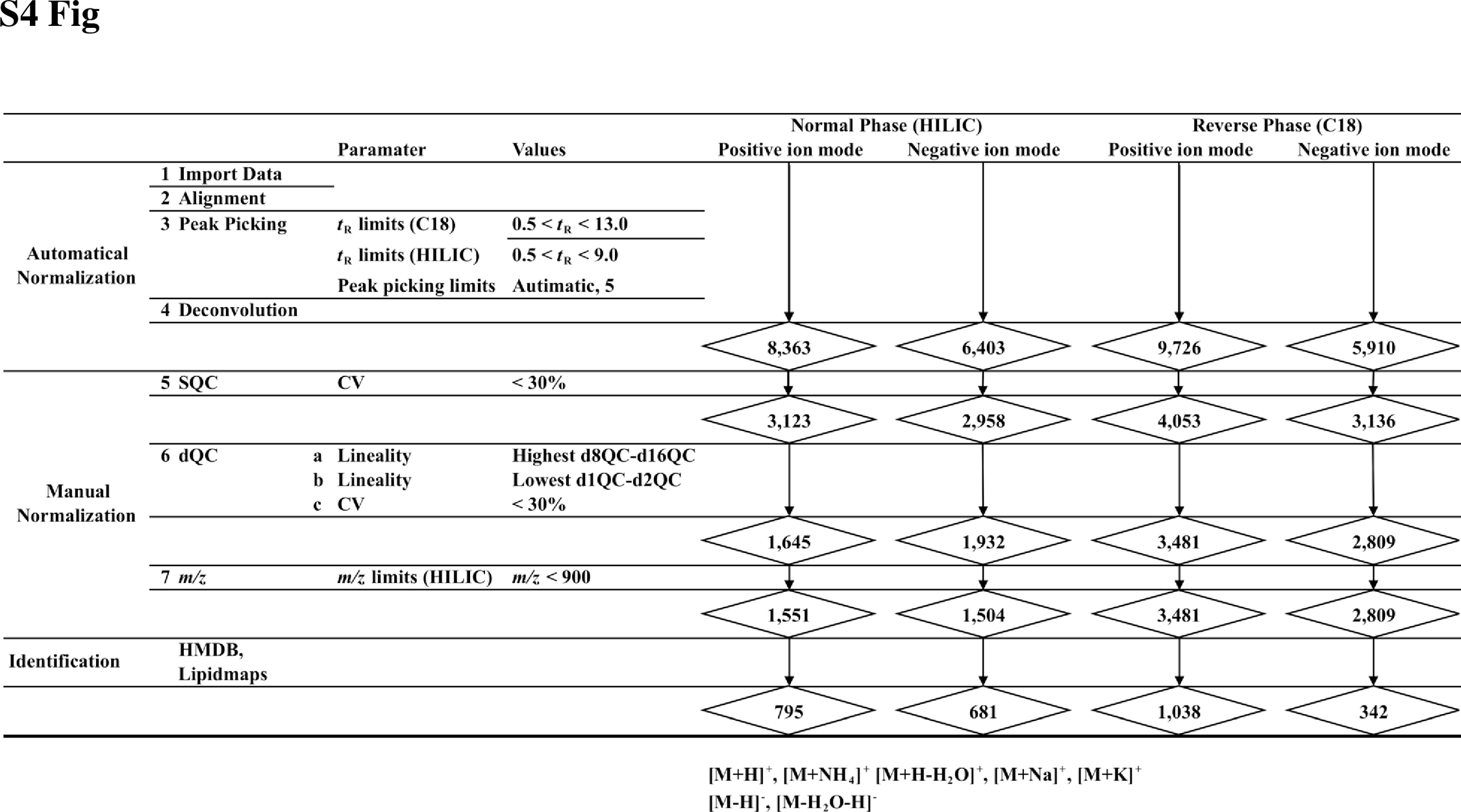

Supplement: S4 Fig — (TIF) [file pone.0160555.s004.tif]

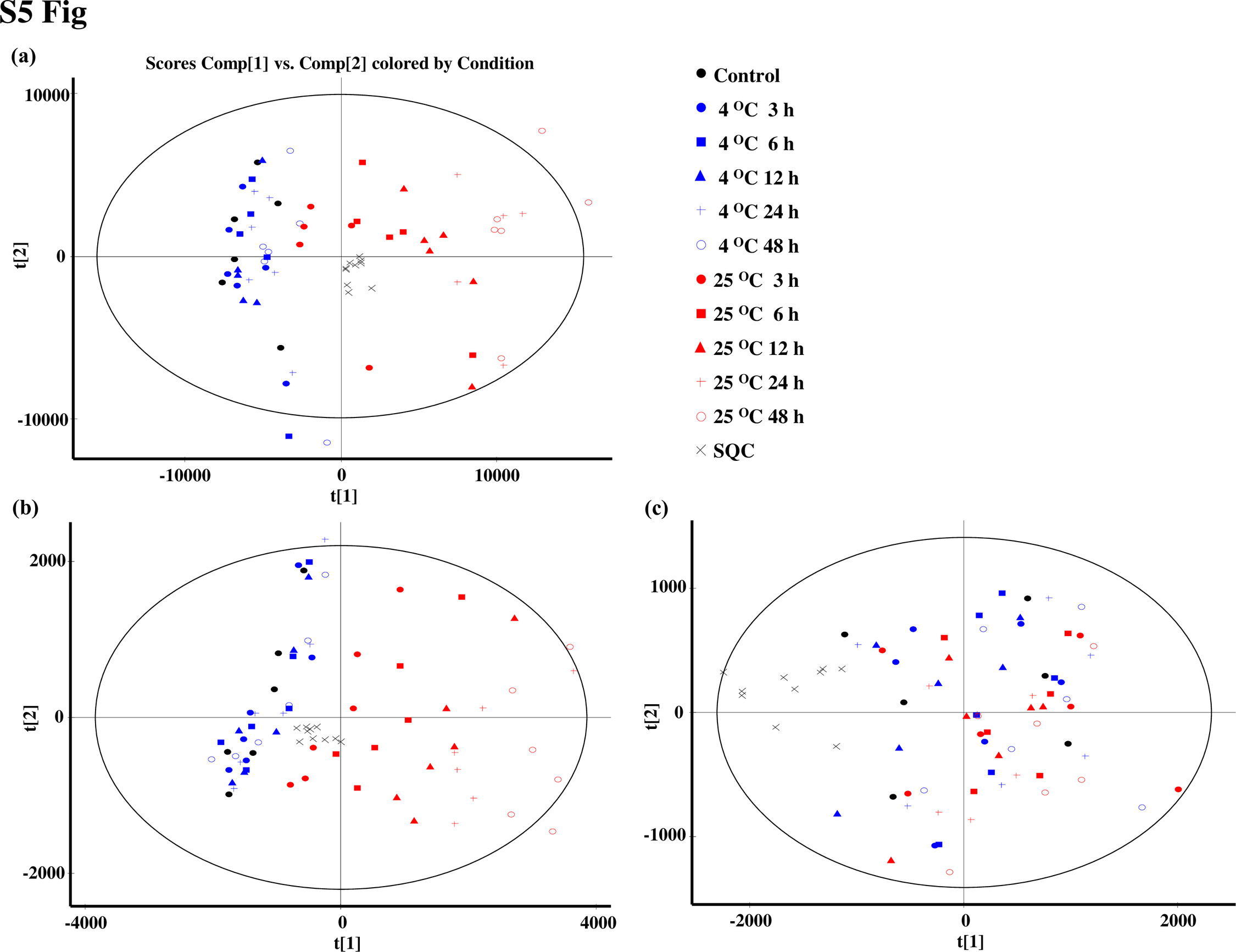

Supplement: S5 Fig — Changes in the metabolomic profiles caused by the storage of EDTA blood are visualized by PCA (score plot) based on the chemical features of the plasma samples detected using three different assays: HILICneg (a), C18pos (b), and C18neg (c). Sample storage conditions are represented by symbols colour-coded blue and red for 4°C and 25°C, respectively; by dots, squares, triangles, crosses, and circles represent 3, 6, 12, 24, and 48 h, respectively. Control and SQC samples are represented by black dots and diagonal crosses, respectively. (TIF) [file pone.0160555.s005.tif]

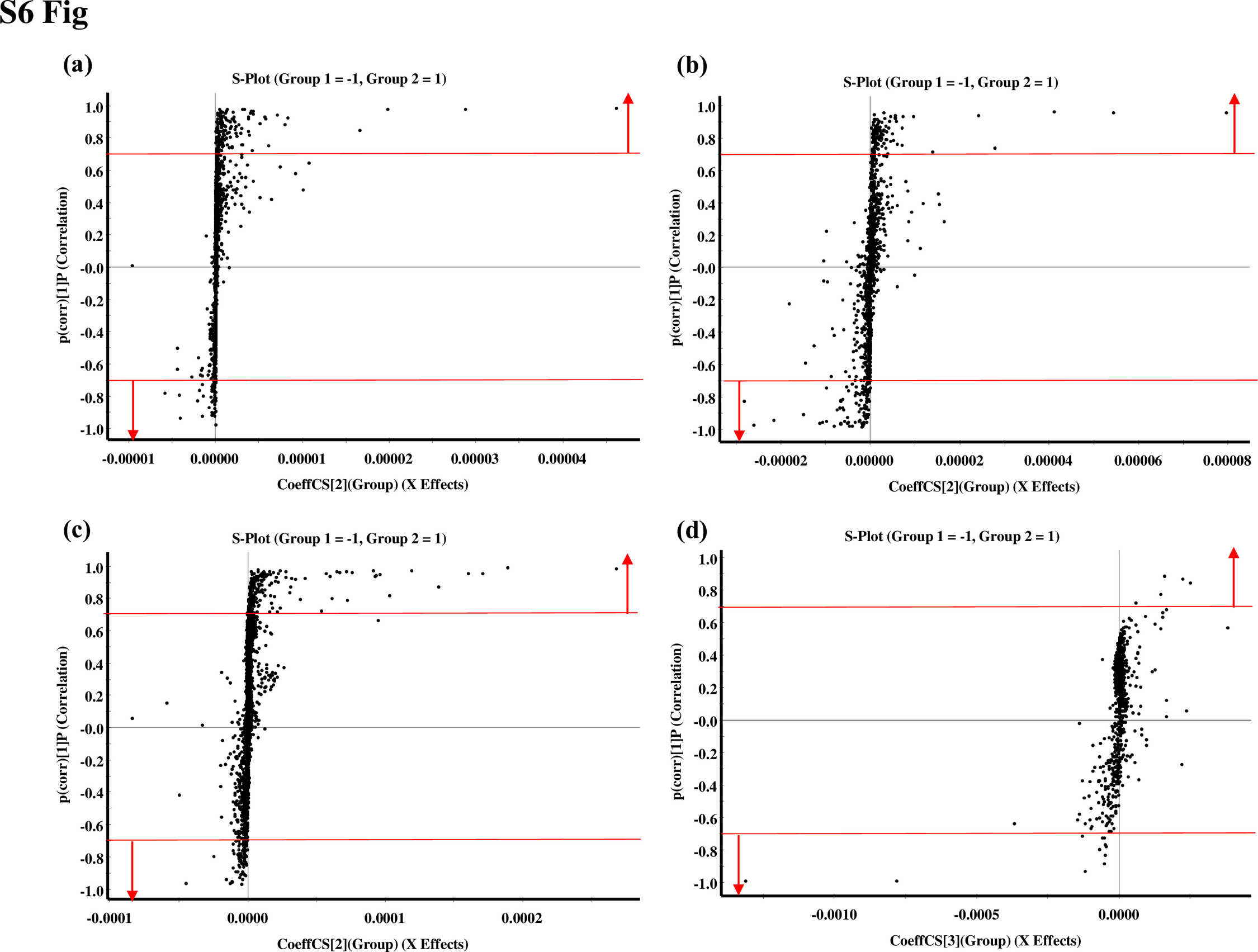

Supplement: S6 Fig — S-plot analysis of OPLS-DA for extracting features in the study on the effects of EDTA blood storage conditions in four assays: HILICpos (a). HILICneg (b), C18pos (c), and C18neg (d). Correlation values (p(corr)[1]P) greater than 0.7 (up to 1.0) and less than -0.7 (as low as -1.0) for the selected features are shown as red lines. (TIF) [file pone.0160555.s006.tif]

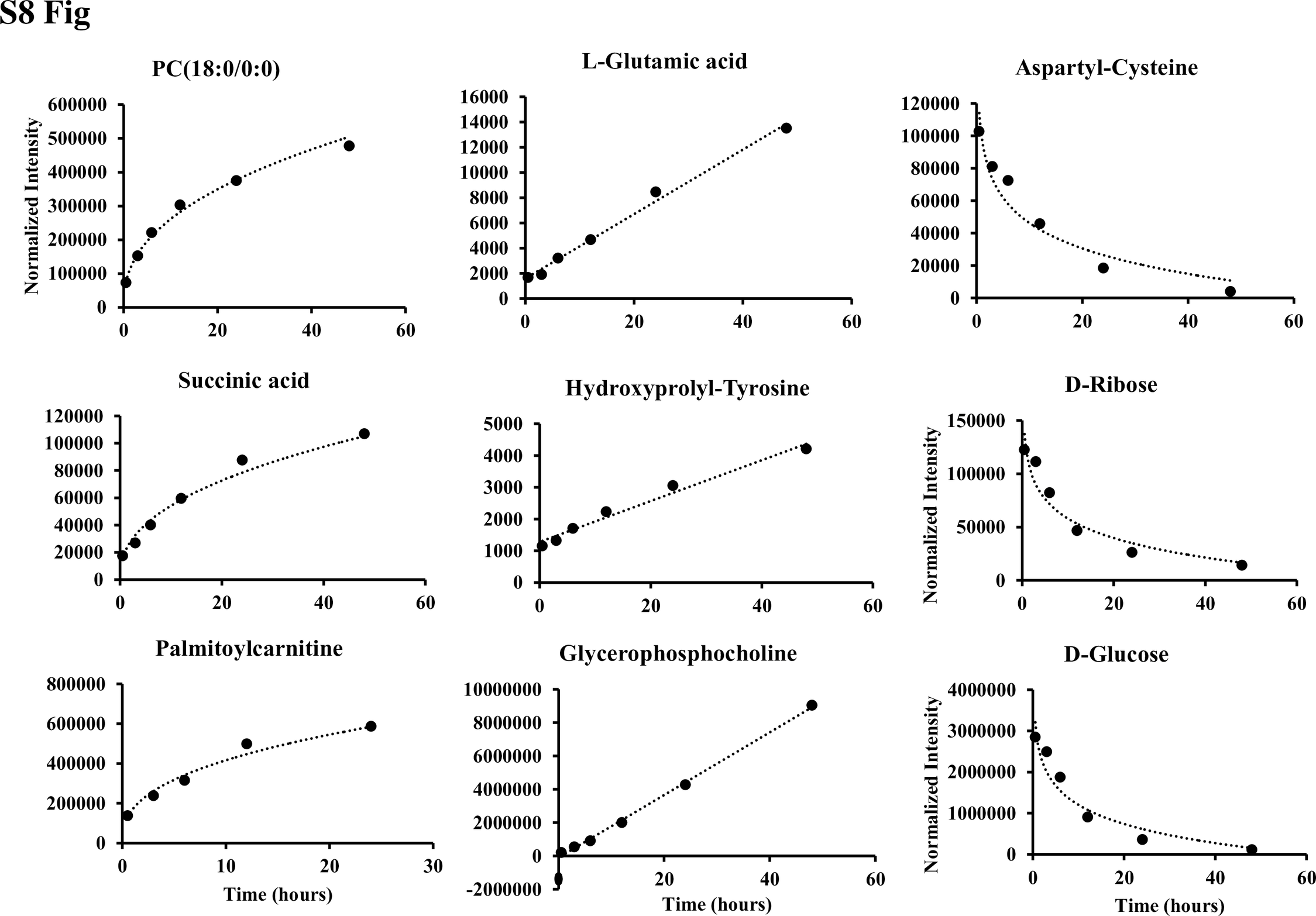

Supplement: S8 Fig — NLAs of plasma metabolites in the log scale after storage at 25°C for 0 (control), 3, 6, 12, 24, and 48 h were fitted to appropriate equations for one of the following trendlines: power y = axb, linear y = ax + b, or logarithmic y = alnx + b (x represents hours of storage, and y represents the NLA of each metabolite in the log scale). (TIF) [file pone.0160555.s008.tif]

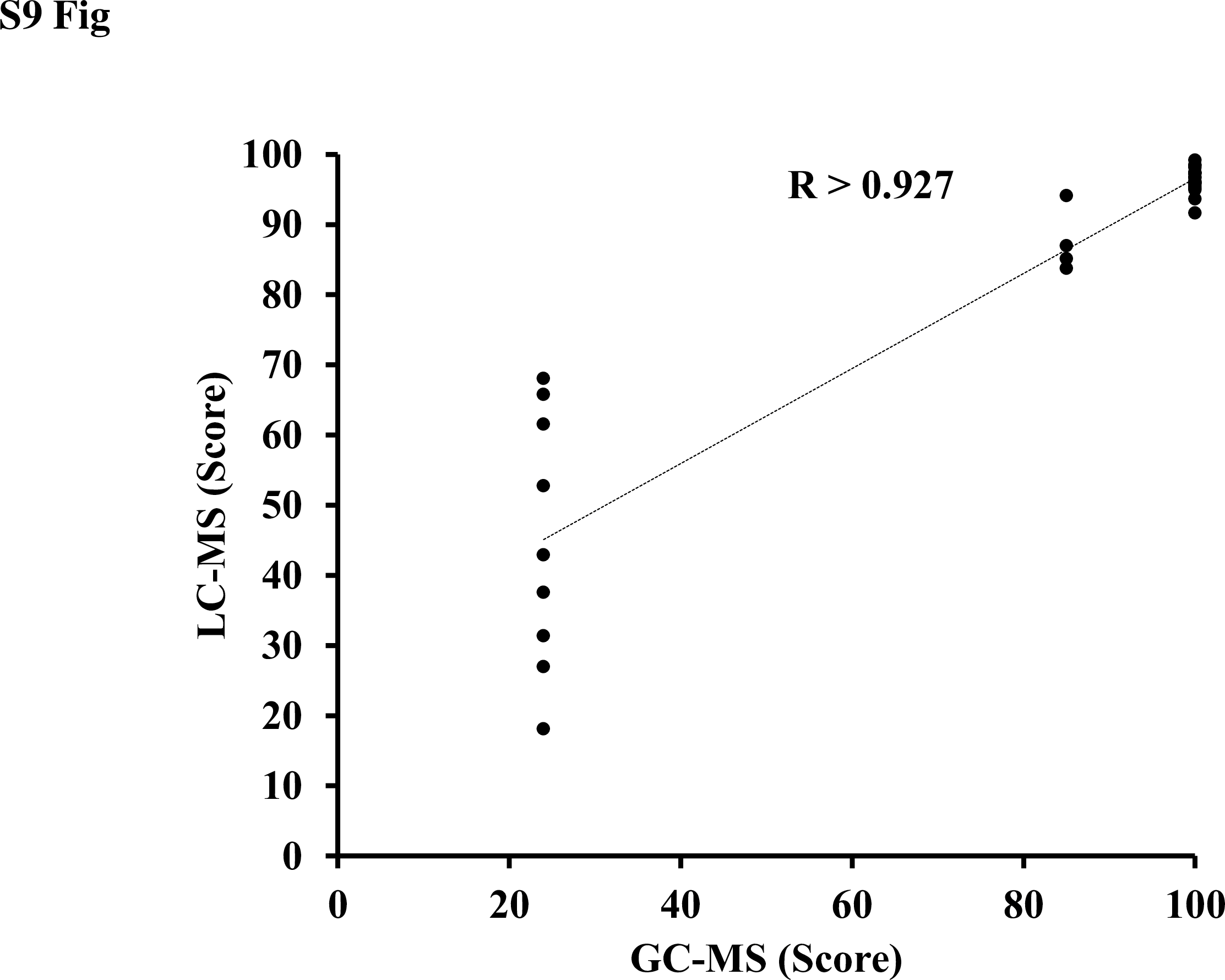

Supplement: S9 Fig — (TIF) [file pone.0160555.s009.tif]

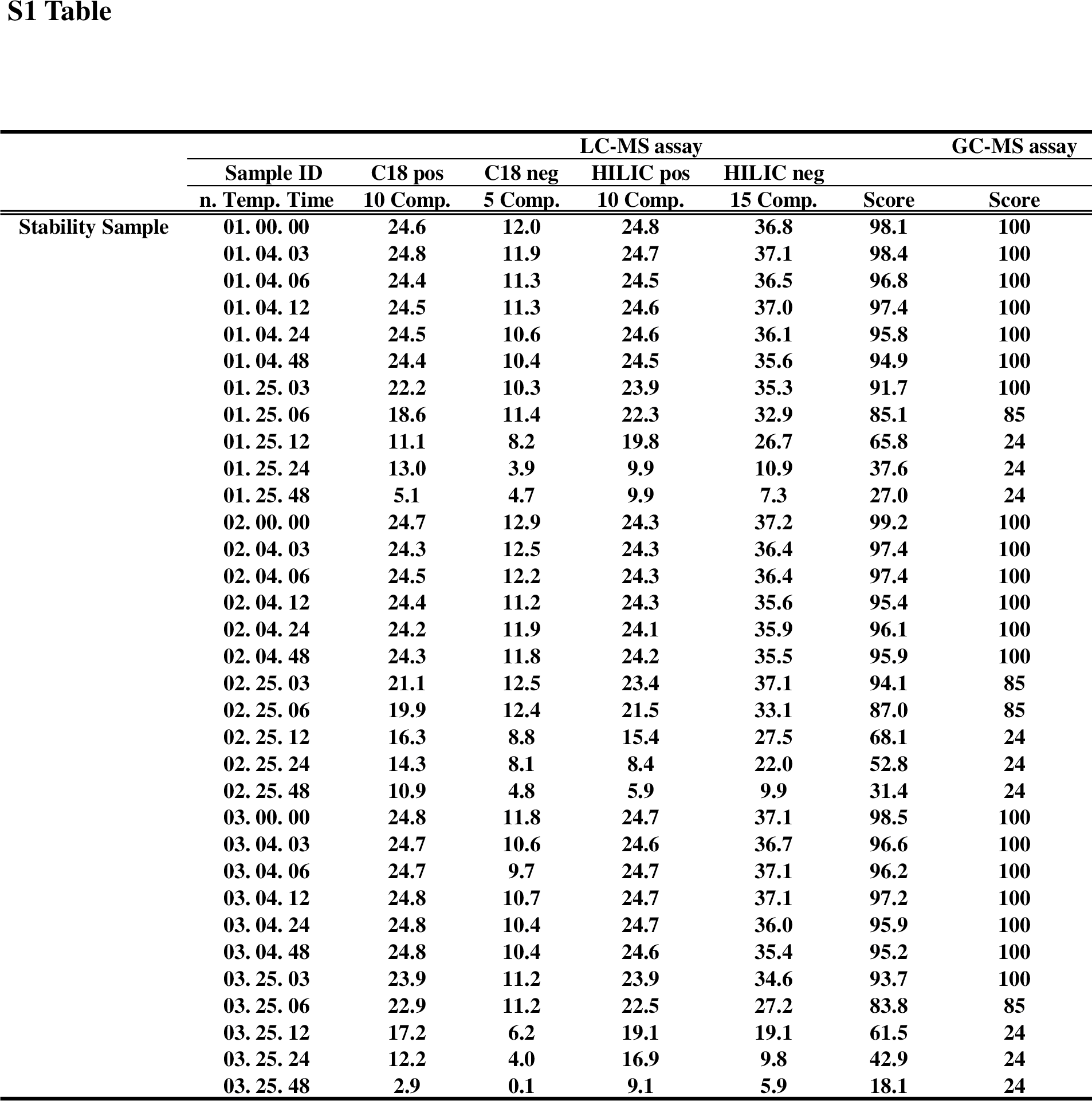

Supplement: S1 Table — LC-MS score was summarized with four assays. (TIF) [file pone.0160555.s010.tif]

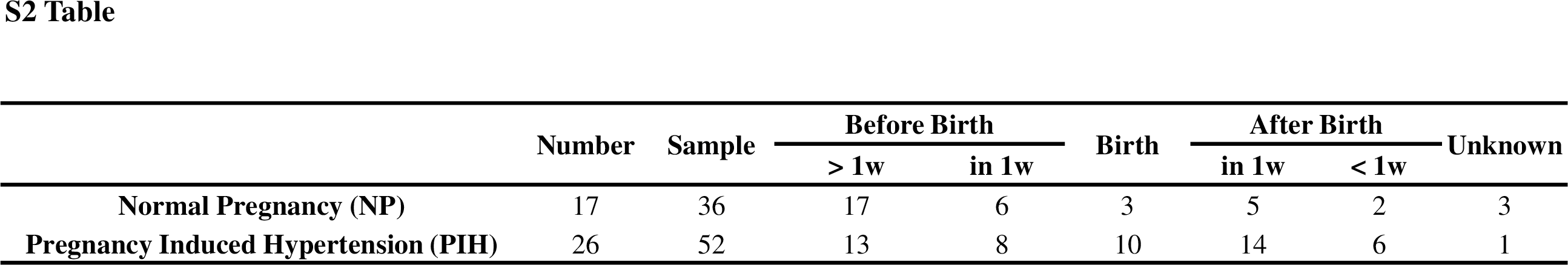

Supplement: S2 Table — (TIF) [file pone.0160555.s011.tif]

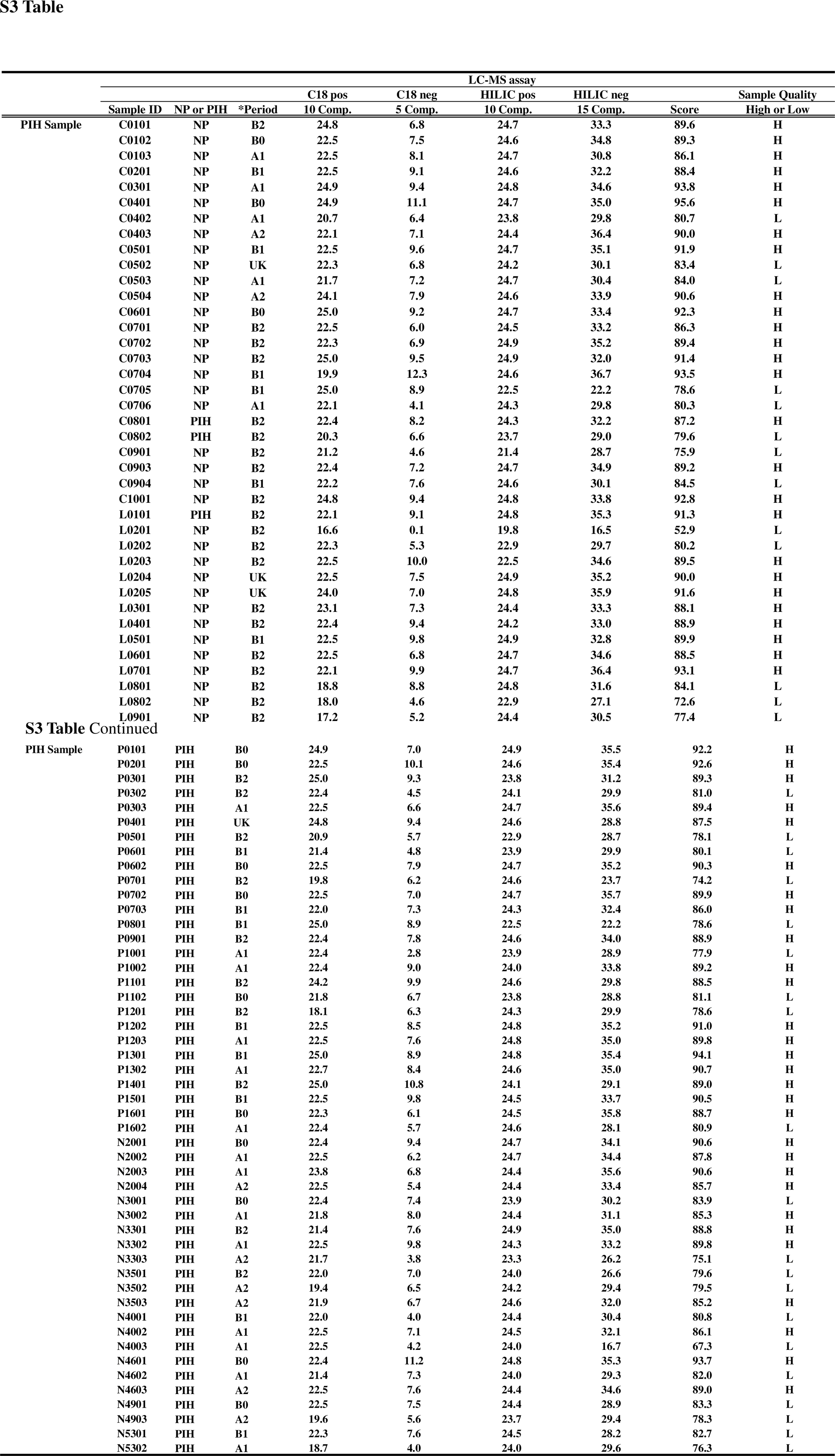

Supplement: S3 Table — Sample quality was evaluated by the mean quality; more than 85 points indicated high quality. *B2, before birth > 1w; B1, before birth in 1 w; B0, birth; A1, after birth in 1 w; A2, after birth < 1 w; UK, unknown. (TIF) [file pone.0160555.s012.tif]

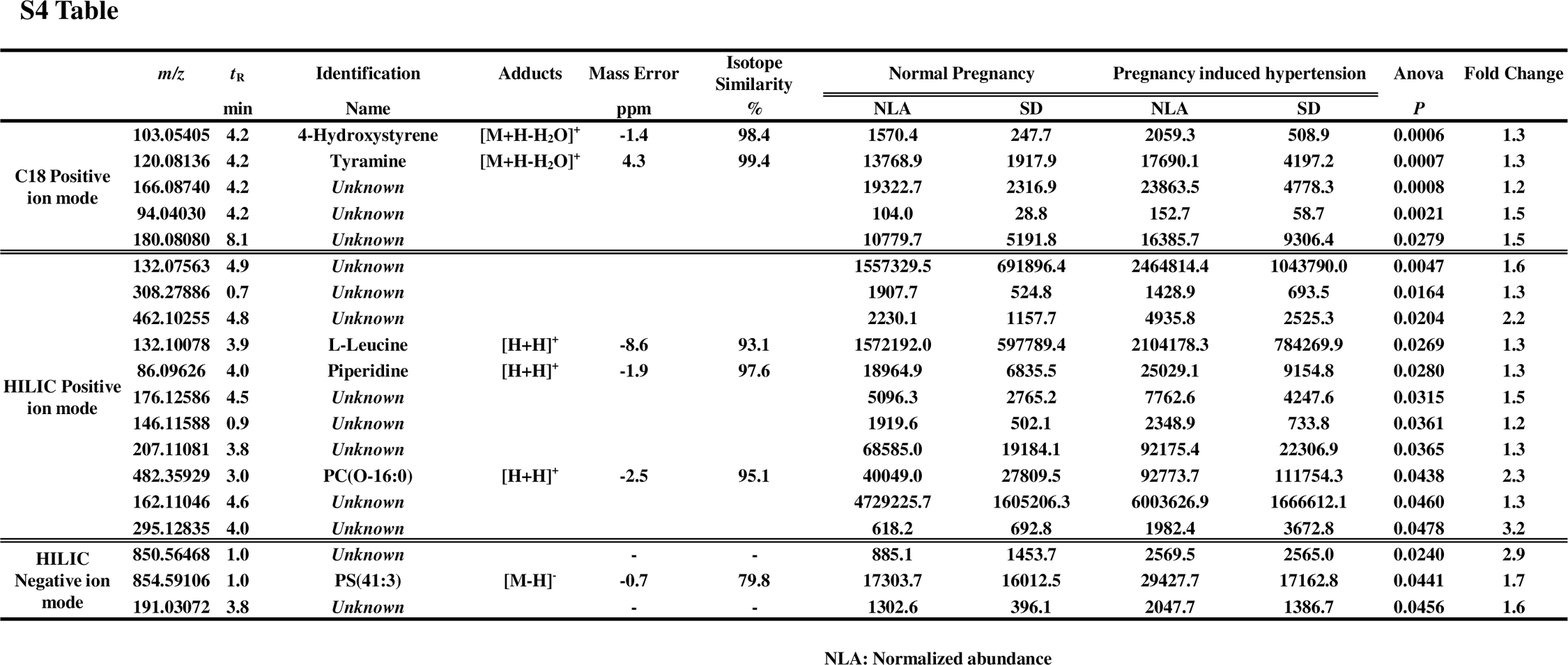

Supplement: S4 Table — The samples were used prior to or at the time of delivery to identify predictive biomarkers for PIH. Features were selected with a value of p(corr)[1]P (correlation) greater than 0.55, or lower than -0.55 (below to -1.0) on an S-plot, and a value of “Anova (p) <0.05” calculated by Progenesis QI between NP and PIH. (TIFF) [file pone.0160555.s013.tiff]
