# Supplementary material for: Establishment of Protocols for Global Metabolomics by LC-MS for Biomarker Discovery
Source: PLoS One. 2016 Aug 31;11(8):e0160555. doi: 10.1371/journal.pone.0160555 (PMC5006994; doi:10.1371/journal.pone.0160555)
Supplement: S7 Fig — These features were selected based on correlation values (p(corr)[1]P) greater than 0.7 (up to 1.0) and less than -0.7 (as low as -1.0) derived from the S-plot analysis of OPLS-DA in four assays: C18pos, C18neg, HILICpos and HILICneg. (PDF) [file pone.0160555.s007.pdf]

S7 Fig

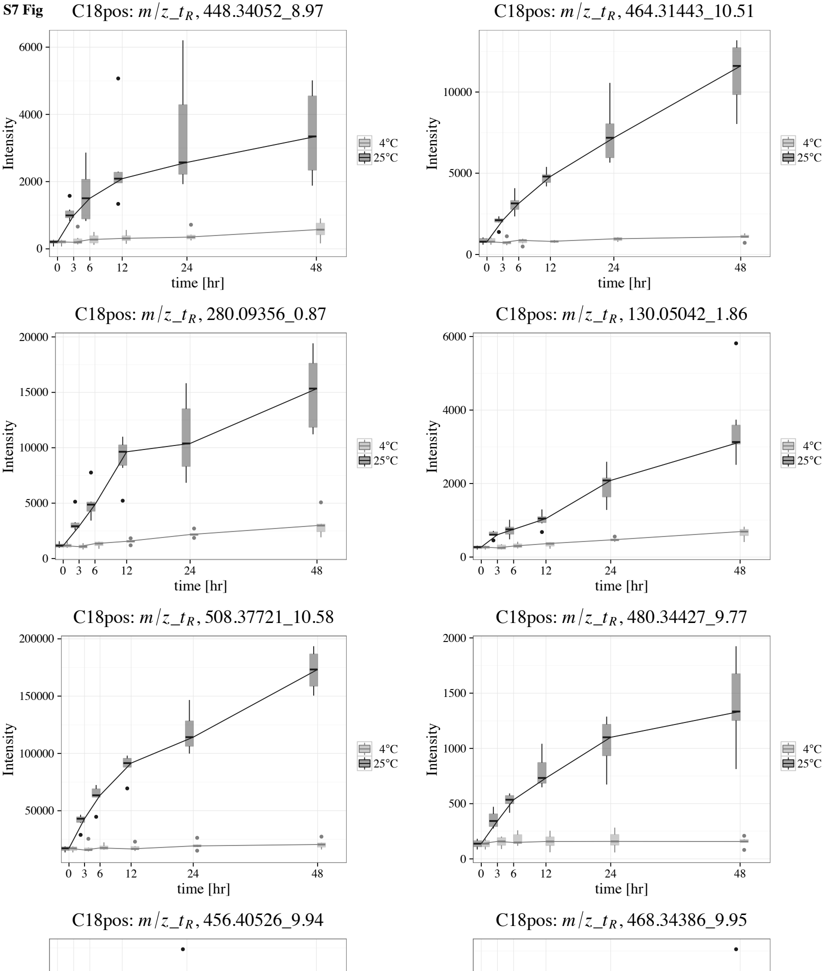
C18pos: m/z_t_R, 448.34052_8.97
C18pos: m/z_t_R, 464.31443_10.51
C18pos: m/z_t_R, 280.09356_0.87
C18pos: m/z_t_R, 130.05042_1.86
C18pos: m/z_t_R, 508.37721_10.58
C18pos: m/z_t_R, 480.34427_9.77
C18pos: m/z_t_R, 456.40526_9.94
C18pos: m/z_t_R, 468.34386_9.95
Intensity
time [hr]
0 3 6 12 24 48
4°C
25°C

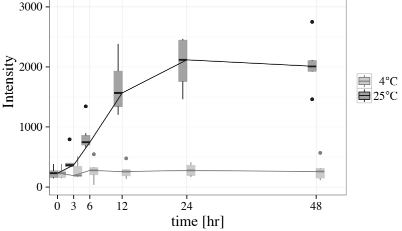

C18pos: m/z_t_R, 524.37165_11.06

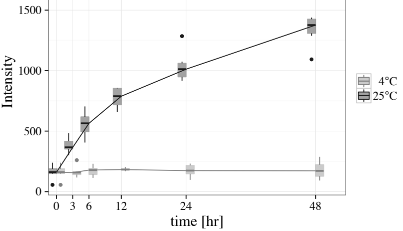

C18pos: m/z_t_R, 446.32659_8.73

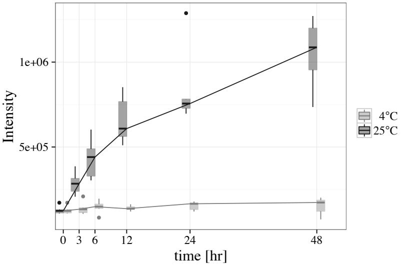

C18pos: m/z_t_R, 506.35904_10.02

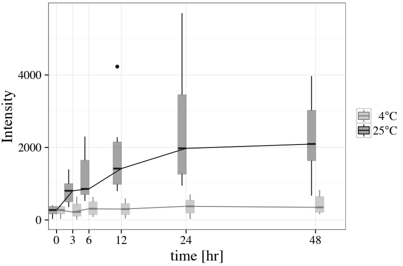

C18pos: m/z_t_R, 1017.68516_10.18

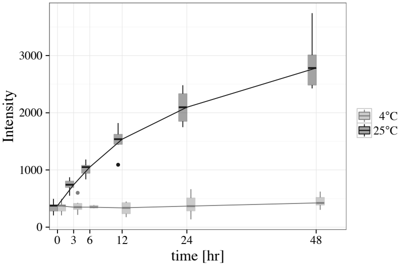

C18pos: m/z_t_R, 546.35411_10.79

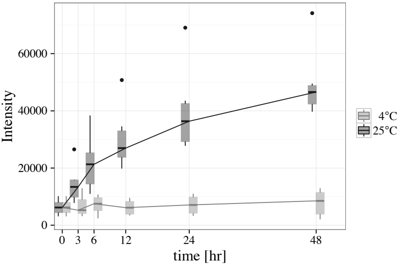

C18pos: m/z_t_R, 506.36043_10.54

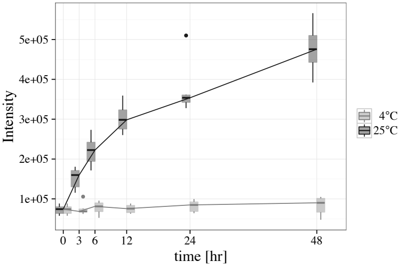

C18pos: m/z_t_R, 510.35731_10.53

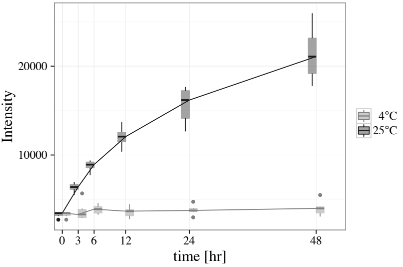

C18pos: m/z_t_R, 511.35702_10.28

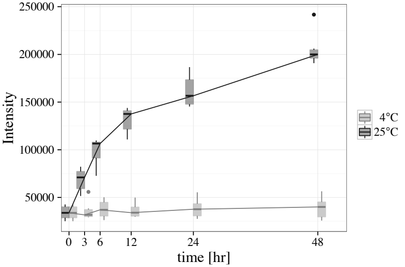

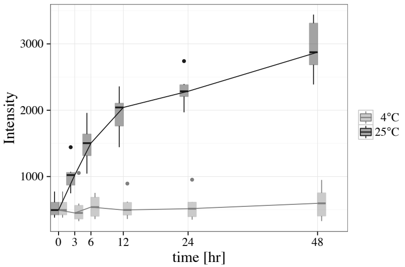

C18pos: m/z_t_R, 438.29851_10.31

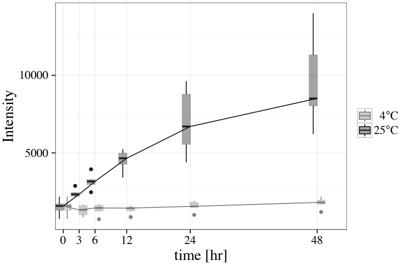

C18pos: m/z_t_R, 482.36166_10.37

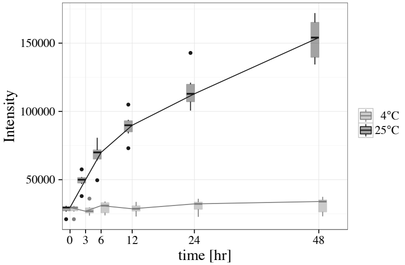

C18pos: m/z_t_R, 558.35657_10.69

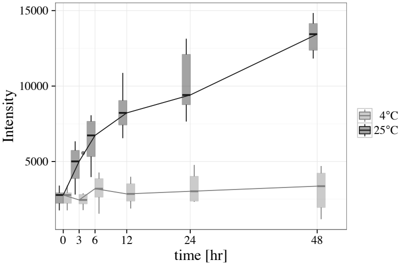

C18pos: m/z_t_R, 482.32514_9.73

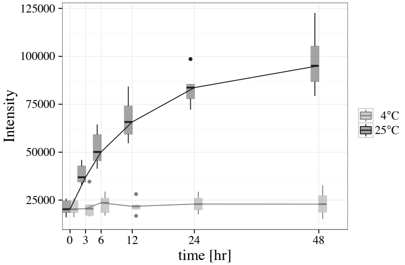

C18pos: m/z_t_R, 512.34750_10.37

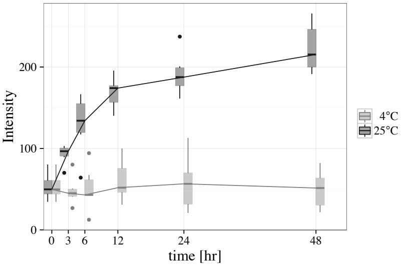

C18pos: m/z_t_R, 480.34544_10.35

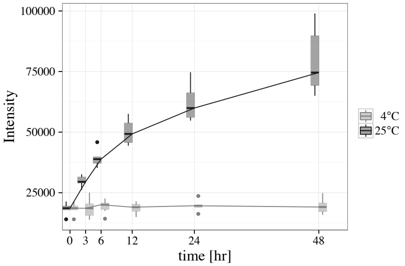

C18pos: m/z_t_R, 468.30969_9.35

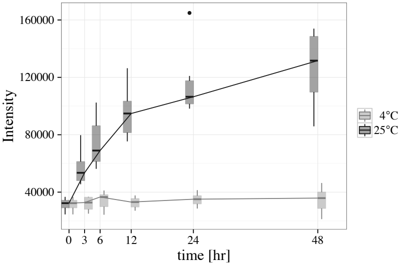

C18pos: m/z_t_R, 548.37031_10.52

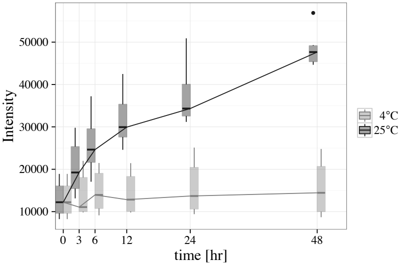

C18pos: m/z_t_R, 494.32532_9.58

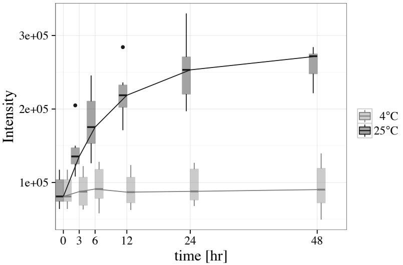

C18pos: m/z_t_R, 424.34214_8.97

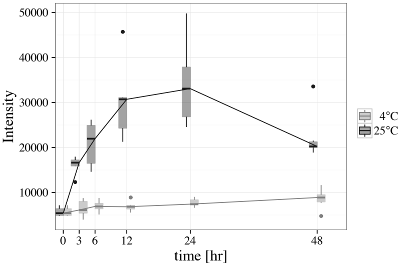

C18pos: m/z_t_R, 283.13156_9.35

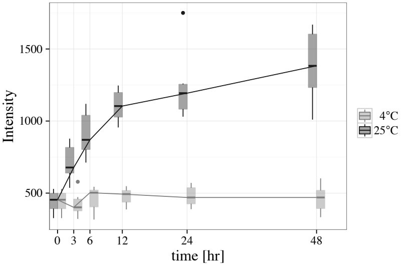

C18pos: m/z_t_R, 508.34039_9.94

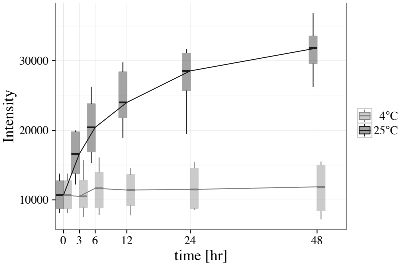

C18pos: m/z_t_R, 572.36754_10.38

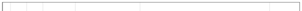

C18pos: m/z_t_R, 177.06220_0.76

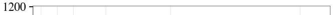

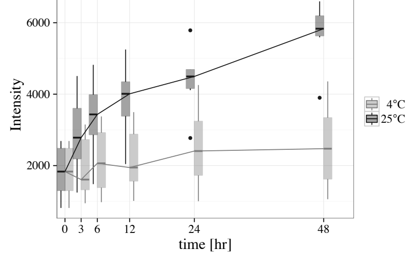

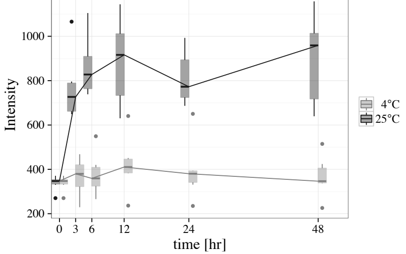

C18pos: m/z_t_R, 426.35909_9.47

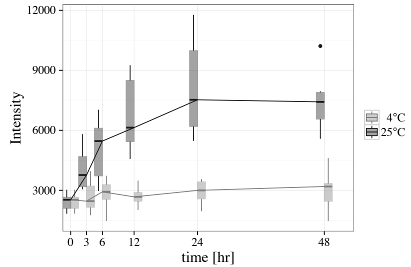

C18pos: m/z_t_R, 991.67186_10.12

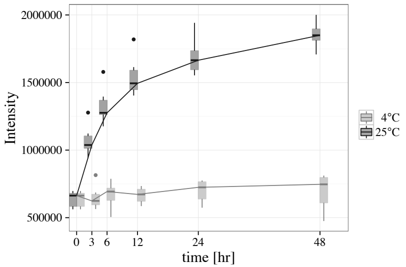

C18pos: m/z_t_R, 496.34144_9.96

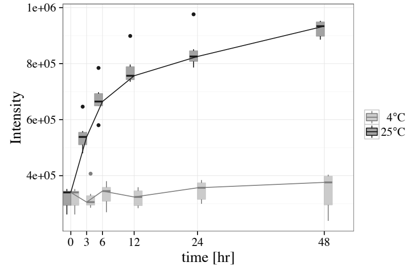

C18pos: m/z_t_R, 548.36924_10.37

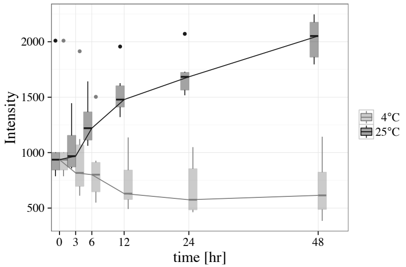

C18pos: m/z_t_R, 540.30693_9.42

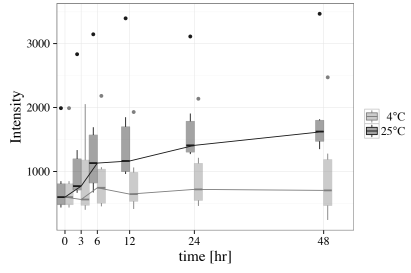

C18pos: m/z_t_R, 542.32522_9.44

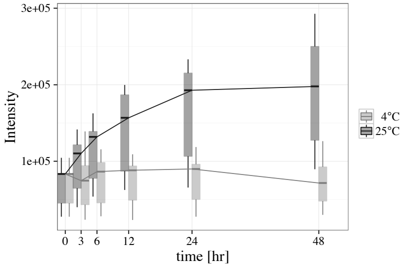

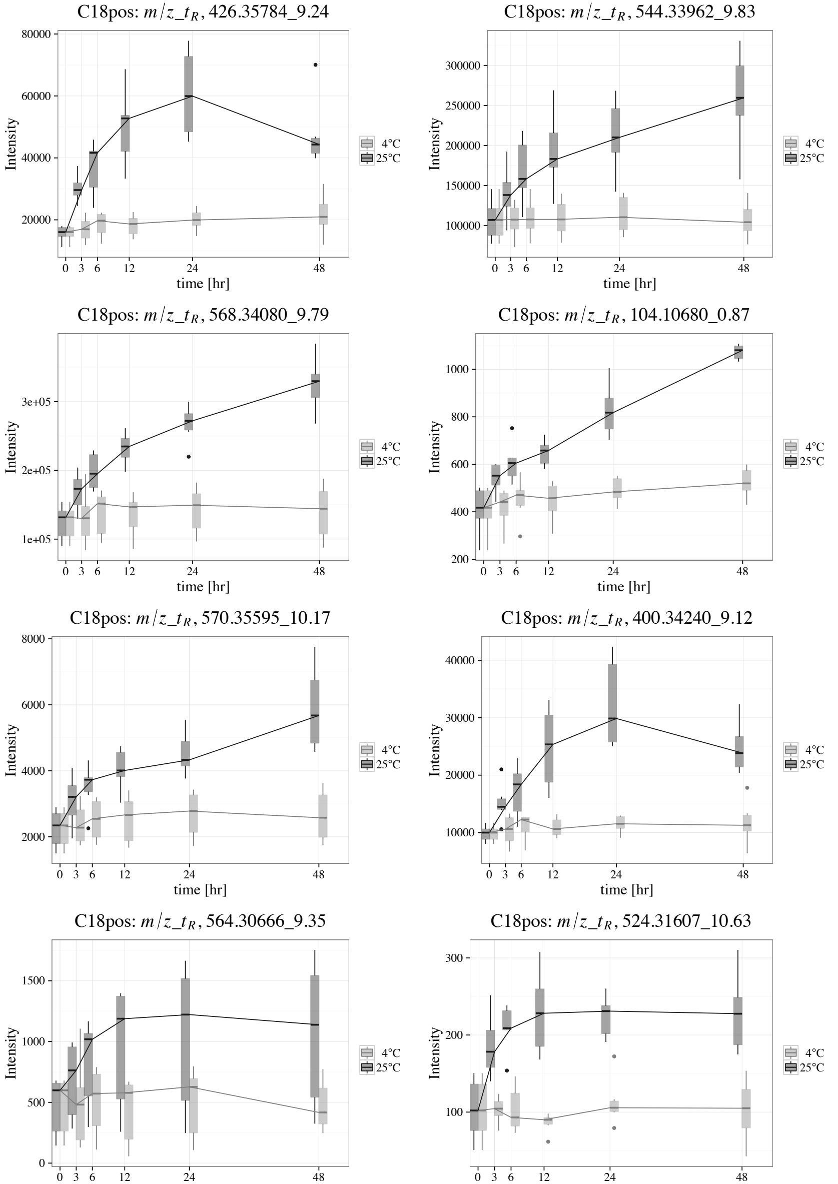
C18pos: m/z_t_R, 426.35784_9.24
C18pos: m/z_t_R, 544.33962_9.83
C18pos: m/z_t_R, 568.34080_9.79
C18pos: m/z_t_R, 104.10680_0.87
C18pos: m/z_t_R, 570.35595_10.17
C18pos: m/z_t_R, 400.34240_9.12
C18pos: m/z_t_R, 564.30666_9.35
C18pos: m/z_t_R, 524.31607_10.63
Intensity
time [hr]
4°C
25°C

time [hr]

time [hr]

C18pos: m/z_t_R, 568.34041_9.69

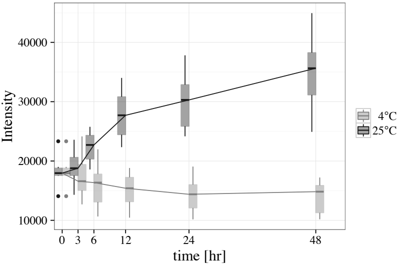

C18pos: m/z_t_R, 227.12755_8.46

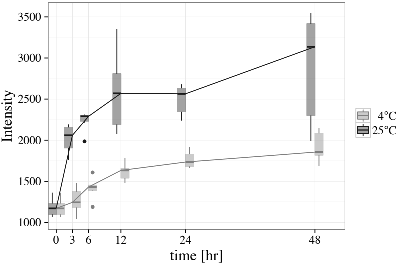

C18pos: m/z_t_R, 522.35717_10.33

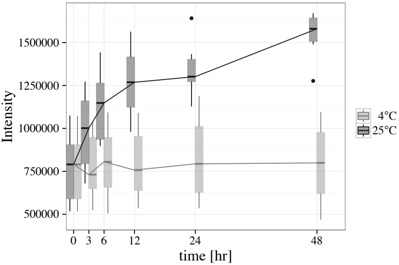

C18pos: m/z_t_R, 485.26560_10.33

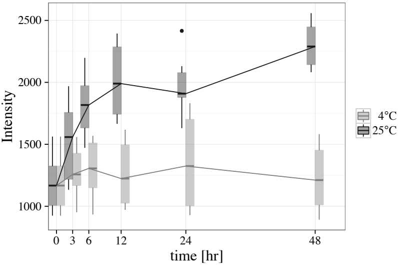

C18pos: m/z_t_R, 130.08689_0.70

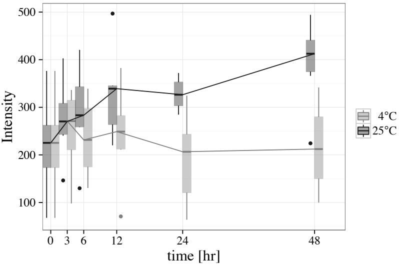

C18pos: m/z_t_R, 205.19456_10.48

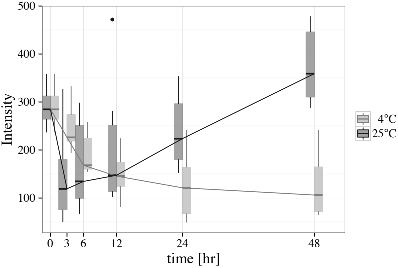

C18pos: m/z_t_R, 429.11978_9.39

C18pos: m/z_t_R, 395.03816_0.77

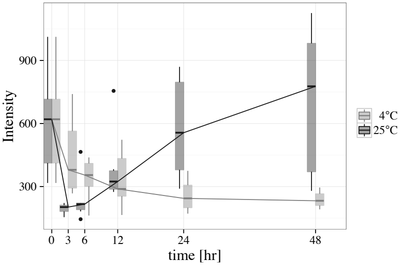

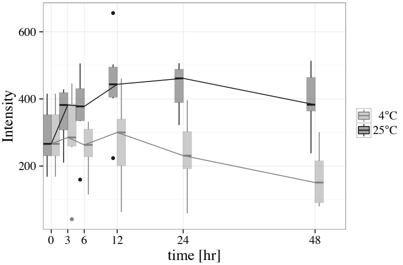

C18pos: m/z_t_R, 257.07582_0.85

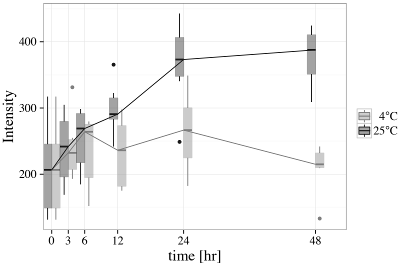

C18pos: m/z_t_R, 566.32252_9.72

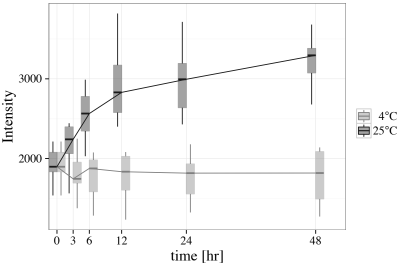

C18pos: m/z_t_R, 570.35599_9.97

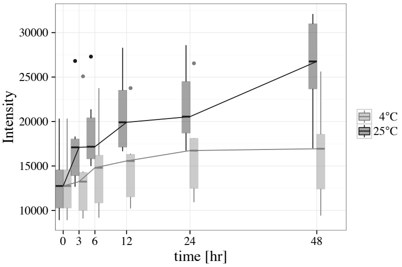

C18pos: m/z_t_R, 508.33892_10.17

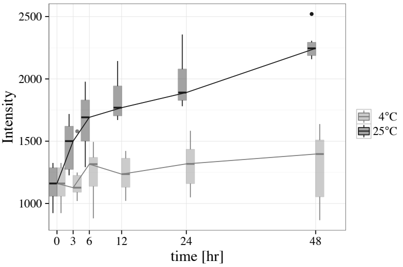

C18pos: m/z_t_R, 240.10128_9.96

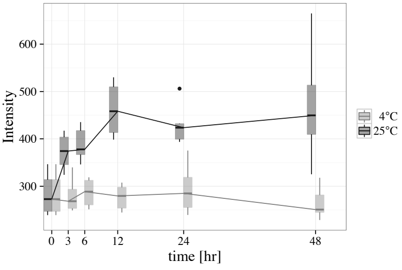

C18pos: m/z_t_R, 480.31070_10.30

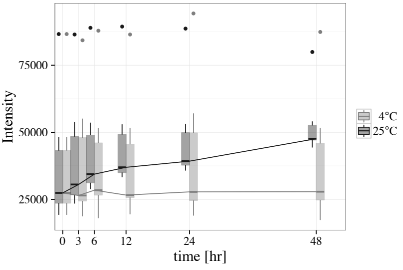

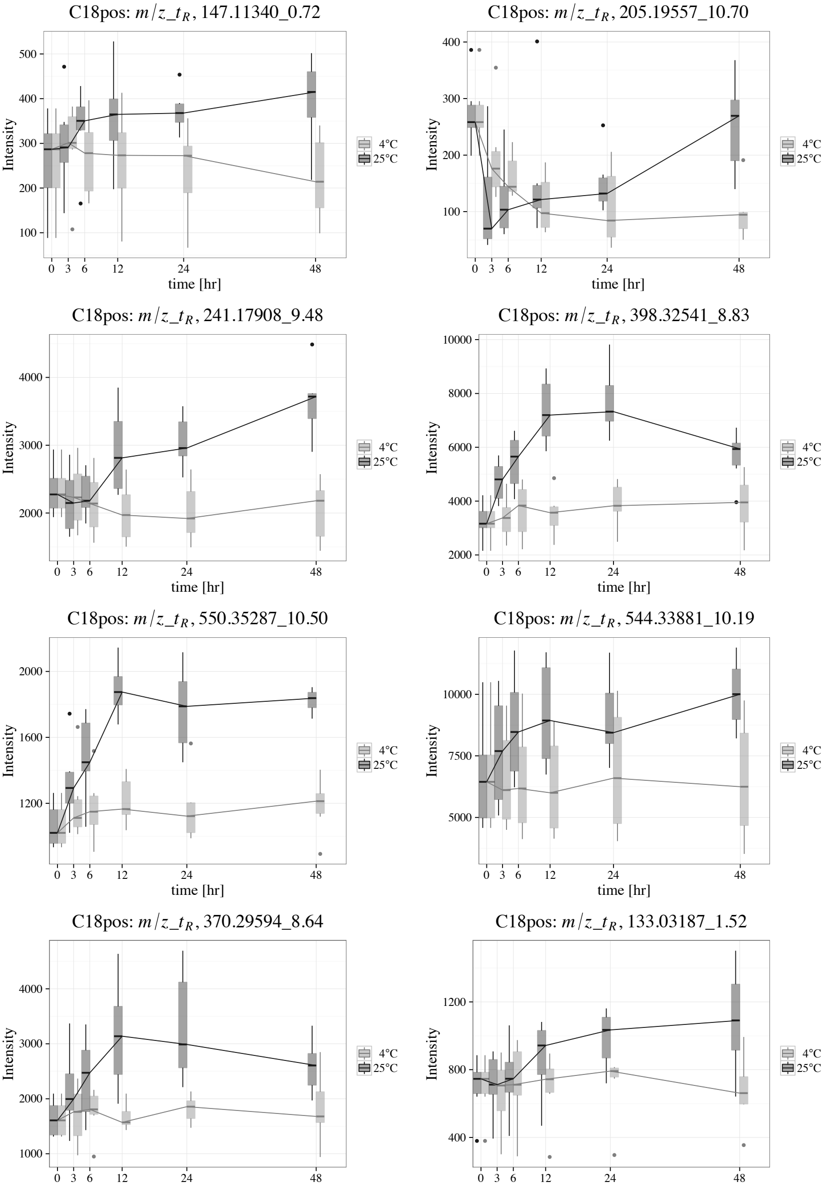
C18pos: m/z_t_R, 147.11340_0.72
C18pos: m/z_t_R, 205.19557_10.70
C18pos: m/z_t_R, 241.17908_9.48
C18pos: m/z_t_R, 398.32541_8.83
C18pos: m/z_t_R, 550.35287_10.50
C18pos: m/z_t_R, 544.33881_10.19
C18pos: m/z_t_R, 370.29594_8.64
C18pos: m/z_t_R, 133.03187_1.52
Intensity
time [hr]
0
3
6
12
24
48
4°C
25°C

time [hr]

time [hr]

C18pos: m/z_t_R, 227.17539_6.25

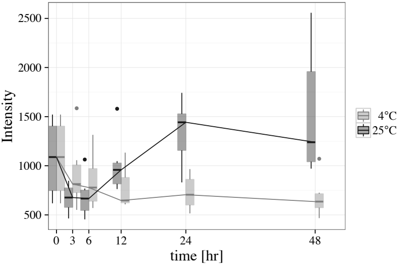

C18pos: m/z_t_R, 297.14726_9.96

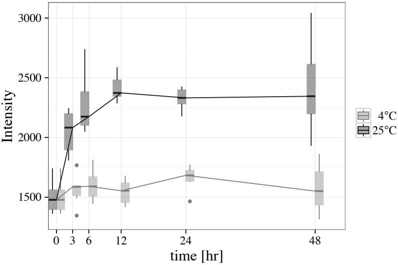

C18pos: m/z_t_R, 546.35621_10.10

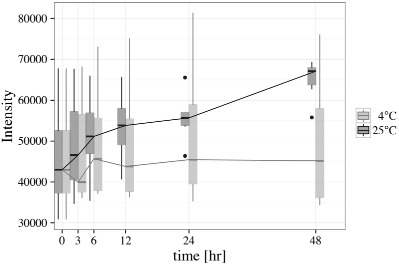

C18pos: m/z_t_R, 288.29065_8.02

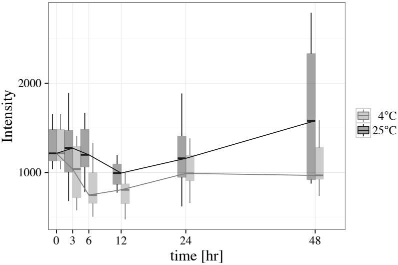

C18pos: m/z_t_R, 119.04934_2.11

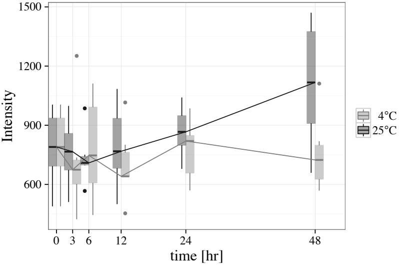

C18pos: m/z_t_R, 132.10245_2.18

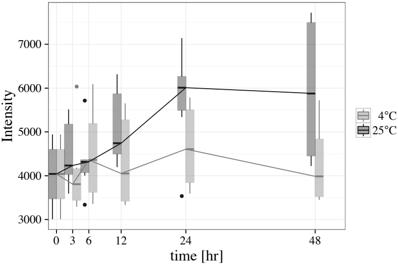

C18pos: m/z_t_R, 280.15347_5.58

C18pos: m/z_t_R, 191.07826_0.77

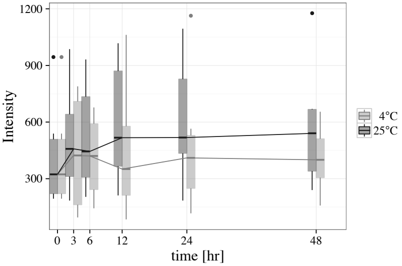

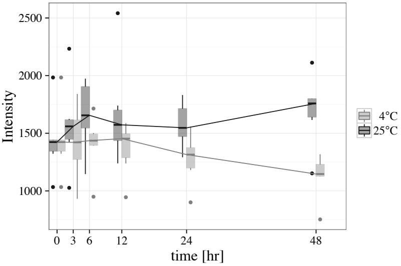

C18pos: m/z_t_R, 125.02138_0.82

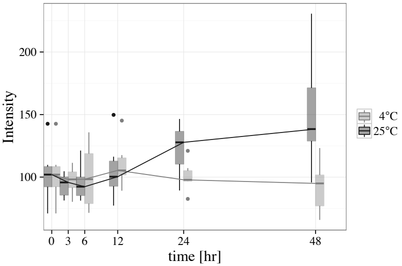

C18pos: m/z_t_R, 288.29098_8.11

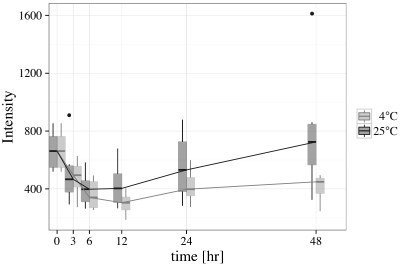

C18pos: m/z_t_R, 295.19082_9.42

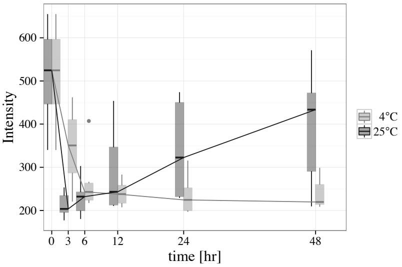

C18pos: m/z_t_R, 935.80615_0.80

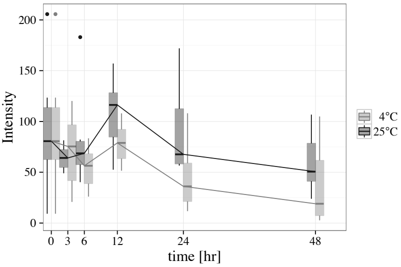

C18pos: m/z_t_R, 459.24999_10.12

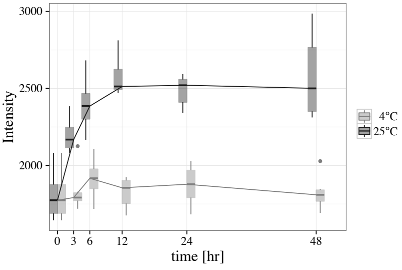

C18pos: m/z_t_R, 123.04459_2.11

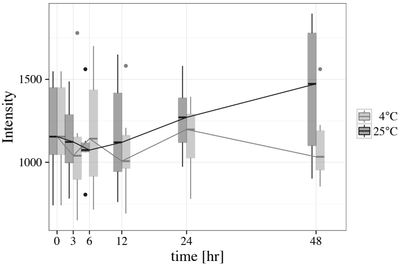

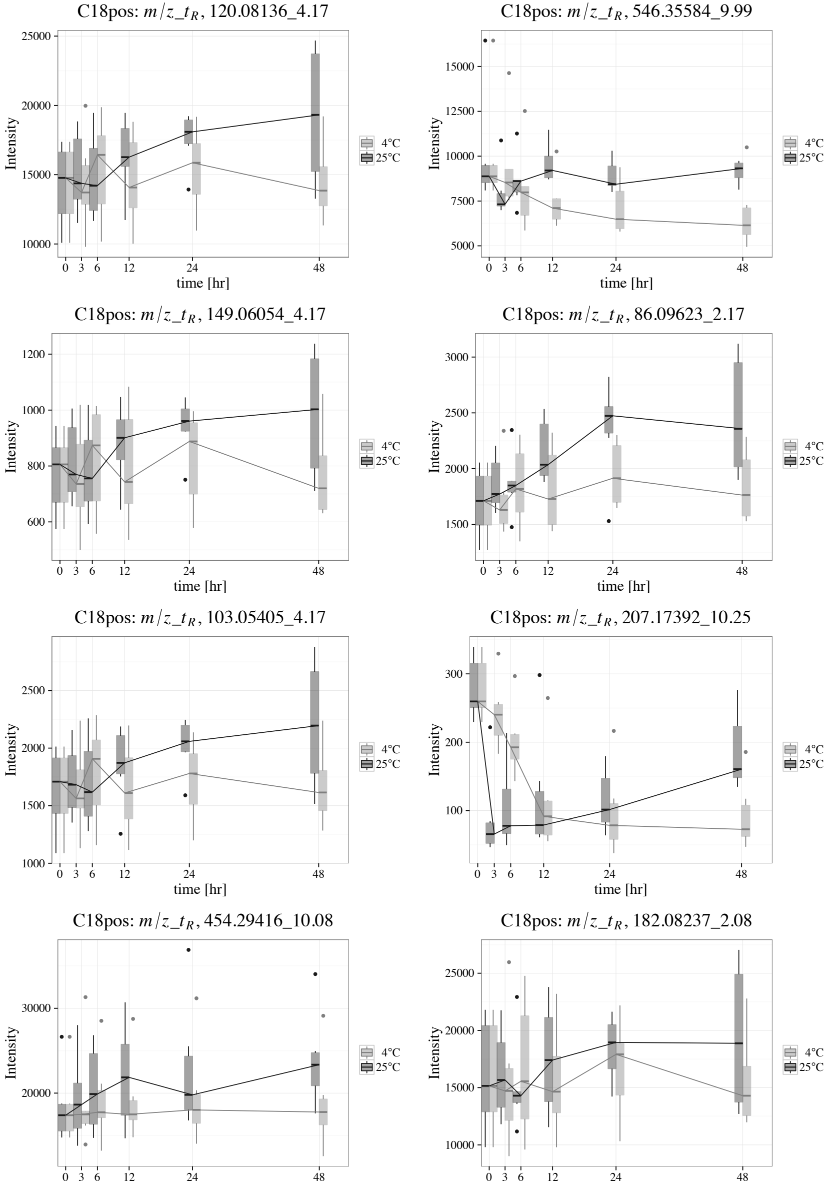
C18pos: m/z_t_R, 120.08136_4.17
C18pos: m/z_t_R, 546.35584_9.99
C18pos: m/z_t_R, 149.06054_4.17
C18pos: m/z_t_R, 86.09623_2.17
C18pos: m/z_t_R, 103.05405_4.17
C18pos: m/z_t_R, 207.17392_10.25
C18pos: m/z_t_R, 454.29416_10.08
C18pos: m/z_t_R, 182.08237_2.08
Intensity
time [hr]
0
3
6
12
24
48
4°C
25°C

time [hr]

time [hr]

C18pos: m/z_t_R, 961.36190_0.79

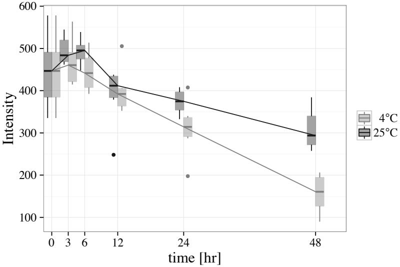

C18pos: m/z_t_R, 635.37845_0.77

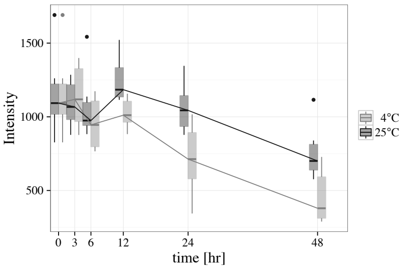

C18pos: m/z_t_R, 138.05563_0.94

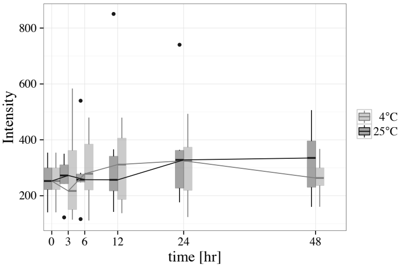

C18pos: m/z_t_R, 577.13458_10.28

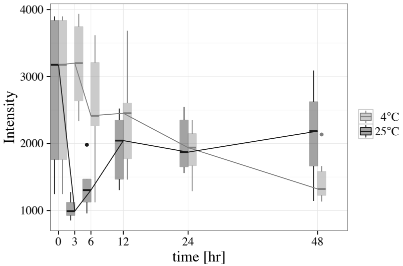

C18pos: m/z_t_R, 1043.30114_0.78

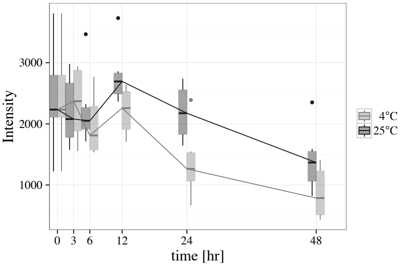

C18pos: m/z_t_R, 132.10225_2.00

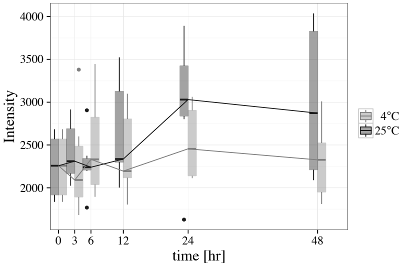

C18pos: m/z_t_R, 185.11657_6.90

C18pos: m/z_t_R, 1187.26049_0.76

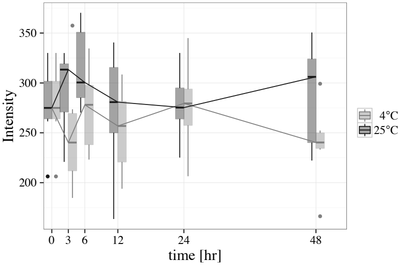

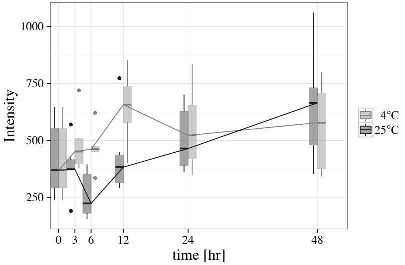

C18pos: m/z_t_R, 359.23498_6.58

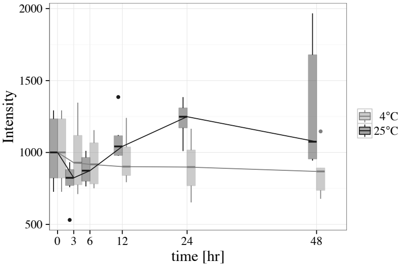

C18pos: m/z_t_R, 86.09637_2.00

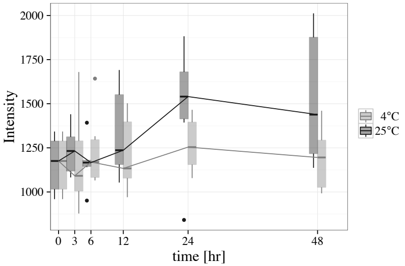

C18pos: m/z_t_R, 339.16068_10.64

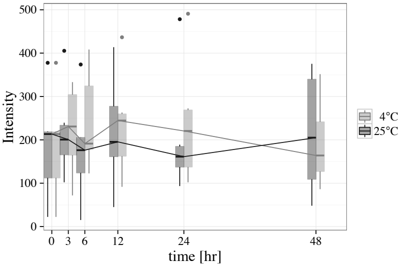

C18pos: m/z_t_R, 197.09701_9.87

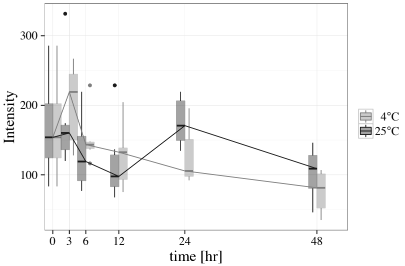

C18pos: m/z_t_R, 114.09166_5.39

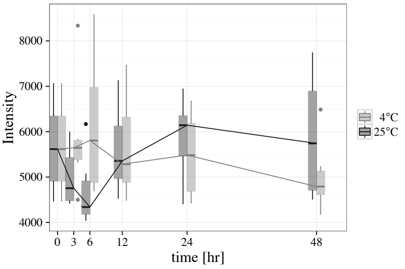

C18pos: m/z_t_R, 489.27702_8.88

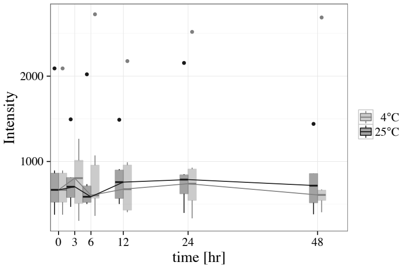

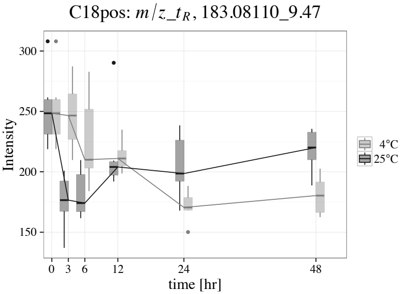
C18pos: m/z_t_R, 183.08110_9.47
Intensity
300
250
200
150
0 3 6 12 24 48
time [hr]
4°C
25°C

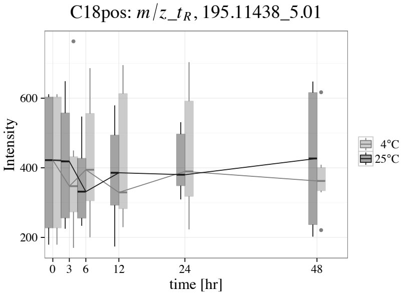
C18pos: m/z_t_R, 195.11438_5.01
Intensity
600
400
200
0 3 6 12 24 48
time [hr]
4°C
25°C

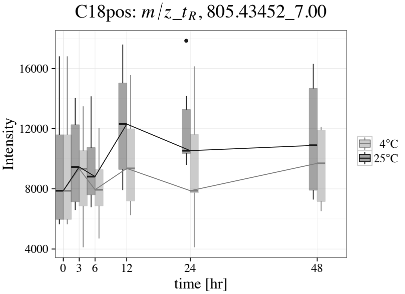
C18pos: m/z_t_R, 805.43452_7.00
Intensity
16000
12000
8000
4000
0 3 6 12 24 48
time [hr]
4°C
25°C

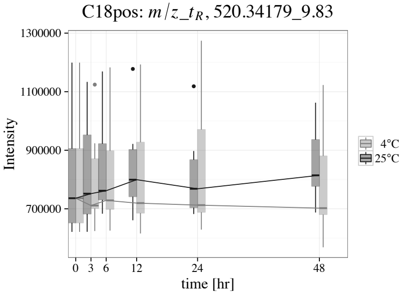
C18pos: m/z_t_R, 520.34179_9.83
Intensity
1300000
1100000
900000
700000
0 3 6 12 24 48
time [hr]
4°C
25°C

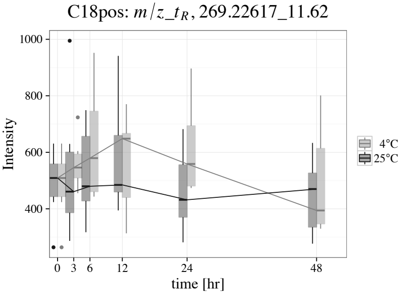
C18pos: m/z_t_R, 269.22617_11.62
Intensity
1000
800
600
400
0 3 6 12 24 48
time [hr]
4°C
25°C

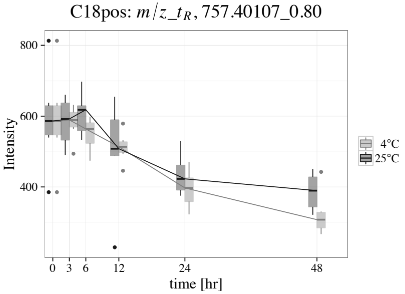
C18pos: m/z_t_R, 757.40107_0.80
Intensity
800
600
400
0 3 6 12 24 48
time [hr]
4°C
25°C

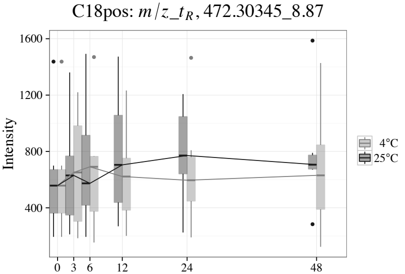
C18pos: m/z_t_R, 472.30345_8.87
Intensity
1600
1200
800
400
0 3 6 12 24 48
4°C
25°C

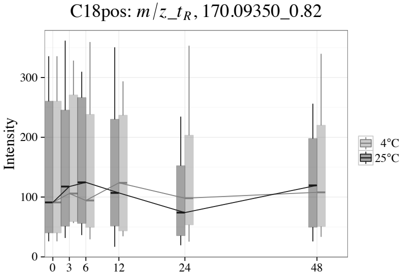
C18pos: m/z_t_R, 170.09350_0.82
Intensity
300
200
100
0
0 3 6 12 24 48
4°C
25°C

time [hr]

C18pos: m/z_t_R, 172.16976_8.99

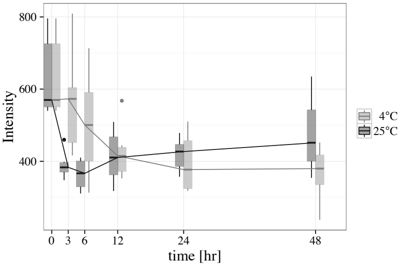

C18pos: m/z_t_R, 634.87637_0.77

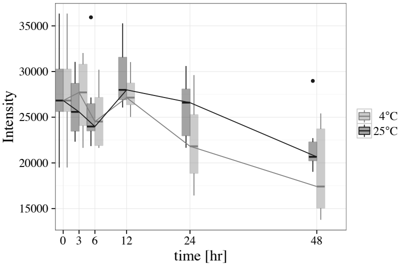

C18pos: m/z_t_R, 603.96435_7.03

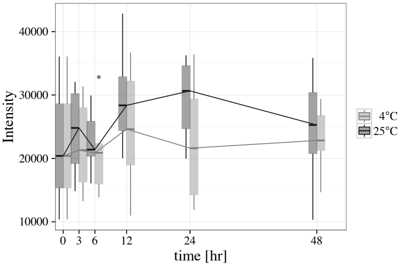

C18pos: m/z_t_R, 457.96155_0.80

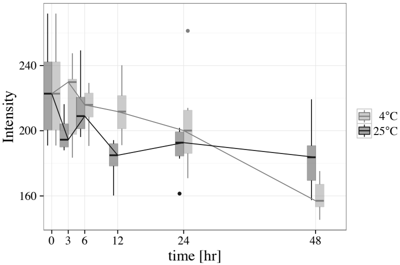

C18pos: m/z_t_R, 919.49274_7.00

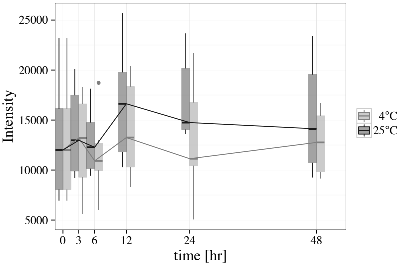

C18pos: m/z_t_R, 204.12470_1.50

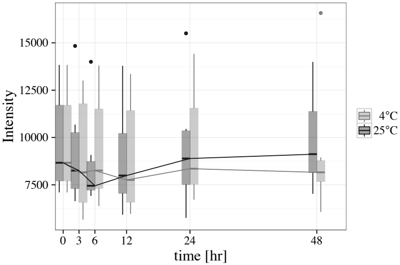

C18pos: m/z_t_R, 829.44517_7.01

C18pos: m/z_t_R, 791.39630_0.80

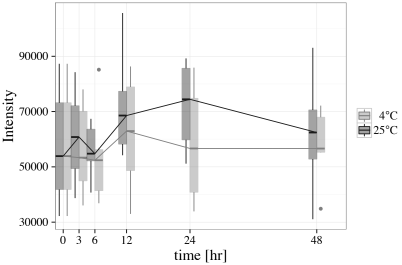

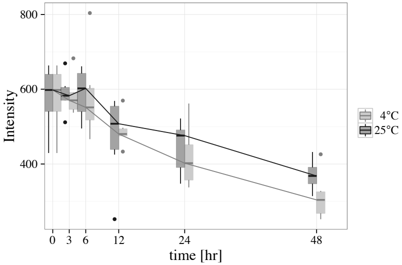

C18pos: m/z_t_R, 224.12890_1.34

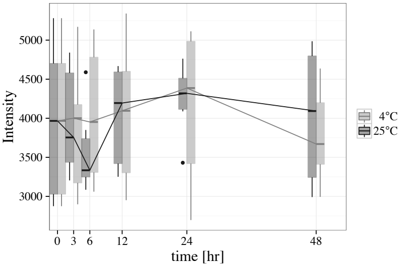

C18pos: m/z_t_R, 245.07669_1.52

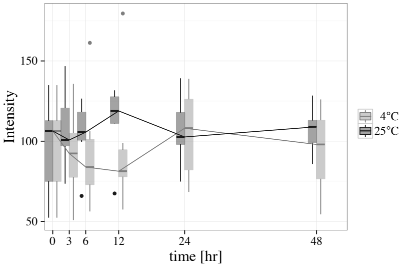

C18pos: m/z_t_R, 249.13560_9.15

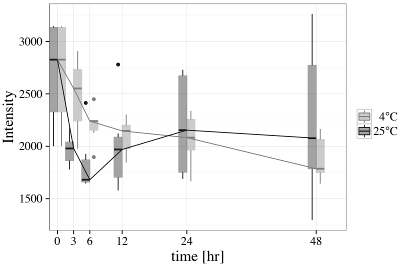

C18pos: m/z_t_R, 160.13374_1.00

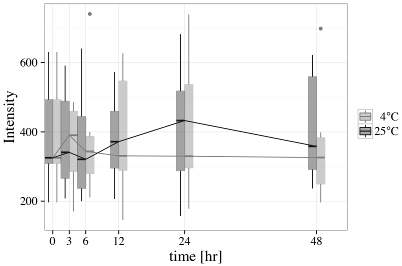

C18pos: m/z_t_R, 297.14754_10.14

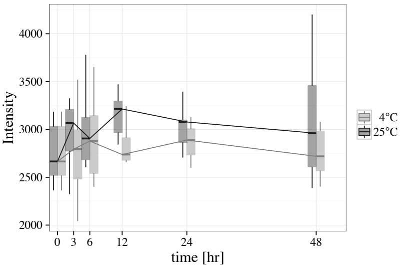

C18pos: m/z_t_R, 919.77796_7.01

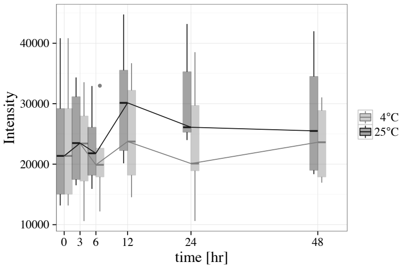

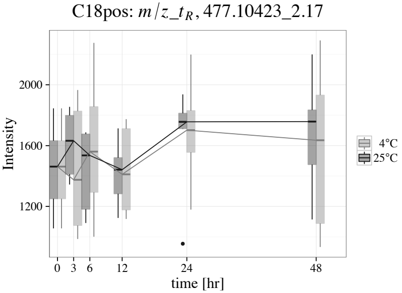
C18pos: m/z_t_R, 477.10423_2.17
Intensity
2000
1600
1200
0 3 6 12 24 48
time [hr]
4°C
25°C

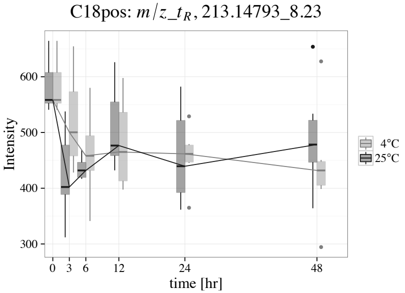
C18pos: m/z_t_R, 213.14793_8.23
Intensity
600
500
400
300
0 3 6 12 24 48
time [hr]
4°C
25°C

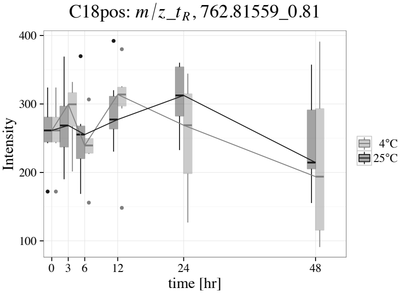
C18pos: m/z_t_R, 762.81559_0.81
Intensity
400
300
200
100
0 3 6 12 24 48
time [hr]
4°C
25°C

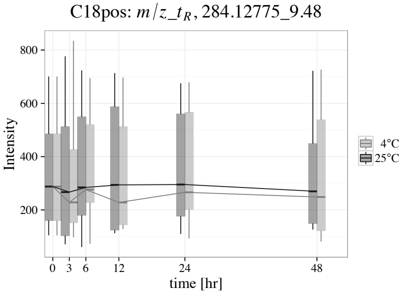
C18pos: m/z_t_R, 284.12775_9.48
Intensity
800
600
400
200
0 3 6 12 24 48
time [hr]
4°C
25°C

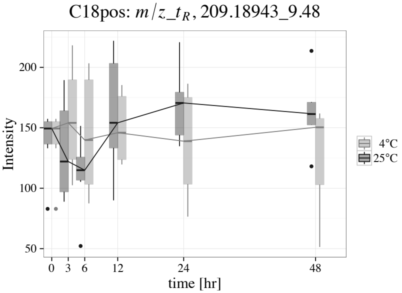
C18pos: m/z_t_R, 209.18943_9.48
Intensity
200
150
100
50
0 3 6 12 24 48
time [hr]
4°C
25°C

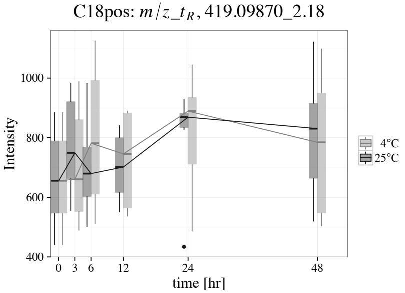
C18pos: m/z_t_R, 419.09870_2.18
Intensity
1000
800
600
400
0 3 6 12 24 48
time [hr]
4°C
25°C

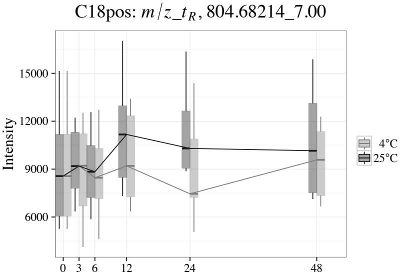
C18pos: m/z_t_R, 804.68214_7.00
Intensity
15000
12000
9000
6000
0 3 6 12 24 48
4°C
25°C

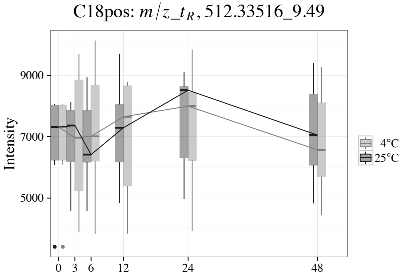
C18pos: m/z_t_R, 512.33516_9.49
Intensity
9000
7000
5000
0 3 6 12 24 48
4°C
25°C

time [hr]

time [hr]

C18pos: m/z_t_R, 723.52796_10.64

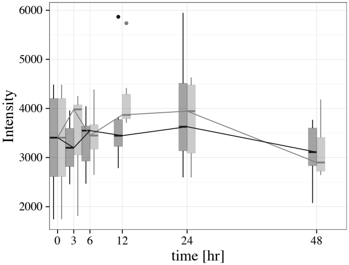

C18pos: m/z_t_R, 204.12424_1.01

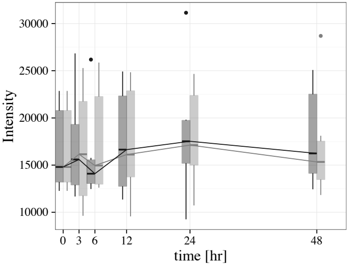

C18pos: m/z_t_R, 245.08007_9.07

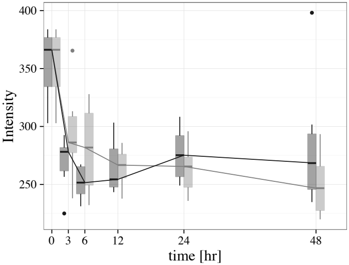

C18pos: m/z_t_R, 130.06584_4.84

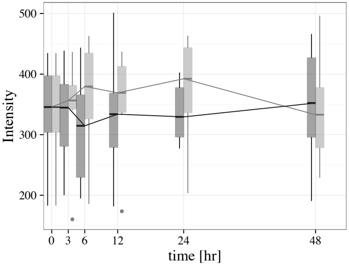

C18pos: m/z_t_R, 348.19275_5.51

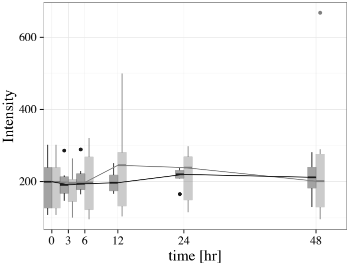

C18pos: m/z_t_R, 621.42620_0.80

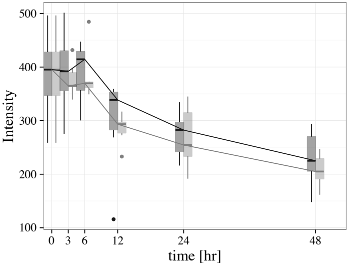

C18pos: m/z_t_R, 197.12945_5.60

C18pos: m/z_t_R, 102.05457_1.18

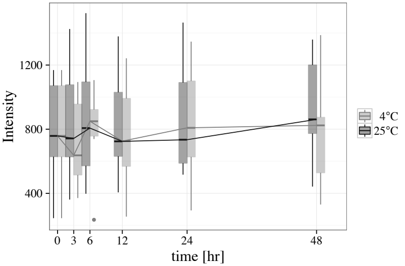

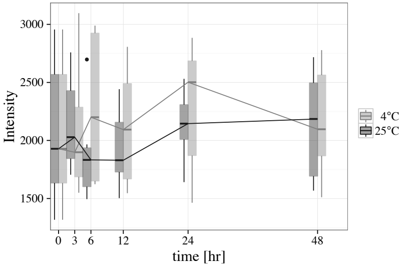

C18pos: m/z_t_R, 195.10349_8.15

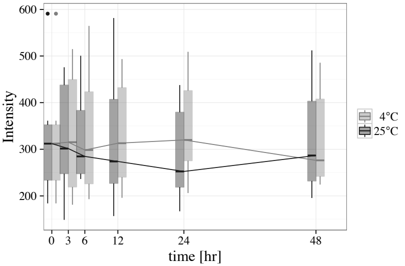

C18pos: m/z_t_R, 172.16956_9.39

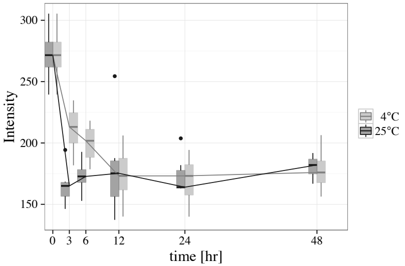

C18pos: m/z_t_R, 290.16023_9.15

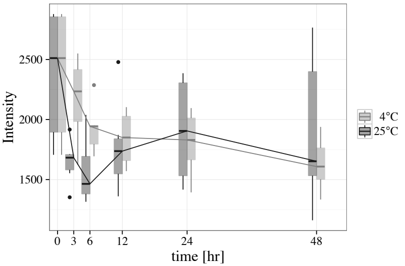

C18pos: m/z_t_R, 463.08855_1.01

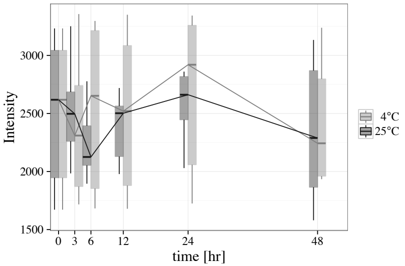

C18pos: m/z_t_R, 326.21612_10.59

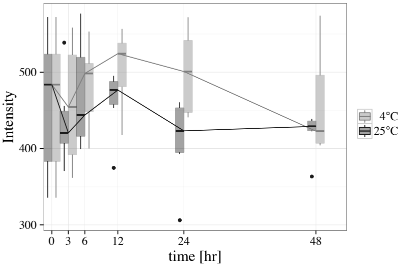

C18pos: m/z_t_R, 177.05519_9.09

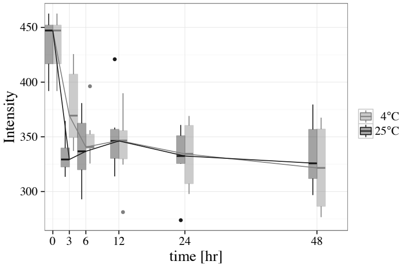

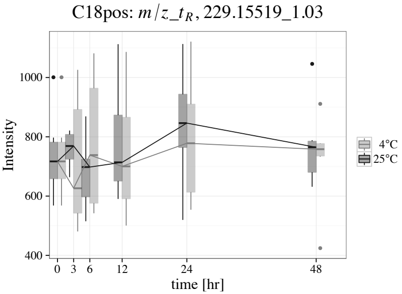
C18pos: m/z_t_R, 229.15519_1.03
Intensity
time [hr]
4°C
25°C

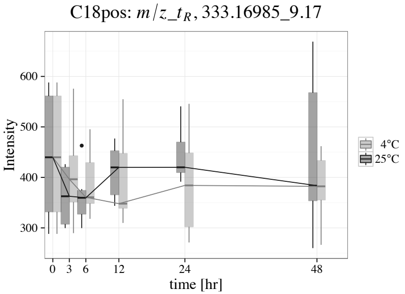
C18pos: m/z_t_R, 333.16985_9.17
Intensity
time [hr]
4°C
25°C

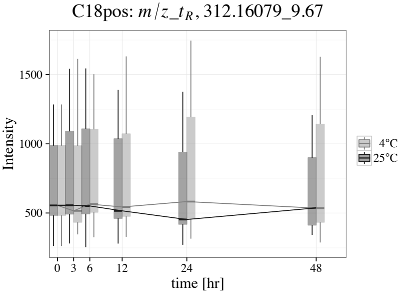
C18pos: m/z_t_R, 312.16079_9.67
Intensity
time [hr]
4°C
25°C

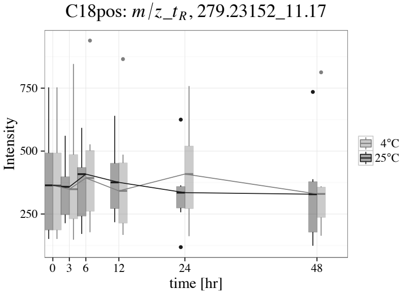
C18pos: m/z_t_R, 279.23152_11.17
Intensity
time [hr]
4°C
25°C

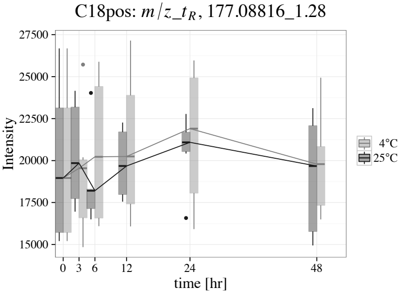
C18pos: m/z_t_R, 177.08816_1.28
Intensity
time [hr]
4°C
25°C

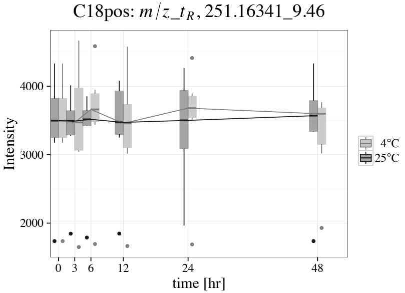
C18pos: m/z_t_R, 251.16341_9.46
Intensity
time [hr]
4°C
25°C

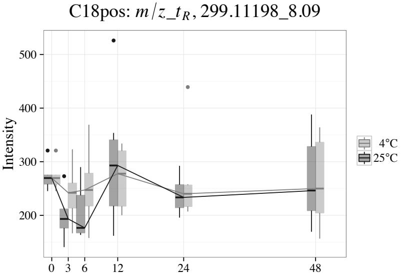
C18pos: m/z_t_R, 299.11198_8.09
Intensity
4°C
25°C

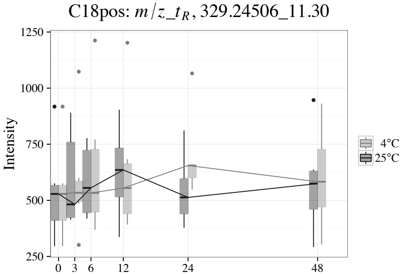
C18pos: m/z_t_R, 329.24506_11.30
Intensity
4°C
25°C

time [hr]

time [hr]

C18pos: m/z_t_R, 414.29967_8.88

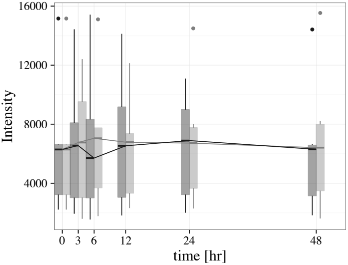

C18pos: m/z_t_R, 174.12292_10.99

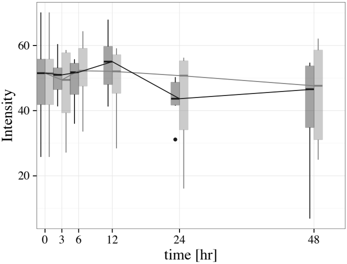

C18pos: m/z_t_R, 385.27504_9.67

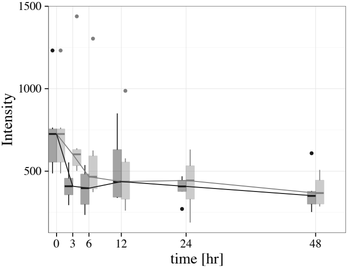

C18pos: m/z_t_R, 324.00019_0.85

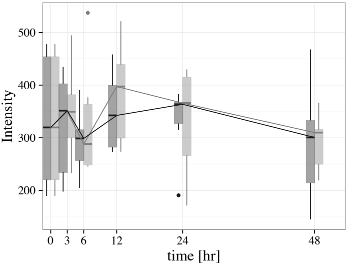

C18pos: m/z_t_R, 195.10370_9.66

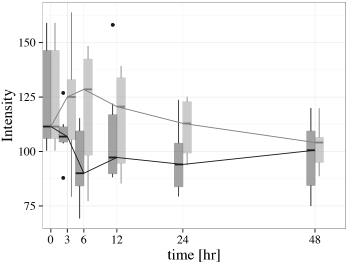

C18pos: m/z_t_R, 432.31024_8.88

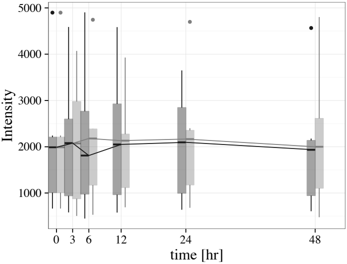

C18pos: m/z_t_R, 190.08760_7.53

C18pos: m/z_t_R, 331.21029_7.13

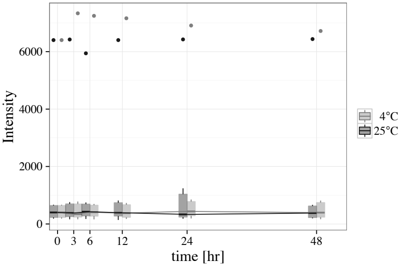

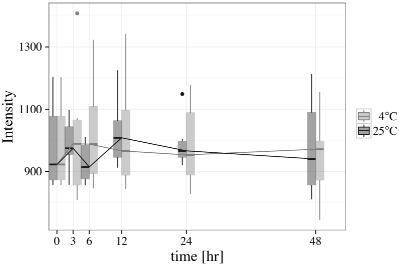

C18pos: m/z_t_R, 263.09112_8.14

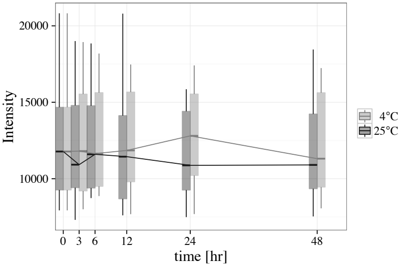

C18pos: m/z_t_R, 312.16076_9.62

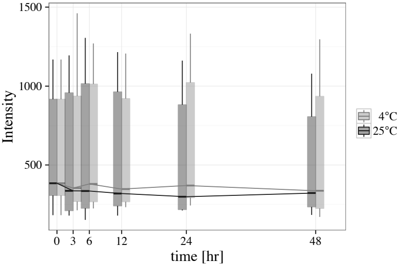

C18pos: m/z_t_R, 285.25796_8.48

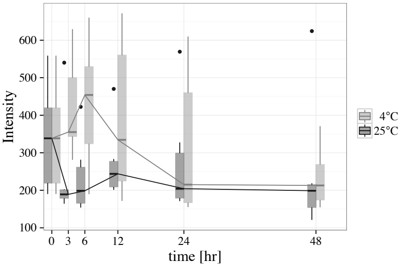

C18pos: m/z_t_R, 279.15957_10.62

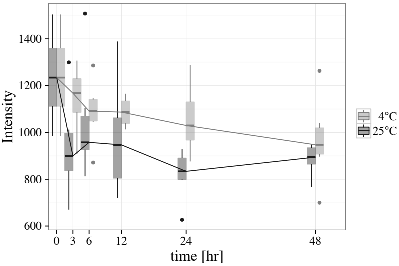

C18pos: m/z_t_R, 211.14519_6.18

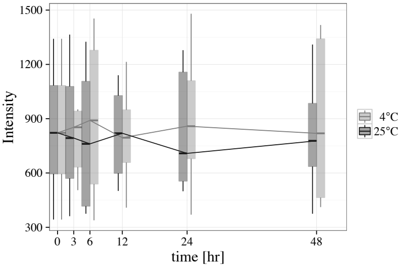

C18pos: m/z_t_R, 302.23323_7.01

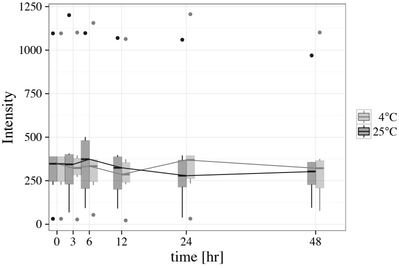

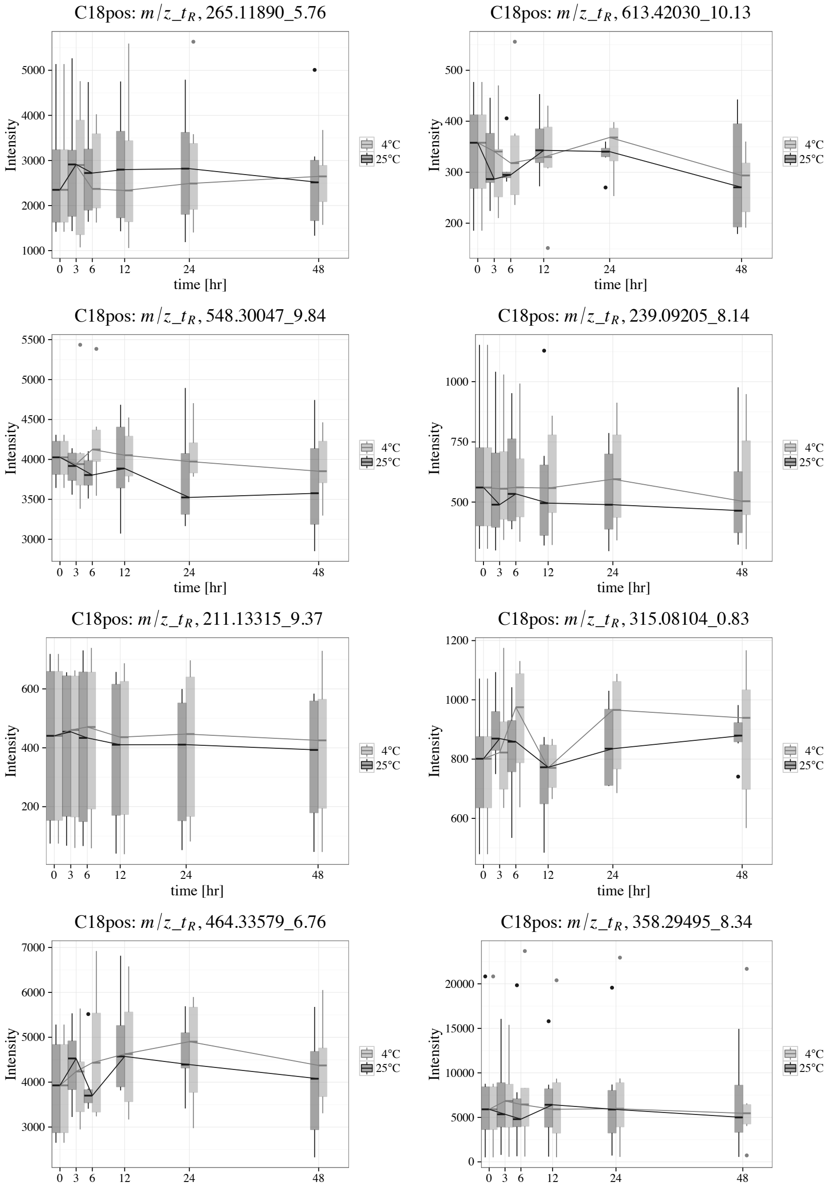

C18pos: m/z_t_R, 265.11890_5.76
C18pos: m/z_t_R, 613.42030_10.13
C18pos: m/z_t_R, 548.30047_9.84
C18pos: m/z_t_R, 239.09205_8.14
C18pos: m/z_t_R, 211.13315_9.37
C18pos: m/z_t_R, 315.08104_0.83
C18pos: m/z_t_R, 464.33579_6.76
C18pos: m/z_t_R, 358.29495_8.34
Intensity
time [hr]
0 3 6 12 24 48
4°C
25°C

time [hr]

C18pos: m/z_t_R, 500.27887_9.42

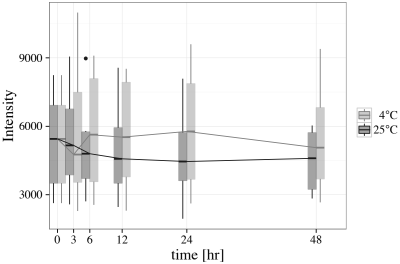

C18pos: m/z_t_R, 309.20556_11.14

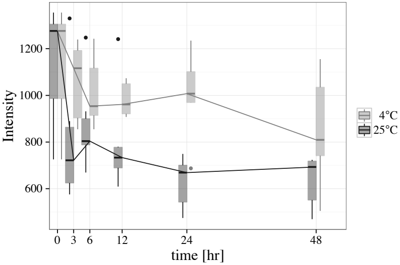

C18pos: m/z_t_R, 328.24869_7.52

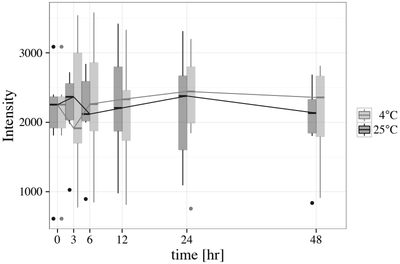

C18pos: m/z_t_R, 195.08975_5.63

time [hr]

C18pos: m/z_t_R, 225.14867_8.84

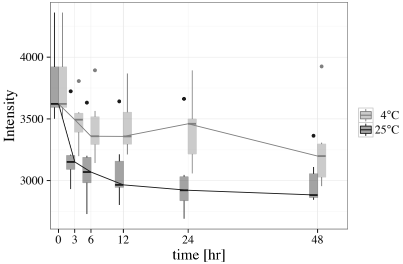

C18pos: m/z_t_R, 286.14495_9.29

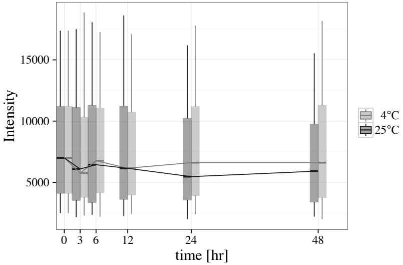

C18pos: m/z_t_R, 201.05510_9.17

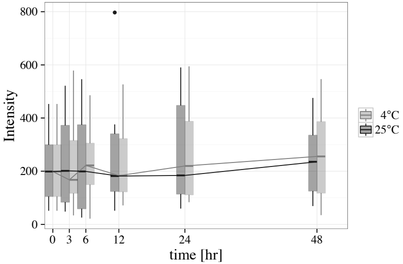

C18pos: m/z_t_R, 277.21674_10.01

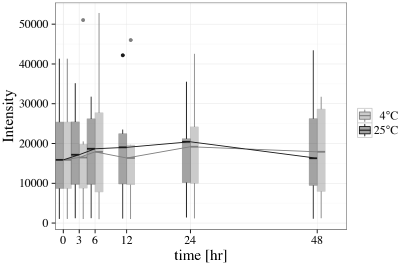

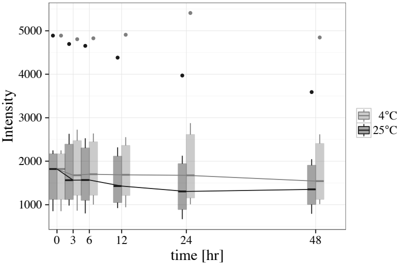

C18pos: m/z_t_R, 260.08121_8.14

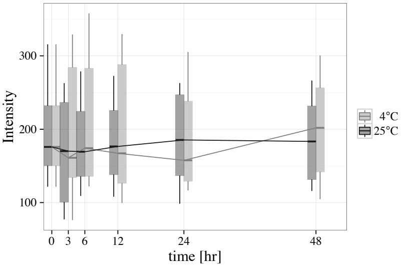

C18pos: m/z_t_R, 1119.27466_0.77

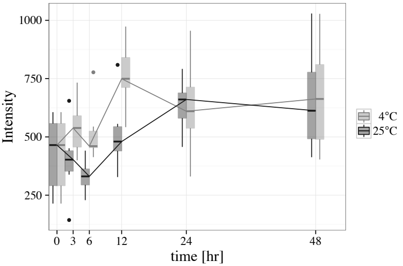

C18pos: m/z_t_R, 286.14495_9.17

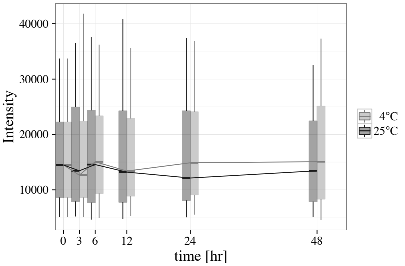

C18pos: m/z_t_R, 287.10122_5.76

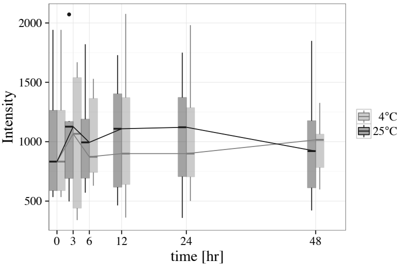

C18pos: m/z_t_R, 524.30046_10.11

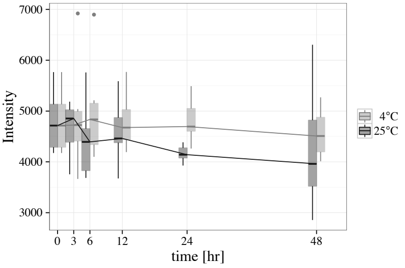

C18pos: m/z_t_R, 130.06582_7.05

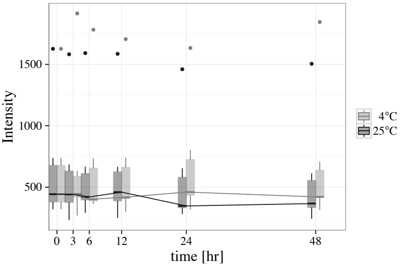

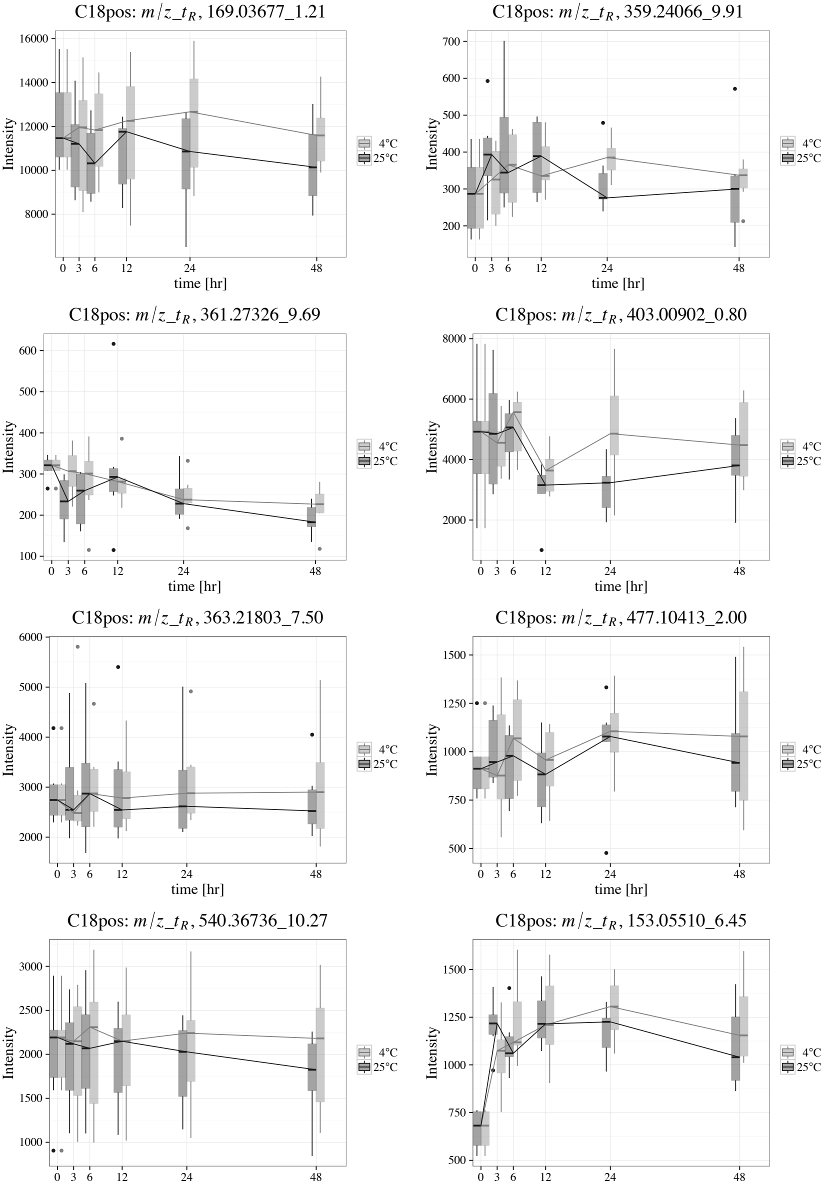

C18pos: m/z_t_R, 169.03677_1.21
C18pos: m/z_t_R, 359.24066_9.91
C18pos: m/z_t_R, 361.27326_9.69
C18pos: m/z_t_R, 403.00902_0.80
C18pos: m/z_t_R, 363.21803_7.50
C18pos: m/z_t_R, 477.10413_2.00
C18pos: m/z_t_R, 540.36736_10.27
C18pos: m/z_t_R, 153.05510_6.45
Intensity
time [hr]
0
3
6
12
24
48
4°C
25°C

time [hr]

time [hr]

C18pos: m/z_t_R, 118.08674_0.89

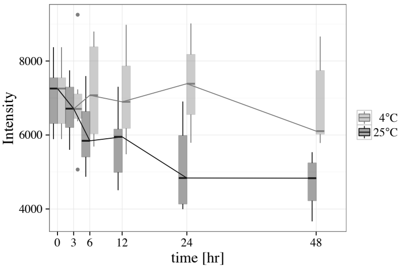

C18pos: m/z_t_R, 777.69471_7.75

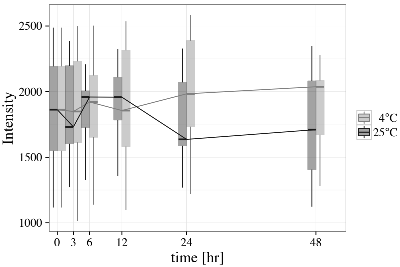

C18pos: m/z_t_R, 369.23373_8.38

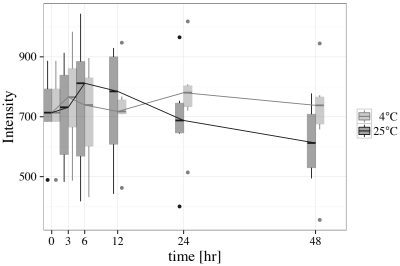

C18pos: m/z_t_R, 288.00413_2.27

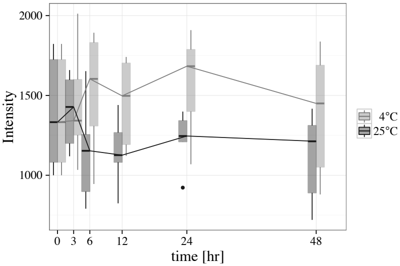

C18pos: m/z_t_R, 277.18035_10.16

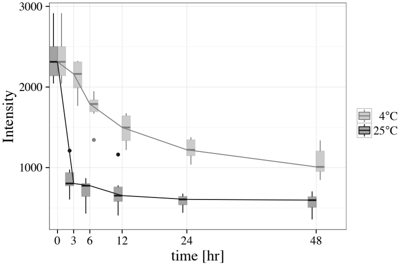

C18pos: m/z_t_R, 217.08357_1.16

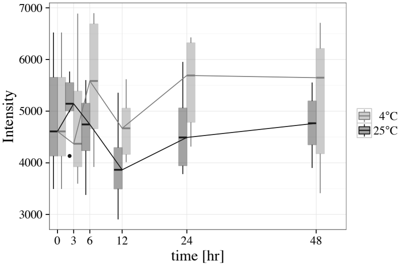

C18pos: m/z_t_R, 677.35896_0.76

C18pos: m/z_t_R, 125.06028_6.45

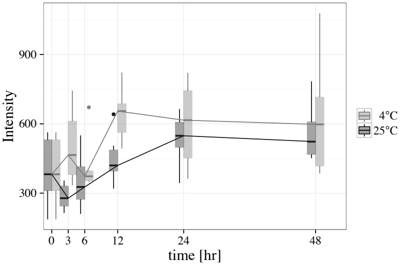

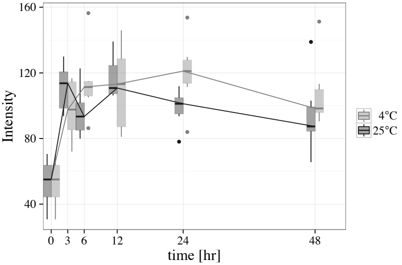

C18pos: m/z_t_R, 975.08469_7.01

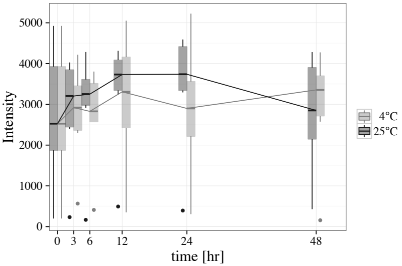

C18pos: m/z_t_R, 472.31993_6.76

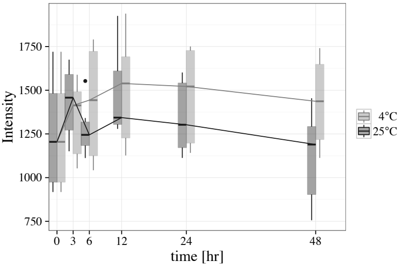

C18pos: m/z_t_R, 262.14513_10.44

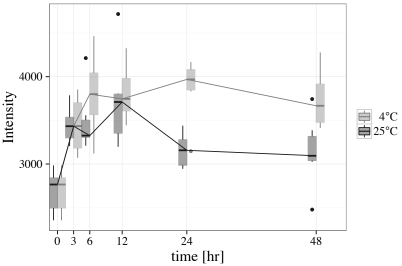

C18pos: m/z_t_R, 357.27918_9.72

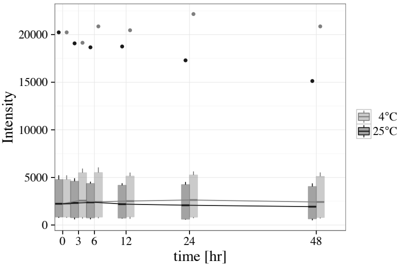

C18pos: m/z_t_R, 512.33352_8.25

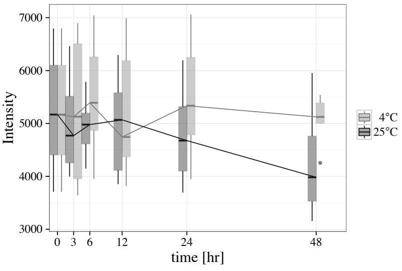

C18pos: m/z_t_R, 220.93548_0.70

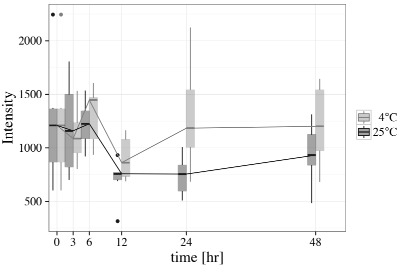

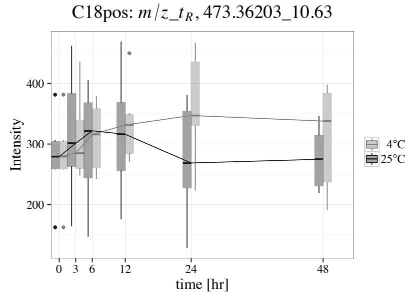
C18pos: m/z_t_R, 473.36203_10.63
Intensity
400
300
200
0 3 6 12 24 48
time [hr]
4°C
25°C

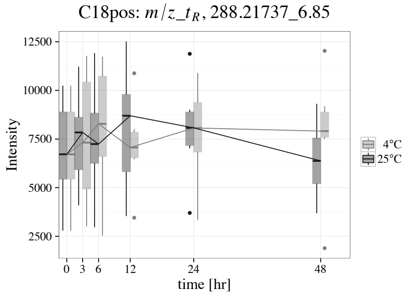
C18pos: m/z_t_R, 288.21737_6.85
Intensity
12500
10000
7500
5000
2500
0 3 6 12 24 48
time [hr]
4°C
25°C

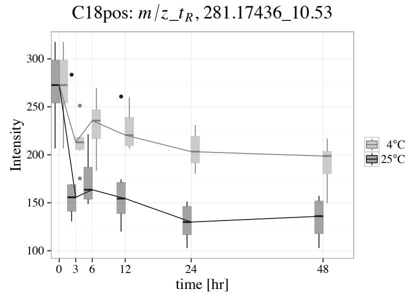
C18pos: m/z_t_R, 281.17436_10.53
Intensity
300
250
200
150
100
0 3 6 12 24 48
time [hr]
4°C
25°C

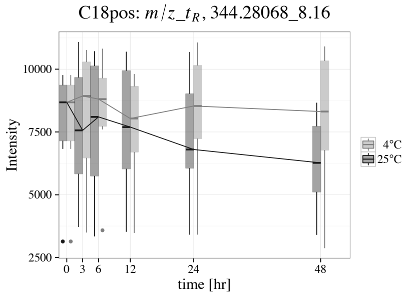
C18pos: m/z_t_R, 344.28068_8.16
Intensity
10000
7500
5000
2500
0 3 6 12 24 48
time [hr]
4°C
25°C

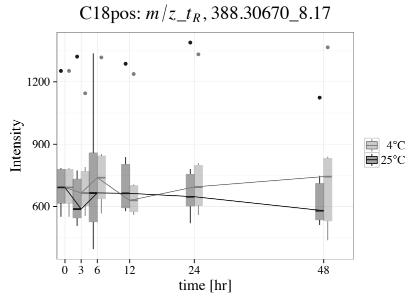
C18pos: m/z_t_R, 388.30670_8.17
Intensity
1200
900
600
0 3 6 12 24 48
time [hr]
4°C
25°C

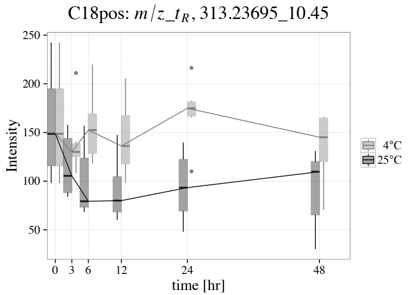
C18pos: m/z_t_R, 313.23695_10.45
Intensity
250
200
150
100
50
0 3 6 12 24 48
time [hr]
4°C
25°C

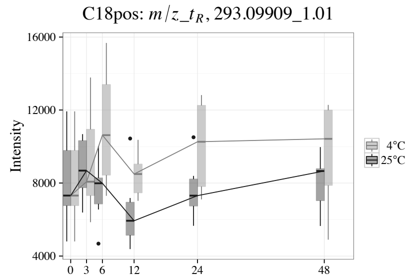
C18pos: m/z_t_R, 293.09909_1.01
Intensity
16000
12000
8000
4000
0 3 6 12 24 48
4°C
25°C

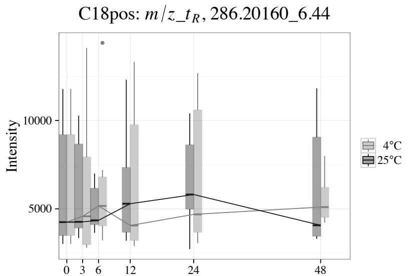
C18pos: m/z_t_R, 286.20160_6.44
Intensity
10000
5000
0 3 6 12 24 48
4°C
25°C

time [hr]

time [hr]

C18pos: m/z_t_R, 195.13760_9.41

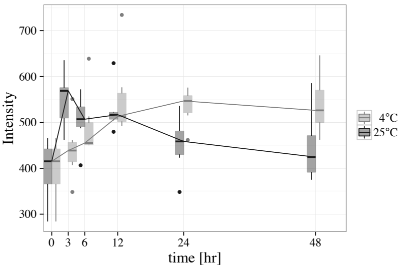

C18pos: m/z_t_R, 181.12255_9.33

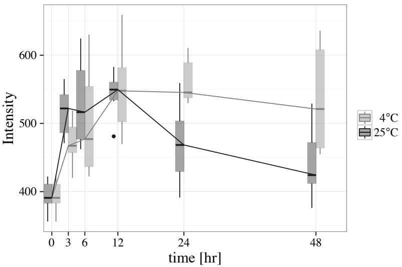

C18pos: m/z_t_R, 211.14414_6.34

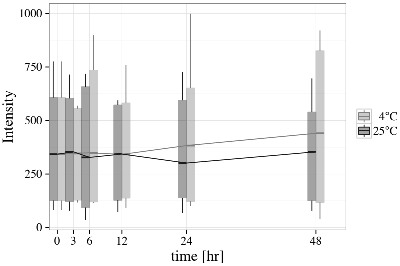

C18pos: m/z_t_R, 368.28065_8.14

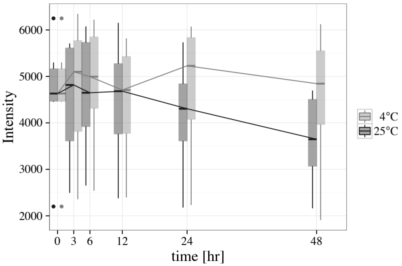

C18pos: m/z_t_R, 357.27934_9.22

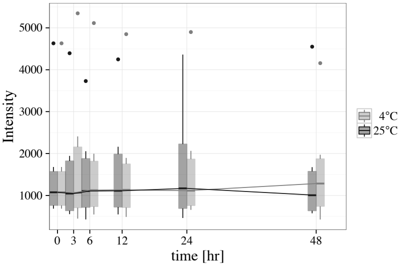

C18pos: m/z_t_R, 185.03073_1.81

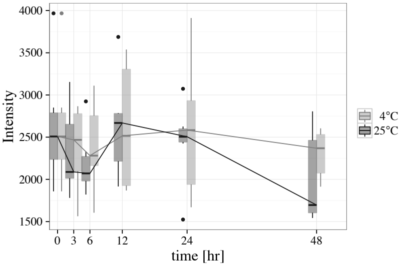

C18pos: m/z_t_R, 286.14502_9.35

C18pos: m/z_t_R, 392.27993_8.16

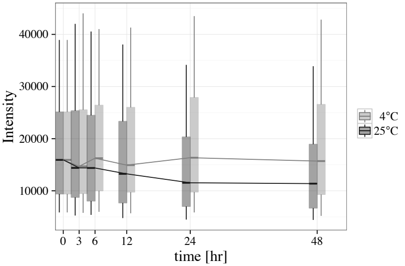

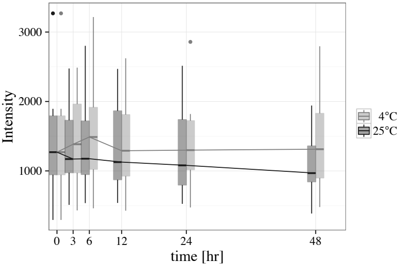

C18pos: m/z_t_R, 502.32788_9.83

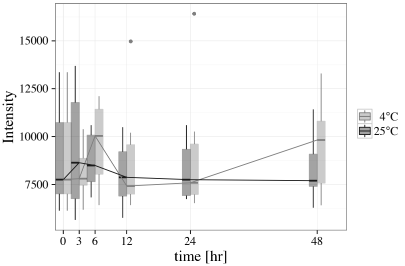

C18pos: m/z_t_R, 526.29446_9.76

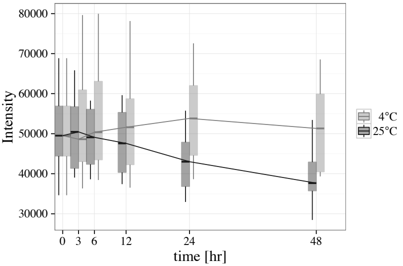

C18pos: m/z_t_R, 189.08840_1.21

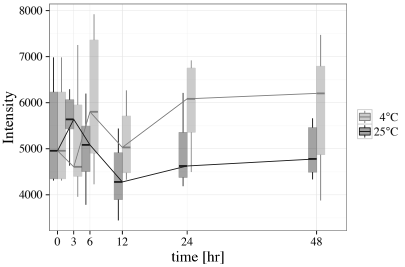

C18pos: m/z_t_R, 100.07596_4.39

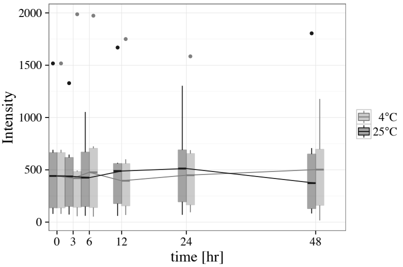

C18pos: m/z_t_R, 191.04208_0.84

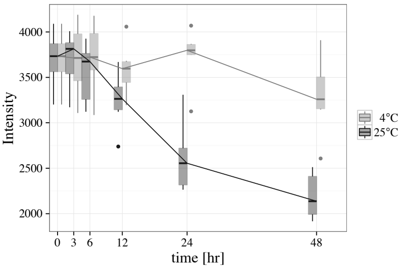

C18pos: m/z_t_R, 337.27491_9.67

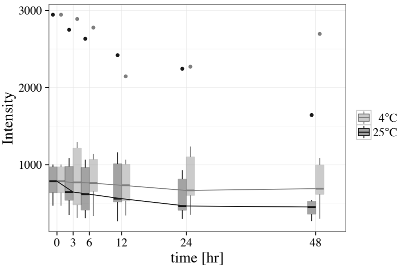

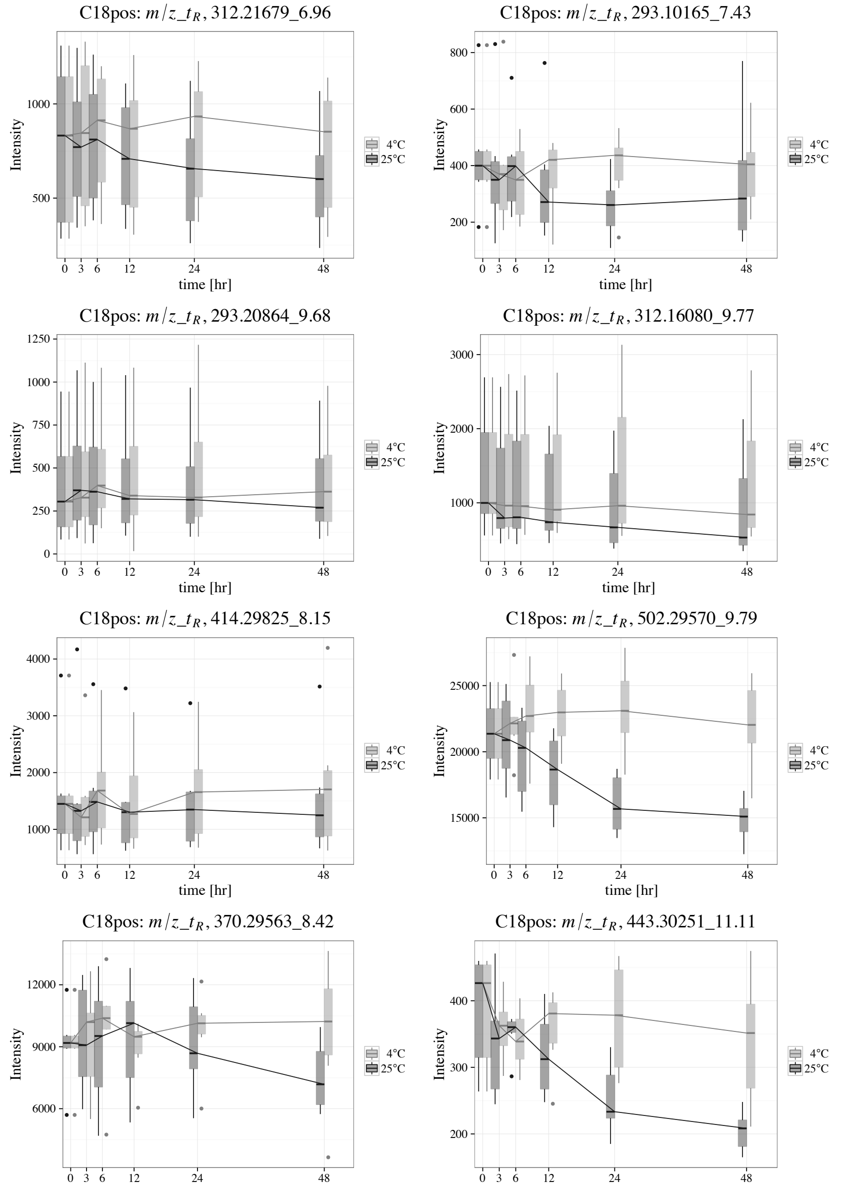

C18pos: m/z_tR, 312.21679_6.96
C18pos: m/z_tR, 293.10165_7.43
C18pos: m/z_tR, 293.20864_9.68
C18pos: m/z_tR, 312.16080_9.77
C18pos: m/z_tR, 414.29825_8.15
C18pos: m/z_tR, 502.29570_9.79
C18pos: m/z_tR, 370.29563_8.42
C18pos: m/z_tR, 443.30251_11.11
Intensity
time [hr]
4°C
25°C

time [hr]

time [hr]

C18pos: m/z_t_R, 339.29082_10.16

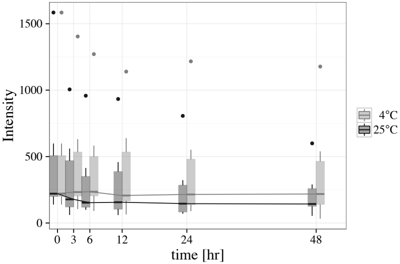

C18pos: m/z_t_R, 314.23318_7.28

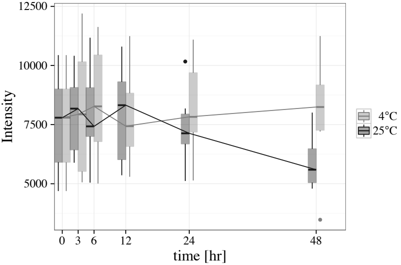

C18pos: m/z_t_R, 257.22576_10.01

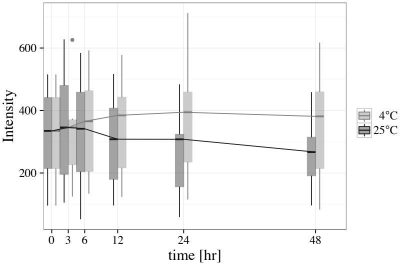

C18pos: m/z_t_R, 355.26321_8.81

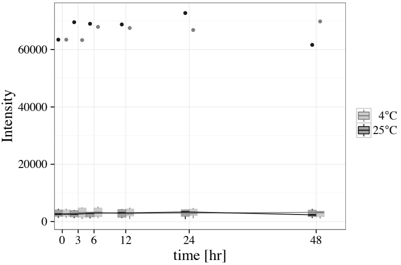

C18pos: m/z_t_R, 184.09427_0.82

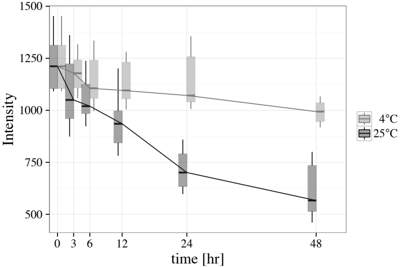

C18pos: m/z_t_R, 189.08871_0.99

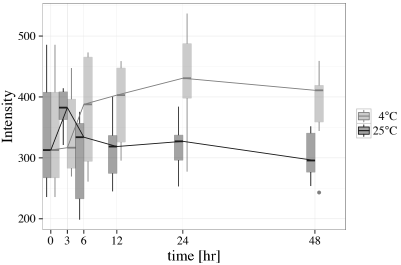

C18pos: m/z_t_R, 431.31578_10.28

C18pos: m/z_t_R, 204.13849_10.29

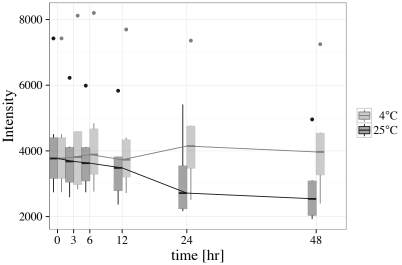

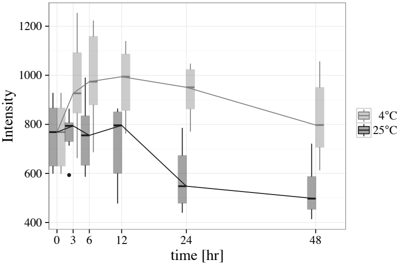

C18pos: m/z_t_R, 337.27507_9.79

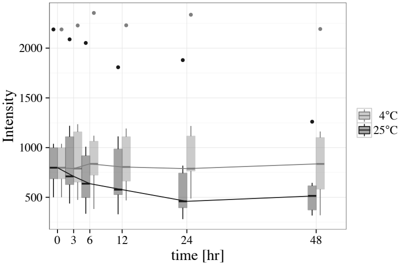

C18pos: m/z_t_R, 315.08111_1.03

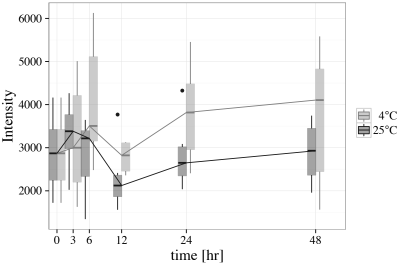

C18pos: m/z_t_R, 263.23777_10.31

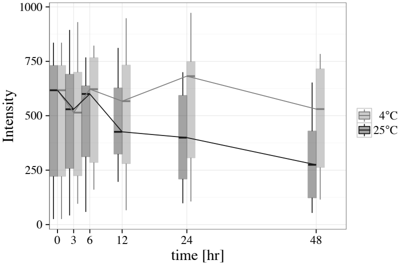

C18pos: m/z_t_R, 314.23379_7.39

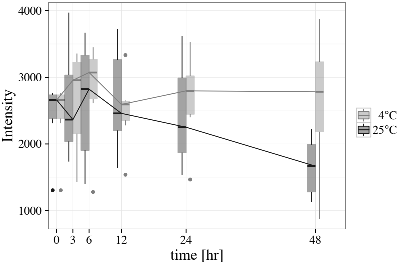

C18pos: m/z_t_R, 316.24805_7.55

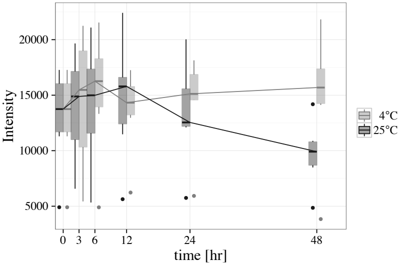

C18pos: m/z_t_R, 269.20957_10.43

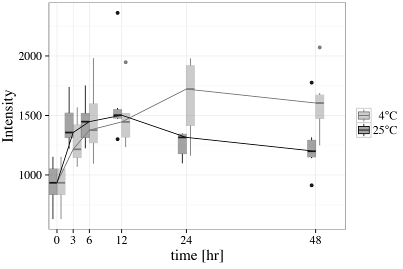

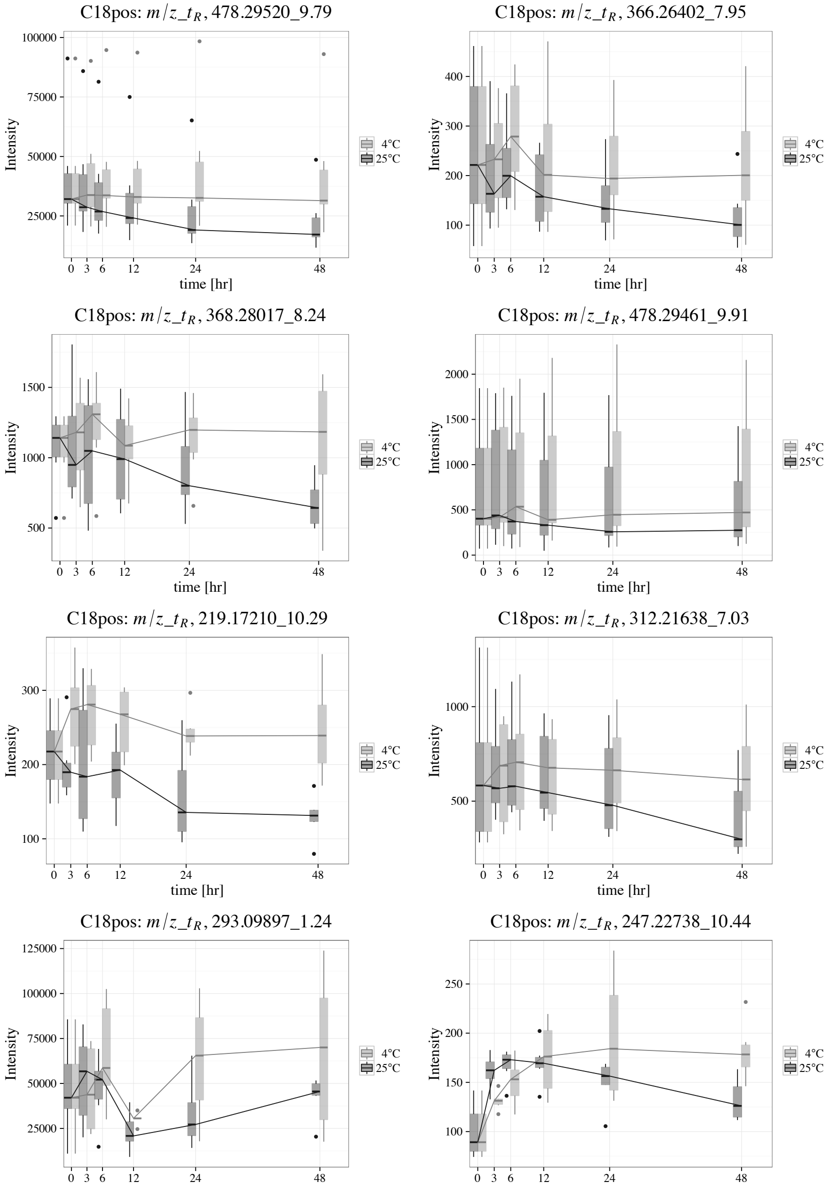
C18pos: m/z_t_R, 478.29520_9.79
Intensity
time [hr]
4°C
25°C
C18pos: m/z_t_R, 366.26402_7.95
Intensity
time [hr]
4°C
25°C
C18pos: m/z_t_R, 368.28017_8.24
Intensity
time [hr]
4°C
25°C
C18pos: m/z_t_R, 478.29461_9.91
Intensity
time [hr]
4°C
25°C
C18pos: m/z_t_R, 219.17210_10.29
Intensity
time [hr]
4°C
25°C
C18pos: m/z_t_R, 312.21638_7.03
Intensity
time [hr]
4°C
25°C
C18pos: m/z_t_R, 293.09897_1.24
Intensity
time [hr]
4°C
25°C
C18pos: m/z_t_R, 247.22738_10.44
Intensity
time [hr]
4°C
25°C

time [hr]

time [hr]

C18pos: m/z_t_R, 515.31386_10.11

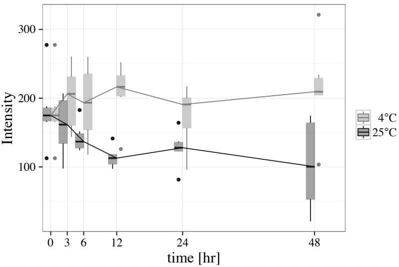

C18pos: m/z_t_R, 368.27901_8.35

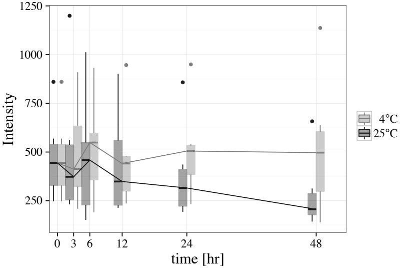

C18pos: m/z_t_R, 203.15049_0.92

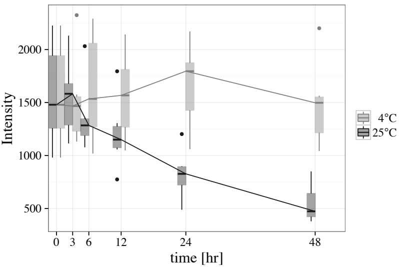

C18pos: m/z_t_R, 239.16508_10.15

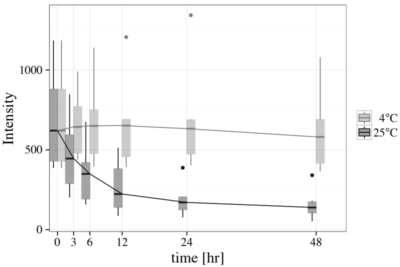

C18pos: m/z_t_R, 203.05412_0.87

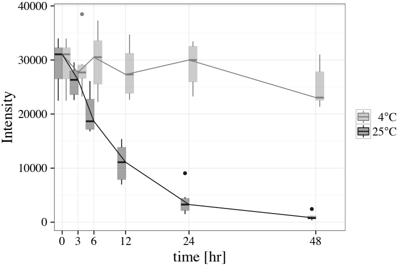

C18pos: m/z_t_R, 310.20204_6.99

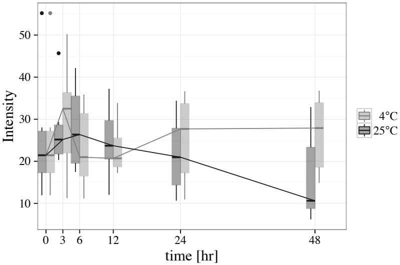

C18pos: m/z_t_R, 391.00727_0.80

C18pos: m/z_t_R, 923.80969_0.77

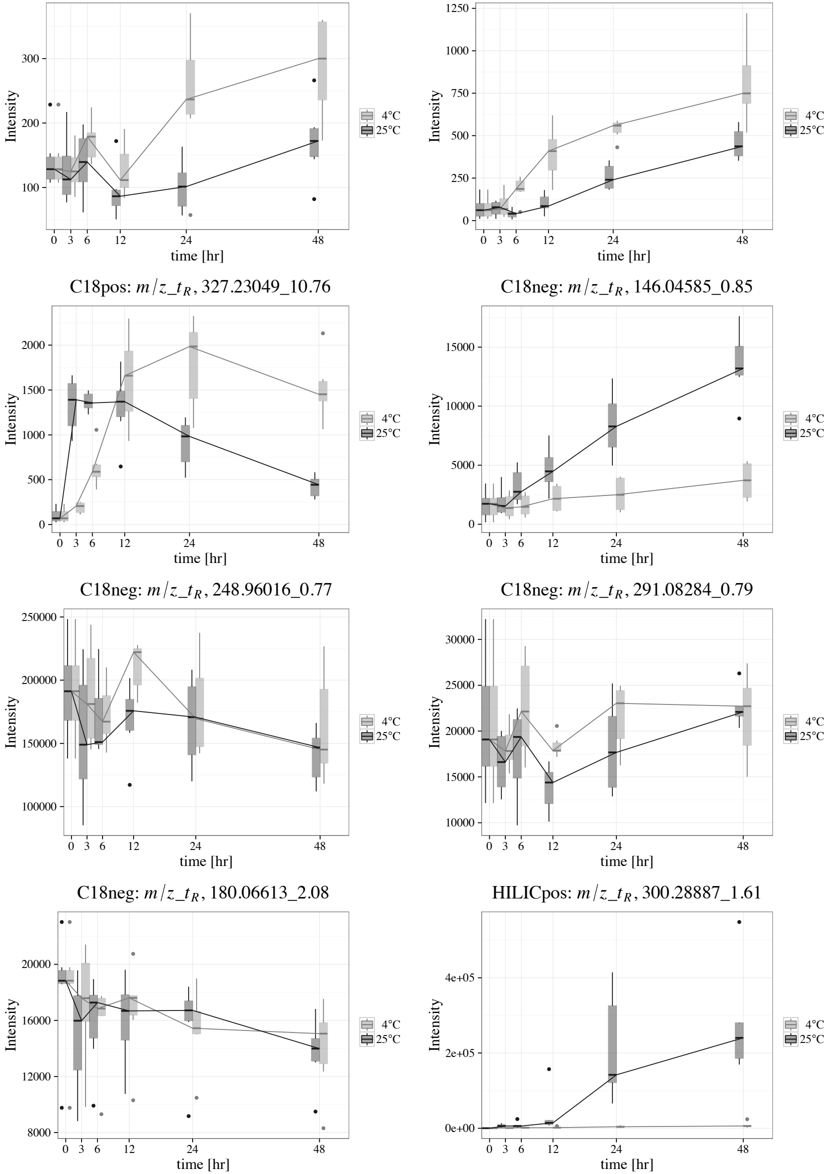

Intensity
time [hr]
4°C
25°C
C18pos: m/z_t_R, 327.23049_10.76
C18neg: m/z_t_R, 146.04585_0.85
C18neg: m/z_t_R, 248.96016_0.77
C18neg: m/z_t_R, 291.08284_0.79
C18neg: m/z_t_R, 180.06613_2.08
HILICpos: m/z_t_R, 300.28887_1.61

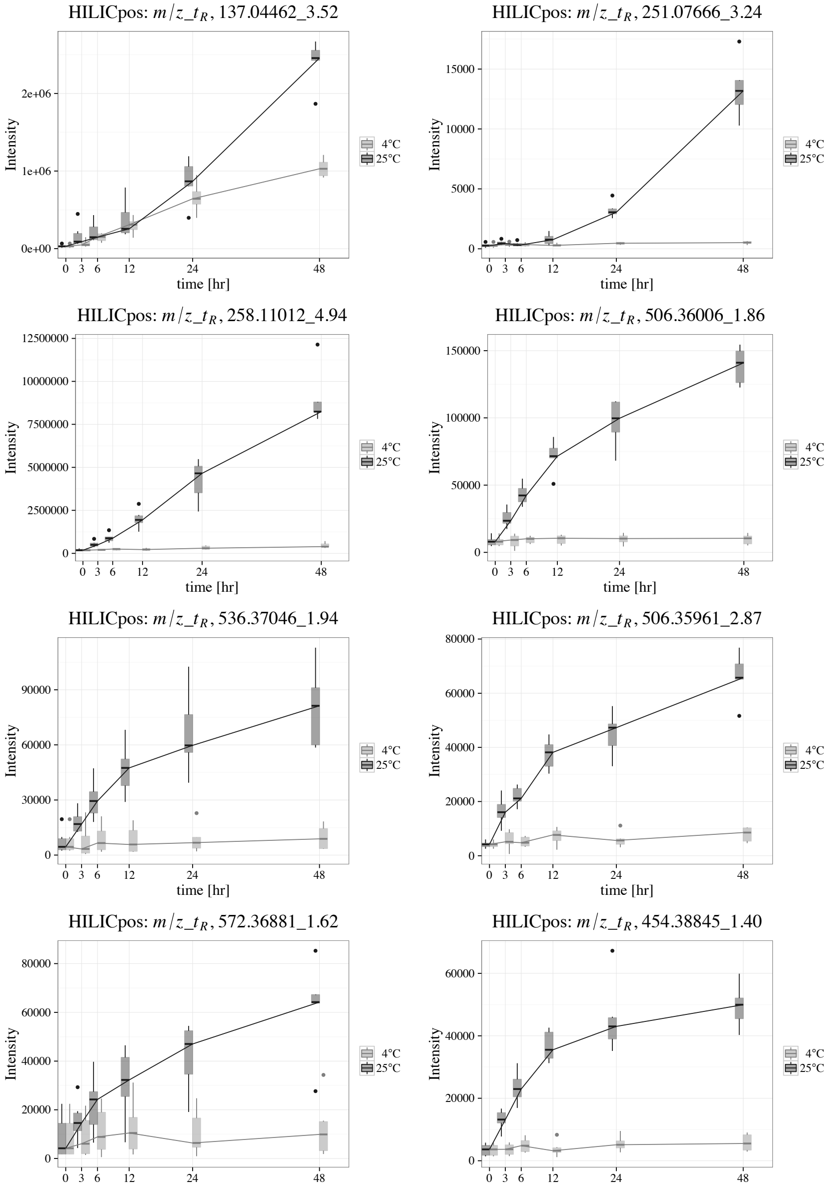
HILICpos: m/z_t_R, 137.04462_3.52
HILICpos: m/z_t_R, 251.07666_3.24
HILICpos: m/z_t_R, 258.11012_4.94
HILICpos: m/z_t_R, 506.36006_1.86
HILICpos: m/z_t_R, 536.37046_1.94
HILICpos: m/z_t_R, 506.35961_2.87
HILICpos: m/z_t_R, 572.36881_1.62
HILICpos: m/z_t_R, 454.38845_1.40
Intensity
time [hr]
0
3
6
12
24
48
4°C
25°C
0e+00
1e+06
2e+06
0
5000
10000
15000
2500000
5000000
7500000
10000000
12500000
50000
100000
150000
30000
60000
90000
20000
40000
60000
80000

time [hr] time [hr]

HILICpos: m/z_t_R, 538.38606_1.72

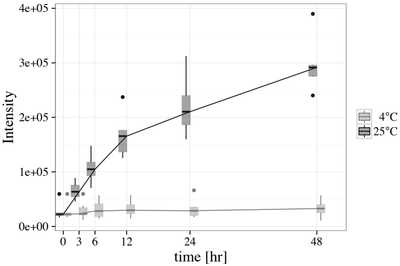

HILICpos: m/z_t_R, 466.32818_3.02

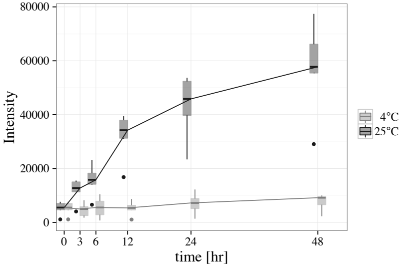

HILICpos: m/z_t_R, 508.37482_2.96

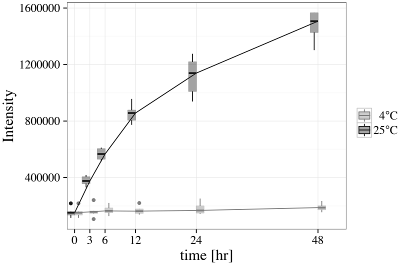

HILICpos: m/z_t_R, 510.39071_2.94

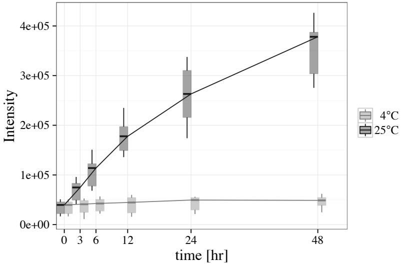

HILICpos: m/z_t_R, 175.10750_3.56

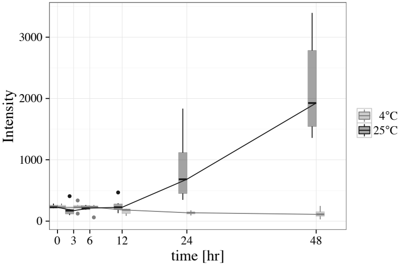

HILICpos: m/z_t_R, 245.18689_1.83

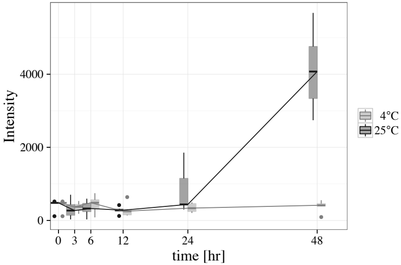

HILICpos: m/z_t_R, 159.02620_3.54

HILICpos: m/z_t_R, 123.05467_1.39

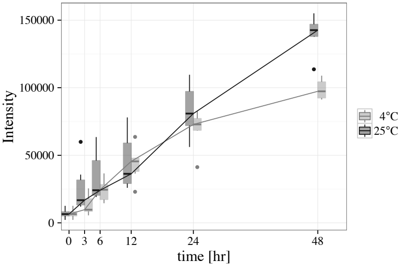

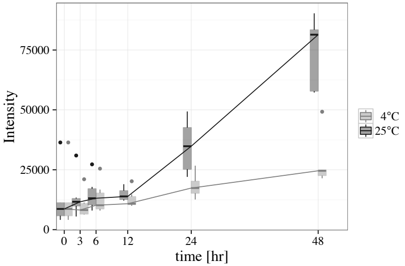

HILICpos: m/z_t_R, 480.34390_1.60

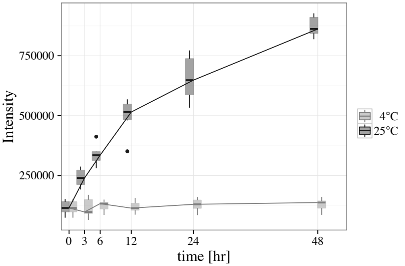

HILICpos: m/z_t_R, 578.41640_1.52

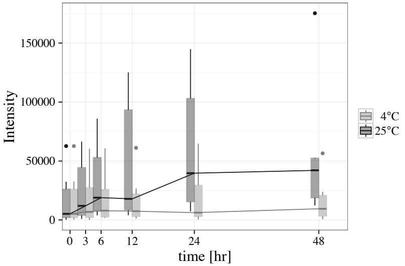

HILICpos: m/z_t_R, 200.05144_3.52

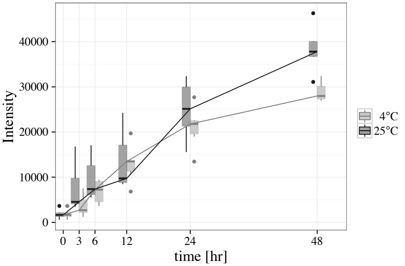

HILICpos: m/z_t_R, 550.38608_1.63

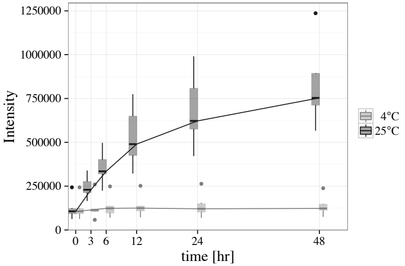

HILICpos: m/z_t_R, 261.14412_4.00

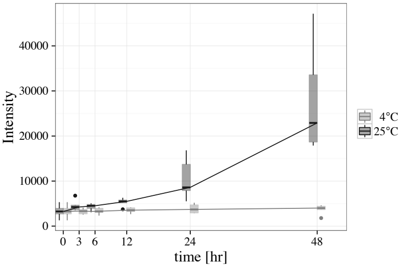

HILICpos: m/z_t_R, 422.32590_1.57

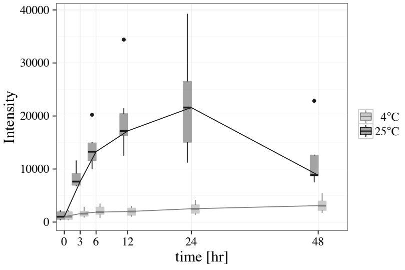

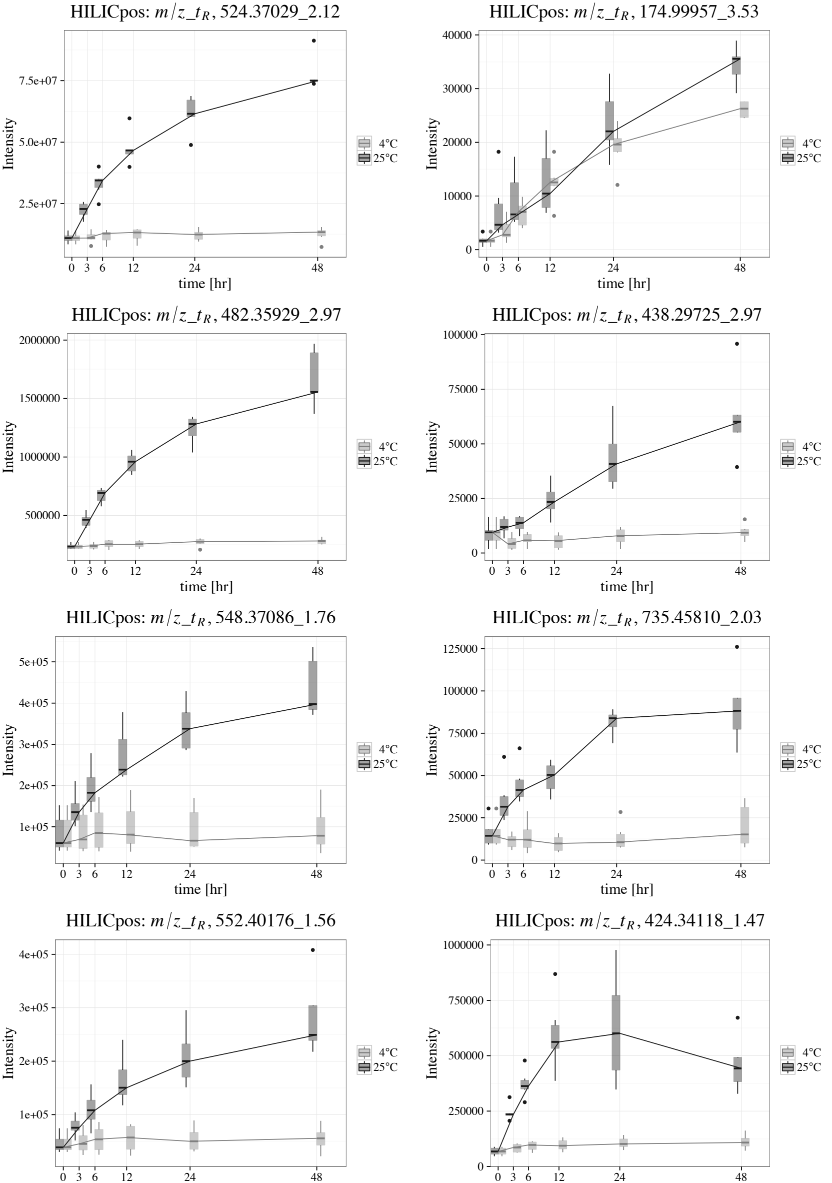
HILICpos: m/z_t_R, 524.37029_2.12
Intensity
7.5e+07
5.0e+07
2.5e+07
0 3 6 12 24 48
time [hr]
4°C
25°C
HILICpos: m/z_t_R, 174.99957_3.53
Intensity
40000
30000
20000
10000
0
0 3 6 12 24 48
time [hr]
4°C
25°C
HILICpos: m/z_t_R, 482.35929_2.97
Intensity
2000000
1500000
1000000
500000
0 3 6 12 24 48
time [hr]
4°C
25°C
HILICpos: m/z_t_R, 438.29725_2.97
Intensity
100000
75000
50000
25000
0
0 3 6 12 24 48
time [hr]
4°C
25°C
HILICpos: m/z_t_R, 548.37086_1.76
Intensity
5e+05
4e+05
3e+05
2e+05
1e+05
0 3 6 12 24 48
time [hr]
4°C
25°C
HILICpos: m/z_t_R, 735.45810_2.03
Intensity
125000
100000
75000
50000
25000
0
0 3 6 12 24 48
time [hr]
4°C
25°C
HILICpos: m/z_t_R, 552.40176_1.56
Intensity
4e+05
3e+05
2e+05
1e+05
0 3 6 12 24 48
4°C
25°C
HILICpos: m/z_t_R, 424.34118_1.47
Intensity
1000000
750000
500000
250000
0
0 3 6 12 24 48
4°C
25°C

time [hr]

time [hr]

HILICpos: m/z_t_R, 580.43289_1.53

HILICpos: m/z_t_R, 145.04289_4.55

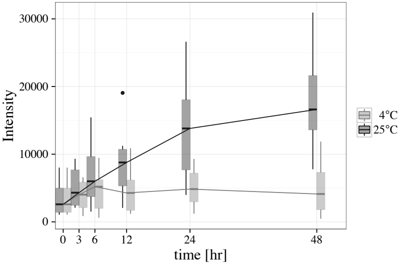

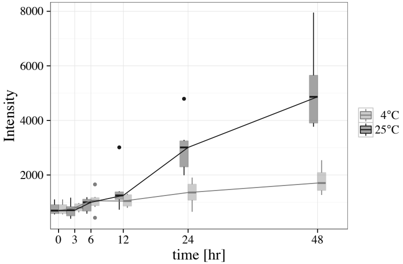

HILICpos: m/z_t_R, 347.16090_0.88

HILICpos: m/z_t_R, 427.09471_5.29

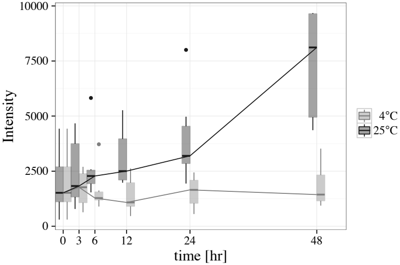

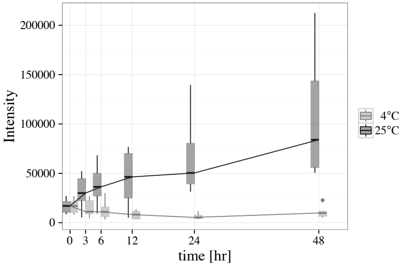

HILICpos: m/z_t_R, 480.34379_2.90

HILICpos: m/z_t_R, 510.35448_2.92

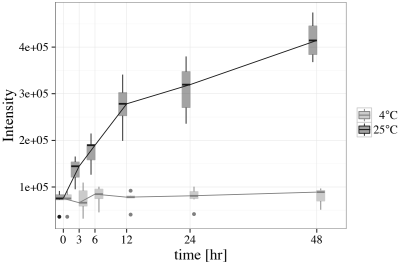

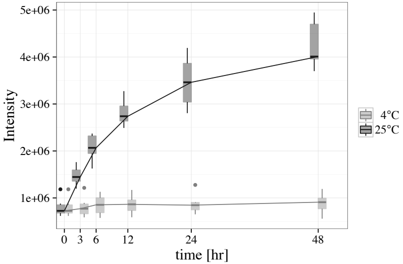

HILICpos: m/z_t_R, 247.12768_4.27

HILICpos: m/z_t_R, 752.55492_1.03

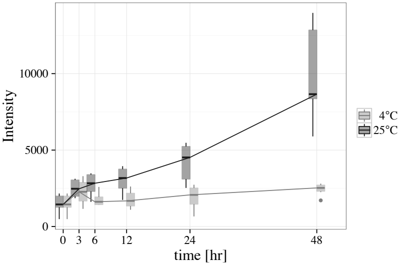

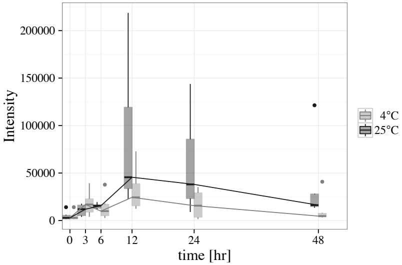

HILICpos: m/z_t_R, 538.38585_2.89

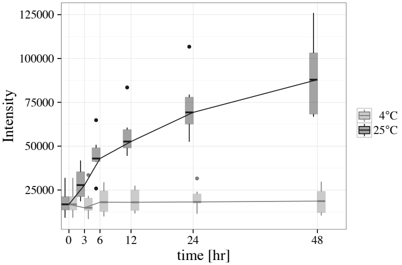

HILICpos: m/z_t_R, 536.37030_2.90

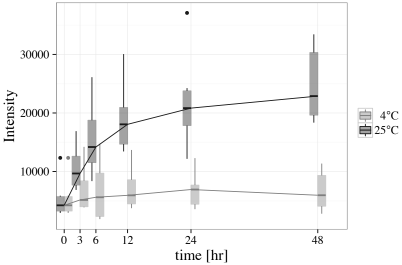

HILICpos: m/z_t_R, 259.16451_3.25

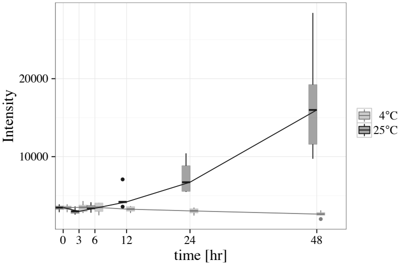

HILICpos: m/z_t_R, 426.35690_1.44

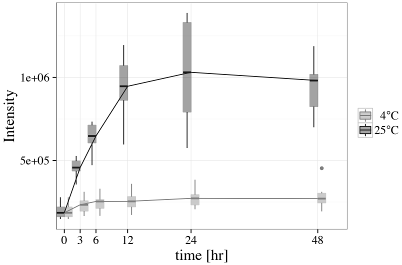

HILICpos: m/z_t_R, 428.37273_1.42

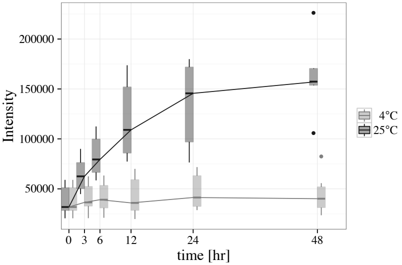

HILICpos: m/z_t_R, 524.37020_2.90

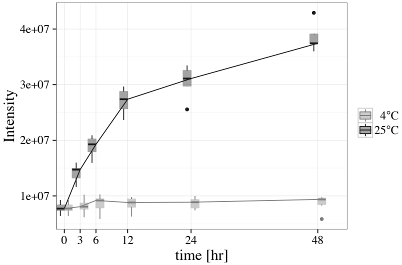

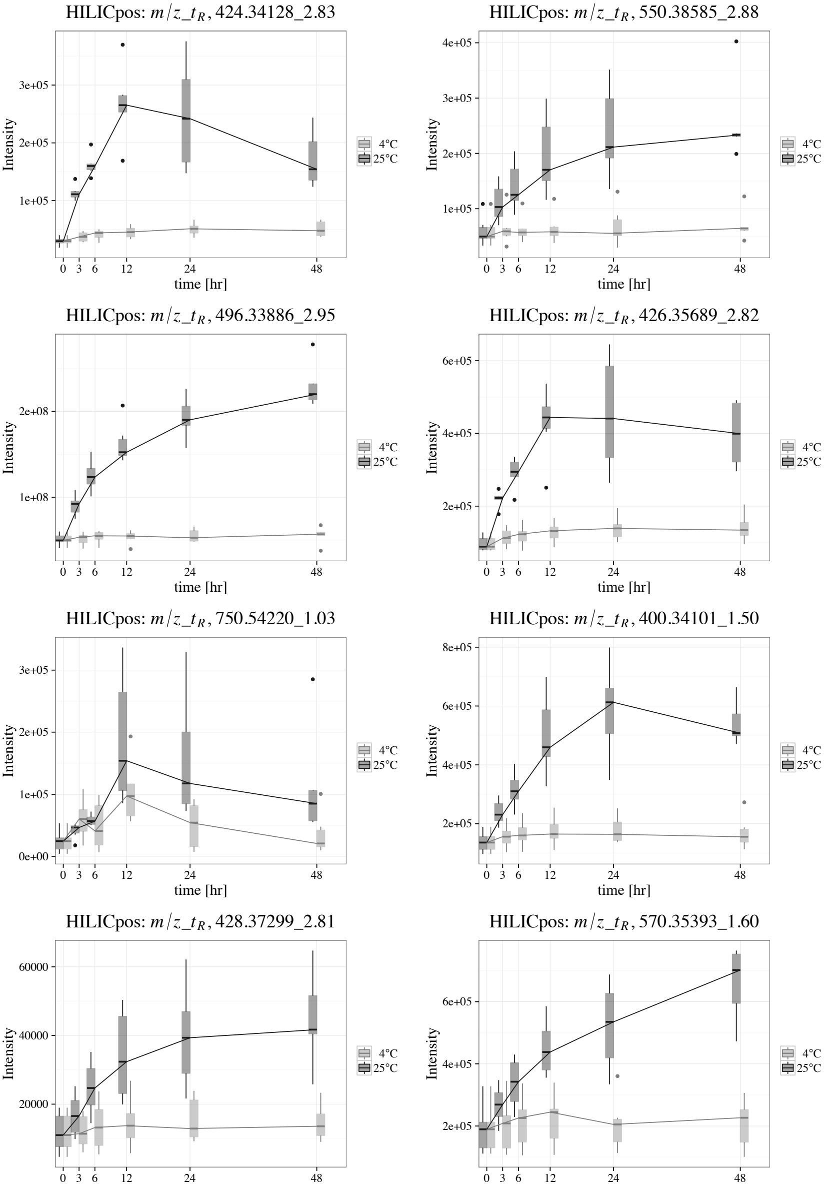
HILICpos: m/z_t_R, 424.34128_2.83
HILICpos: m/z_t_R, 550.38585_2.88
HILICpos: m/z_t_R, 496.33886_2.95
HILICpos: m/z_t_R, 426.35689_2.82
HILICpos: m/z_t_R, 750.54220_1.03
HILICpos: m/z_t_R, 400.34101_1.50
HILICpos: m/z_t_R, 428.37299_2.81
HILICpos: m/z_t_R, 570.35393_1.60
Intensity
time [hr]
0 3 6 12 24 48
4°C
25°C
1e+05
2e+05
3e+05
4e+05
6e+05
8e+05
0e+00
1e+08
2e+08
20000
40000
60000

time [hr]

time [hr]

HILICpos: m/z_t_R, 230.09431_4.89

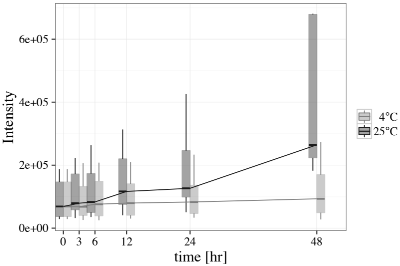

HILICpos: m/z_t_R, 548.37046_2.90

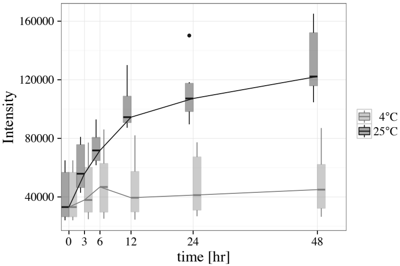

HILICpos: m/z_t_R, 104.10650_6.17

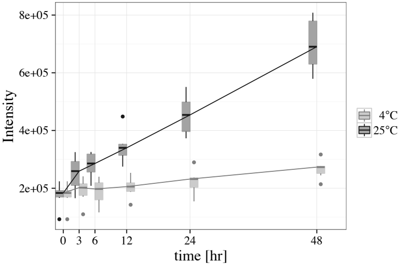

HILICpos: m/z_t_R, 552.40166_2.86

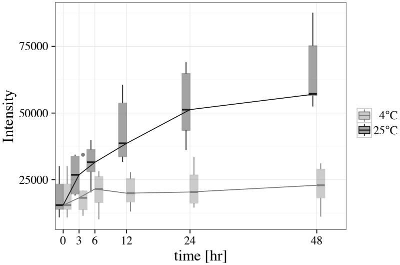

HILICpos: m/z_t_R, 310.11271_4.45

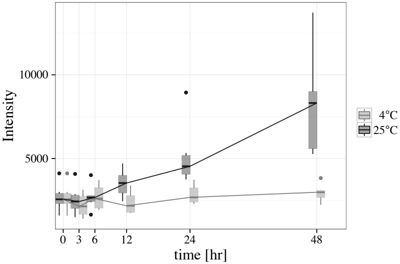

HILICpos: m/z_t_R, 544.33899_2.12

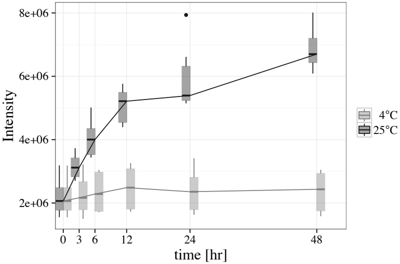

HILICpos: m/z_t_R, 468.30742_2.99

HILICpos: m/z_t_R, 568.33837_1.59

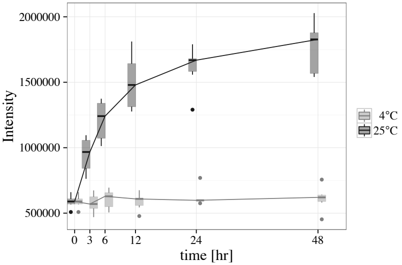

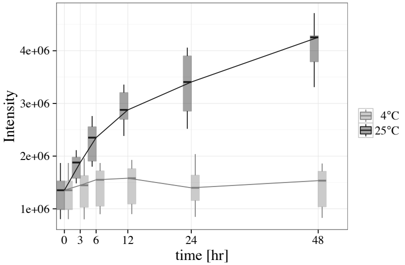

HILICpos: m/z_t_R, 626.53262_0.68

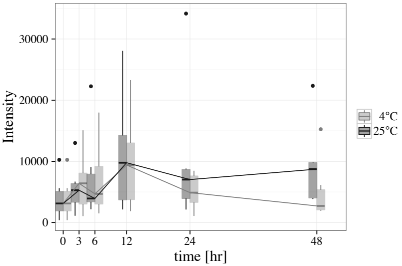

HILICpos: m/z_t_R, 538.51897_0.70

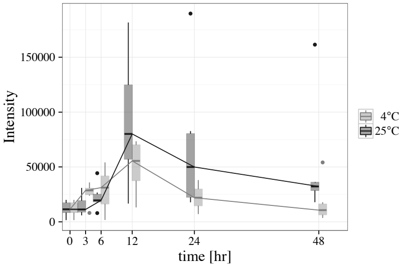

HILICpos: m/z_t_R, 313.15501_0.93

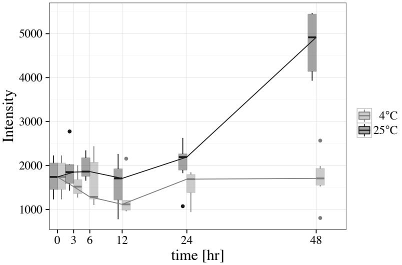

HILICpos: m/z_t_R, 522.35478_2.91

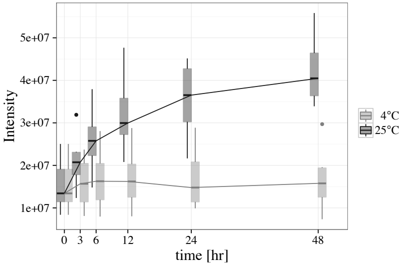

HILICpos: m/z_t_R, 400.34102_2.86

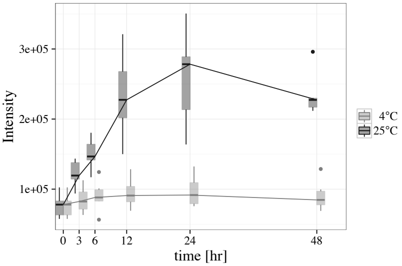

HILICpos: m/z_t_R, 546.35476_1.68

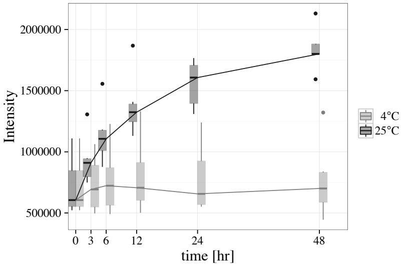

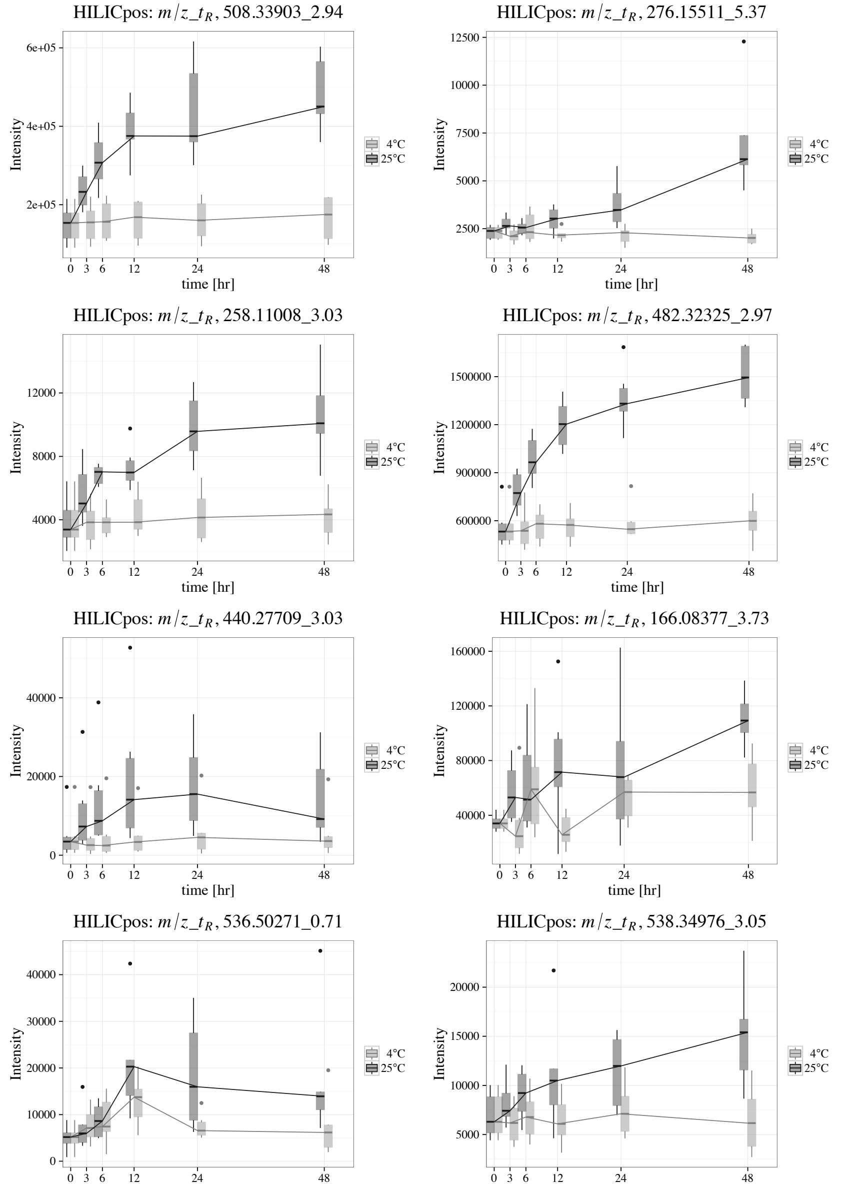
HILICpos: m/z_t_R, 508.33903_2.94
Intensity
6e+05
4e+05
2e+05
time [hr]
0 3 6 12 24 48
4°C
25°C
HILICpos: m/z_t_R, 276.15511_5.37
Intensity
12500
10000
7500
5000
2500
time [hr]
0 3 6 12 24 48
4°C
25°C
HILICpos: m/z_t_R, 258.11008_3.03
Intensity
12000
8000
4000
time [hr]
0 3 6 12 24 48
4°C
25°C
HILICpos: m/z_t_R, 482.32325_2.97
Intensity
1500000
1200000
900000
600000
time [hr]
0 3 6 12 24 48
4°C
25°C
HILICpos: m/z_t_R, 440.27709_3.03
Intensity
40000
20000
0
time [hr]
0 3 6 12 24 48
4°C
25°C
HILICpos: m/z_t_R, 166.08377_3.73
Intensity
160000
120000
80000
40000
time [hr]
0 3 6 12 24 48
4°C
25°C
HILICpos: m/z_t_R, 536.50271_0.71
Intensity
40000
30000
20000
10000
0
0 3 6 12 24 48
4°C
25°C
HILICpos: m/z_t_R, 538.34976_3.05
Intensity
20000
15000
10000
5000
0 3 6 12 24 48
4°C
25°C

time [hr]

HILICpos: m/z_t_R, 764.52196_1.02

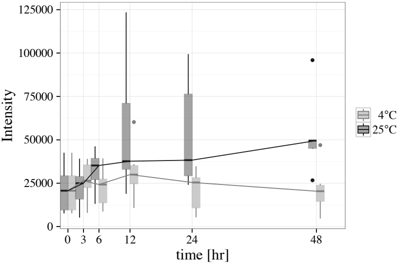

HILICpos: m/z_t_R, 116.07083_6.61

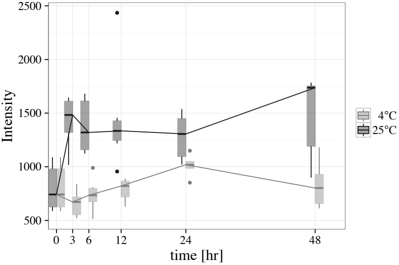

HILICpos: m/z_t_R, 544.33892_2.90

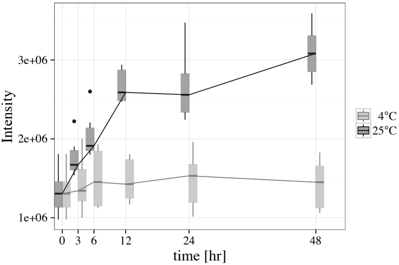

HILICpos: m/z_t_R, 255.09680_7.70

time [hr]

HILICpos: m/z_t_R, 298.05238_4.89

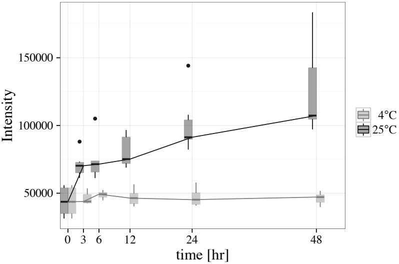

HILICpos: m/z_t_R, 494.32328_2.96

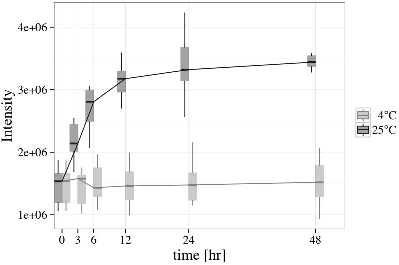

HILICpos: m/z_t_R, 542.32299_2.91

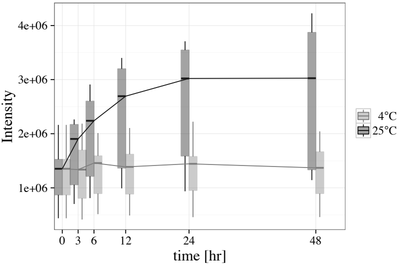

HILICpos: m/z_t_R, 570.35382_2.89

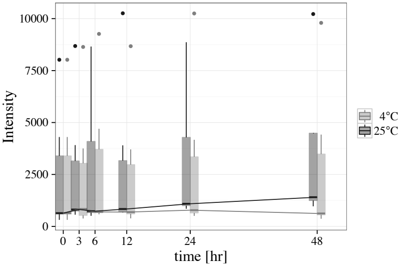

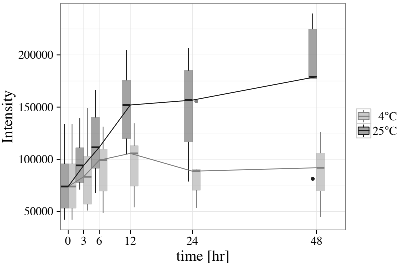

HILICpos: m/z_t_R, 288.28895_0.85

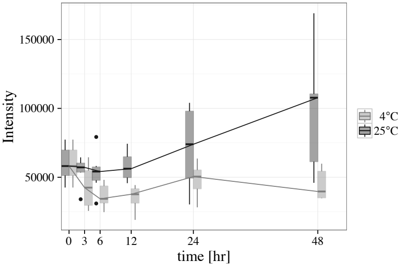

HILICpos: m/z_t_R, 398.32522_1.59

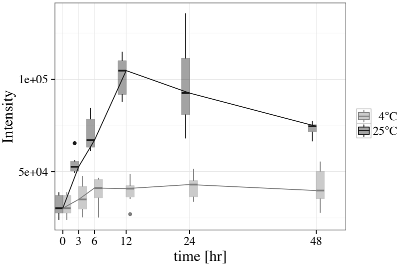

HILICpos: m/z_t_R, 262.16457_4.02

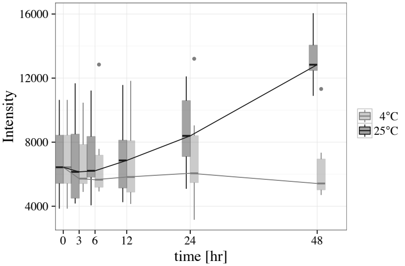

HILICpos: m/z_t_R, 608.46413_1.44

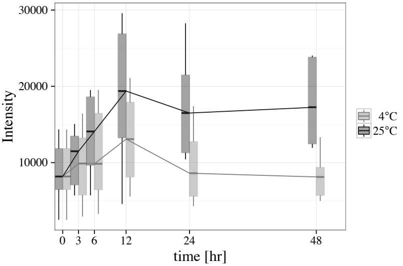

HILICpos: m/z_t_R, 352.22379_3.34

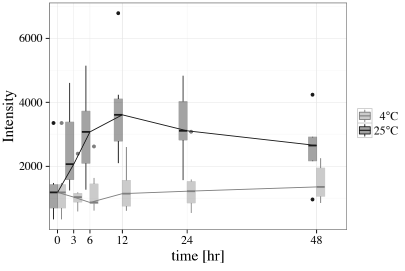

HILICpos: m/z_t_R, 628.54810_0.62

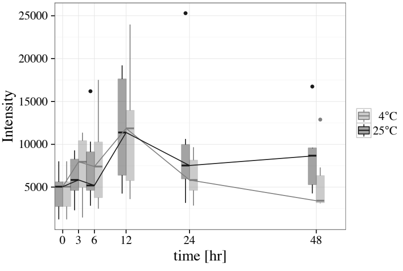

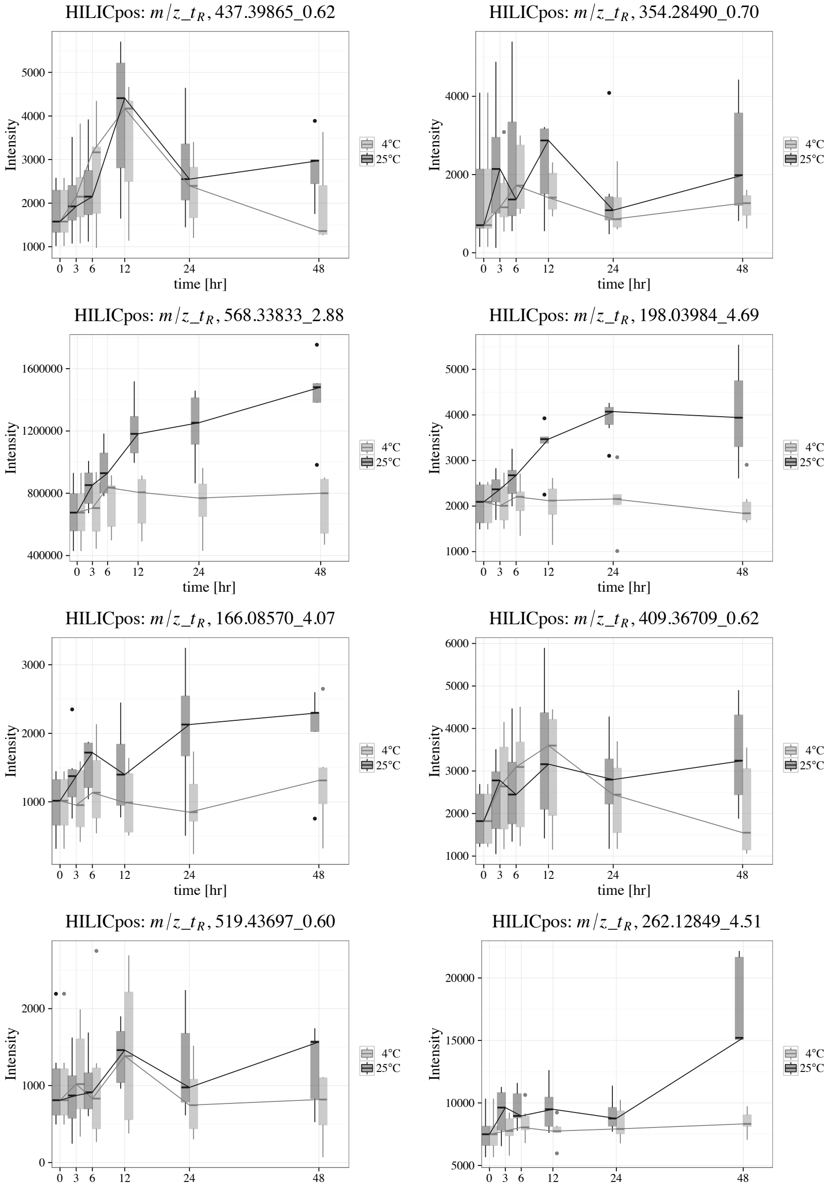
HILICpos: m/z_t_R, 437.39865_0.62
Intensity
1000
2000
3000
4000
5000
time [hr]
0
3
6
12
24
48
4°C
25°C
HILICpos: m/z_t_R, 354.28490_0.70
Intensity
0
2000
4000
time [hr]
0
3
6
12
24
48
4°C
25°C
HILICpos: m/z_t_R, 568.33833_2.88
Intensity
400000
800000
1200000
1600000
time [hr]
0
3
6
12
24
48
4°C
25°C
HILICpos: m/z_t_R, 198.03984_4.69
Intensity
1000
2000
3000
4000
5000
time [hr]
0
3
6
12
24
48
4°C
25°C
HILICpos: m/z_t_R, 166.08570_4.07
Intensity
1000
2000
3000
time [hr]
0
3
6
12
24
48
4°C
25°C
HILICpos: m/z_t_R, 409.36709_0.62
Intensity
1000
2000
3000
4000
5000
6000
time [hr]
0
3
6
12
24
48
4°C
25°C
HILICpos: m/z_t_R, 519.43697_0.60
Intensity
0
1000
2000
0
3
6
12
24
48
4°C
25°C
HILICpos: m/z_t_R, 262.12849_4.51
Intensity
5000
10000
15000
20000
0
3
6
12
24
48
4°C
25°C

time [hr]

time [hr]

HILICpos: m/z_t_R, 316.31974_0.81

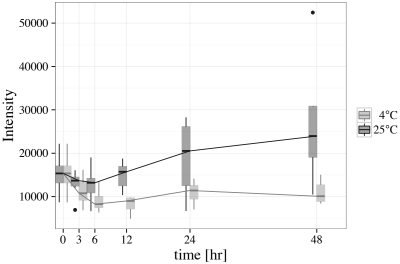

HILICpos: m/z_t_R, 799.66511_1.37

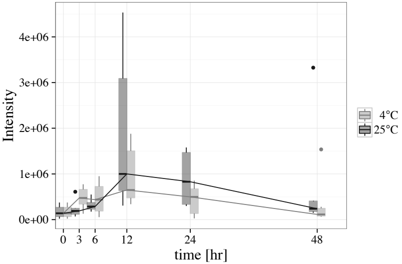

HILICpos: m/z_t_R, 134.06336_0.92

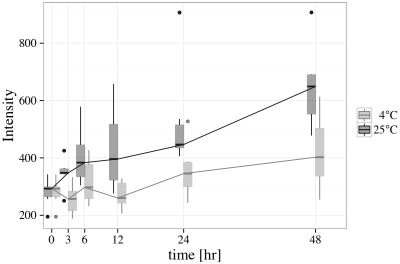

HILICpos: m/z_t_R, 349.27280_0.64

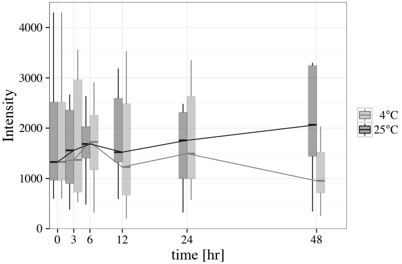

HILICpos: m/z_t_R, 822.56089_1.33

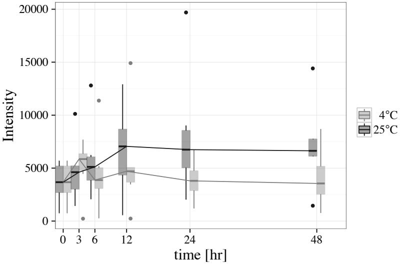

HILICpos: m/z_t_R, 838.55442_0.99

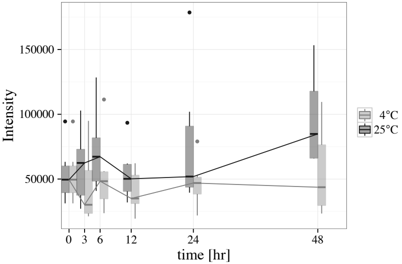

HILICpos: m/z_t_R, 449.36106_0.62

HILICpos: m/z_t_R, 486.11331_4.85

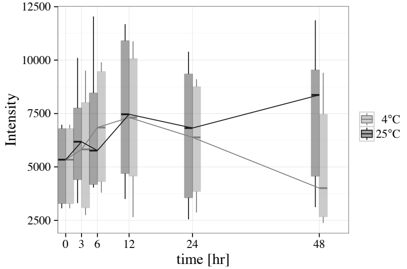

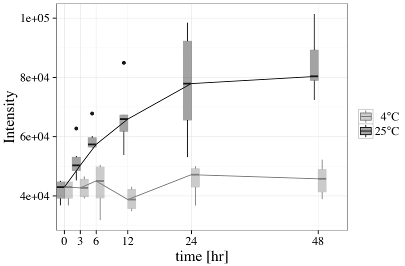

HILICpos: m/z_t_R, 326.30452_0.75

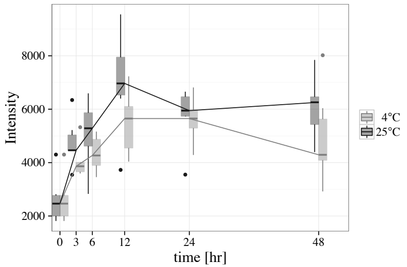

HILICpos: m/z_t_R, 108.05519_4.82

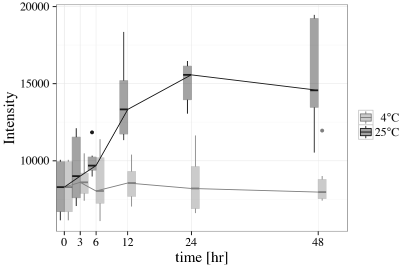

HILICpos: m/z_t_R, 421.09478_4.85

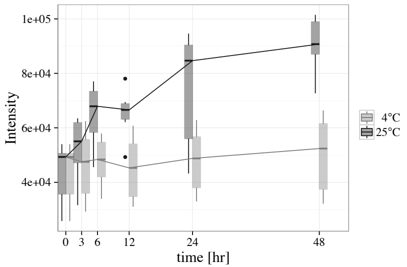

HILICpos: m/z_t_R, 466.31645_3.07

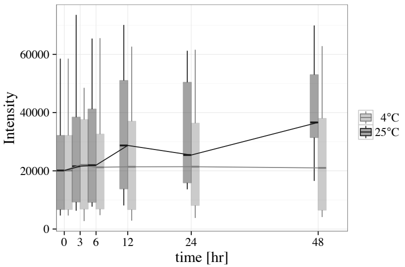

HILICpos: m/z_t_R, 829.67466_1.41

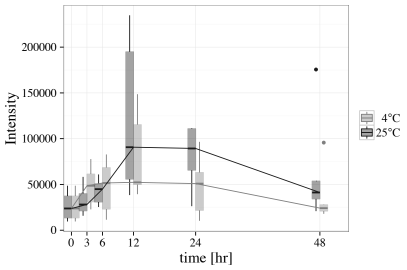

HILICpos: m/z_t_R, 262.14064_5.65

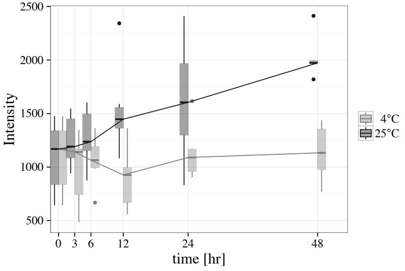

HILICpos: m/z_t_R, 204.14229_4.49

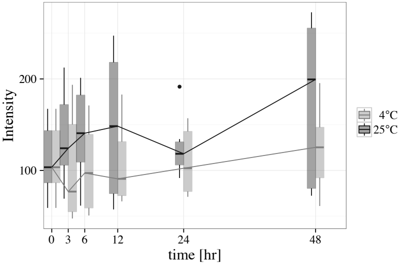

HILICpos: m/z_t_R, 70.06520_6.56

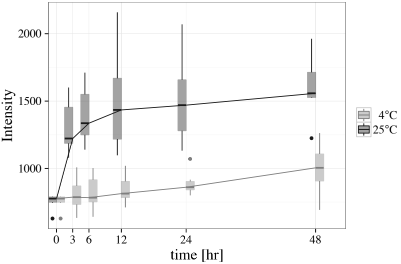

HILICpos: m/z_t_R, 420.09207_4.85

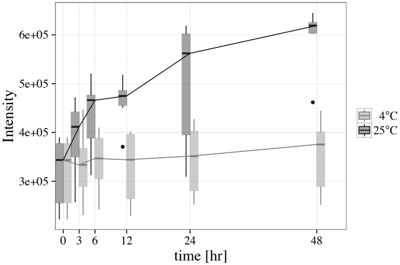

HILICpos: m/z_t_R, 746.60536_1.03

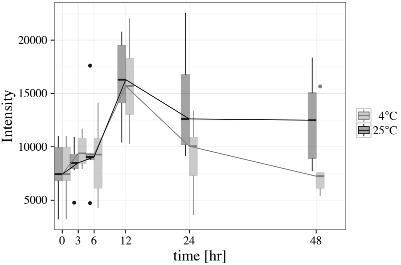

HILICpos: m/z_t_R, 468.40338_0.63

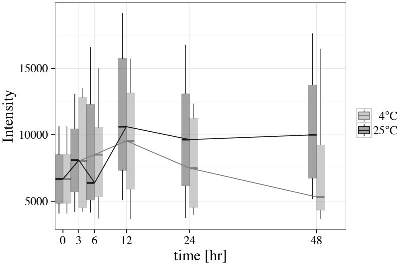

HILICpos: m/z_t_R, 774.60378_1.03

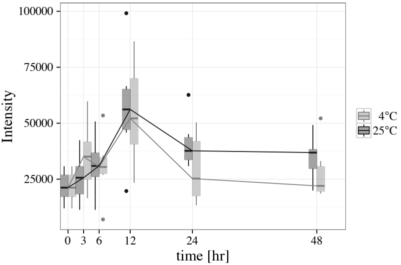

HILICpos: m/z_t_R, 581.23930_0.75

HILICpos: m/z_t_R, 133.10432_7.08

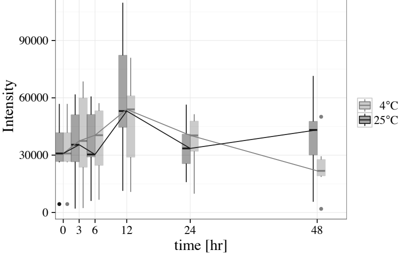

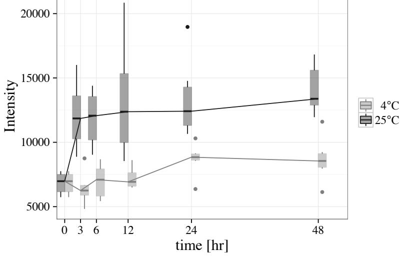

HILICpos: m/z_t_R, 366.37215_0.71

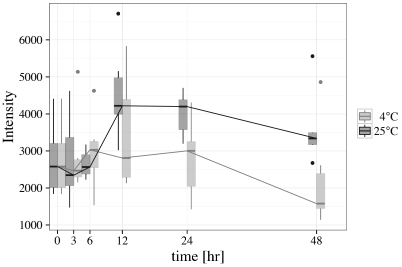

HILICpos: m/z_t_R, 746.60365_1.15

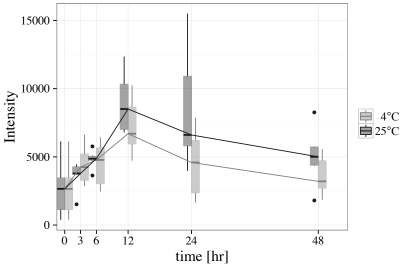

HILICpos: m/z_t_R, 506.38387_0.90

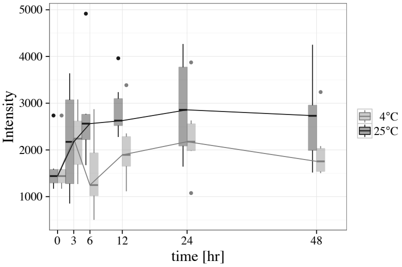

HILICpos: m/z_t_R, 298.27286_0.76

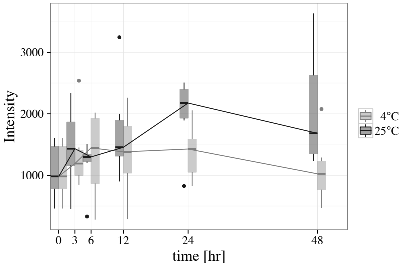

HILICpos: m/z_t_R, 470.38280_0.63

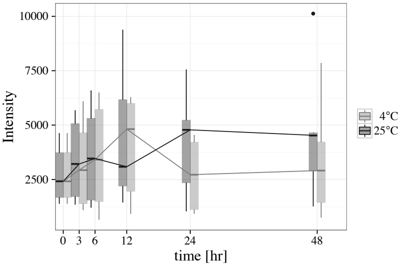

HILICpos: m/z_t_R, 147.11282_6.29

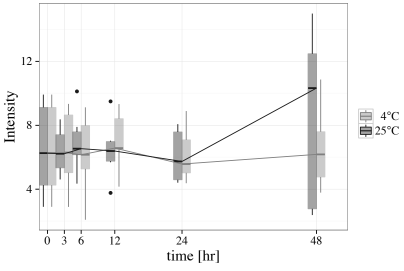

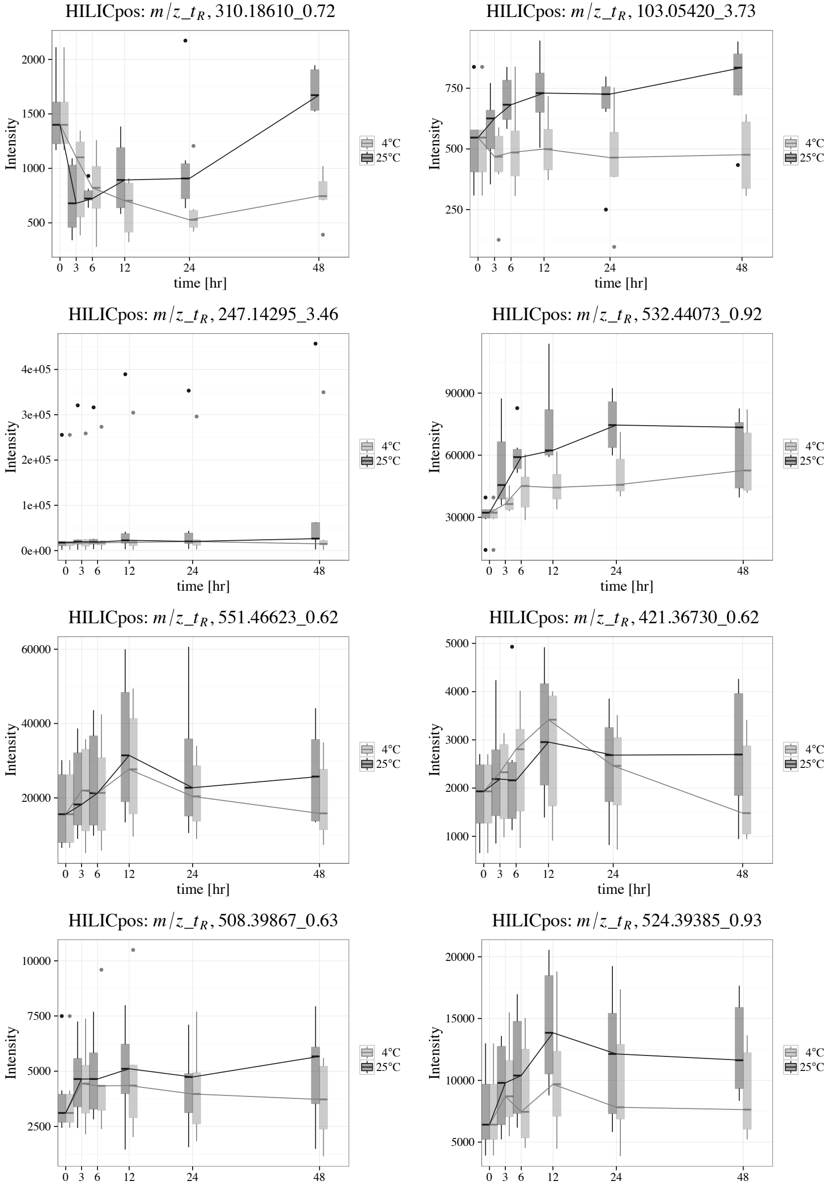
HILICpos: m/z_t_R, 310.18610_0.72
HILICpos: m/z_t_R, 103.05420_3.73
HILICpos: m/z_t_R, 247.14295_3.46
HILICpos: m/z_t_R, 532.44073_0.92
HILICpos: m/z_t_R, 551.46623_0.62
HILICpos: m/z_t_R, 421.36730_0.62
HILICpos: m/z_t_R, 508.39867_0.63
HILICpos: m/z_t_R, 524.39385_0.93
Intensity
time [hr]
0
3
6
12
24
48
4°C
25°C

time [hr]

time [hr]

HILICpos: m/z_t_R, 439.37770_0.63

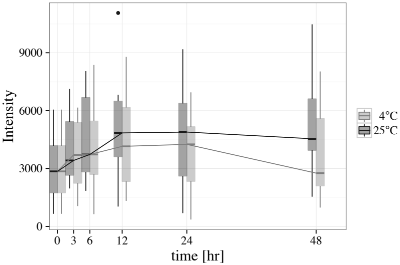

HILICpos: m/z_t_R, 264.11896_5.07

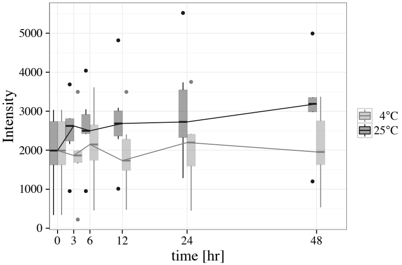

HILICpos: m/z_t_R, 148.04195_2.68

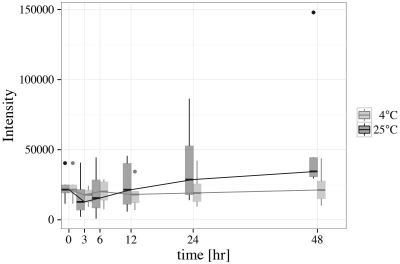

HILICpos: m/z_t_R, 583.25441_0.76

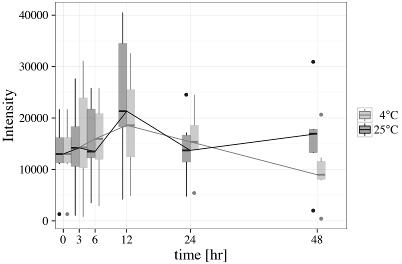

HILICpos: m/z_t_R, 744.58648_1.71

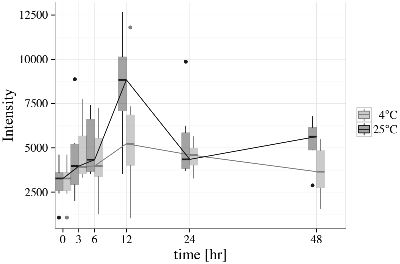

HILICpos: m/z_t_R, 433.36716_0.68

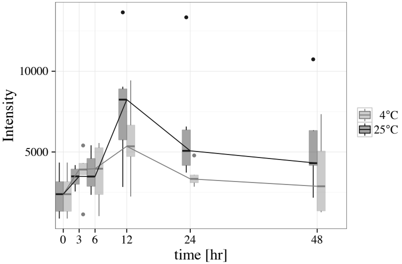

HILICpos: m/z_t_R, 378.23940_3.30

HILICpos: m/z_t_R, 248.14816_4.18

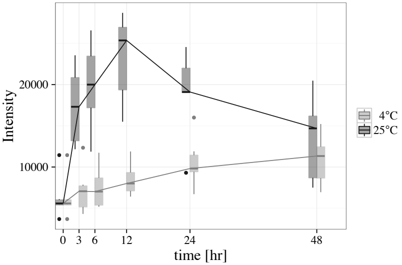

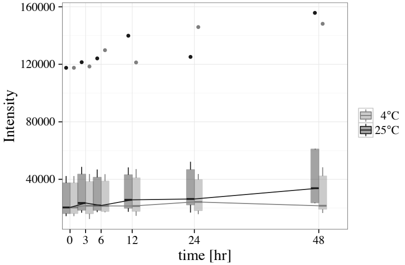

HILICpos: m/z_t_R, 547.43555_0.80

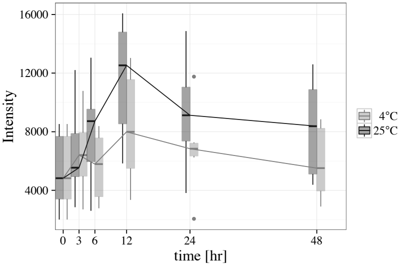

HILICpos: m/z_t_R, 111.05470_4.81

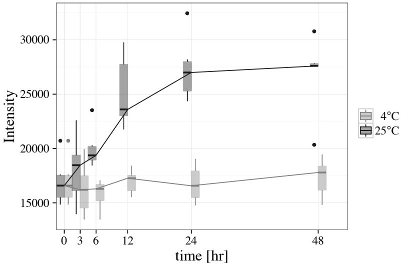

HILICpos: m/z_t_R, 553.48284_0.72

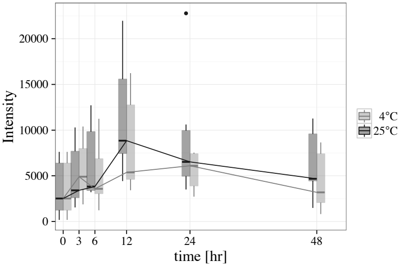

HILICpos: m/z_t_R, 581.51356_0.72

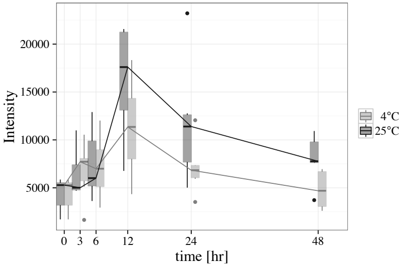

HILICpos: m/z_t_R, 484.34729_3.08

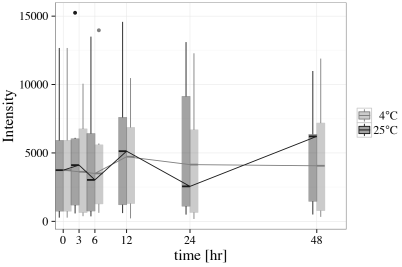

HILICpos: m/z_t_R, 485.33283_3.15

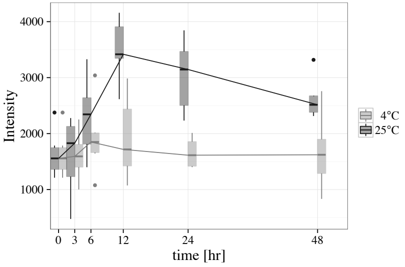

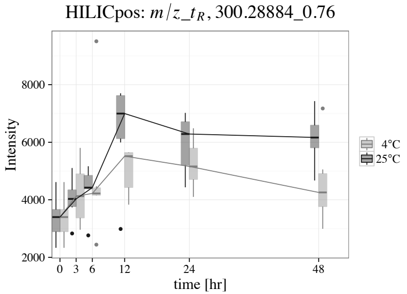
HILICpos: m/z_tR, 300.28884_0.76
Intensity
2000
4000
6000
8000
time [hr]
0 3 6 12 24 48
4°C
25°C

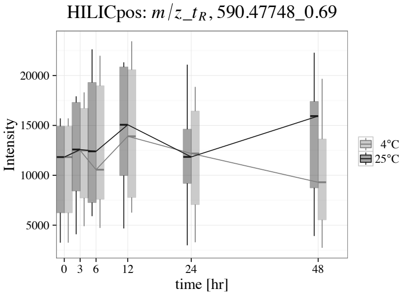
HILICpos: m/z_tR, 590.47748_0.69
Intensity
5000
10000
15000
20000
time [hr]
0 3 6 12 24 48
4°C
25°C

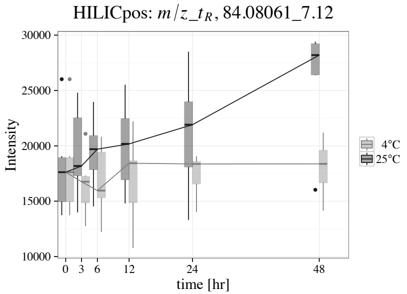
HILICpos: m/z_tR, 84.08061_7.12
Intensity
10000
15000
20000
25000
30000
time [hr]
0 3 6 12 24 48
4°C
25°C

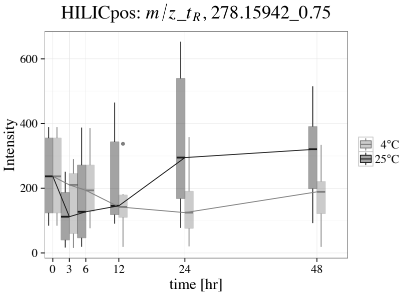
HILICpos: m/z_tR, 278.15942_0.75
Intensity
0
200
400
600
time [hr]
0 3 6 12 24 48
4°C
25°C

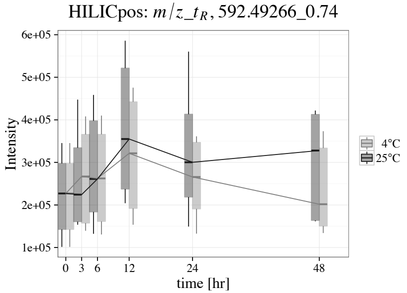
HILICpos: m/z_tR, 592.49266_0.74
Intensity
1e+05
2e+05
3e+05
4e+05
5e+05
6e+05
time [hr]
0 3 6 12 24 48
4°C
25°C

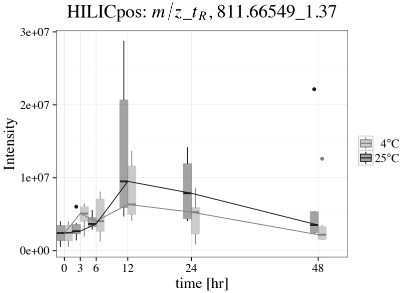
HILICpos: m/z_tR, 811.66549_1.37
Intensity
0e+00
1e+07
2e+07
3e+07
time [hr]
0 3 6 12 24 48
4°C
25°C

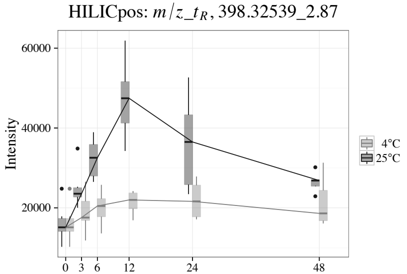
HILICpos: m/z_tR, 398.32539_2.87
Intensity
20000
40000
60000
0 3 6 12 24 48
4°C
25°C

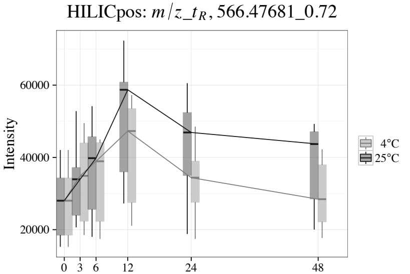
HILICpos: m/z_tR, 566.47681_0.72
Intensity
20000
40000
60000
0 3 6 12 24 48
4°C
25°C

time [hr]

time [hr]

HILICpos: m/z_t_R, 798.56387_0.99

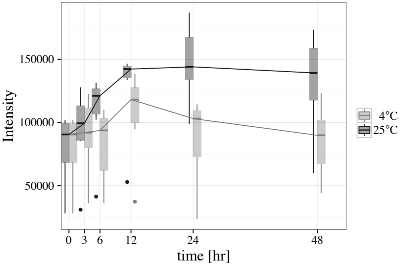

HILICpos: m/z_t_R, 456.36727_0.92

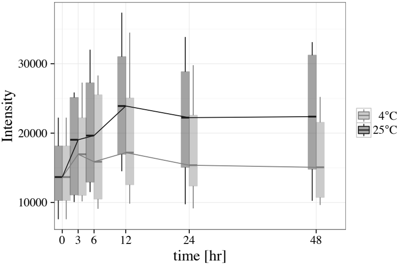

HILICpos: m/z_t_R, 166.08333_6.39

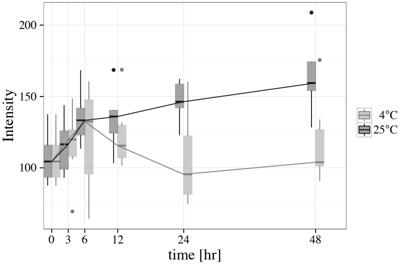

HILICpos: m/z_t_R, 166.08361_5.27

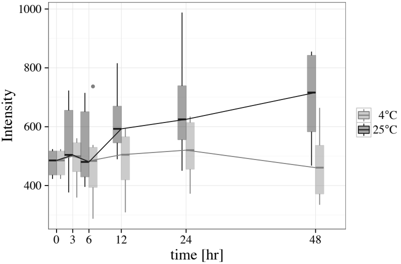

HILICpos: m/z_t_R, 283.10316_3.21

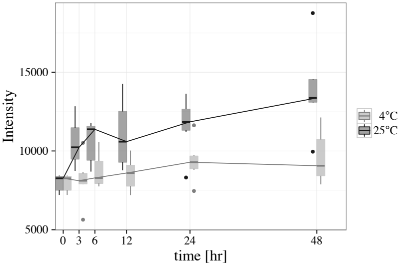

HILICpos: m/z_t_R, 84.08070_7.30

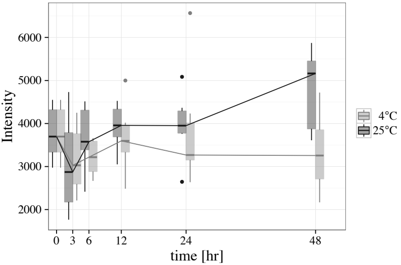

HILICpos: m/z_t_R, 523.43541_0.62

HILICpos: m/z_t_R, 166.08402_5.52

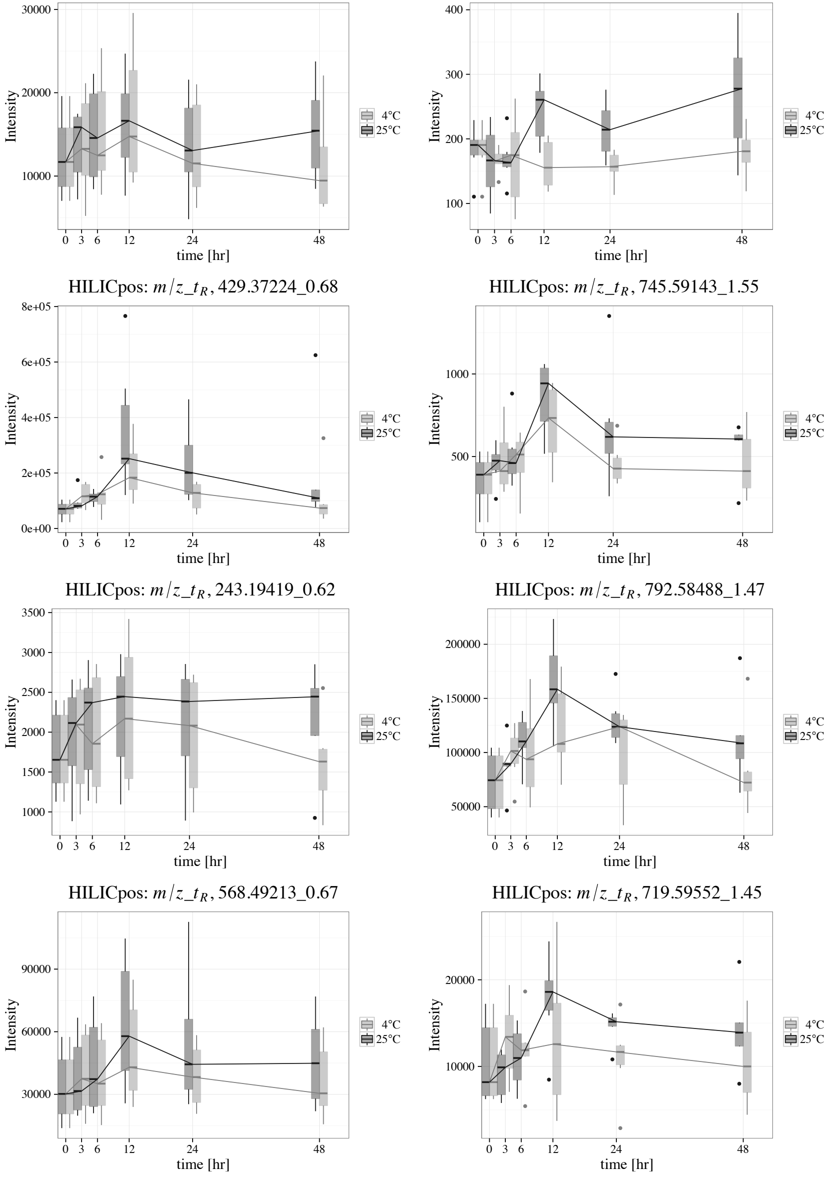

Intensity
time [hr]
4°C
25°C
HILICpos: m/z_t_R, 429.37224_0.68
HILICpos: m/z_t_R, 745.59143_1.55
HILICpos: m/z_t_R, 243.19419_0.62
HILICpos: m/z_t_R, 792.58488_1.47
HILICpos: m/z_t_R, 568.49213_0.67
HILICpos: m/z_t_R, 719.59552_1.45

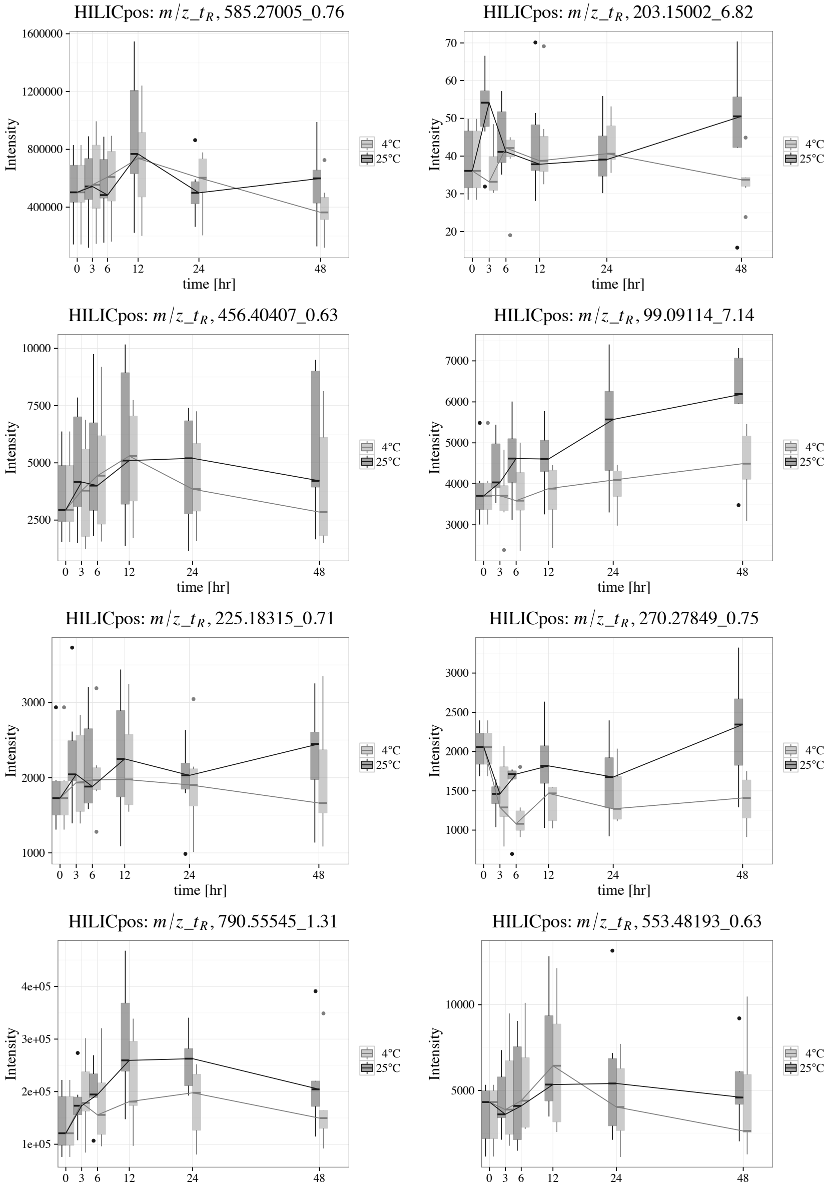
HILICpos: m/z_t_R, 585.27005_0.76
HILICpos: m/z_t_R, 203.15002_6.82
HILICpos: m/z_t_R, 456.40407_0.63
HILICpos: m/z_t_R, 99.09114_7.14
HILICpos: m/z_t_R, 225.18315_0.71
HILICpos: m/z_t_R, 270.27849_0.75
HILICpos: m/z_t_R, 790.55545_1.31
HILICpos: m/z_t_R, 553.48193_0.63
Intensity
time [hr]
4°C
25°C

time [hr]

time [hr]

HILICpos: m/z_t_R, 148.11504_7.11

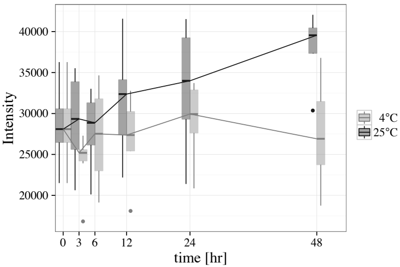

HILICpos: m/z_t_R, 129.10229_7.10

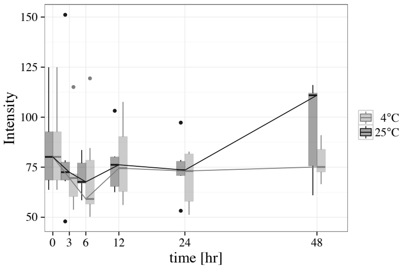

HILICpos: m/z_t_R, 133.10410_3.93

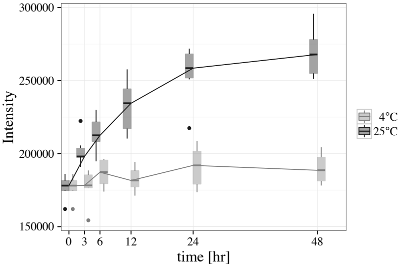

HILICpos: m/z_t_R, 166.08358_5.17

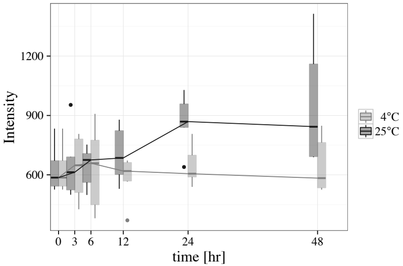

HILICpos: m/z_t_R, 594.50762_0.69

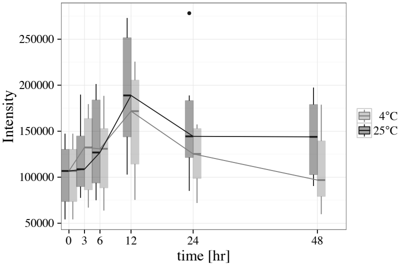

HILICpos: m/z_t_R, 507.44007_0.62

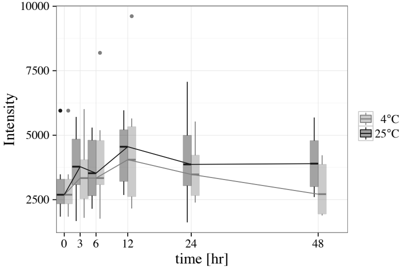

HILICpos: m/z_t_R, 172.11623_3.70

HILICpos: m/z_t_R, 526.40960_0.93

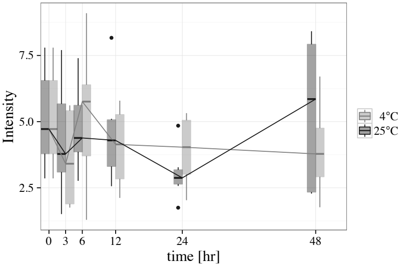

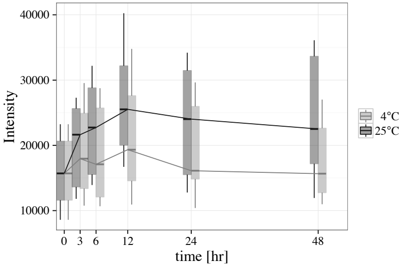

HILICpos: m/z_t_R, 512.33373_3.07

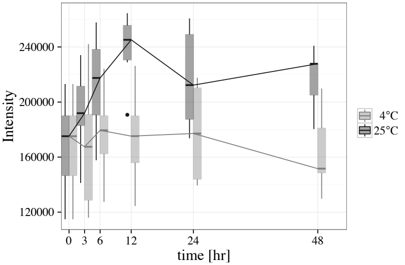

HILICpos: m/z_t_R, 298.18738_6.53

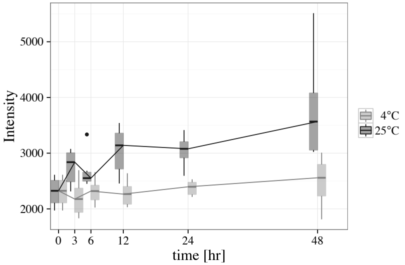

HILICpos: m/z_t_R, 86.09626_3.95

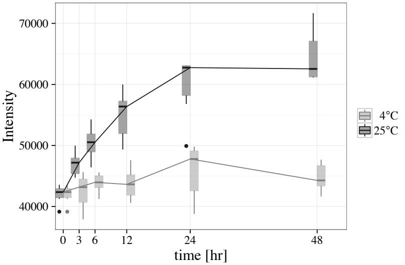

HILICpos: m/z_t_R, 279.23131_0.78

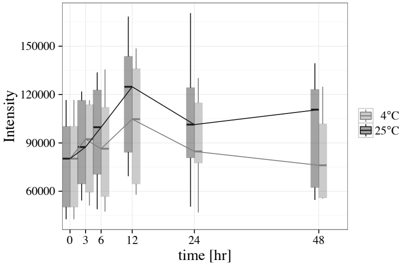

HILICpos: m/z_t_R, 834.59865_0.98

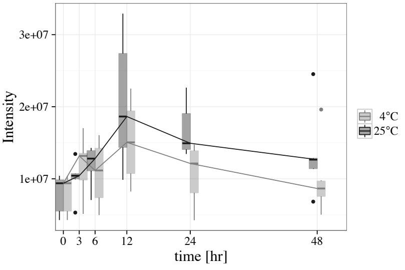

HILICpos: m/z_t_R, 312.32593_0.71

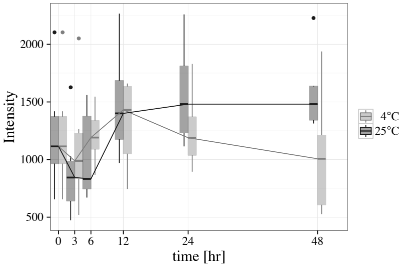

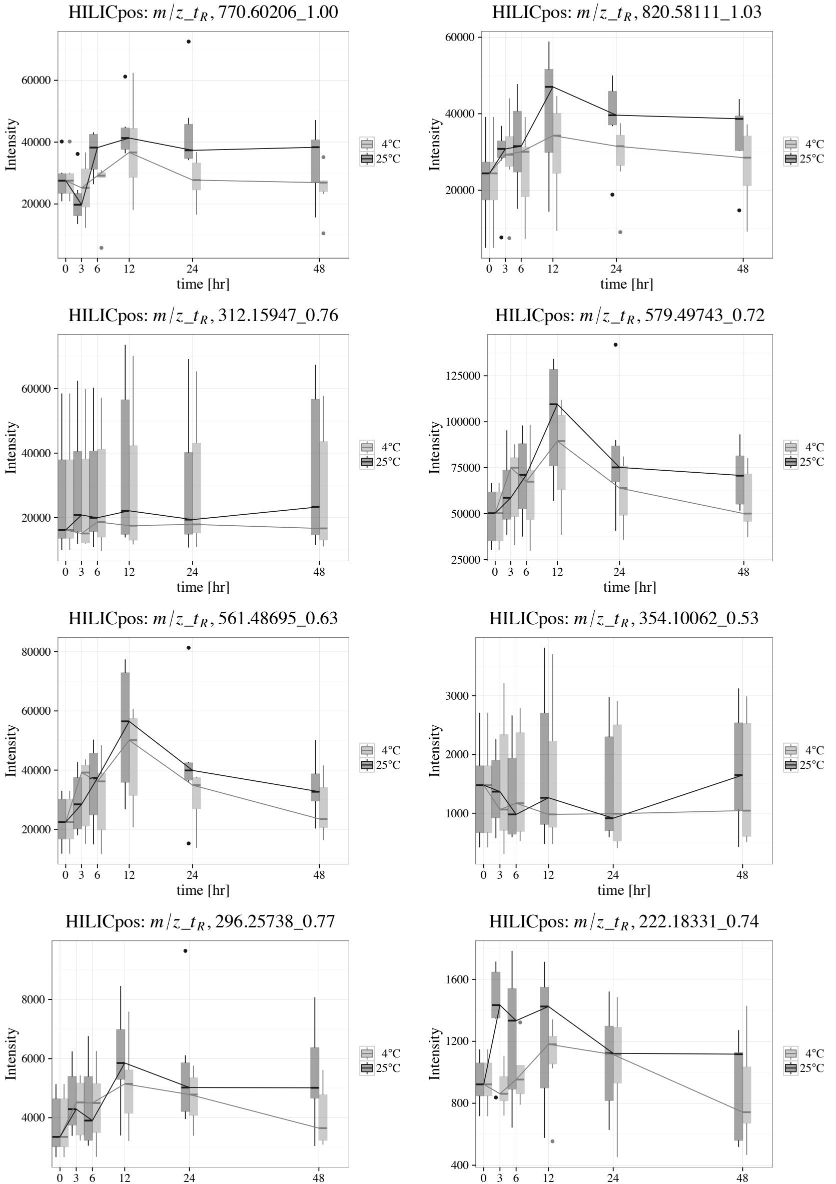

HILICpos: m/z_tR, 770.60206_1.00
HILICpos: m/z_tR, 820.58111_1.03
HILICpos: m/z_tR, 312.15947_0.76
HILICpos: m/z_tR, 579.49743_0.72
HILICpos: m/z_tR, 561.48695_0.63
HILICpos: m/z_tR, 354.10062_0.53
HILICpos: m/z_tR, 296.25738_0.77
HILICpos: m/z_tR, 222.18331_0.74
Intensity
time [hr]
0 3 6 12 24 48
4°C
25°C

time [hr]

time [hr]

HILICpos: m/z_t_R, 276.14392_4.50

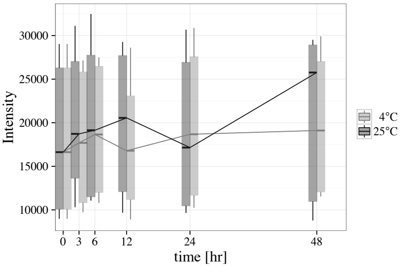

HILICpos: m/z_t_R, 540.46197_0.62

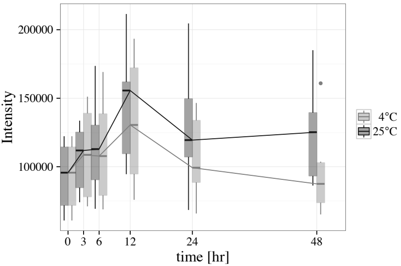

HILICpos: m/z_t_R, 243.16929_1.39

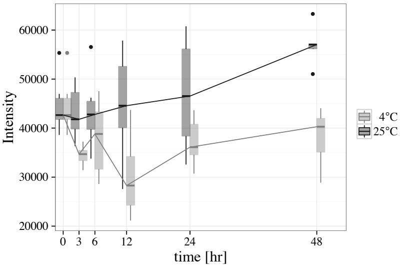

HILICpos: m/z_t_R, 107.05244_5.04

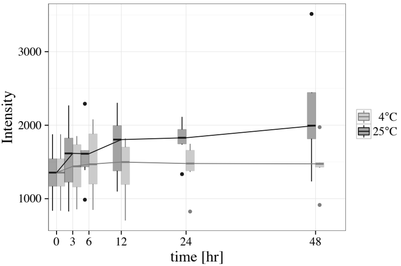

HILICpos: m/z_t_R, 549.44976_0.62

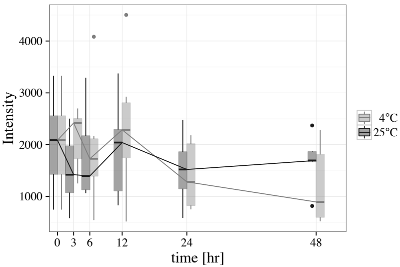

HILICpos: m/z_t_R, 151.06099_3.20

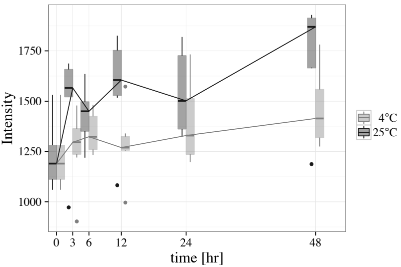

HILICpos: m/z_t_R, 172.11586_3.71

HILICpos: m/z_t_R, 245.14929_3.65

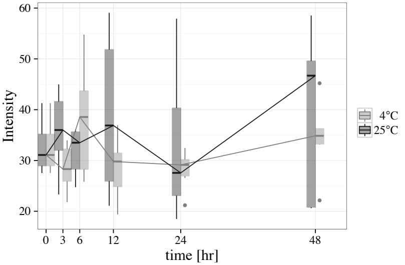

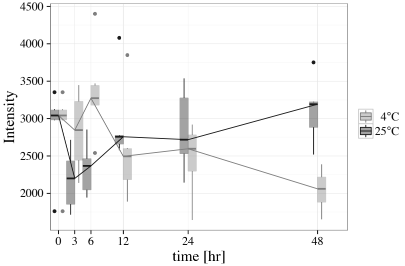

HILICpos: m/z_t_R, 286.27418_0.72

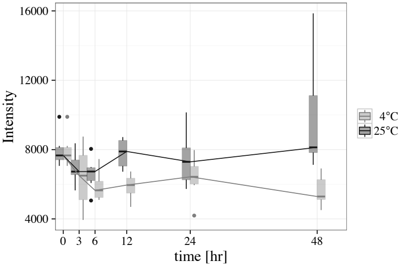

HILICpos: m/z_t_R, 792.58404_1.26

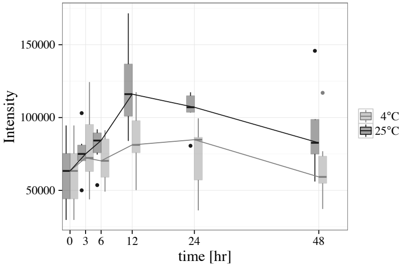

HILICpos: m/z_t_R, 470.41934_0.62

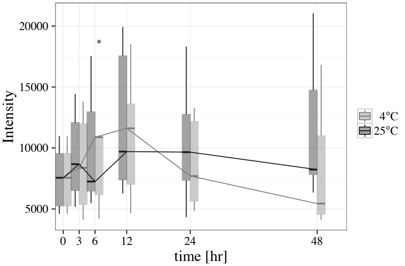

HILICpos: m/z_t_R, 265.21608_0.61

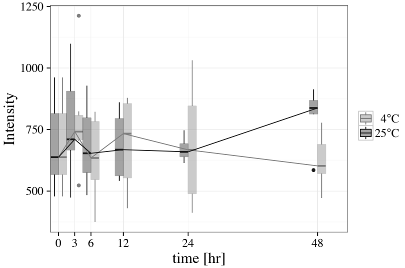

HILICpos: m/z_t_R, 349.05515_4.83

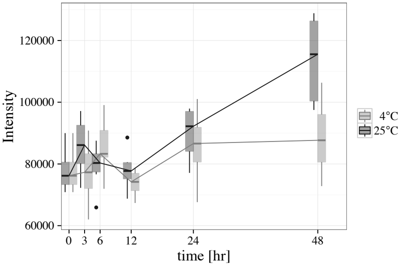

HILICpos: m/z_t_R, 536.43012_0.79

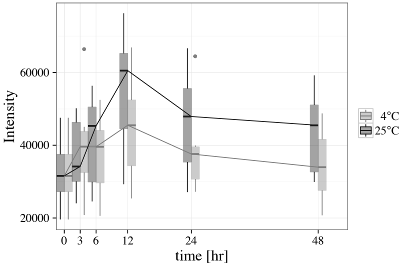

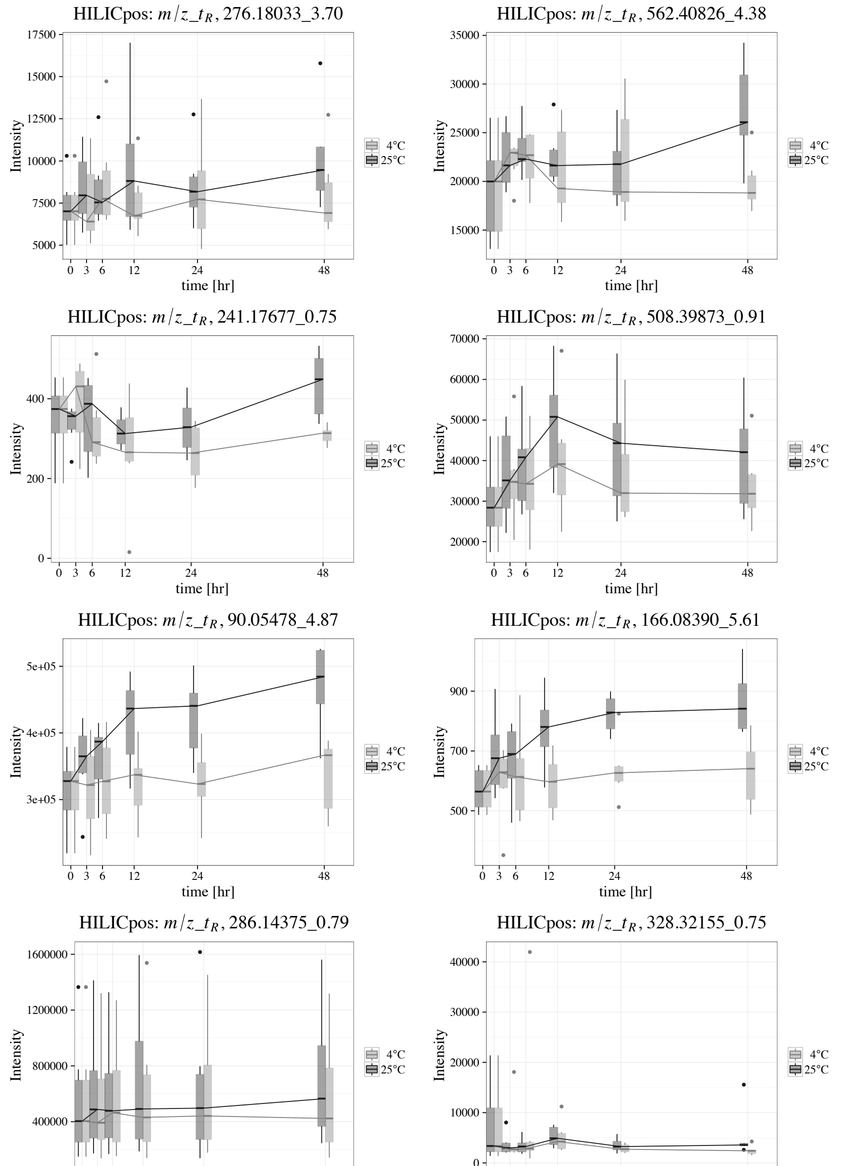
HILICpos: m/z_t_R, 276.18033_3.70
HILICpos: m/z_t_R, 562.40826_4.38
HILICpos: m/z_t_R, 241.17677_0.75
HILICpos: m/z_t_R, 508.39873_0.91
HILICpos: m/z_t_R, 90.05478_4.87
HILICpos: m/z_t_R, 166.08390_5.61
HILICpos: m/z_t_R, 286.14375_0.79
HILICpos: m/z_t_R, 328.32155_0.75
Intensity
time [hr]
4°C
25°C

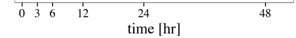

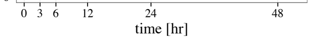

HILICpos: m/z_t_R, 747.52020_0.67

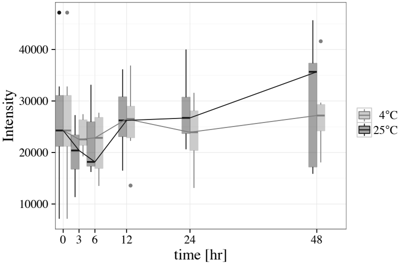

HILICpos: m/z_t_R, 166.08430_5.71

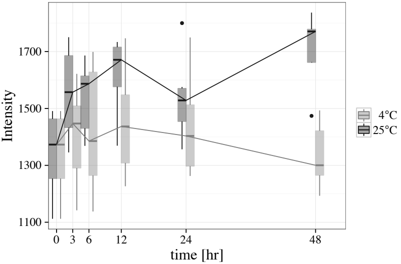

HILICpos: m/z_t_R, 280.17475_0.80

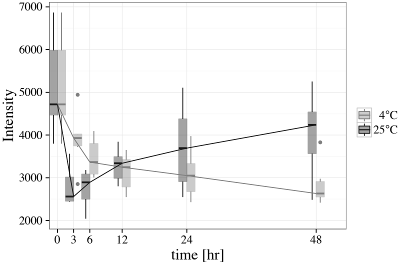

HILICpos: m/z_t_R, 174.11190_4.19

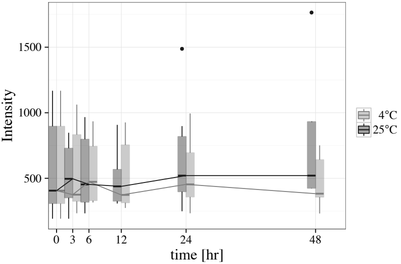

HILICpos: m/z_t_R, 809.64880_1.37

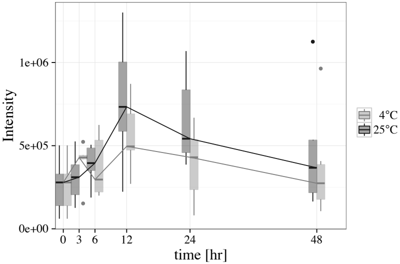

HILICpos: m/z_t_R, 520.33893_2.94

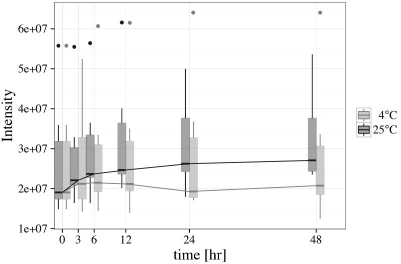

HILICpos: m/z_t_R, 147.11268_8.71

HILICpos: m/z_t_R, 454.29170_3.01

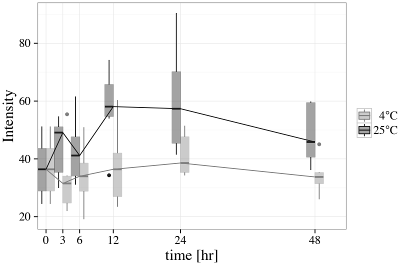

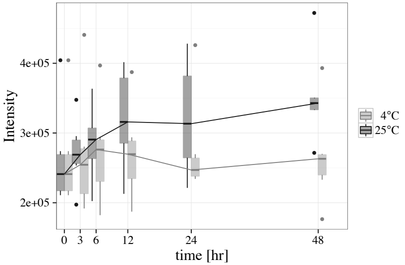

HILICpos: m/z_t_R, 299.13836_0.74

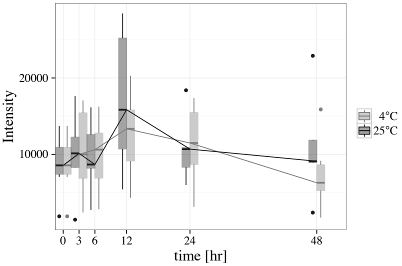

HILICpos: m/z_t_R, 175.14391_6.27

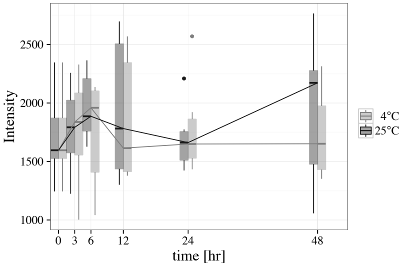

HILICpos: m/z_t_R, 184.05823_0.74

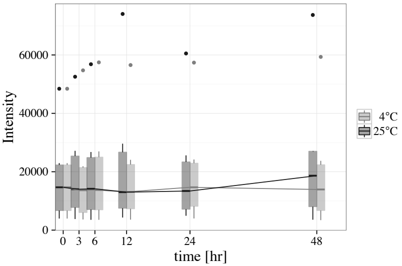

HILICpos: m/z_t_R, 244.15316_3.47

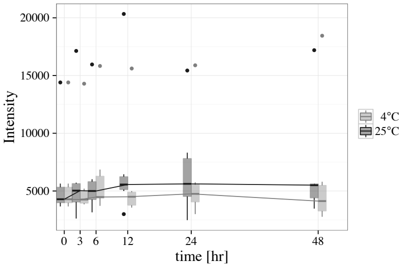

HILICpos: m/z_t_R, 243.16912_3.14

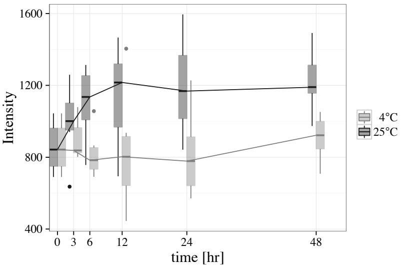

HILICpos: m/z_t_R, 744.58773_1.49

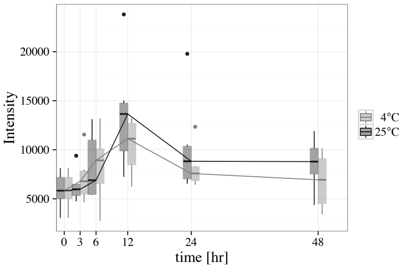

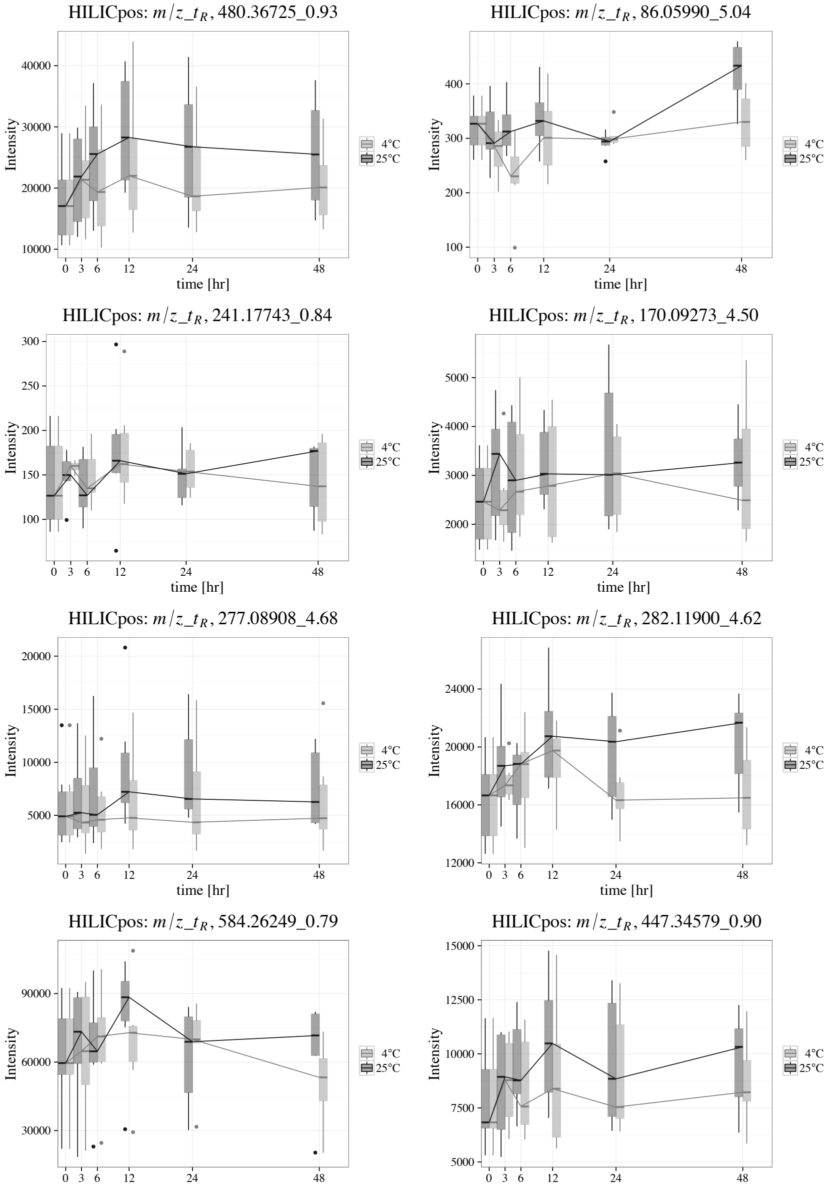

HILICpos: m/z_tR, 480.36725_0.93
HILICpos: m/z_tR, 86.05990_5.04
HILICpos: m/z_tR, 241.17743_0.84
HILICpos: m/z_tR, 170.09273_4.50
HILICpos: m/z_tR, 277.08908_4.68
HILICpos: m/z_tR, 282.11900_4.62
HILICpos: m/z_tR, 584.26249_0.79
HILICpos: m/z_tR, 447.34579_0.90
Intensity
time [hr]
0
3
6
12
24
48
4°C
25°C

time [hr]

HILICpos: m/z_t_R, 372.30997_2.91

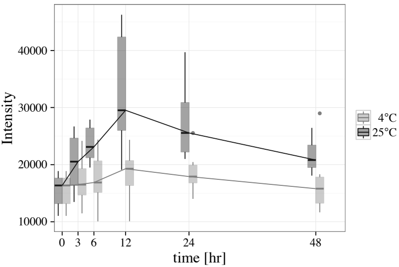

HILICpos: m/z_t_R, 818.59202_0.97

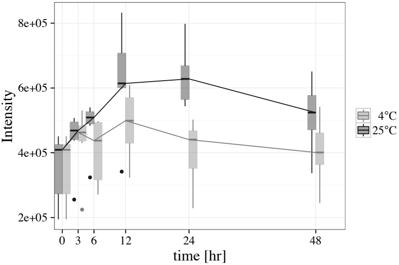

HILICpos: m/z_t_R, 221.09206_3.77

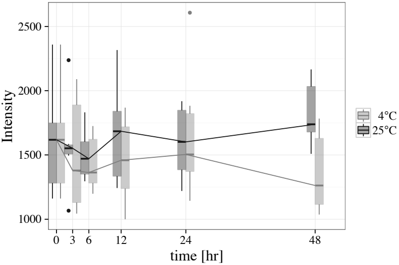

HILICpos: m/z_t_R, 792.58715_0.99

time [hr]

HILICpos: m/z_t_R, 482.38303_0.91

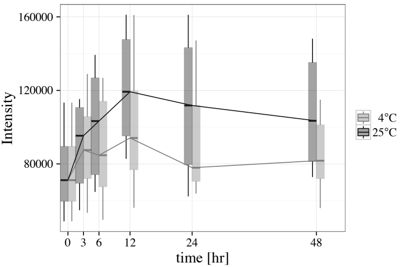

HILICpos: m/z_t_R, 458.38310_0.84

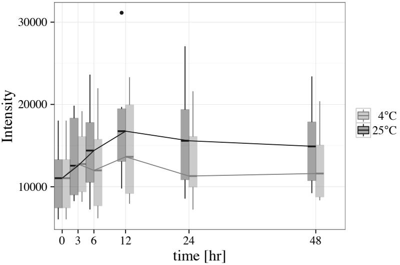

HILICpos: m/z_t_R, 243.19461_0.86

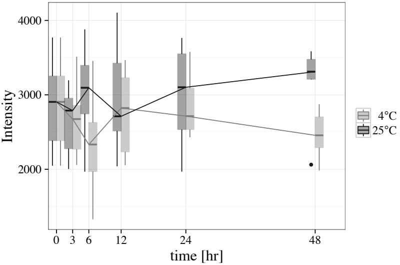

HILICpos: m/z_t_R, 731.60358_1.39

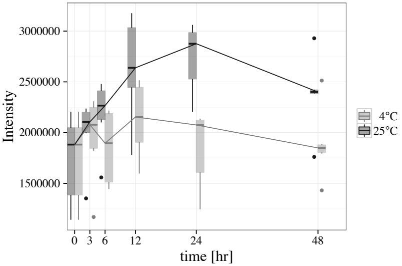

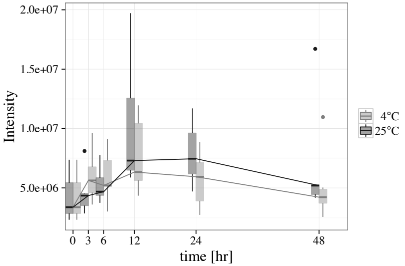

HILICpos: m/z_t_R, 832.58044_0.98

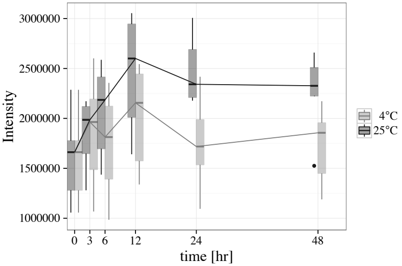

HILICpos: m/z_t_R, 166.08448_5.82

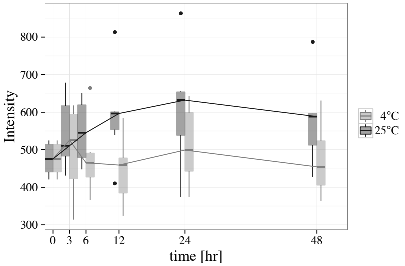

HILICpos: m/z_t_R, 358.20033_7.10

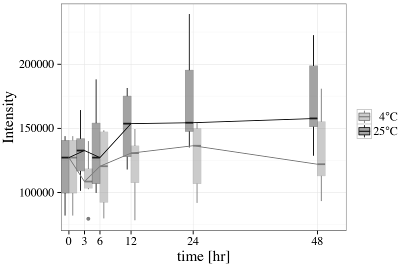

HILICpos: m/z_t_R, 228.26769_3.66

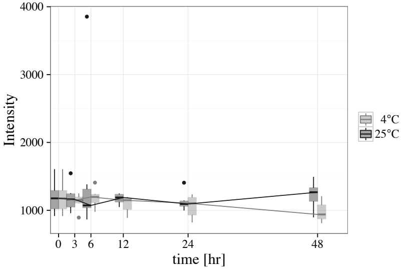

HILICpos: m/z_t_R, 262.12853_4.18

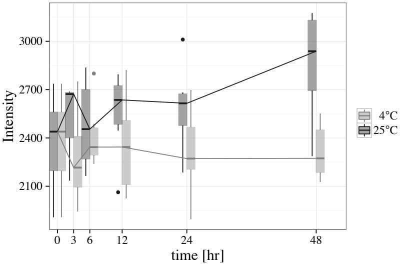

HILICpos: m/z_t_R, 130.06810_4.90

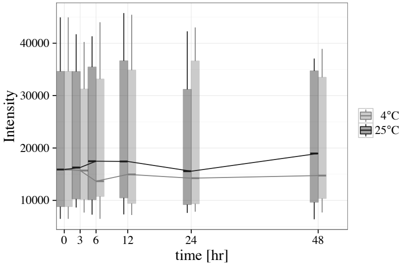

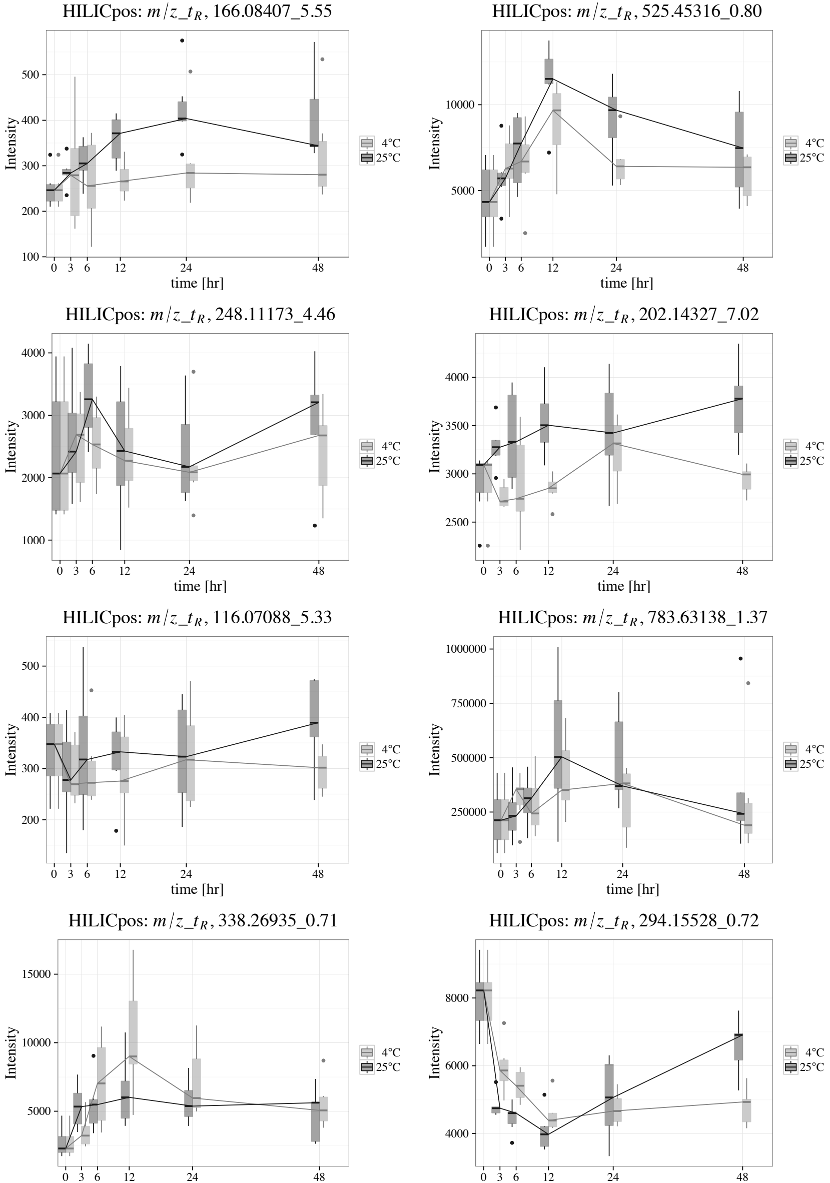
HILICpos: m/z_tR, 166.08407_5.55
HILICpos: m/z_tR, 525.45316_0.80
HILICpos: m/z_tR, 248.11173_4.46
HILICpos: m/z_tR, 202.14327_7.02
HILICpos: m/z_tR, 116.07088_5.33
HILICpos: m/z_tR, 783.63138_1.37
HILICpos: m/z_tR, 338.26935_0.71
HILICpos: m/z_tR, 294.15528_0.72
Intensity
time [hr]
0
3
6
12
24
48
4°C
25°C

time [hr]

time [hr]

HILICpos: m/z_t_R, 209.08926_3.85

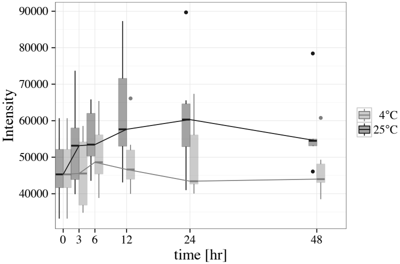

HILICpos: m/z_t_R, 241.02975_5.06

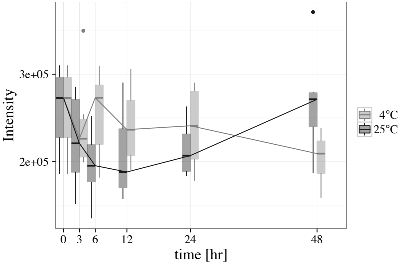

HILICpos: m/z_t_R, 182.07874_4.32

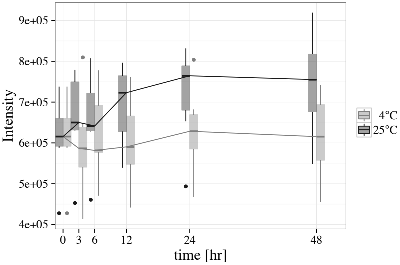

HILICpos: m/z_t_R, 316.28334_0.91

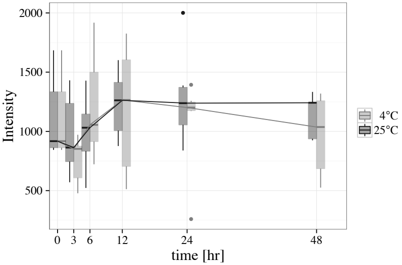

HILICpos: m/z_t_R, 170.09338_4.73

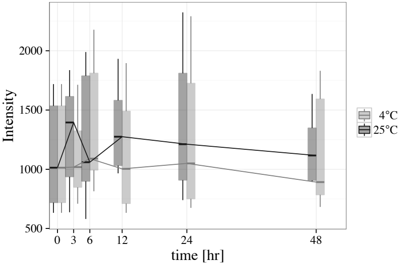

HILICpos: m/z_t_R, 330.22747_3.27

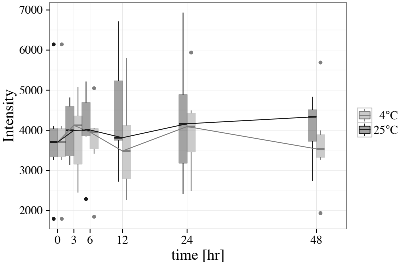

HILICpos: m/z_t_R, 166.08409_7.01

HILICpos: m/z_t_R, 129.06607_5.43

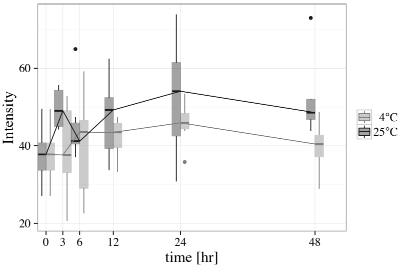

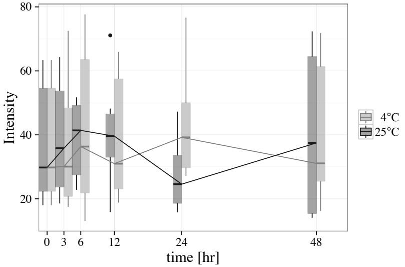

HILICpos: m/z_t_R, 731.60373_2.77

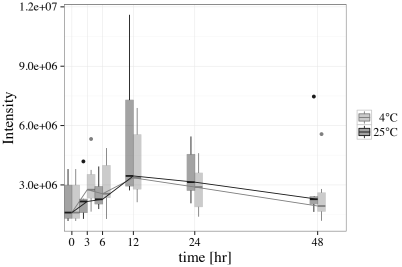

HILICpos: m/z_t_R, 157.05896_3.92

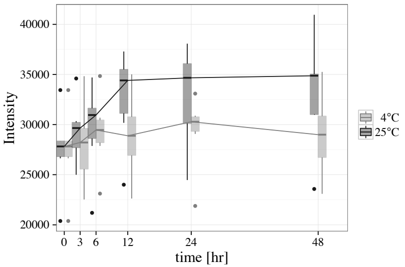

HILICpos: m/z_t_R, 106.08632_5.33

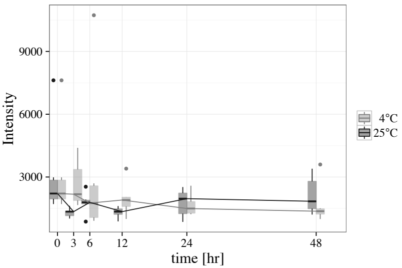

HILICpos: m/z_t_R, 182.08086_4.31

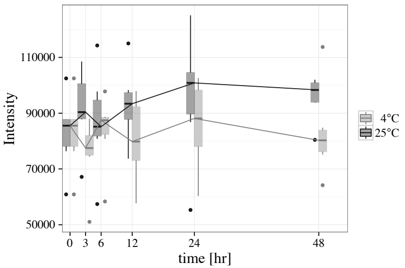

HILICpos: m/z_t_R, 147.11256_6.33

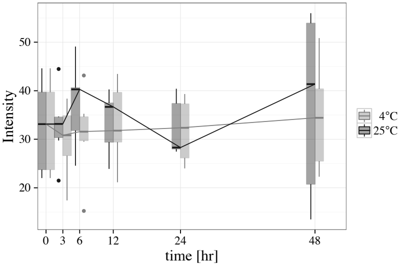

HILICpos: m/z_t_R, 298.23667_0.85

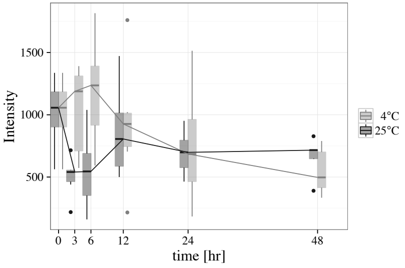

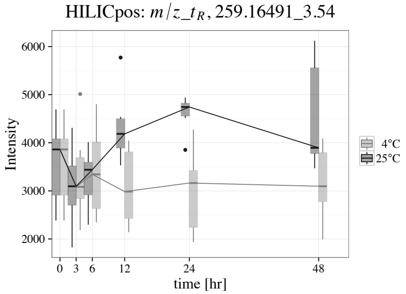
HILICpos: m/z_t_R, 259.16491_3.54
Intensity
2000
3000
4000
5000
6000
time [hr]
0
3
6
12
24
48
4°C
25°C

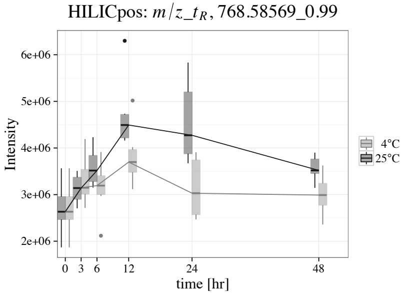
HILICpos: m/z_t_R, 768.58569_0.99
Intensity
2e+06
3e+06
4e+06
5e+06
6e+06
time [hr]
0
3
6
12
24
48
4°C
25°C

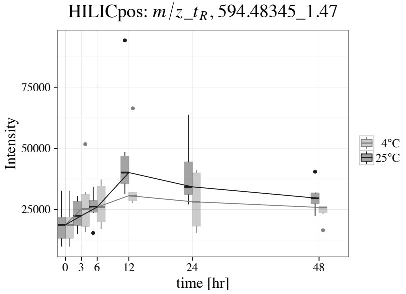
HILICpos: m/z_t_R, 594.48345_1.47
Intensity
25000
50000
75000
time [hr]
0
3
6
12
24
48
4°C
25°C

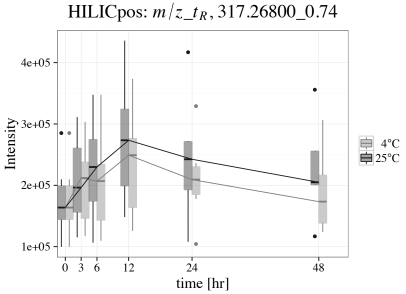
HILICpos: m/z_t_R, 317.26800_0.74
Intensity
1e+05
2e+05
3e+05
4e+05
time [hr]
0
3
6
12
24
48
4°C
25°C

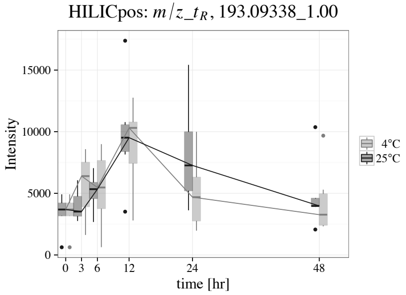
HILICpos: m/z_t_R, 193.09338_1.00
Intensity
0
5000
10000
15000
time [hr]
0
3
6
12
24
48
4°C
25°C

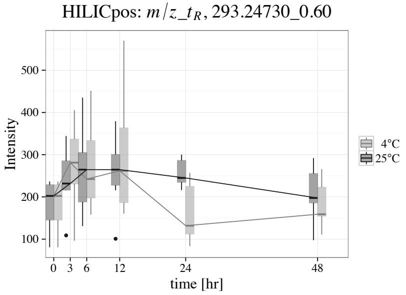
HILICpos: m/z_t_R, 293.24730_0.60
Intensity
100
200
300
400
500
time [hr]
0
3
6
12
24
48
4°C
25°C

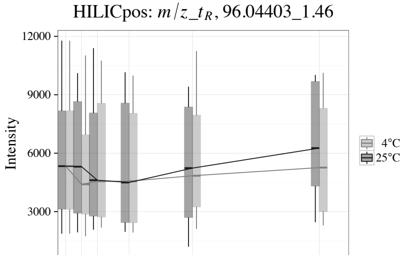
HILICpos: m/z_t_R, 96.04403_1.46
Intensity
3000
6000
9000
12000
4°C
25°C

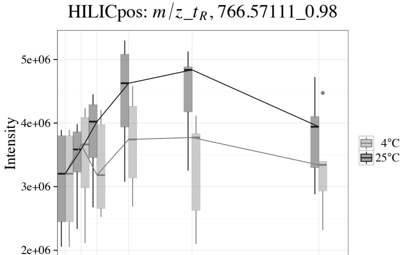
HILICpos: m/z_t_R, 766.57111_0.98
Intensity
2e+06
3e+06
4e+06
5e+06
4°C
25°C

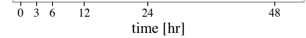

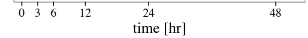

HILICpos: m/z_t_R, 166.08422_5.98

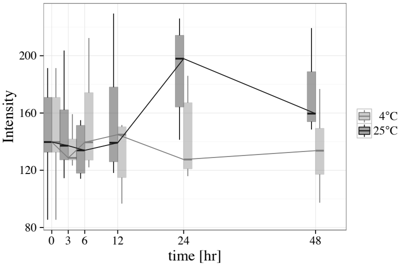

HILICpos: m/z_t_R, 70.06513_4.36

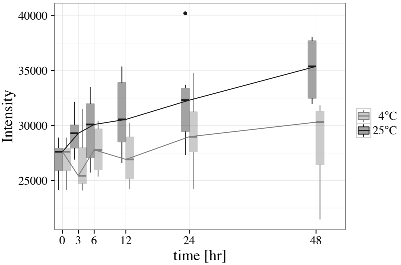

HILICpos: m/z_t_R, 261.20571_0.86

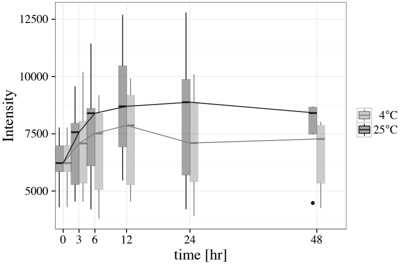

HILICpos: m/z_t_R, 204.13568_0.79

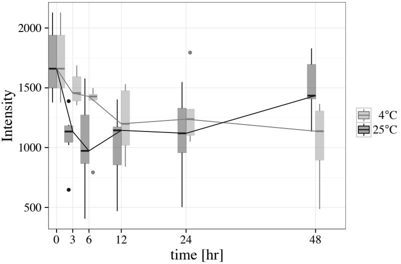

HILICpos: m/z_t_R, 276.15487_5.63

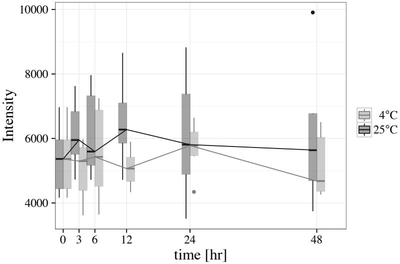

HILICpos: m/z_t_R, 223.16645_0.81

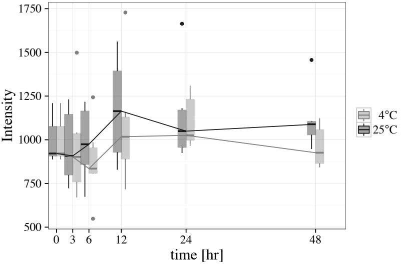

HILICpos: m/z_t_R, 318.22739_3.31

HILICpos: m/z_t_R, 85.02794_4.04

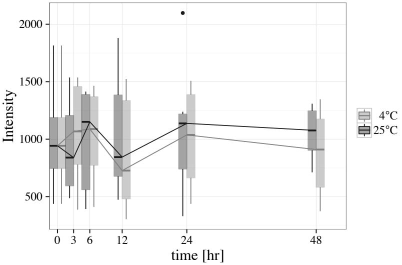

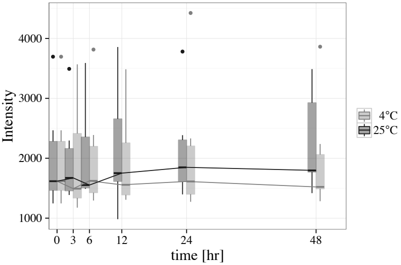

HILICpos: m/z_t_R, 703.57194_1.40

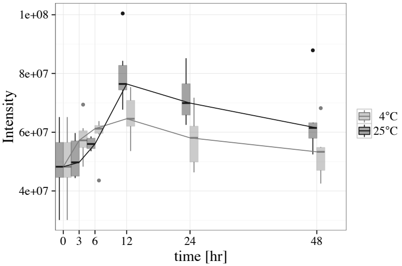

HILICpos: m/z_t_R, 84.08079_7.52

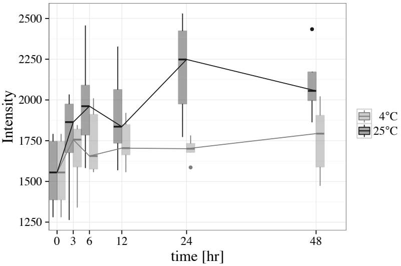

HILICpos: m/z_t_R, 116.10651_5.17

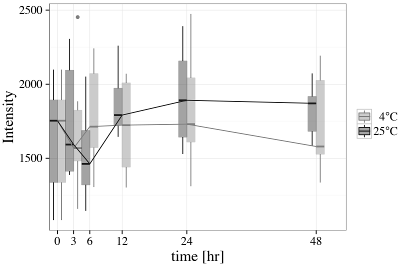

HILICpos: m/z_t_R, 532.43845_0.67

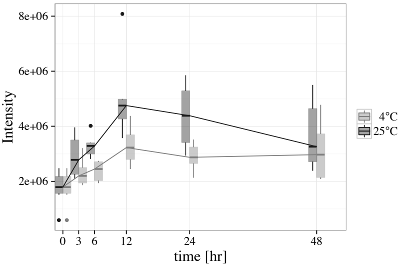

HILICpos: m/z_t_R, 725.55486_1.41

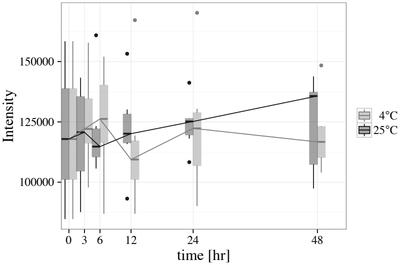

HILICpos: m/z_t_R, 675.54000_1.42

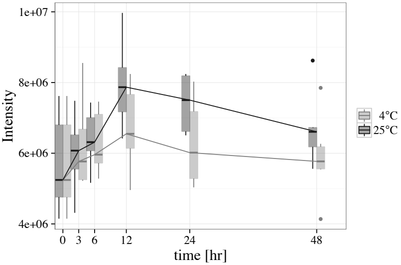

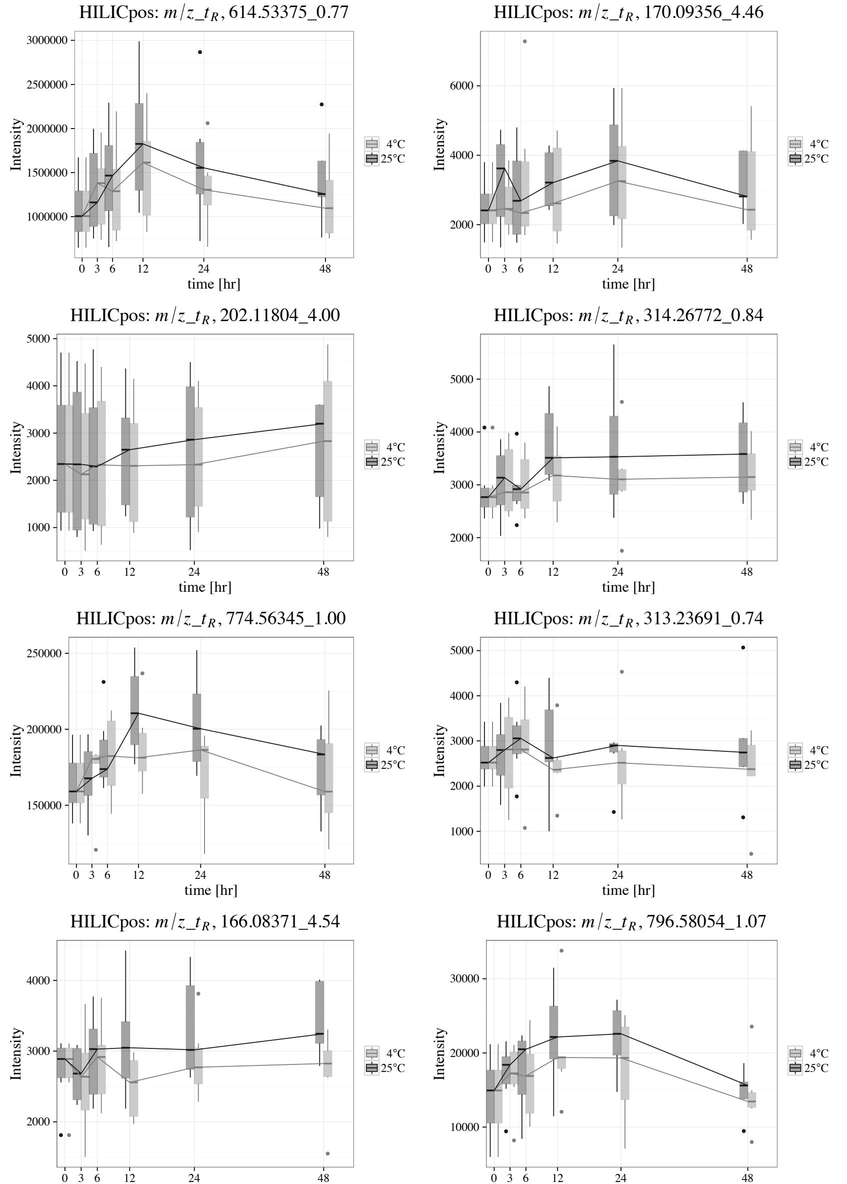
HILICpos: m/z_t_R, 614.53375_0.77
Intensity
3000000
2500000
2000000
1500000
1000000
0 3 6 12 24 48
time [hr]
4°C
25°C
HILICpos: m/z_t_R, 170.09356_4.46
Intensity
6000
4000
2000
0 3 6 12 24 48
time [hr]
4°C
25°C
HILICpos: m/z_t_R, 202.11804_4.00
Intensity
5000
4000
3000
2000
1000
0 3 6 12 24 48
time [hr]
4°C
25°C
HILICpos: m/z_t_R, 314.26772_0.84
Intensity
5000
4000
3000
2000
0 3 6 12 24 48
time [hr]
4°C
25°C
HILICpos: m/z_t_R, 774.56345_1.00
Intensity
250000
200000
150000
0 3 6 12 24 48
time [hr]
4°C
25°C
HILICpos: m/z_t_R, 313.23691_0.74
Intensity
5000
4000
3000
2000
1000
0 3 6 12 24 48
time [hr]
4°C
25°C
HILICpos: m/z_t_R, 166.08371_4.54
Intensity
4000
3000
2000
0 3 6 12 24 48
4°C
25°C
HILICpos: m/z_t_R, 796.58054_1.07
Intensity
30000
20000
10000
0 3 6 12 24 48
4°C
25°C

time [hr]

time [hr]

HILICpos: m/z_t_R, 155.11756_1.04

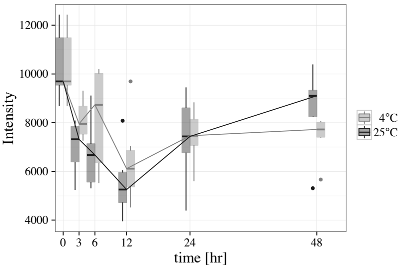

HILICpos: m/z_t_R, 229.08350_4.58

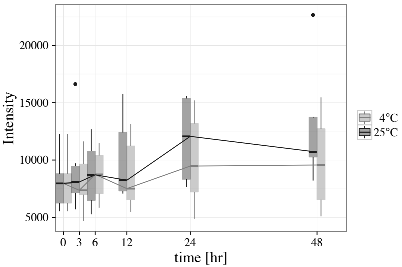

HILICpos: m/z_t_R, 249.18397_0.75

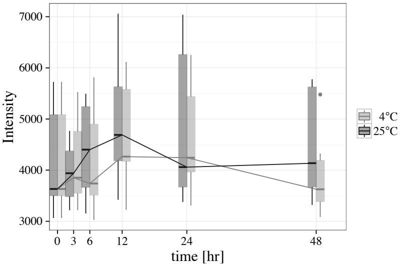

HILICpos: m/z_t_R, 746.60489_1.09

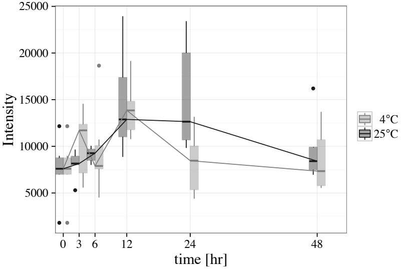

HILICpos: m/z_t_R, 198.12440_0.92

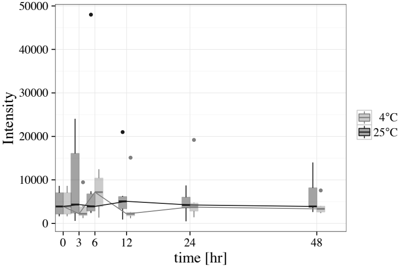

HILICpos: m/z_t_R, 517.38919_0.69

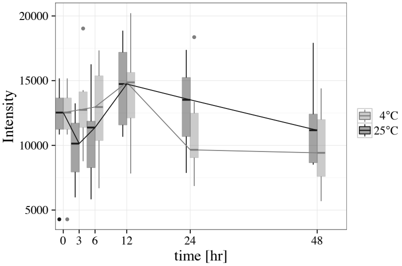

HILICpos: m/z_t_R, 701.55643_1.41

HILICpos: m/z_t_R, 774.59936_0.98

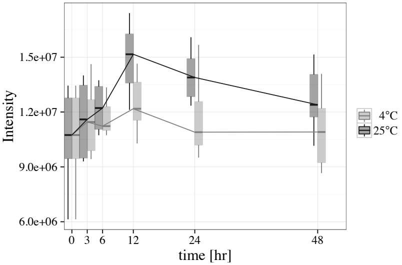

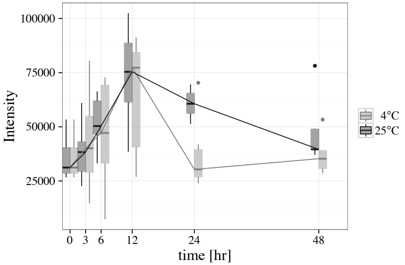

HILICpos: m/z_t_R, 274.16452_3.63

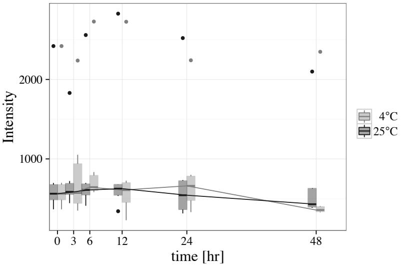

HILICpos: m/z_t_R, 742.57206_0.98

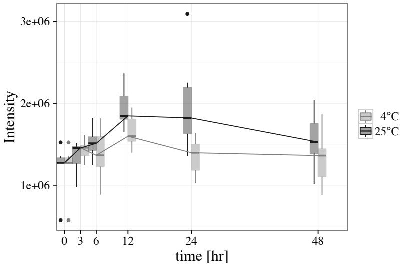

HILICpos: m/z_t_R, 360.27420_3.14

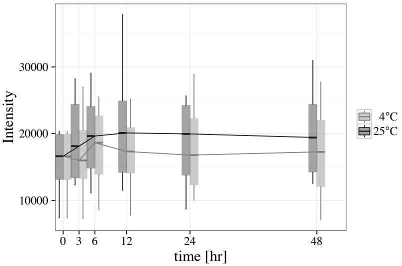

HILICpos: m/z_t_R, 806.56730_0.98

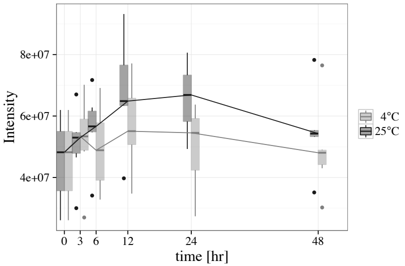

HILICpos: m/z_t_R, 689.55598_1.41

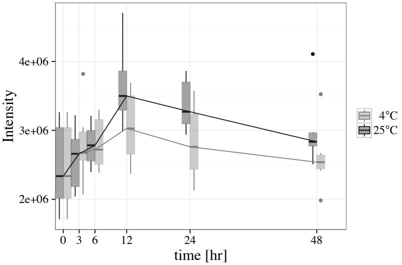

HILICpos: m/z_t_R, 284.12817_0.76

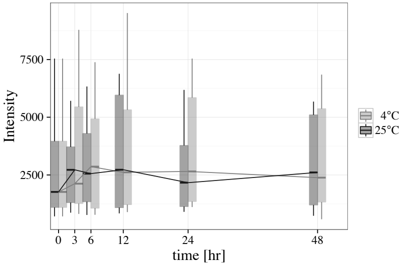

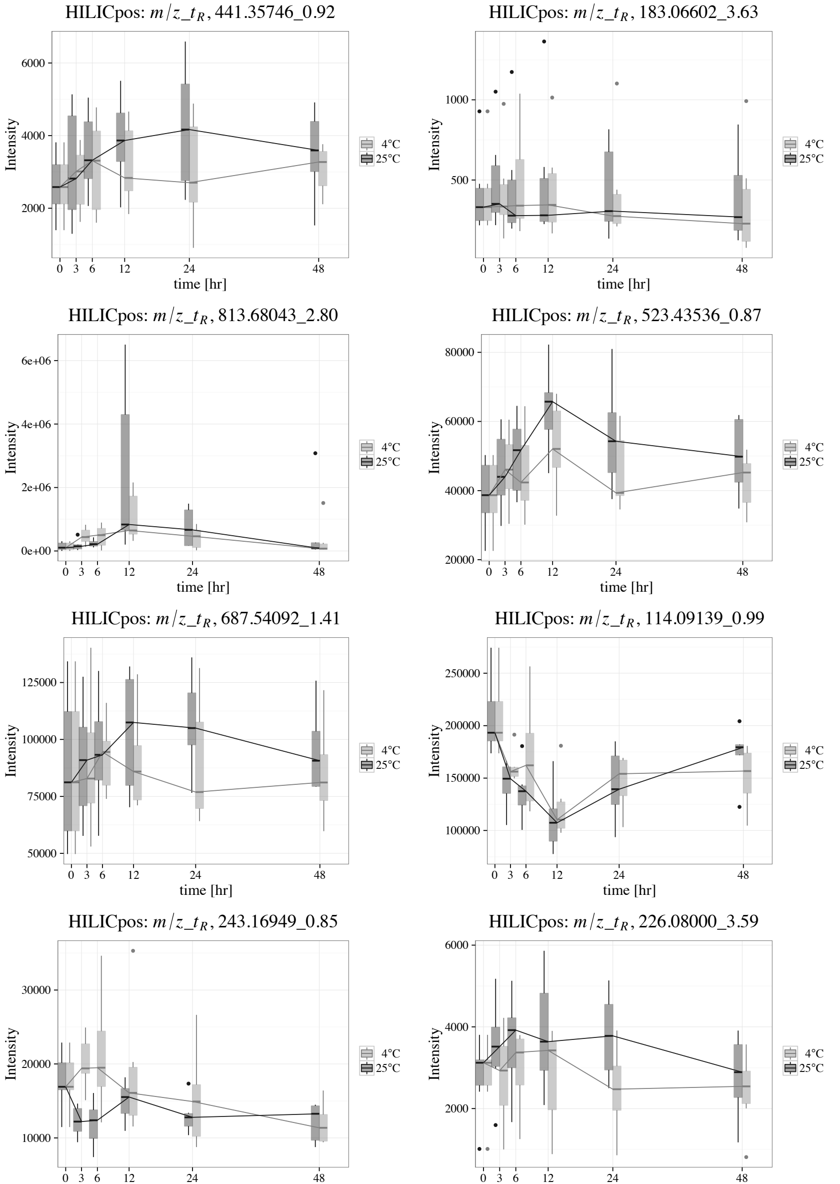

HILICpos: m/z_t_R, 441.35746_0.92
HILICpos: m/z_t_R, 183.06602_3.63
HILICpos: m/z_t_R, 813.68043_2.80
HILICpos: m/z_t_R, 523.43536_0.87
HILICpos: m/z_t_R, 687.54092_1.41
HILICpos: m/z_t_R, 114.09139_0.99
HILICpos: m/z_t_R, 243.16949_0.85
HILICpos: m/z_t_R, 226.08000_3.59
Intensity
time [hr]
0
3
6
12
24
48
4°C
25°C

time [hr] time [hr]

HILICpos: m/z_t_R, 127.12298_5.65

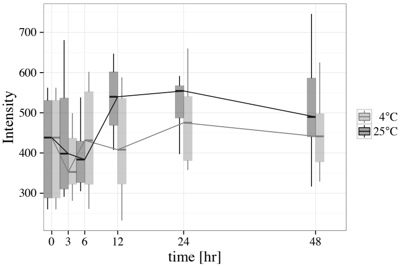

HILICpos: m/z_t_R, 167.10448_0.91

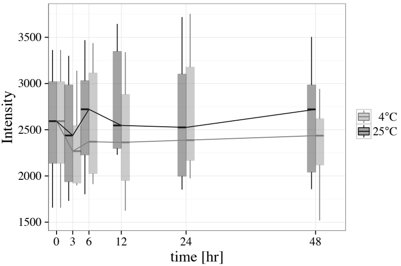

HILICpos: m/z_t_R, 72.08075_4.29

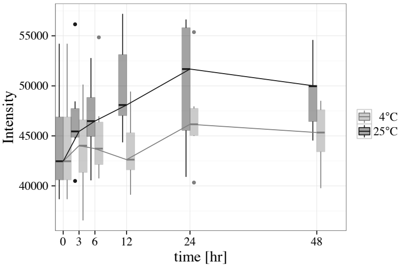

HILICpos: m/z_t_R, 126.10199_1.22

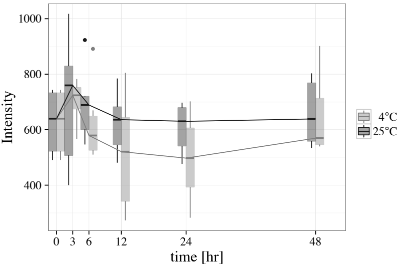

HILICpos: m/z_t_R, 221.15210_0.93

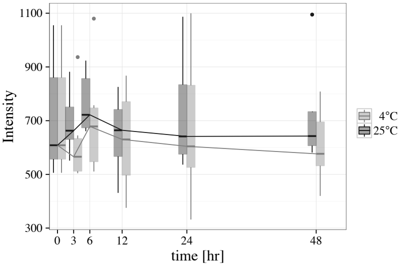

HILICpos: m/z_t_R, 296.20624_0.75

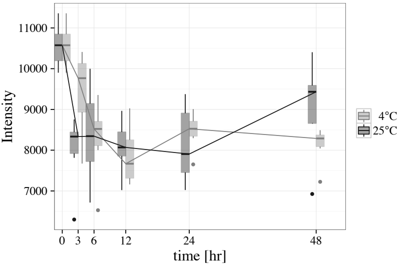

HILICpos: m/z_t_R, 862.61880_1.47 HILICpos: m/z_t_R, 299.25744_0.77

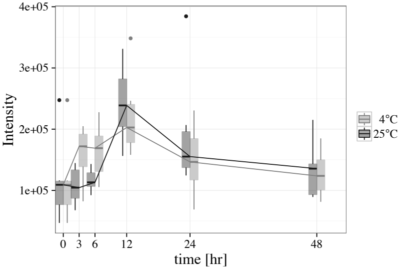

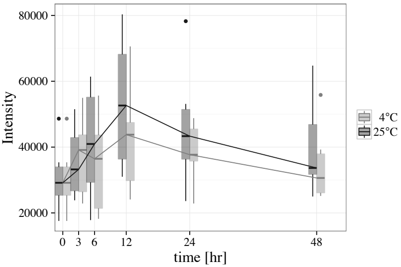

HILICpos: m/z_t_R, 275.21199_0.78

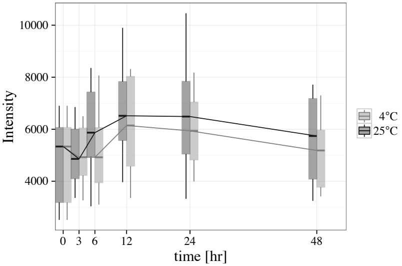

HILICpos: m/z_t_R, 729.58831_1.39

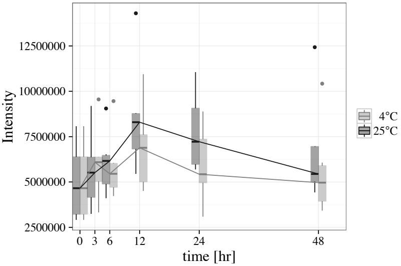

HILICpos: m/z_t_R, 727.57223_1.39

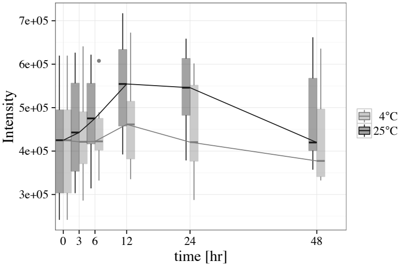

HILICpos: m/z_t_R, 386.28976_3.06

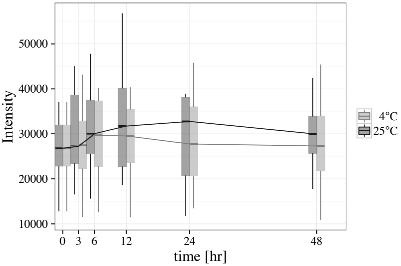

HILICpos: m/z_t_R, 209.15302_0.70

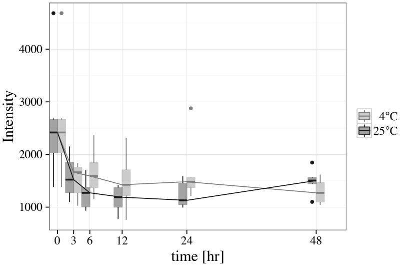

HILICpos: m/z_t_R, 673.52456_1.44

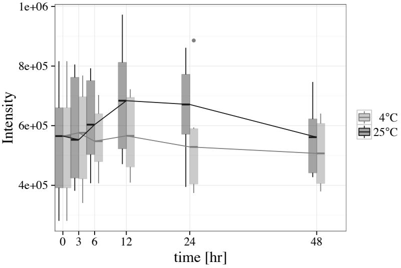

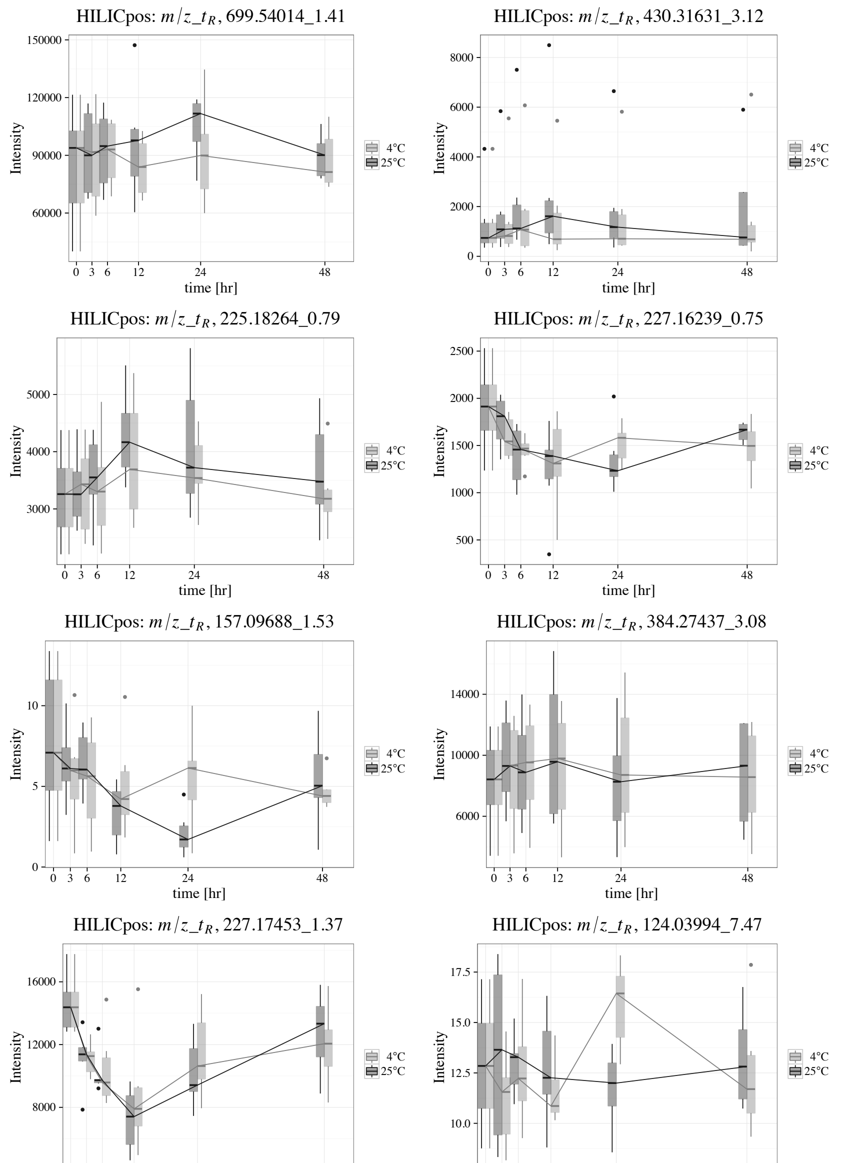

HILICpos: m/z_t_R, 699.54014_1.41
HILICpos: m/z_t_R, 430.31631_3.12
HILICpos: m/z_t_R, 225.18264_0.79
HILICpos: m/z_t_R, 227.16239_0.75
HILICpos: m/z_t_R, 157.09688_1.53
HILICpos: m/z_t_R, 384.27437_3.08
HILICpos: m/z_t_R, 227.17453_1.37
HILICpos: m/z_t_R, 124.03994_7.47
Intensity
time [hr]
4°C
25°C

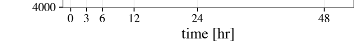

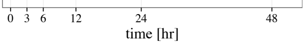

HILICpos: m/z_t_R, 459.38533_0.69

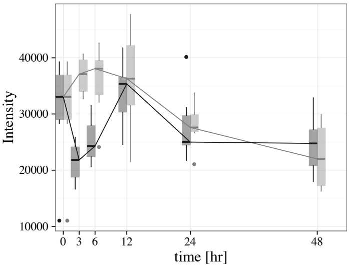

HILICpos: m/z_t_R, 792.58651_1.60

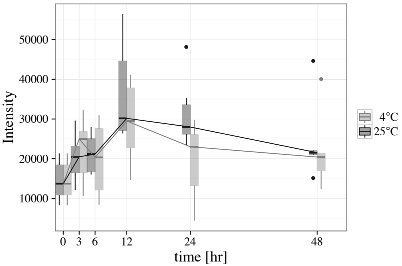

HILICpos: m/z_t_R, 244.18956_0.70

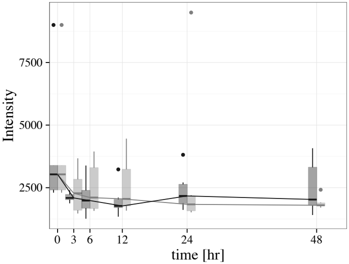

HILICpos: m/z_t_R, 72.08059_5.15

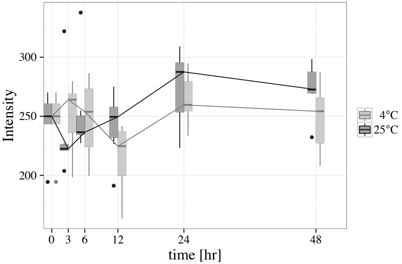

HILICpos: m/z_t_R, 293.21052_0.75

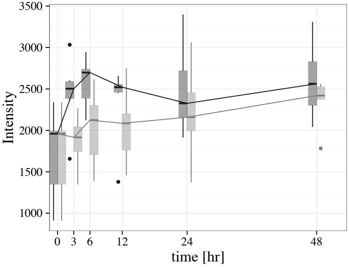

HILICpos: m/z_t_R, 358.25865_3.13

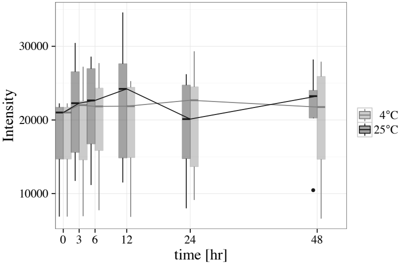

HILICpos: m/z_t_R, 105.07337_4.21

HILICpos: m/z_t_R, 145.10382_3.79

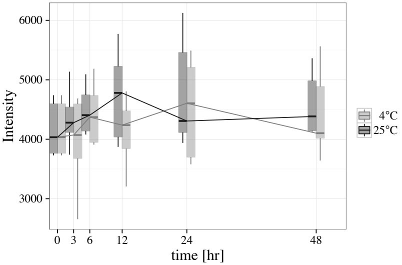

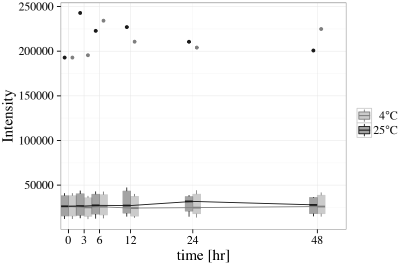

HILICpos: m/z_t_R, 166.08454_6.01

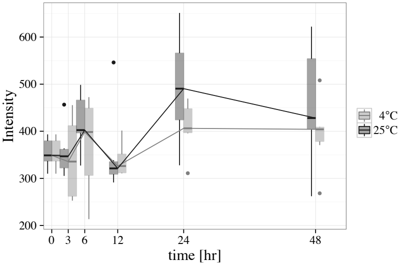

HILICpos: m/z_t_R, 255.15846_0.75

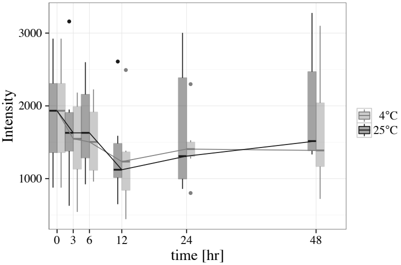

HILICpos: m/z_t_R, 208.15141_0.87

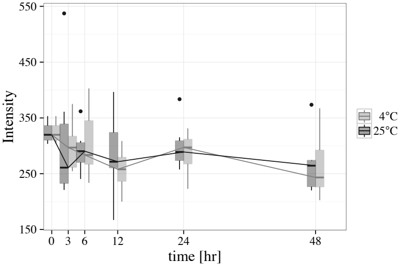

HILICpos: m/z_t_R, 703.57225_2.83

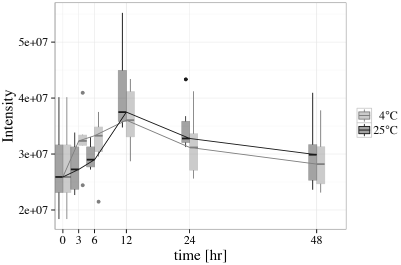

HILICpos: m/z_t_R, 226.16191_0.84

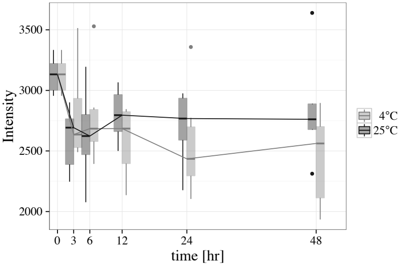

HILICpos: m/z_t_R, 258.18024_3.07

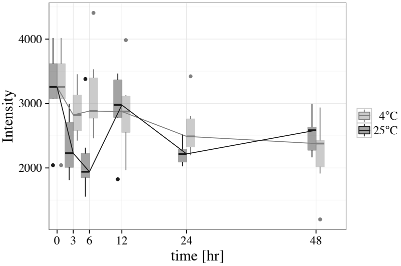

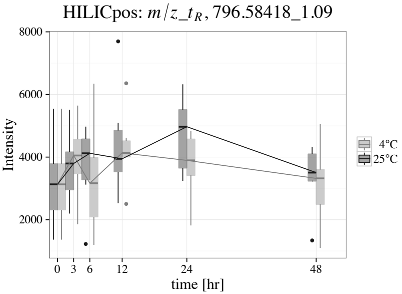
HILICpos: m/z_t_R, 796.58418_1.09
Intensity
time [hr]
4°C
25°C

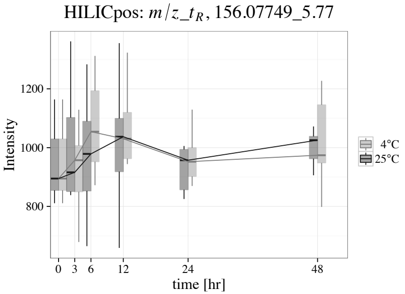
HILICpos: m/z_t_R, 156.07749_5.77
Intensity
time [hr]
4°C
25°C

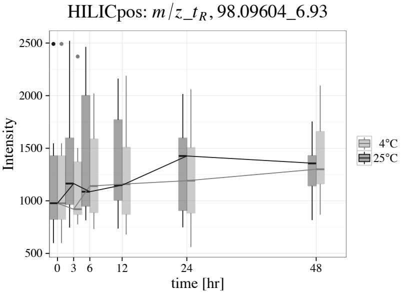
HILICpos: m/z_t_R, 98.09604_6.93
Intensity
time [hr]
4°C
25°C

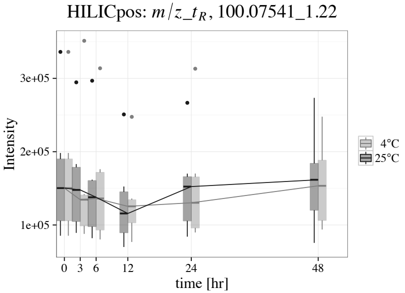
HILICpos: m/z_t_R, 100.07541_1.22
Intensity
time [hr]
4°C
25°C

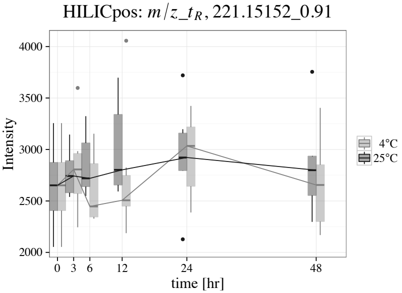
HILICpos: m/z_t_R, 221.15152_0.91
Intensity
time [hr]
4°C
25°C

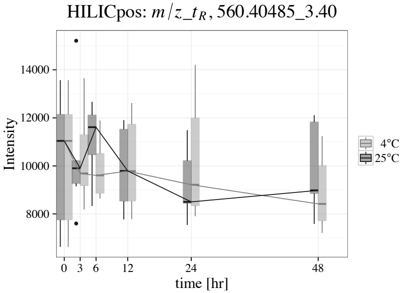
HILICpos: m/z_t_R, 560.40485_3.40
Intensity
time [hr]
4°C
25°C

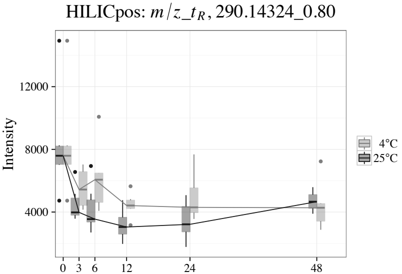
HILICpos: m/z_t_R, 290.14324_0.80
Intensity
4°C
25°C

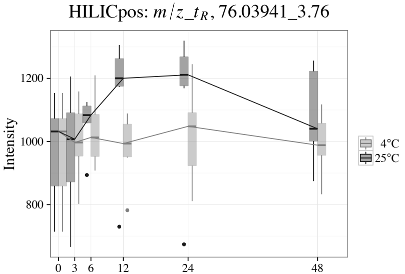
HILICpos: m/z_t_R, 76.03941_3.76
Intensity
4°C
25°C

time [hr]

time [hr]

HILICpos: m/z_t_R, 372.27483_0.69

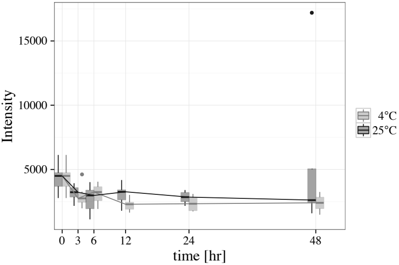

HILICpos: m/z_t_R, 331.04487_4.84

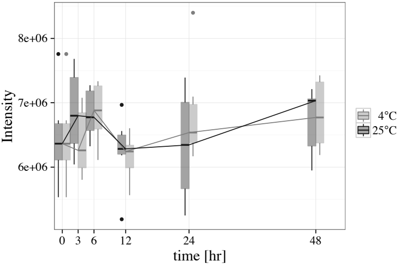

HILICpos: m/z_t_R, 700.56952_0.93

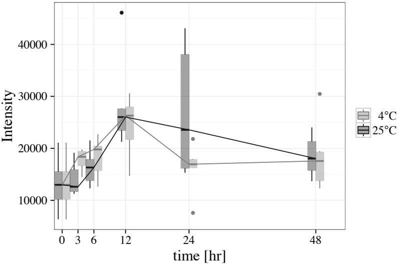

HILICpos: m/z_t_R, 706.58783_1.39

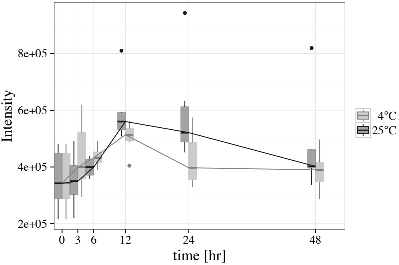

HILICpos: m/z_t_R, 310.20090_3.11

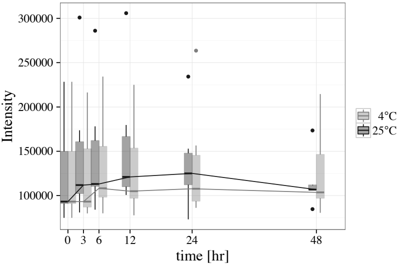

HILICpos: m/z_t_R, 159.14771_3.62

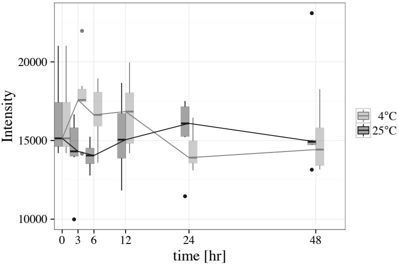

HILICpos: m/z_t_R, 319.16511_3.59

HILICpos: m/z_t_R, 170.09287_4.42

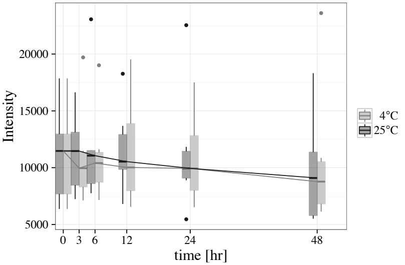

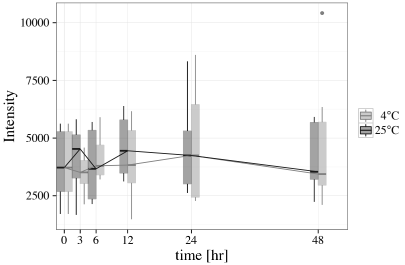

HILICpos: m/z_t_R, 298.22136_0.86

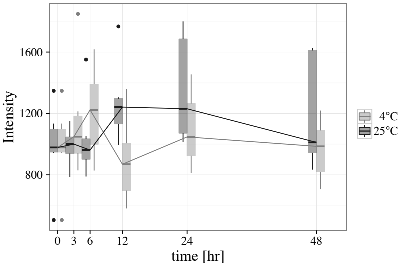

HILICpos: m/z_t_R, 166.07032_3.54

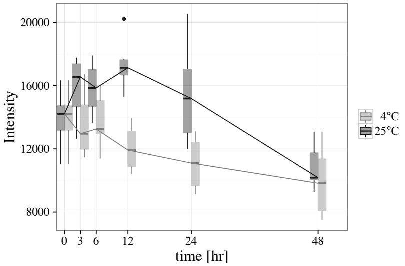

HILICpos: m/z_t_R, 298.11451_3.67

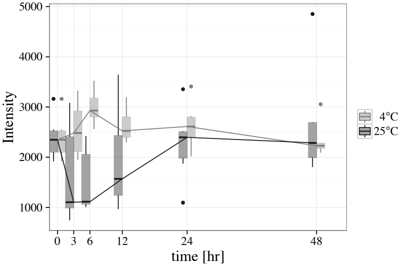

HILICpos: m/z_t_R, 412.20910_3.29

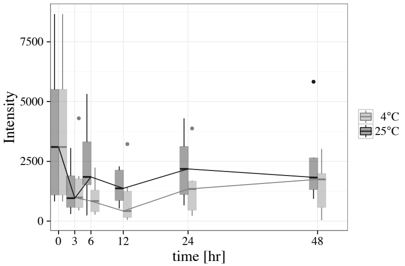

HILICpos: m/z_t_R, 531.40705_0.68

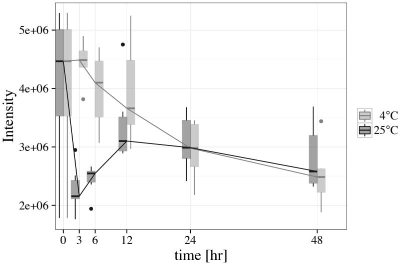

HILICpos: m/z_t_R, 274.20084_3.17

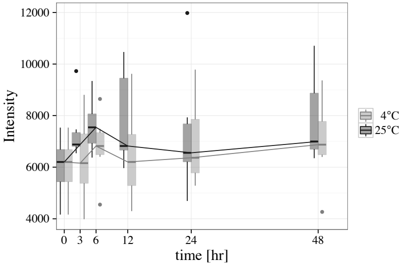

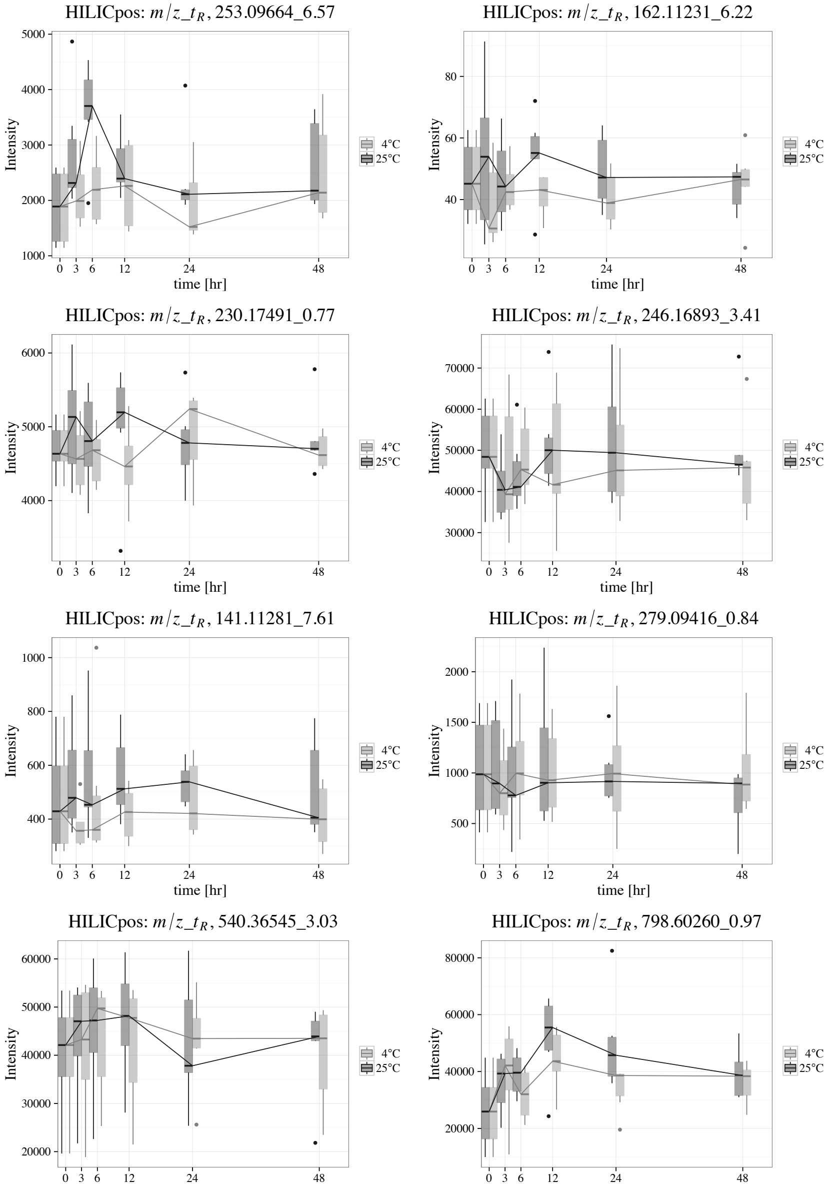
HILICpos: m/z_t_R, 253.09664_6.57
HILICpos: m/z_t_R, 162.11231_6.22
HILICpos: m/z_t_R, 230.17491_0.77
HILICpos: m/z_t_R, 246.16893_3.41
HILICpos: m/z_t_R, 141.11281_7.61
HILICpos: m/z_t_R, 279.09416_0.84
HILICpos: m/z_t_R, 540.36545_3.03
HILICpos: m/z_t_R, 798.60260_0.97
Intensity
time [hr]
0
3
6
12
24
48
4°C
25°C

time [hr]

time [hr]

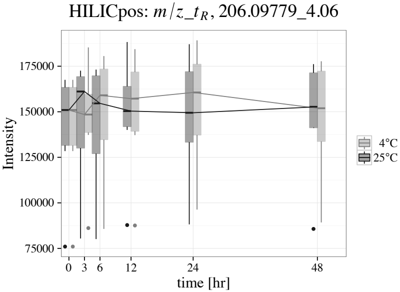

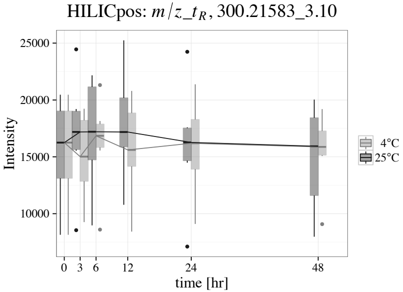

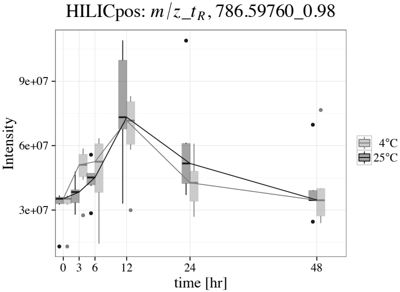

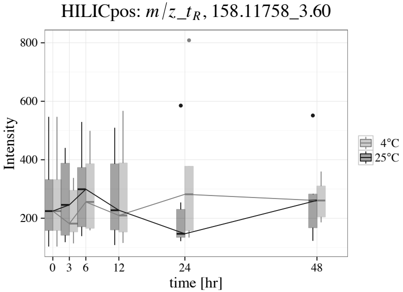

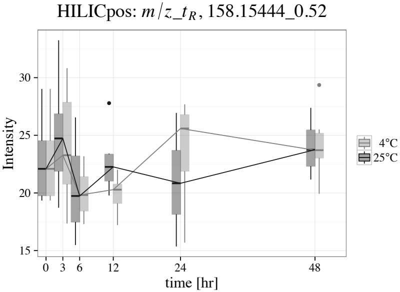

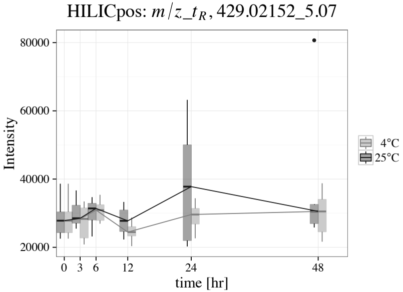

HILICpos: m/z_t_R, 114.06559_3.41

HILICpos: m/z_t_R, 296.14917_3.78

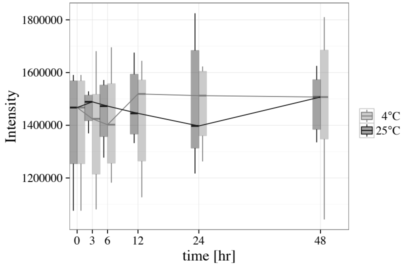

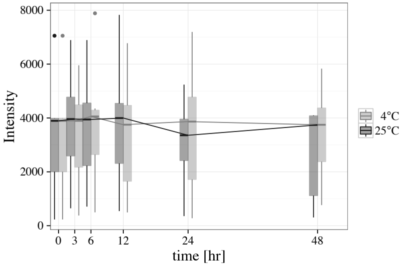

HILICpos: m/z_t_R, 335.03763_4.84

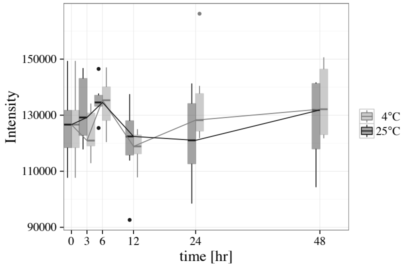

HILICpos: m/z_t_R, 290.15911_4.24

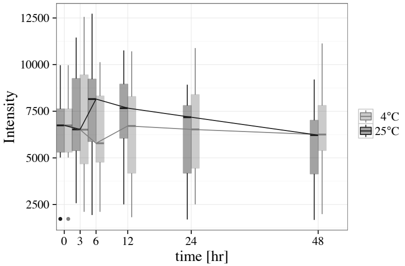

HILICpos: m/z_t_R, 168.07459_3.80

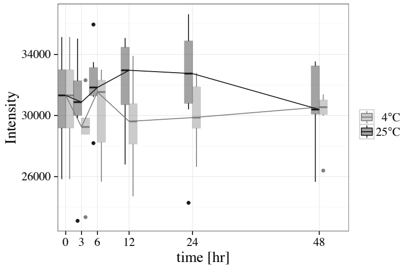

HILICpos: m/z_t_R, 70.06492_7.27

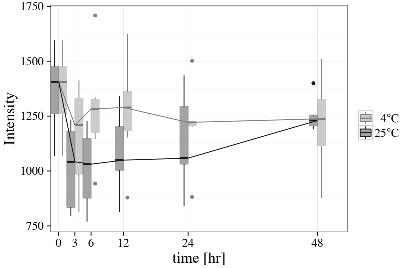

HILICpos: m/z_t_R, 189.13376_8.22

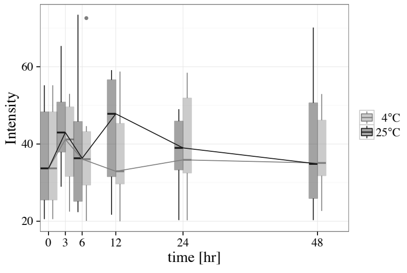

HILICpos: m/z_t_R, 192.12261_3.74

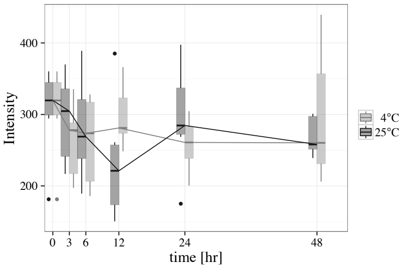

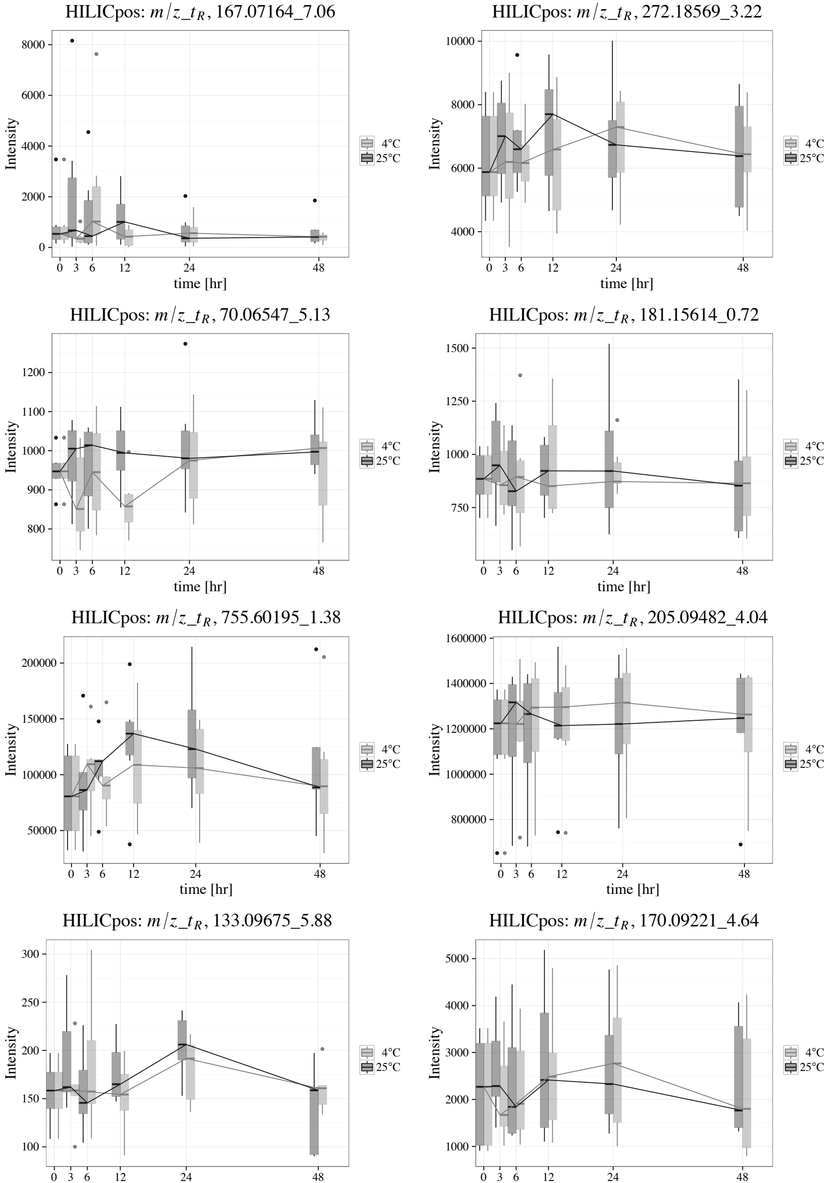

HILICpos: m/z_tR, 167.07164_7.06
Intensity
0
2000
4000
6000
8000
time [hr]
0 3 6 12 24 48
4°C
25°C
HILICpos: m/z_tR, 272.18569_3.22
Intensity
4000
6000
8000
10000
time [hr]
0 3 6 12 24 48
4°C
25°C
HILICpos: m/z_tR, 70.06547_5.13
Intensity
800
900
1000
1100
1200
time [hr]
0 3 6 12 24 48
4°C
25°C
HILICpos: m/z_tR, 181.15614_0.72
Intensity
750
1000
1250
1500
time [hr]
0 3 6 12 24 48
4°C
25°C
HILICpos: m/z_tR, 755.60195_1.38
Intensity
50000
100000
150000
200000
time [hr]
0 3 6 12 24 48
4°C
25°C
HILICpos: m/z_tR, 205.09482_4.04
Intensity
800000
1000000
1200000
1400000
1600000
time [hr]
0 3 6 12 24 48
4°C
25°C
HILICpos: m/z_tR, 133.09675_5.88
Intensity
100
150
200
250
300
0 3 6 12 24 48
4°C
25°C
HILICpos: m/z_tR, 170.09221_4.64
Intensity
1000
2000
3000
4000
5000
0 3 6 12 24 48
4°C
25°C

time [hr]

time [hr]

HILICpos: m/z_t_R, 288.21690_3.07

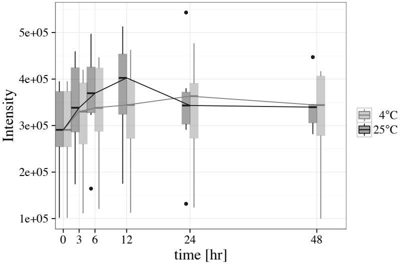

HILICpos: m/z_t_R, 782.56672_0.98

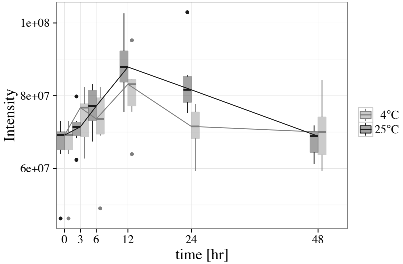

HILICpos: m/z_t_R, 172.13274_5.41

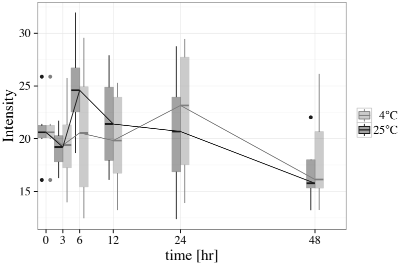

HILICpos: m/z_t_R, 174.11179_5.70

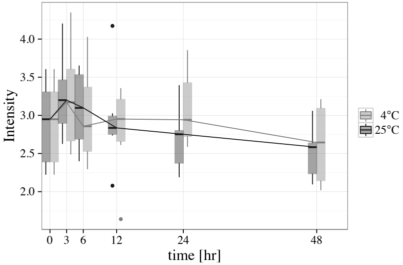

HILICpos: m/z_t_R, 307.11350_4.08

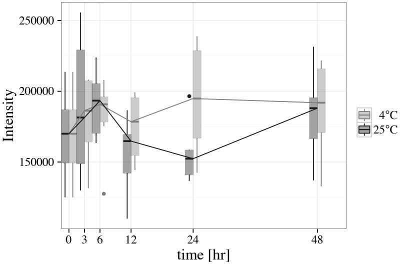

HILICpos: m/z_t_R, 614.53516_3.03

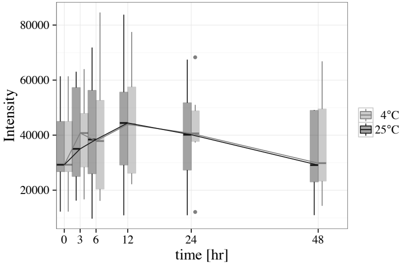

HILICpos: m/z_t_R, 260.18561_3.24

HILICpos: m/z_t_R, 116.07110_4.62

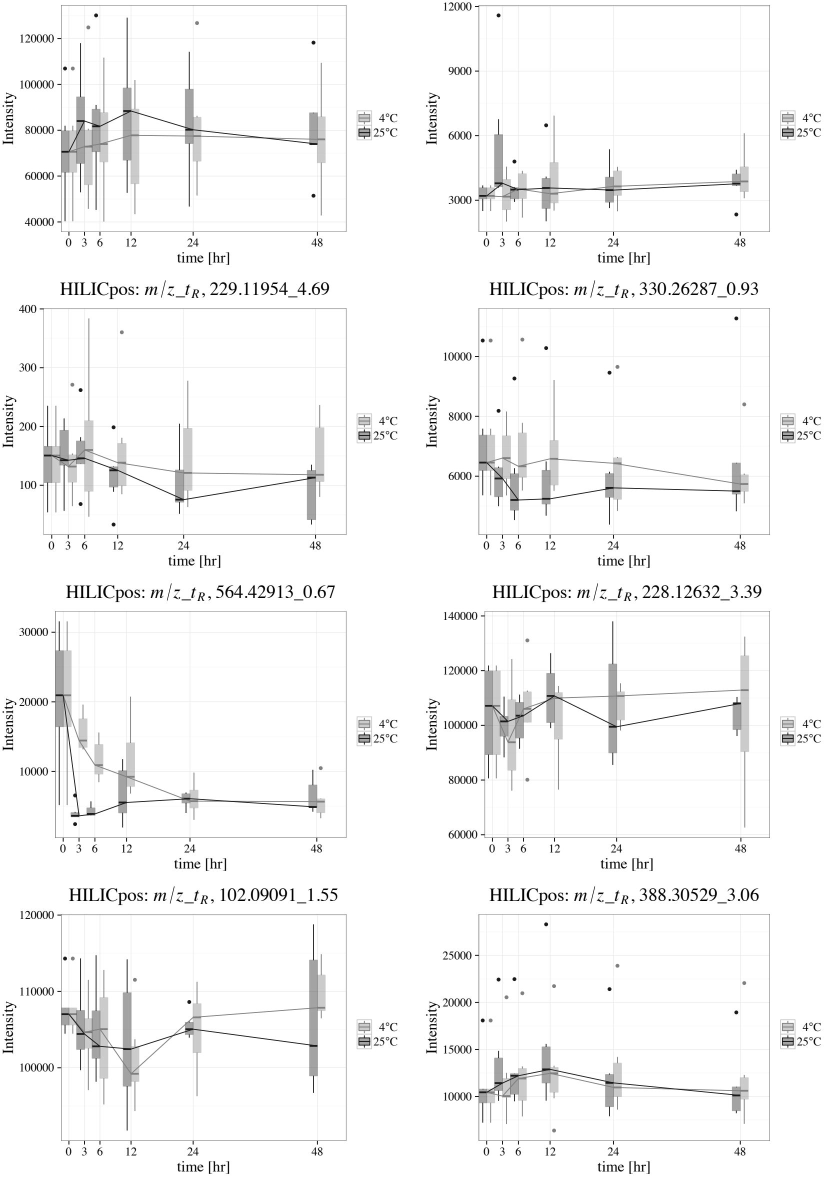
HILICpos: m/z_tR, 229.11954_4.69
HILICpos: m/z_tR, 330.26287_0.93
HILICpos: m/z_tR, 564.42913_0.67
HILICpos: m/z_tR, 228.12632_3.39
HILICpos: m/z_tR, 102.09091_1.55
HILICpos: m/z_tR, 388.30529_3.06
Intensity
time [hr]
4°C
25°C

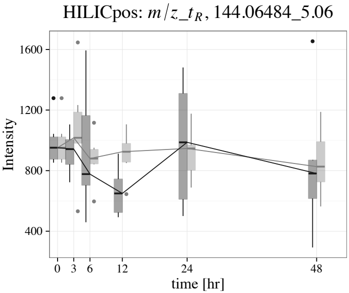
HILICpos: m/z_t_R, 144.06484_5.06
Intensity
time [hr]
4°C
25°C

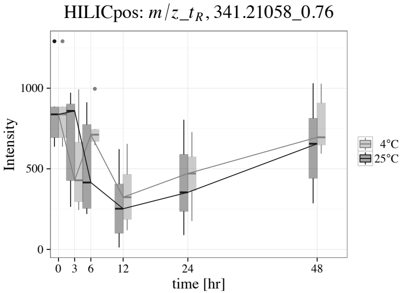
HILICpos: m/z_t_R, 341.21058_0.76
Intensity
time [hr]
4°C
25°C

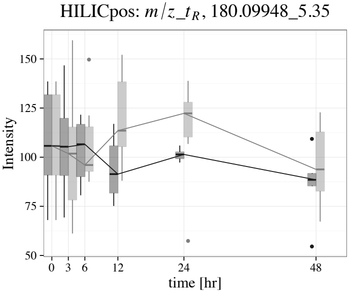
HILICpos: m/z_t_R, 180.09948_5.35
Intensity
time [hr]
4°C
25°C

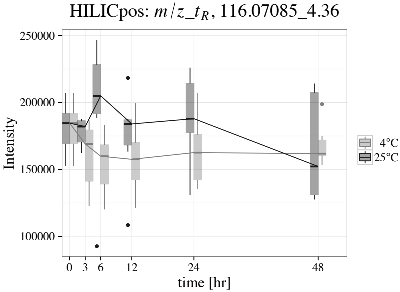
HILICpos: m/z_t_R, 116.07085_4.36
Intensity
time [hr]
4°C
25°C

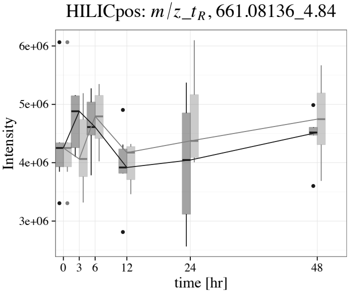
HILICpos: m/z_t_R, 661.08136_4.84
Intensity
time [hr]
4°C
25°C

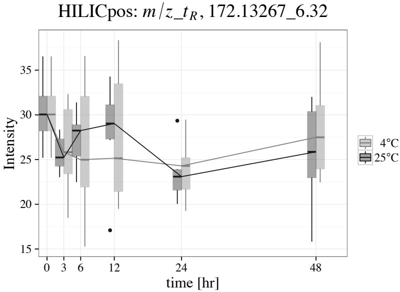
HILICpos: m/z_t_R, 172.13267_6.32
Intensity
time [hr]
4°C
25°C

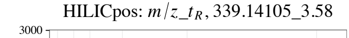
HILICpos: m/z_t_R, 339.14105_3.58
3000

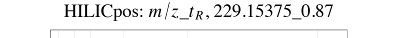
HILICpos: m/z_t_R, 229.15375_0.87

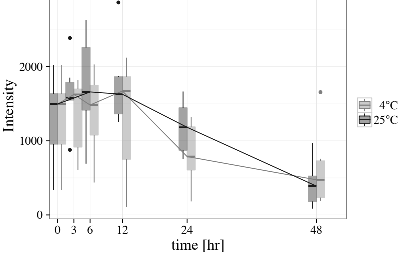

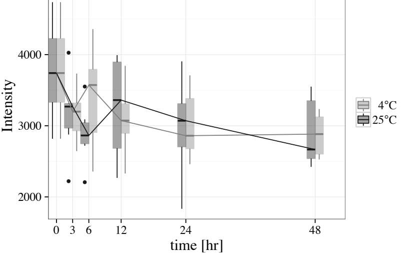

HILICpos: m/z_t_R, 259.09192_4.26

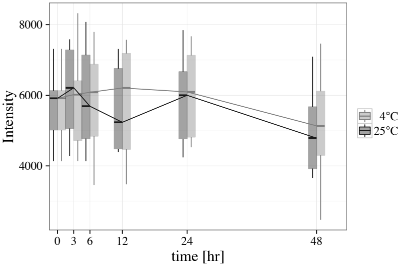

HILICpos: m/z_t_R, 268.22624_0.79

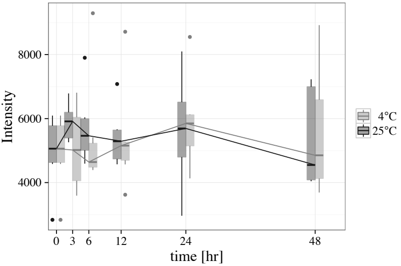

HILICpos: m/z_t_R, 369.35068_0.71

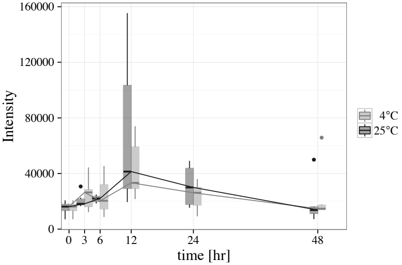

HILICpos: m/z_t_R, 372.07122_4.84

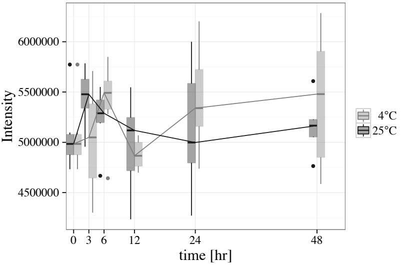

HILICpos: m/z_t_R, 276.11879_4.99

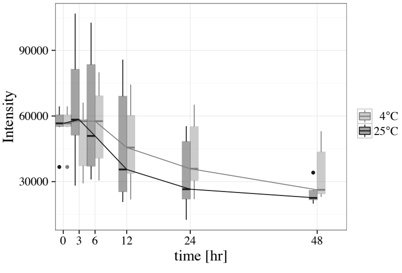

HILICpos: m/z_t_R, 154.08663_6.28

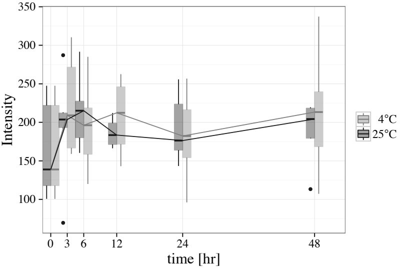

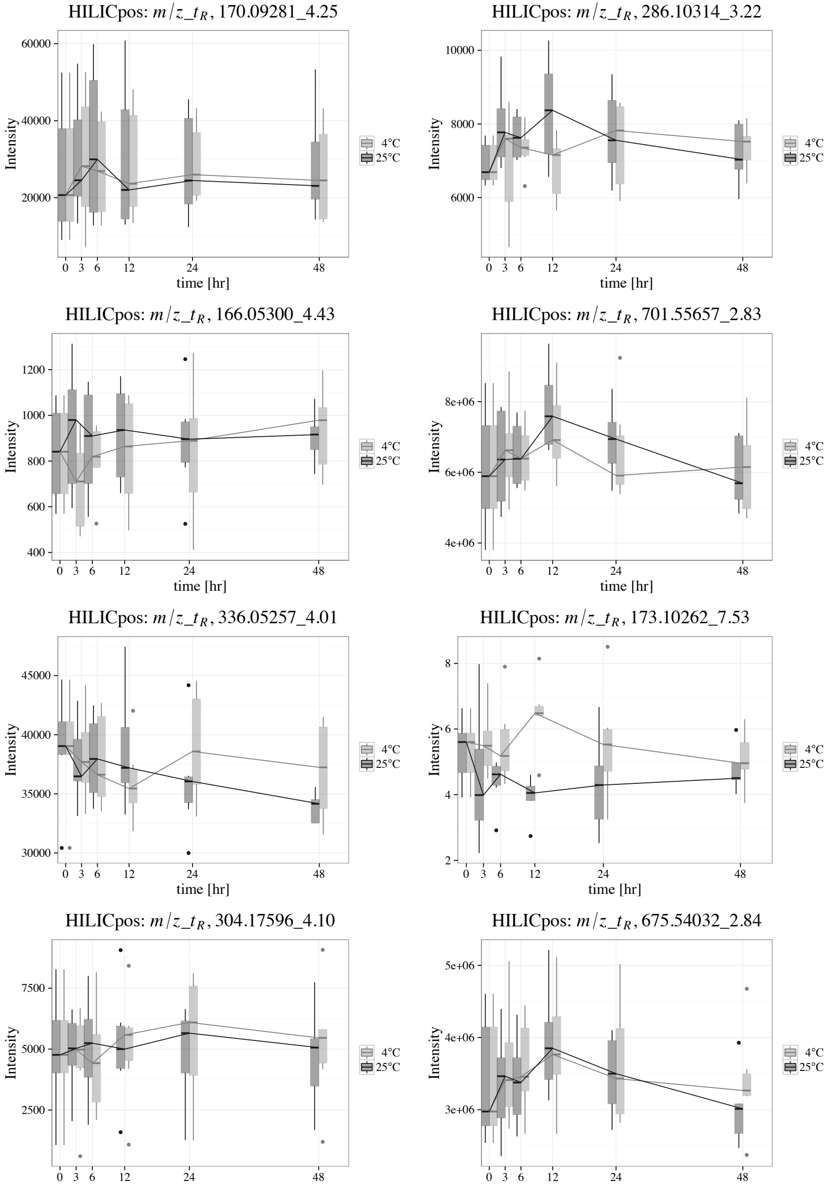
HILICpos: m/z_t_R, 170.09281_4.25
HILICpos: m/z_t_R, 286.10314_3.22
HILICpos: m/z_t_R, 166.05300_4.43
HILICpos: m/z_t_R, 701.55657_2.83
HILICpos: m/z_t_R, 336.05257_4.01
HILICpos: m/z_t_R, 173.10262_7.53
HILICpos: m/z_t_R, 304.17596_4.10
HILICpos: m/z_t_R, 675.54032_2.84
Intensity
time [hr]
0
3
6
12
24
48
4°C
25°C
60000
40000
20000
10000
8000
6000
1200
1000
800
600
400
8e+06
6e+06
4e+06
45000
40000
35000
30000
8
6
4
2
7500
5000
2500
5e+06
4e+06
3e+06

time [hr]

time [hr]

HILICpos: m/z_t_R, 160.07909_5.13

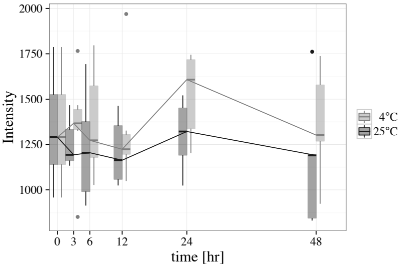

HILICpos: m/z_t_R, 340.04659_4.01

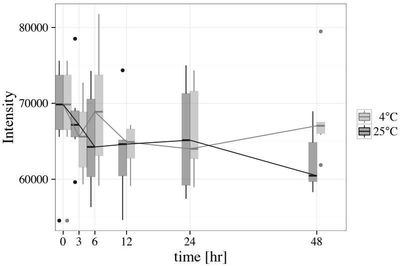

HILICpos: m/z_t_R, 181.11993_0.76

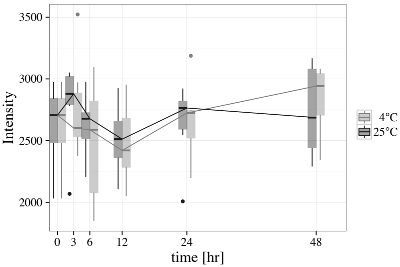

HILICpos: m/z_t_R, 317.06494_4.88

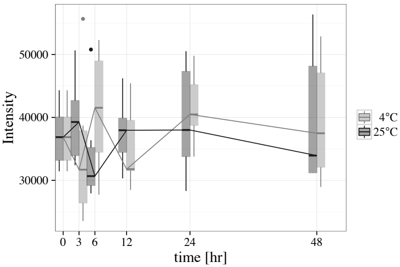

HILICpos: m/z_t_R, 159.14866_6.78

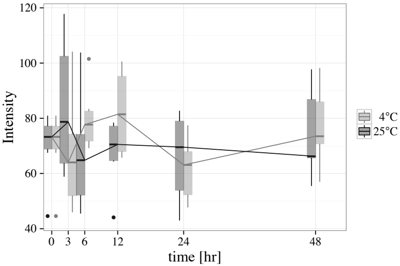

HILICpos: m/z_t_R, 717.05947_4.01

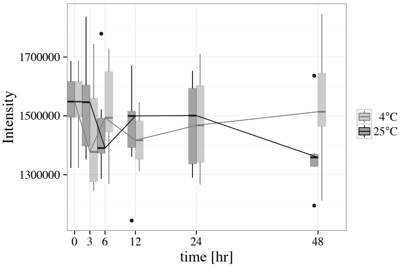

HILICpos: m/z_t_R, 716.55579_0.98

HILICpos: m/z_t_R, 175.11880_7.41

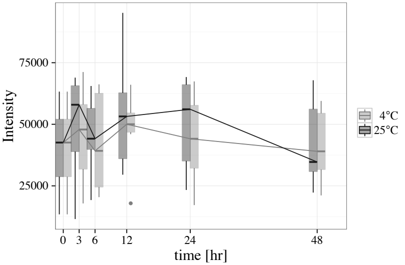

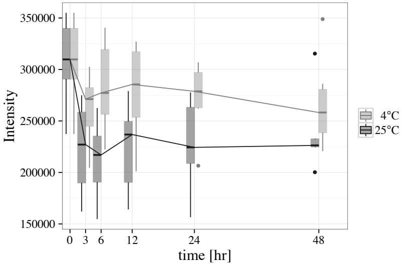

HILICpos: m/z_t_R, 156.07783_6.78

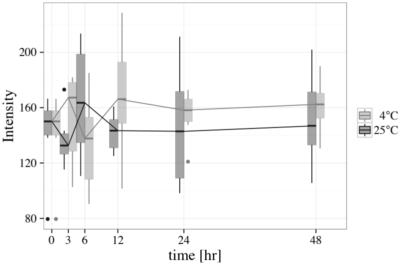

HILICpos: m/z_t_R, 89.07077_1.32

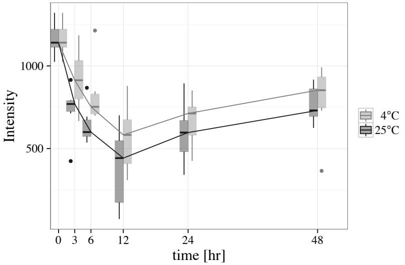

HILICpos: m/z_t_R, 436.08712_5.00

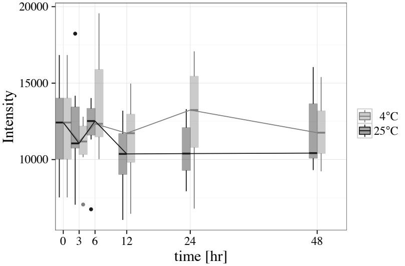

HILICpos: m/z_t_R, 140.10702_1.69

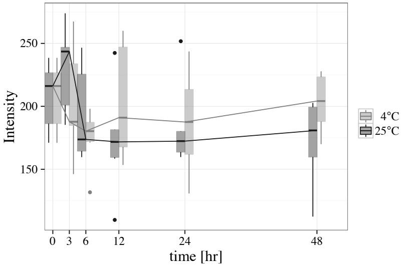

HILICpos: m/z_t_R, 302.13869_0.93

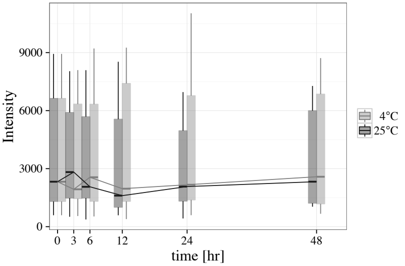

HILICpos: m/z_t_R, 144.06472_7.55

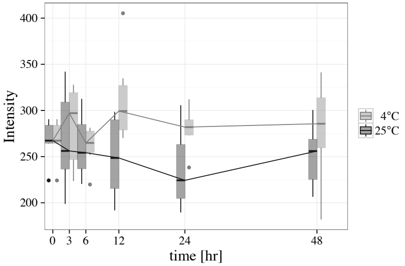

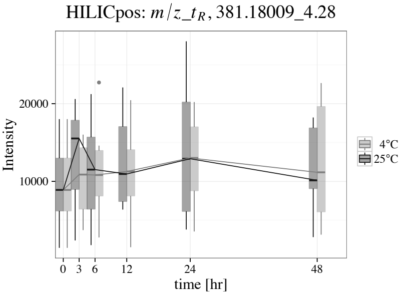
HILICpos: m/z_t_R, 381.18009_4.28
Intensity
10000
20000
0 3 6 12 24 48
time [hr]
4°C
25°C

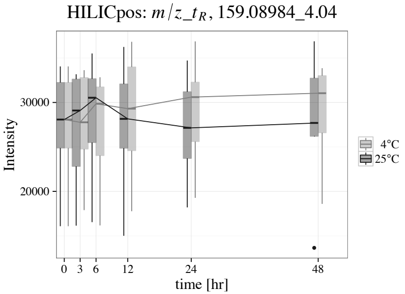
HILICpos: m/z_t_R, 159.08984_4.04
Intensity
20000
30000
0 3 6 12 24 48
time [hr]
4°C
25°C

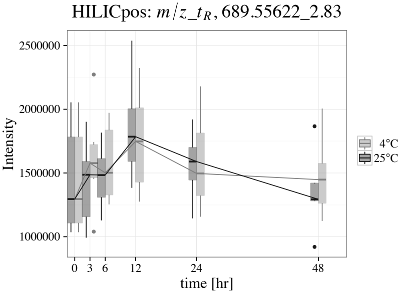
HILICpos: m/z_t_R, 689.55622_2.83
Intensity
1000000
1500000
2000000
2500000
0 3 6 12 24 48
time [hr]
4°C
25°C

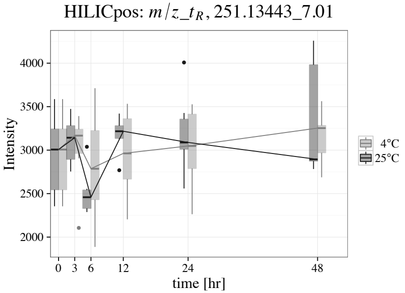
HILICpos: m/z_t_R, 251.13443_7.01
Intensity
2000
2500
3000
3500
4000
0 3 6 12 24 48
time [hr]
4°C
25°C

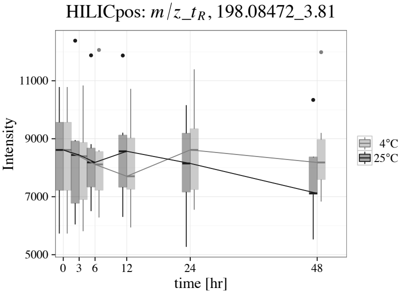
HILICpos: m/z_t_R, 198.08472_3.81
Intensity
5000
7000
9000
11000
0 3 6 12 24 48
time [hr]
4°C
25°C

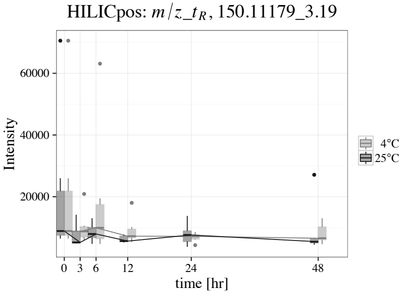
HILICpos: m/z_t_R, 150.11179_3.19
Intensity
20000
40000
60000
0 3 6 12 24 48
time [hr]
4°C
25°C

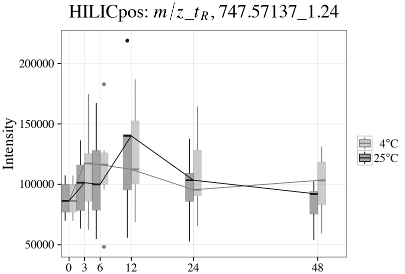
HILICpos: m/z_t_R, 747.57137_1.24
Intensity
50000
100000
150000
200000
0 3 6 12 24 48
4°C
25°C

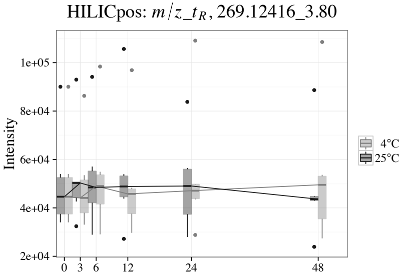
HILICpos: m/z_t_R, 269.12416_3.80
Intensity
2e+04
4e+04
6e+04
8e+04
1e+05
0 3 6 12 24 48
4°C
25°C

time [hr]

time [hr]

HILICpos: m/z_t_R, 345.06017_4.08

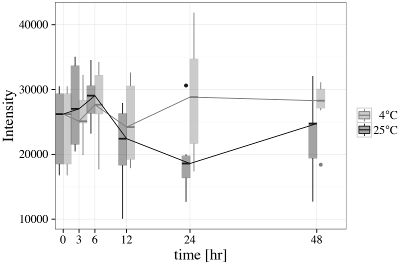

HILICpos: m/z_t_R, 282.12326_4.60

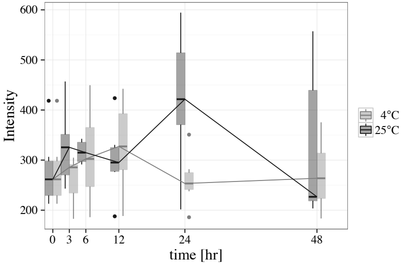

HILICpos: m/z_t_R, 286.20104_3.18

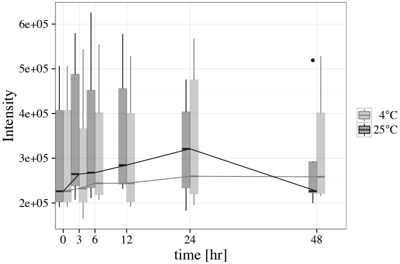

HILICpos: m/z_t_R, 761.58462_0.99

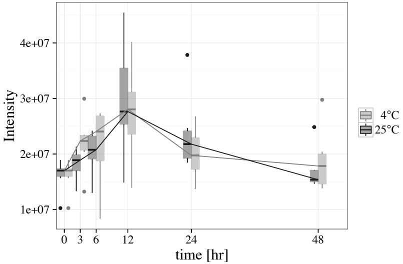

HILICpos: m/z_t_R, 188.06813_4.03

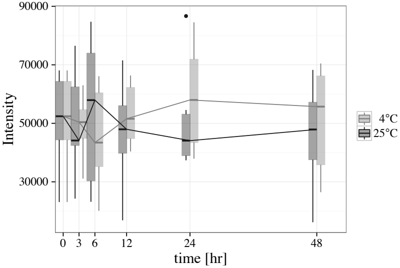

HILICpos: m/z_t_R, 372.21613_6.96

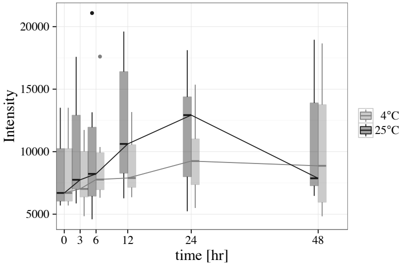

HILICpos: m/z_t_R, 764.55696_0.97

HILICpos: m/z_t_R, 276.14403_4.21

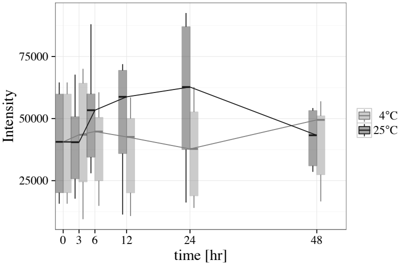

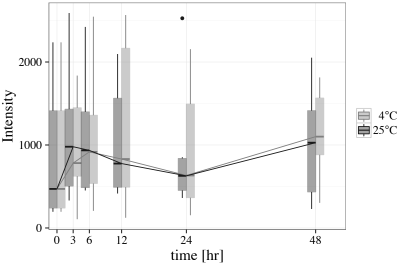

HILICpos: m/z_t_R, 159.14869_0.54

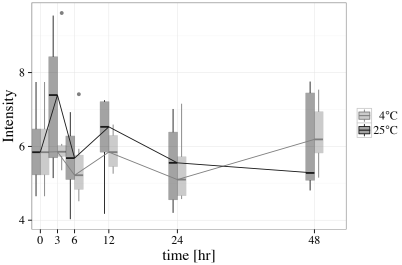

HILICpos: m/z_t_R, 772.58712_1.32

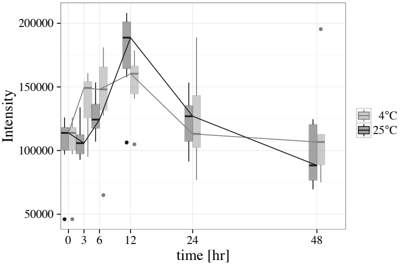

HILICpos: m/z_t_R, 310.23616_0.78

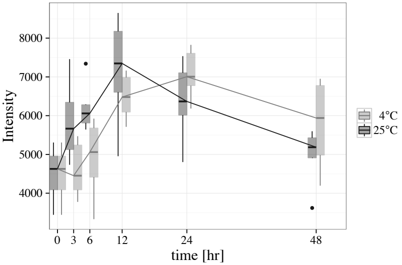

HILICpos: m/z_t_R, 257.11304_3.43

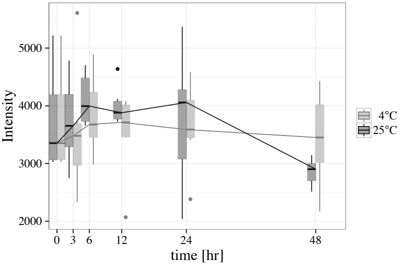

HILICpos: m/z_t_R, 380.25489_3.27

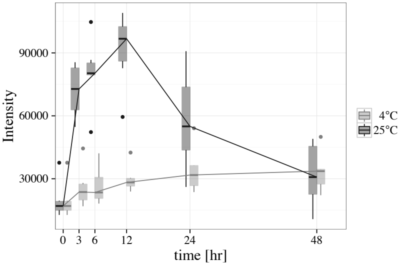

HILICpos: m/z_t_R, 182.08073_4.75

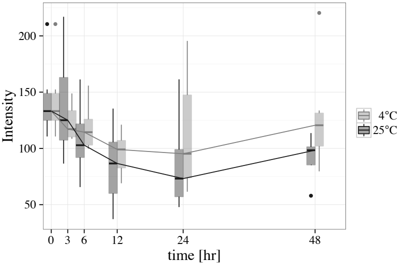

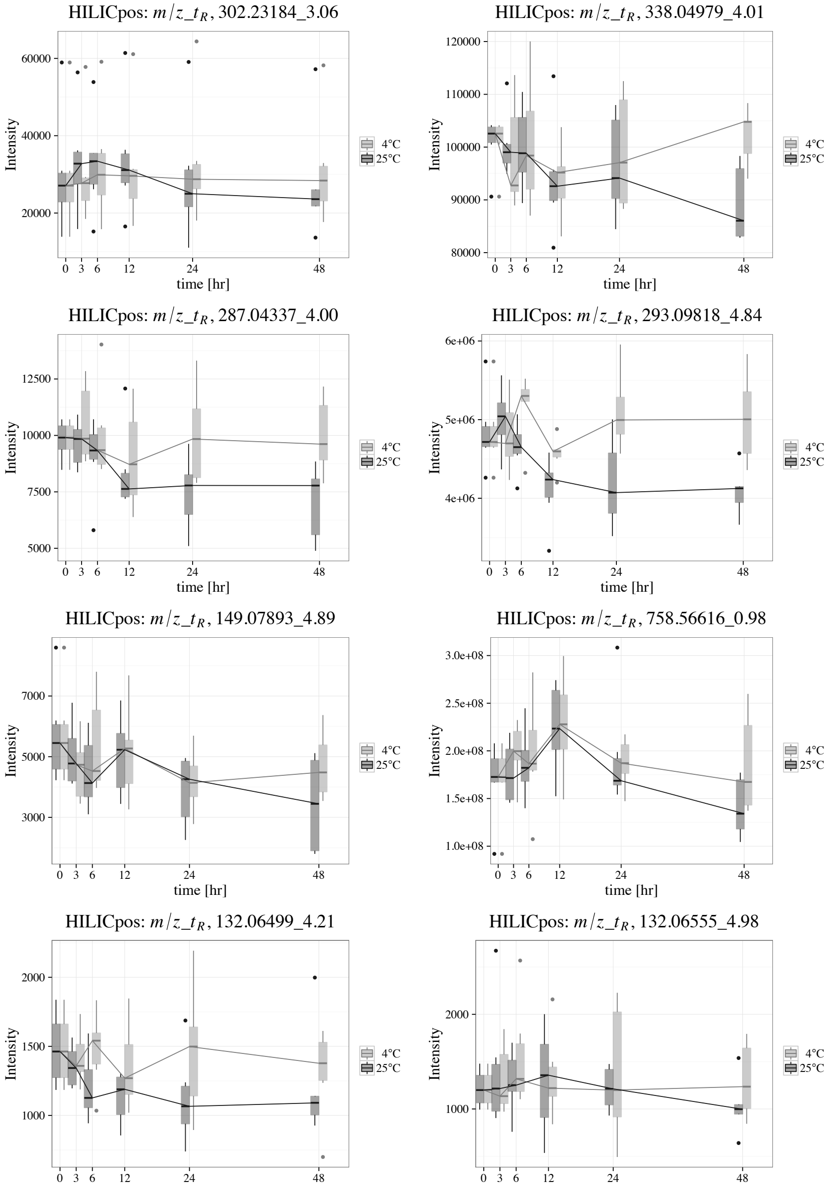

HILICpos: m/z_t_R, 302.23184_3.06
HILICpos: m/z_t_R, 338.04979_4.01
HILICpos: m/z_t_R, 287.04337_4.00
HILICpos: m/z_t_R, 293.09818_4.84
HILICpos: m/z_t_R, 149.07893_4.89
HILICpos: m/z_t_R, 758.56616_0.98
HILICpos: m/z_t_R, 132.06499_4.21
HILICpos: m/z_t_R, 132.06555_4.98
Intensity
time [hr]
4°C
25°C

time [hr]

time [hr]

HILICpos: m/z_t_R, 772.54656_0.97

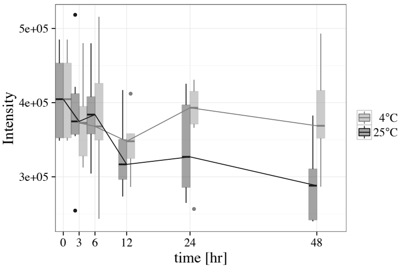

HILICpos: m/z_t_R, 142.08497_3.73

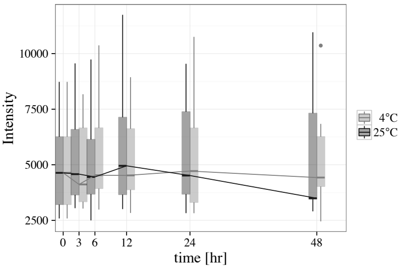

HILICpos: m/z_t_R, 240.10027_3.34

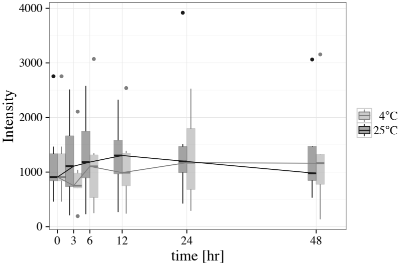

HILICpos: m/z_t_R, 72.08059_5.07

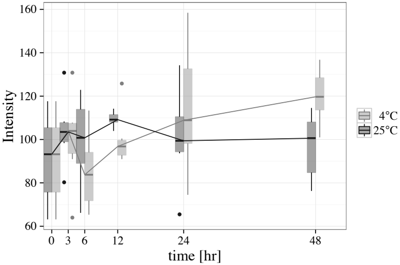

HILICpos: m/z_t_R, 279.10048_4.13

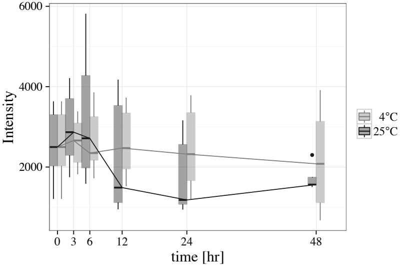

HILICpos: m/z_t_R, 208.13215_3.21

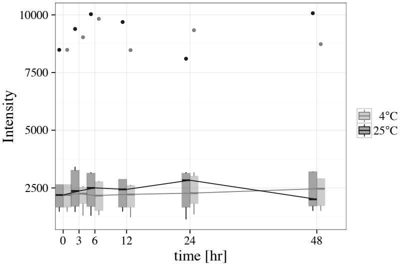

HILICpos: m/z_t_R, 312.21653_3.06

HILICpos: m/z_t_R, 370.29443_2.94

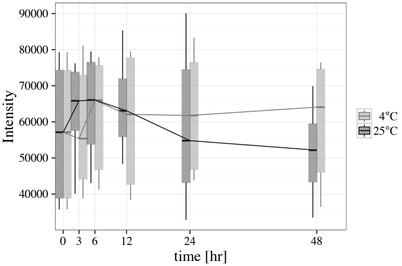

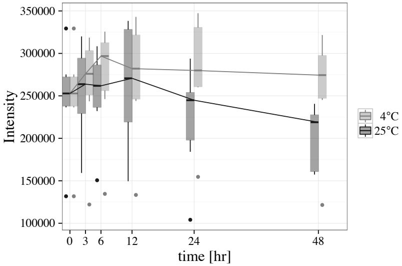

HILICpos: m/z_t_R, 178.08423_4.16

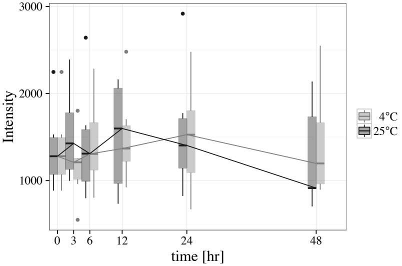

HILICpos: m/z_t_R, 74.05987_4.69

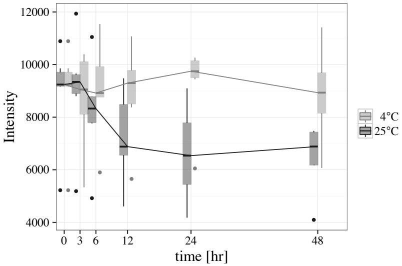

HILICpos: m/z_t_R, 330.22748_3.08

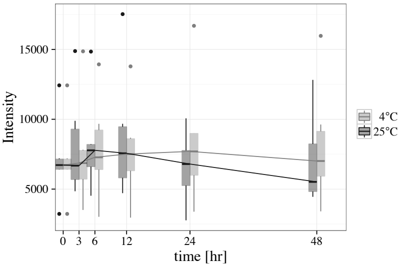

HILICpos: m/z_t_R, 647.51008_1.47

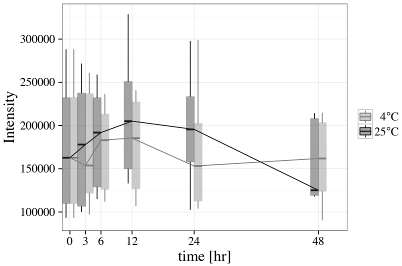

HILICpos: m/z_t_R, 328.24785_3.01

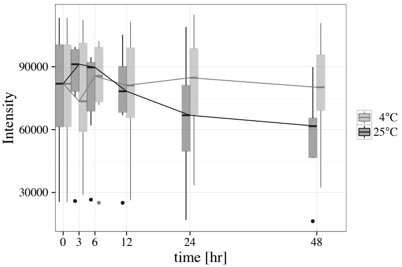

HILICpos: m/z_t_R, 342.04367_4.01

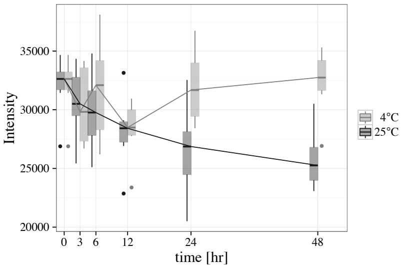

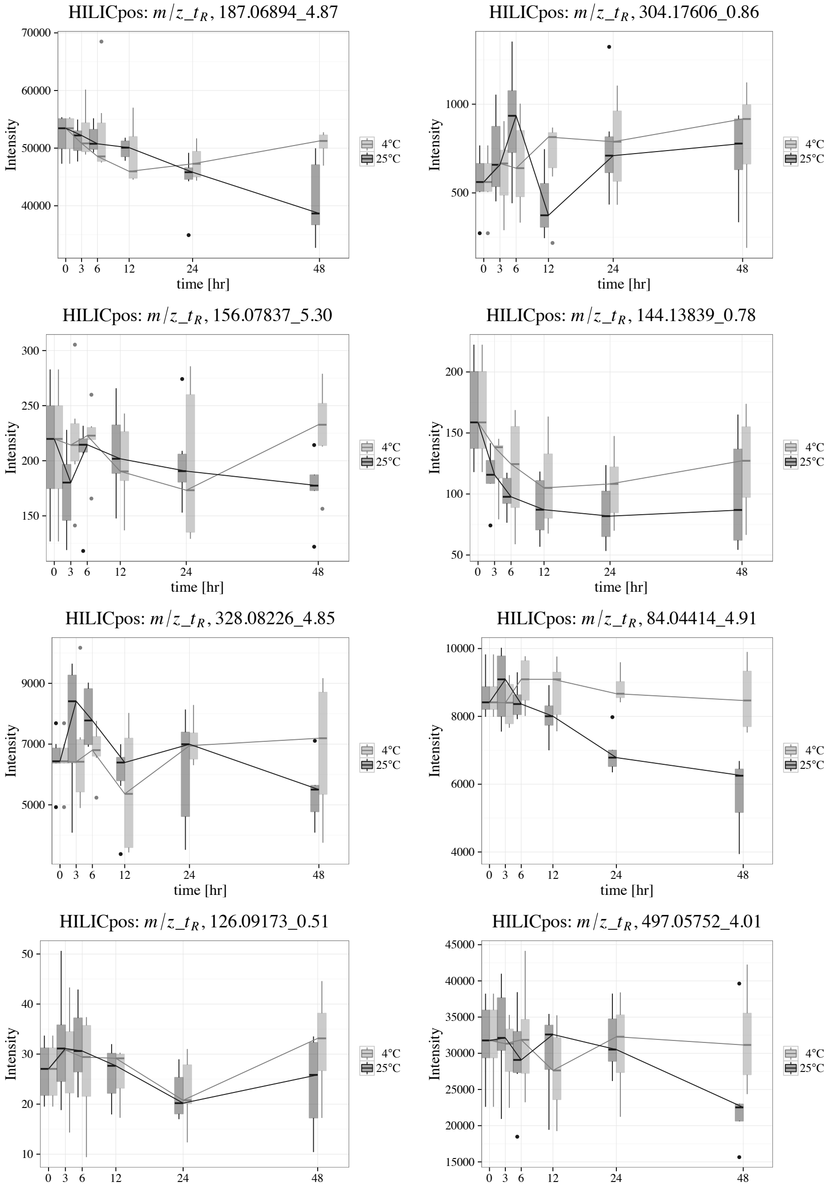
HILICpos: m/z_tR, 187.06894_4.87
HILICpos: m/z_tR, 304.17606_0.86
HILICpos: m/z_tR, 156.07837_5.30
HILICpos: m/z_tR, 144.13839_0.78
HILICpos: m/z_tR, 328.08226_4.85
HILICpos: m/z_tR, 84.04414_4.91
HILICpos: m/z_tR, 126.09173_0.51
HILICpos: m/z_tR, 497.05752_4.01
Intensity
time [hr]
0
3
6
12
24
48
4°C
25°C

time [hr]

time [hr]

HILICpos: m/z_t_R, 480.30775_2.98

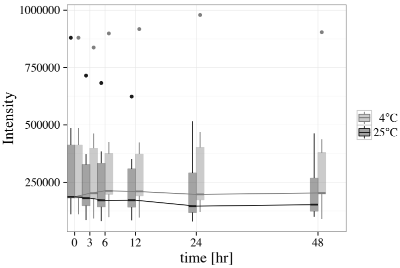

HILICpos: m/z_t_R, 189.08523_4.84

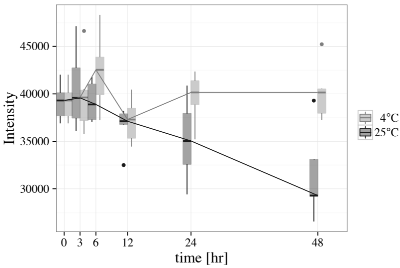

HILICpos: m/z_t_R, 156.07646_4.79

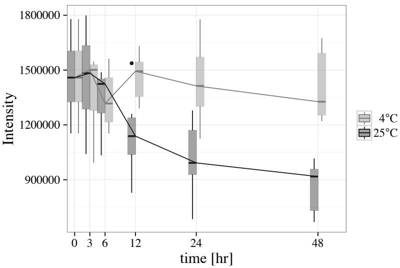

HILICpos: m/z_t_R, 169.05846_1.62

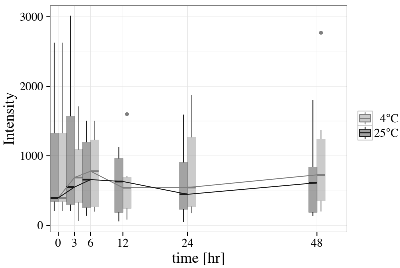

HILICpos: m/z_t_R, 267.11214_7.10

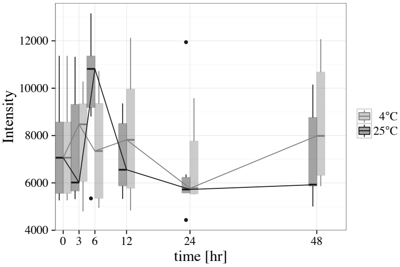

HILICpos: m/z_t_R, 356.24330_3.07

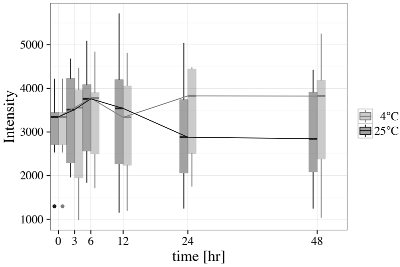

HILICpos: m/z_t_R, 156.07800_4.79

HILICpos: m/z_t_R, 184.13237_1.68

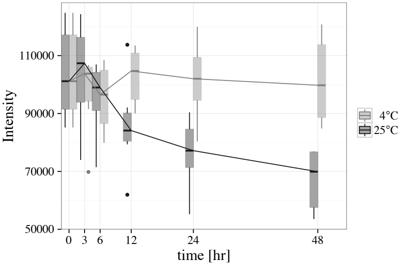

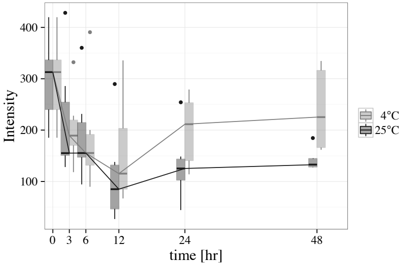

HILICpos: m/z_t_R, 344.27912_2.95

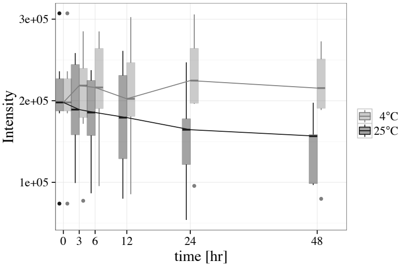

HILICpos: m/z_t_R, 358.29473_2.92

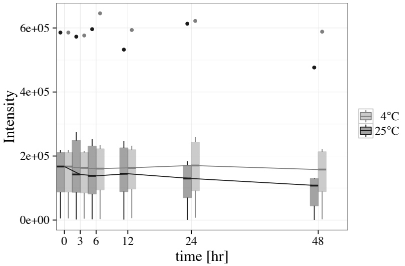

HILICpos: m/z_t_R, 180.13795_3.60

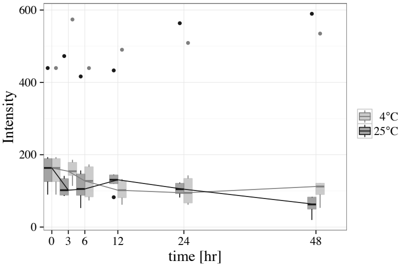

HILICpos: m/z_t_R, 284.18542_3.26

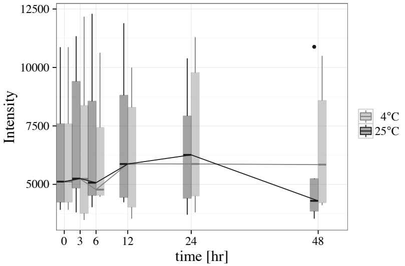

HILICpos: m/z_t_R, 283.04931_4.01

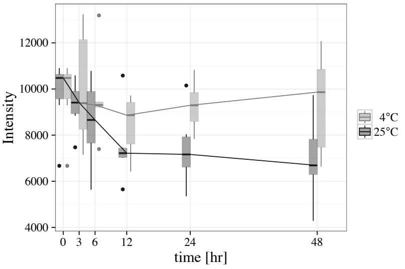

HILICpos: m/z_t_R, 772.58242_1.59

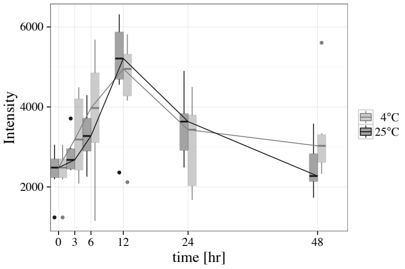

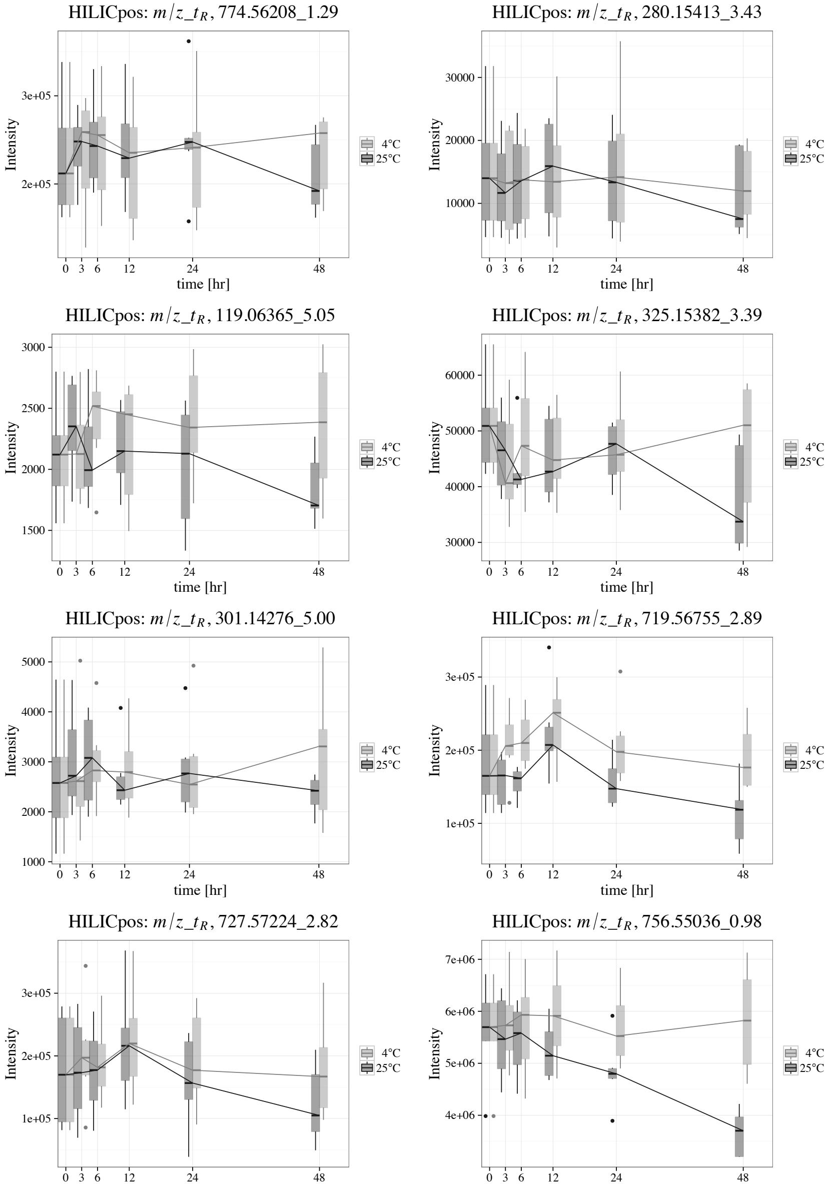
HILICpos: m/z_t_R, 774.56208_1.29
Intensity
2e+05
3e+05
0 3 6 12 24 48
time [hr]
4°C
25°C
HILICpos: m/z_t_R, 280.15413_3.43
10000
20000
30000
HILICpos: m/z_t_R, 119.06365_5.05
1500
2000
2500
3000
HILICpos: m/z_t_R, 325.15382_3.39
30000
40000
50000
60000
HILICpos: m/z_t_R, 301.14276_5.00
1000
2000
3000
4000
5000
HILICpos: m/z_t_R, 719.56755_2.89
1e+05
2e+05
3e+05
HILICpos: m/z_t_R, 727.57224_2.82
1e+05
2e+05
3e+05
HILICpos: m/z_t_R, 756.55036_0.98
4e+06
5e+06
6e+06
7e+06

time [hr]

time [hr]

HILICpos: m/z_t_R, 746.56783_0.97

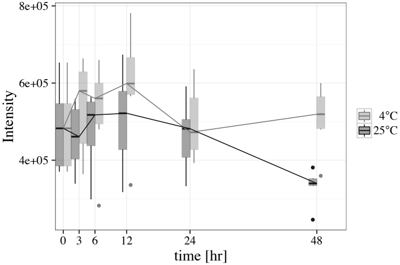

HILICpos: m/z_t_R, 278.01329_5.53

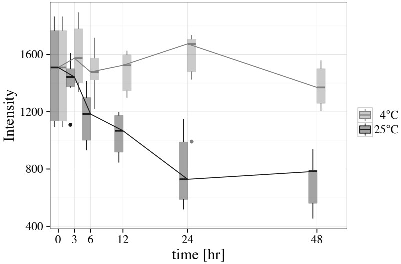

HILICpos: m/z_t_R, 247.09170_4.84

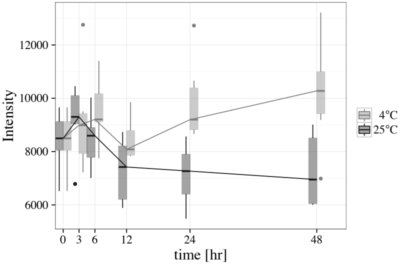

HILICpos: m/z_t_R, 362.16528_3.40

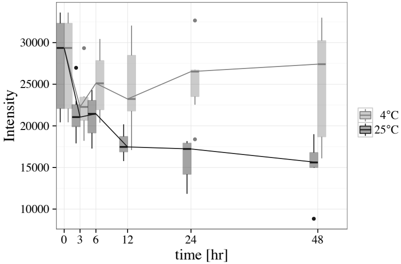

HILICpos: m/z_t_R, 169.05883_3.24

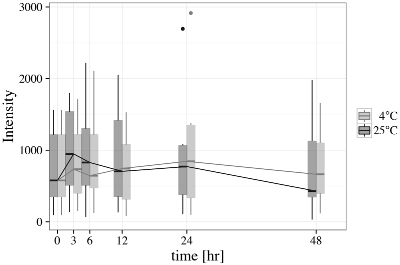

HILICpos: m/z_t_R, 342.26356_2.97

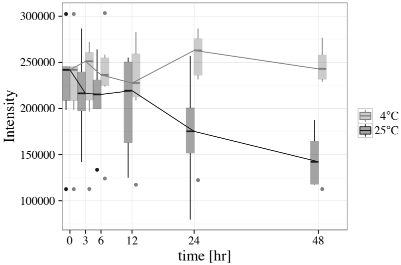

HILICpos: m/z_t_R, 192.10225_3.80

HILICpos: m/z_t_R, 368.27922_2.94

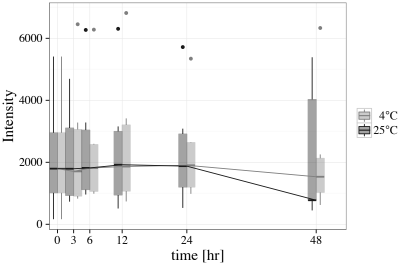

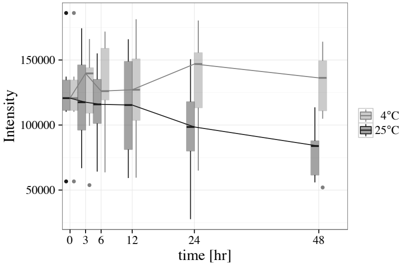

HILICpos: m/z_t_R, 121.06773_4.72

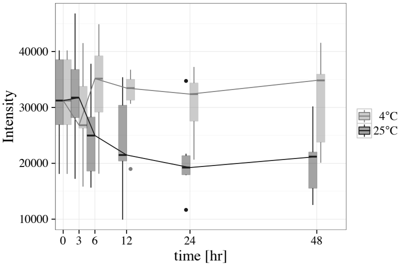

HILICpos: m/z_t_R, 316.24787_3.02

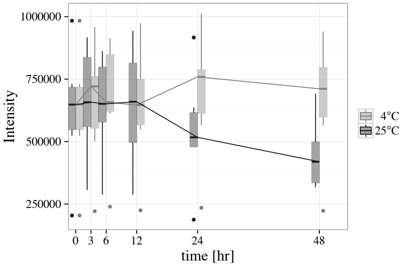

HILICpos: m/z_t_R, 730.53637_0.98

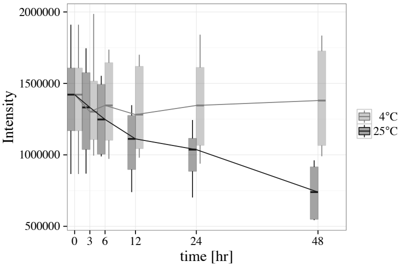

HILICpos: m/z_t_R, 144.10179_3.57

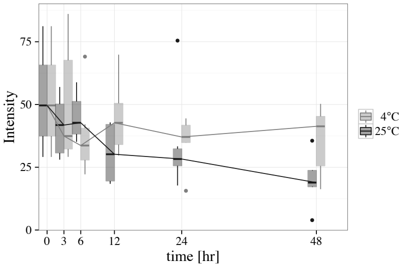

HILICpos: m/z_t_R, 314.23229_3.03

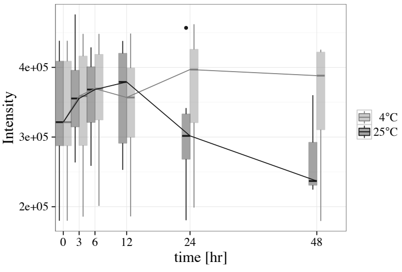

HILICpos: m/z_t_R, 169.05837_2.91

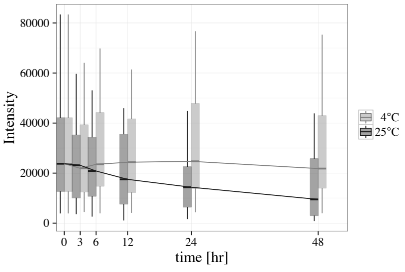

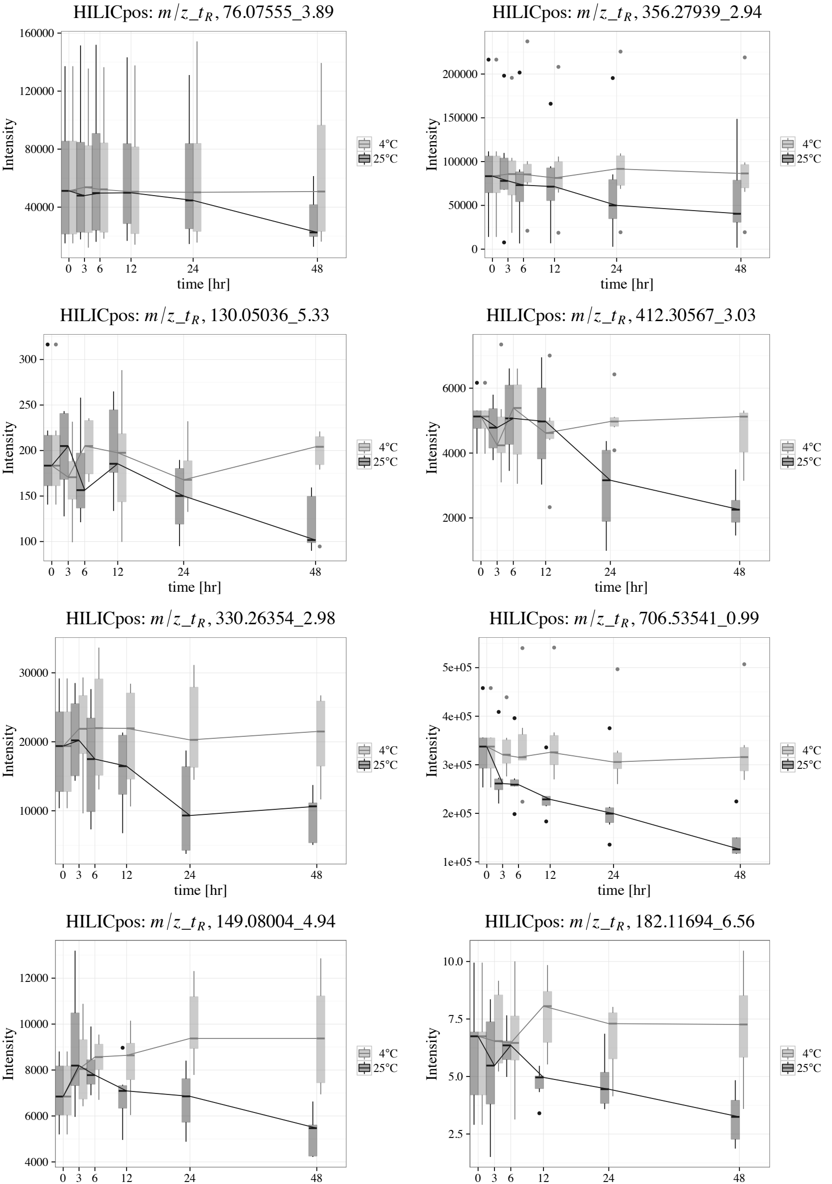
HILICpos: m/z_t_R, 76.07555_3.89
HILICpos: m/z_t_R, 356.27939_2.94
HILICpos: m/z_t_R, 130.05036_5.33
HILICpos: m/z_t_R, 412.30567_3.03
HILICpos: m/z_t_R, 330.26354_2.98
HILICpos: m/z_t_R, 706.53541_0.99
HILICpos: m/z_t_R, 149.08004_4.94
HILICpos: m/z_t_R, 182.11694_6.56
Intensity
time [hr]
0
3
6
12
24
48
4°C
25°C

time [hr]

time [hr]

HILICpos: m/z_t_R, 807.15272_4.90

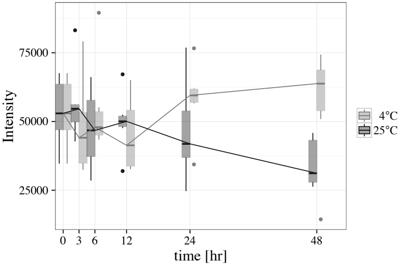

HILICpos: m/z_t_R, 358.16397_4.94

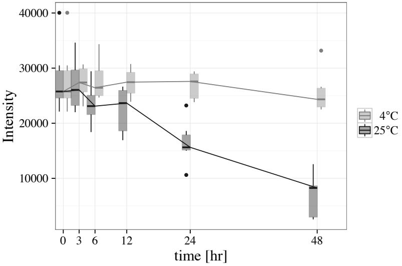

HILICpos: m/z_t_R, 526.29184_2.94

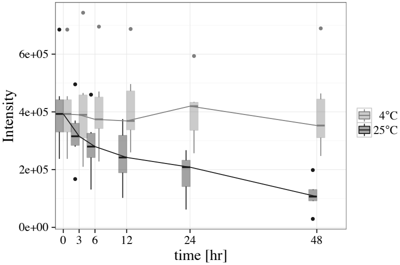

HILICpos: m/z_t_R, 478.29190_2.98

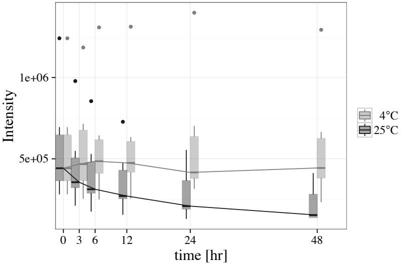

HILICpos: m/z_t_R, 706.53513_1.28

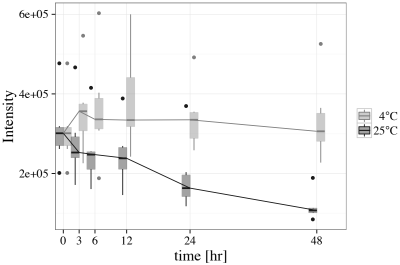

HILICpos: m/z_t_R, 647.51010_2.86

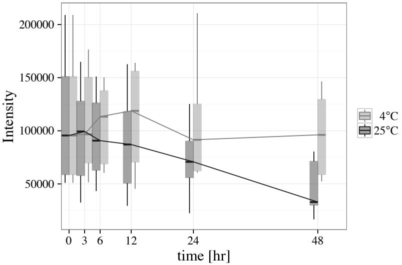

HILICpos: m/z_t_R, 329.14877_5.03

HILICpos: m/z_t_R, 338.23273_3.03

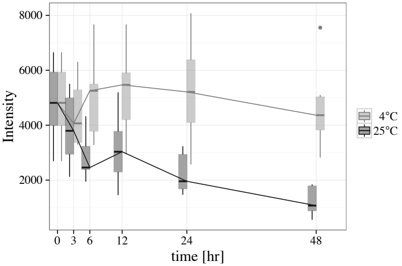

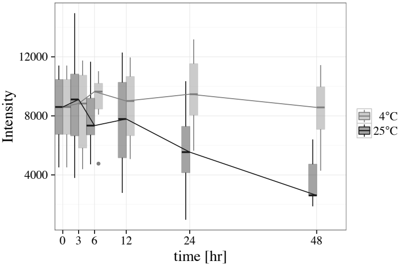

HILICpos: m/z_t_R, 234.07630_4.70

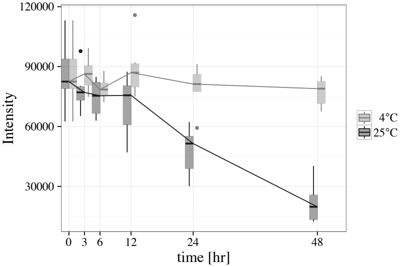

HILICpos: m/z_t_R, 732.55166_0.98

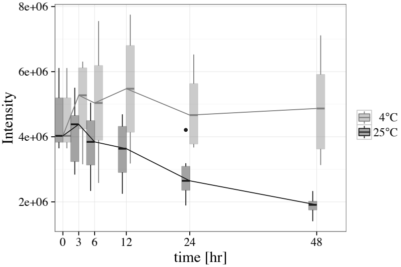

HILICpos: m/z_t_R, 502.29187_2.96

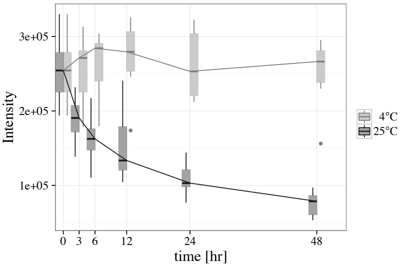

HILICpos: m/z_t_R, 100.07602_3.53

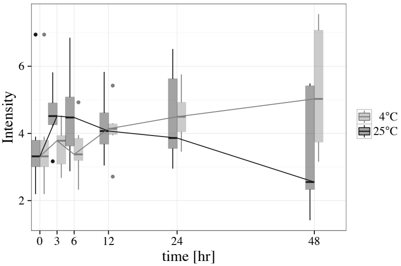

HILICpos: m/z_t_R, 311.12452_4.68

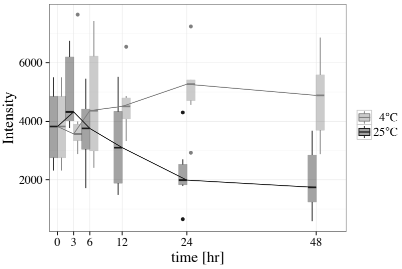

HILICpos: m/z_t_R, 450.32083_2.84

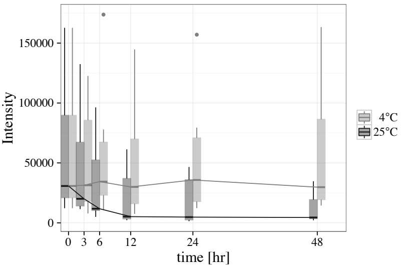

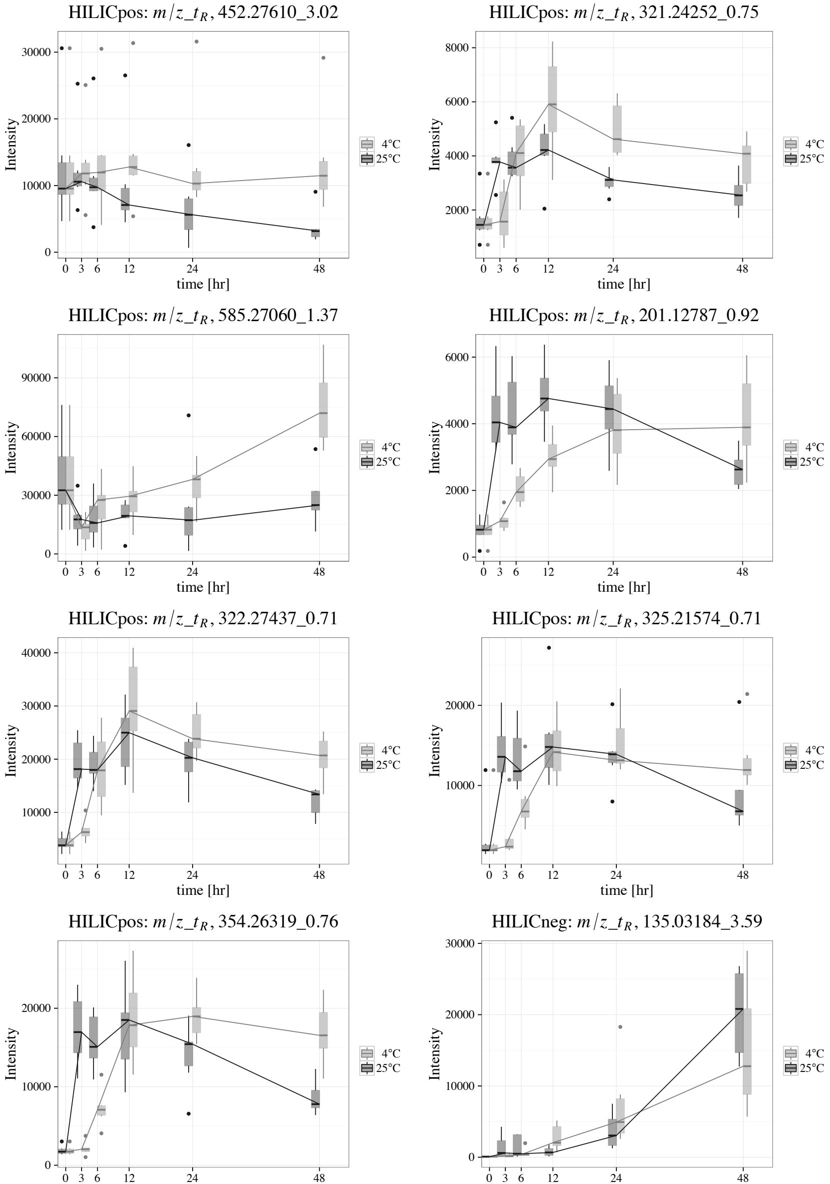
HILICpos: m/z_t_R, 452.27610_3.02
HILICpos: m/z_t_R, 321.24252_0.75
HILICpos: m/z_t_R, 585.27060_1.37
HILICpos: m/z_t_R, 201.12787_0.92
HILICpos: m/z_t_R, 322.27437_0.71
HILICpos: m/z_t_R, 325.21574_0.71
HILICpos: m/z_t_R, 354.26319_0.76
HILICneg: m/z_t_R, 135.03184_3.59
Intensity
time [hr]
4°C
25°C

time [hr]

time [hr]

HILICneg: m/z_t_R, 179.05594_3.01

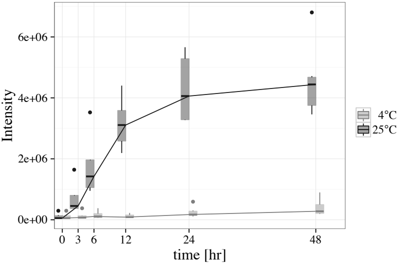

HILICneg: m/z_t_R, 257.07779_3.78

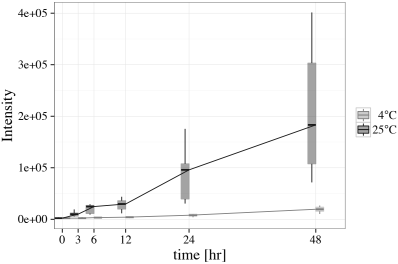

HILICneg: m/z_t_R, 185.05656_3.83

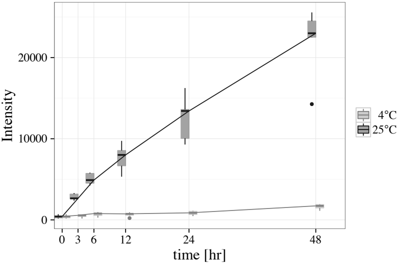

HILICneg: m/z_t_R, 135.03106_3.50

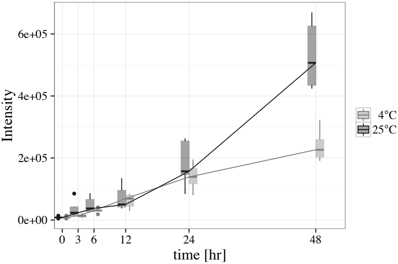

HILICneg: m/z_t_R, 214.04836_5.15

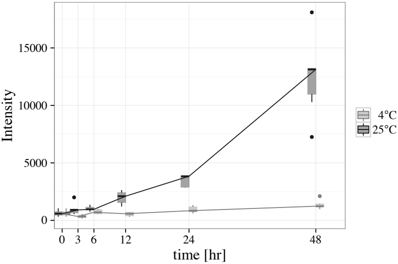

HILICneg: m/z_t_R, 190.05419_2.16

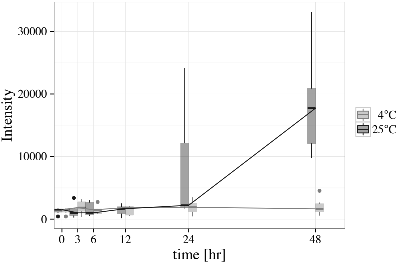

HILICneg: m/z_t_R, 177.04039_3.96

HILICneg: m/z_t_R, 155.00950_3.72

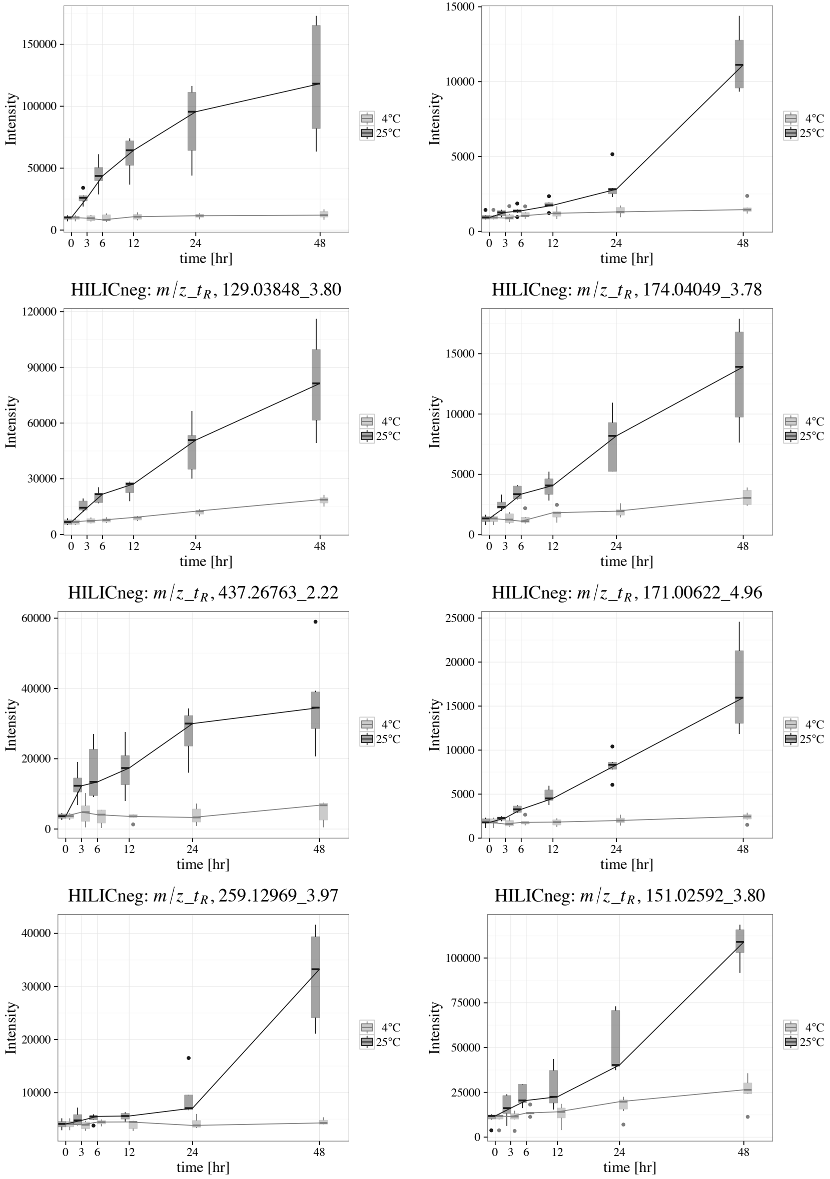

Intensity
time [hr]
4°C
25°C
HILICneg: m/z_t_R, 129.03848_3.80
HILICneg: m/z_t_R, 174.04049_3.78
HILICneg: m/z_t_R, 437.26763_2.22
HILICneg: m/z_t_R, 171.00622_4.96
HILICneg: m/z_t_R, 259.12969_3.97
HILICneg: m/z_t_R, 151.02592_3.80

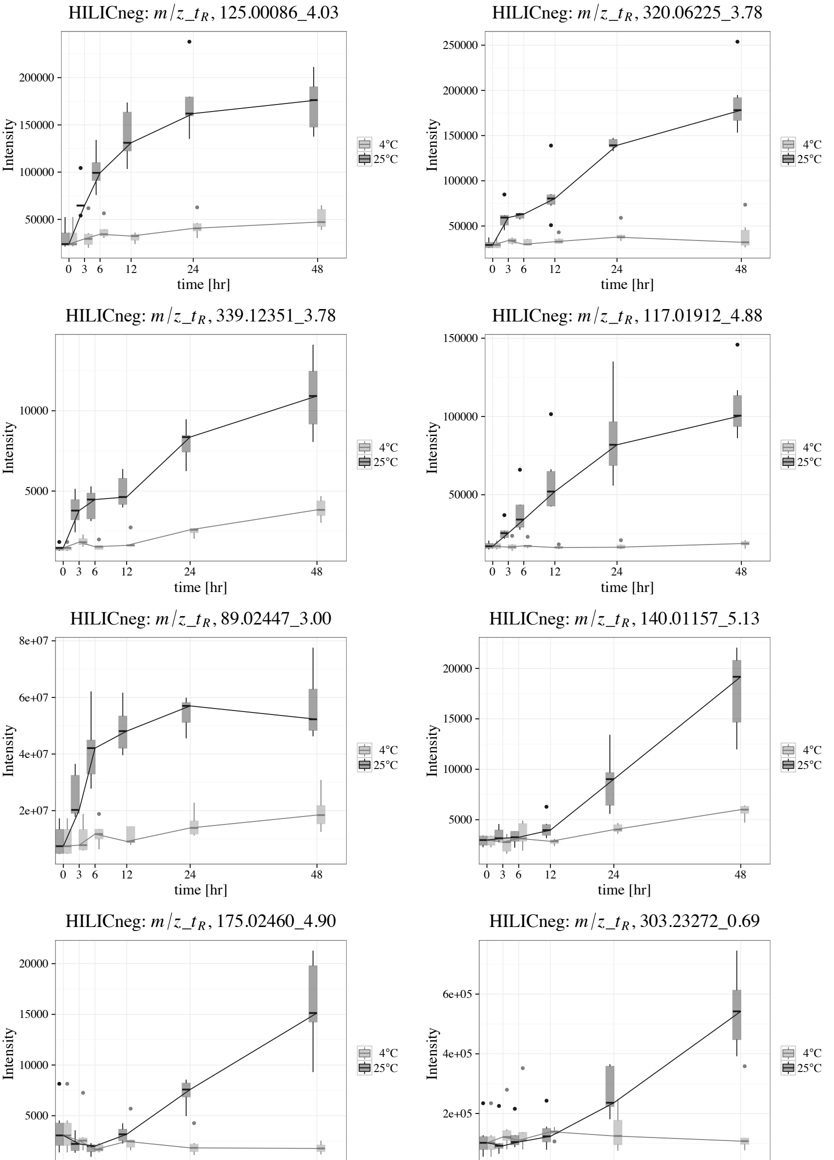

HILICneg: m/z_t_R, 125.00086_4.03
HILICneg: m/z_t_R, 320.06225_3.78
HILICneg: m/z_t_R, 339.12351_3.78
HILICneg: m/z_t_R, 117.01912_4.88
HILICneg: m/z_t_R, 89.02447_3.00
HILICneg: m/z_t_R, 140.01157_5.13
HILICneg: m/z_t_R, 175.02460_4.90
HILICneg: m/z_t_R, 303.23272_0.69
Intensity
time [hr]
0 3 6 12 24 48
4°C
25°C

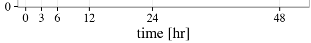

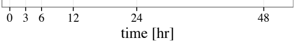

HILICneg: m/z_t_R, 175.07241_3.99

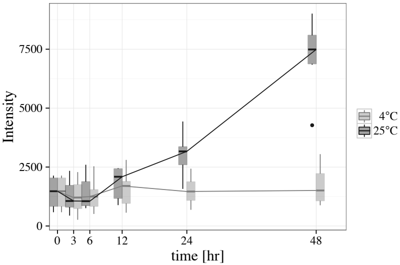

HILICneg: m/z_t_R, 87.00875_1.88

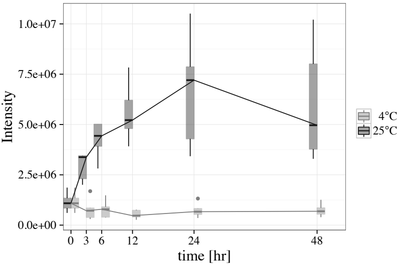

HILICneg: m/z_t_R, 113.03552_1.48

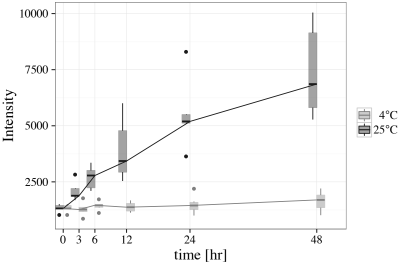

HILICneg: m/z_t_R, 301.21689_0.70

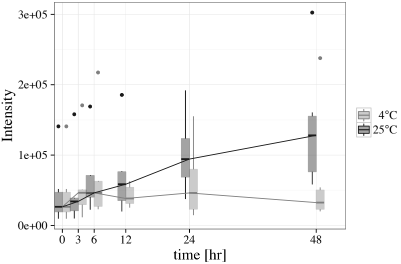

HILICneg: m/z_t_R, 608.35716_2.93

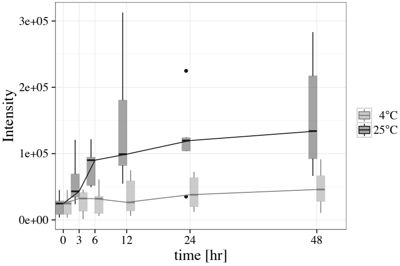

HILICneg: m/z_t_R, 245.11398_4.25

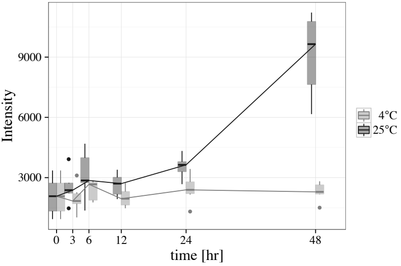

HILICneg: m/z_t_R, 87.00882_1.37

HILICneg: m/z_t_R, 145.01410_4.93

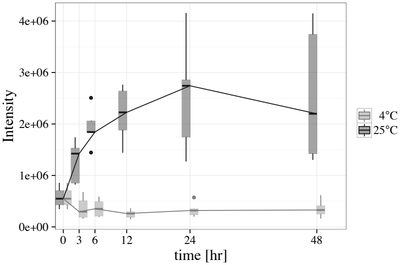

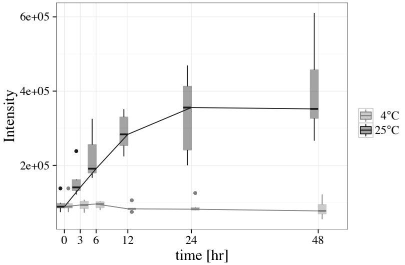

HILICneg: m/z_t_R, 135.02962_3.10

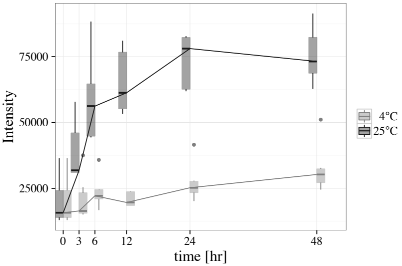

HILICneg: m/z_t_R, 401.08762_0.82

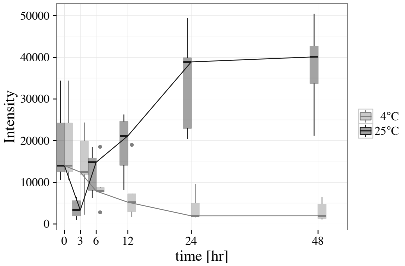

HILICneg: m/z_t_R, 146.04558_4.85

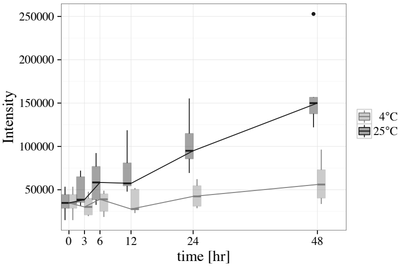

HILICneg: m/z_t_R, 133.01405_5.06

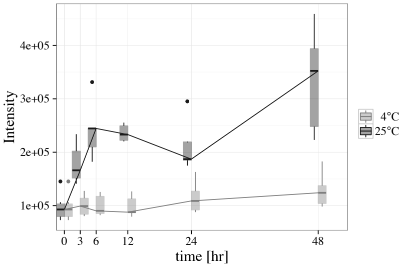

HILICneg: m/z_t_R, 293.11425_3.96

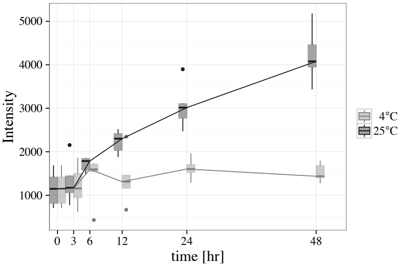

HILICneg: m/z_t_R, 345.09760_4.51

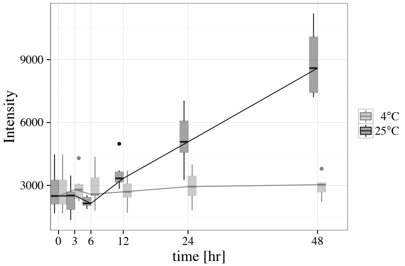

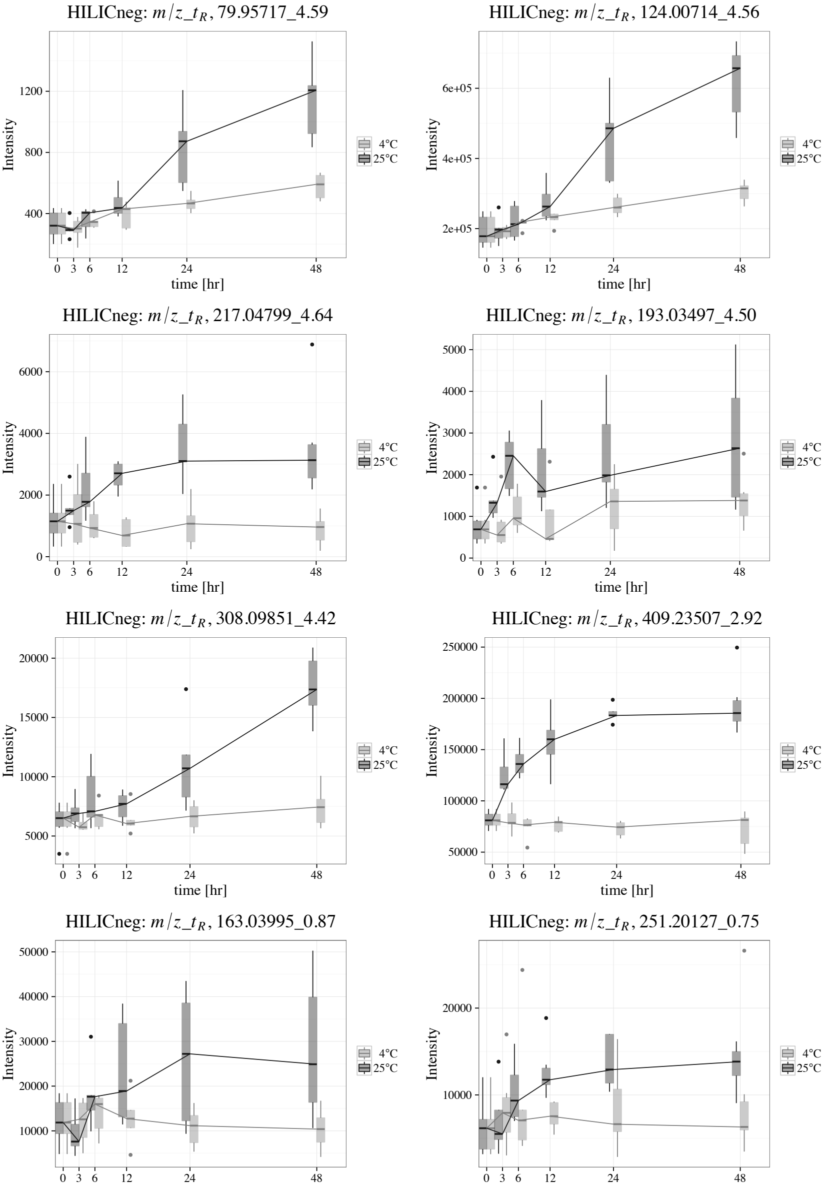
HILICneg: m/z_t_R, 79.95717_4.59
HILICneg: m/z_t_R, 124.00714_4.56
HILICneg: m/z_t_R, 217.04799_4.64
HILICneg: m/z_t_R, 193.03497_4.50
HILICneg: m/z_t_R, 308.09851_4.42
HILICneg: m/z_t_R, 409.23507_2.92
HILICneg: m/z_t_R, 163.03995_0.87
HILICneg: m/z_t_R, 251.20127_0.75
Intensity
time [hr]
0 3 6 12 24 48
4°C
25°C

time [hr]

time [hr]

HILICneg: m/z_t_R, 135.02968_4.05

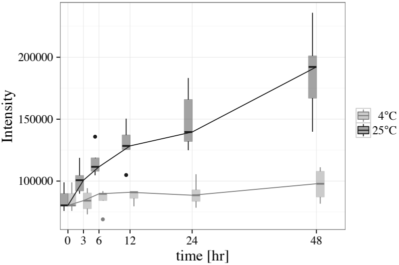

HILICneg: m/z_t_R, 218.10321_3.52

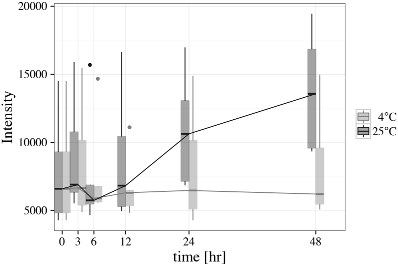

HILICneg: m/z_t_R, 159.02960_0.97

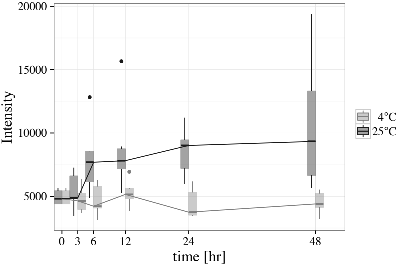

HILICneg: m/z_t_R, 285.11229_4.58

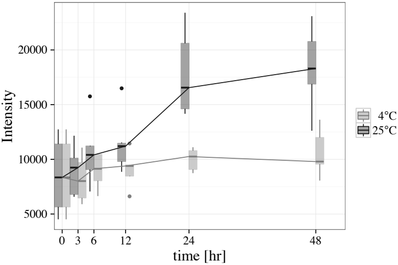

HILICneg: m/z_t_R, 203.06714_5.00

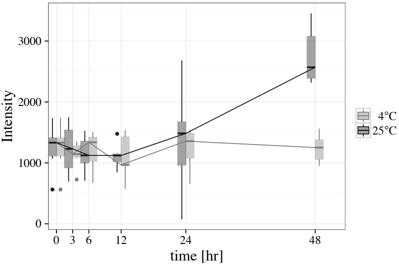

HILICneg: m/z_t_R, 305.24814_0.69

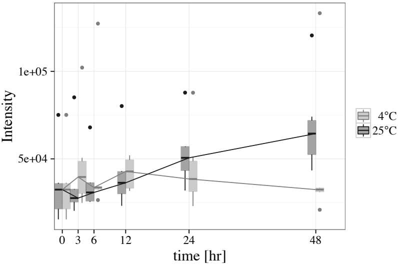

HILICneg: m/z_t_R, 417.08284_4.85

HILICneg: m/z_t_R, 311.29508_0.67

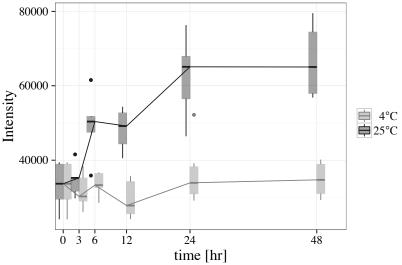

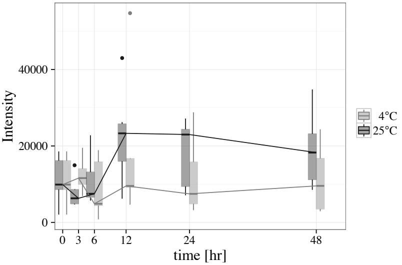

HILICneg: m/z_t_R, 296.03778_4.92

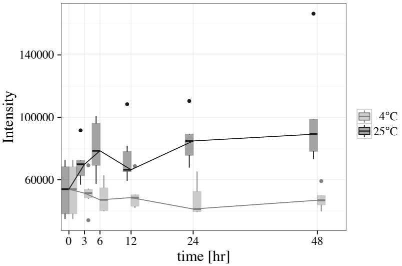

HILICneg: m/z_t_R, 133.01417_6.02

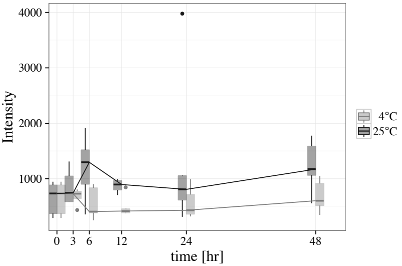

HILICneg: m/z_t_R, 202.05102_2.73

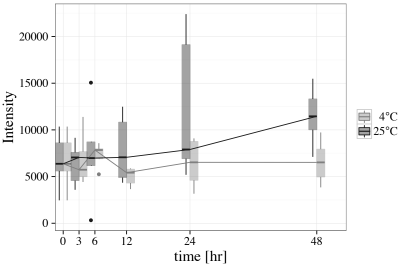

HILICneg: m/z_t_R, 299.25878_0.57

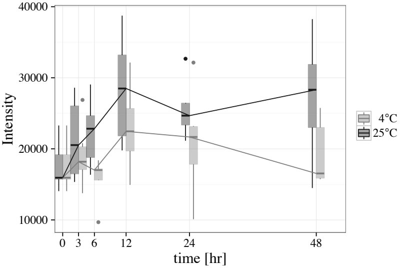

HILICneg: m/z_t_R, 557.45768_0.67

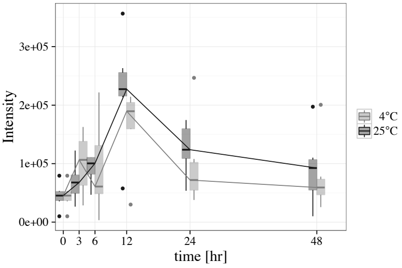

HILICneg: m/z_t_R, 253.21712_0.74

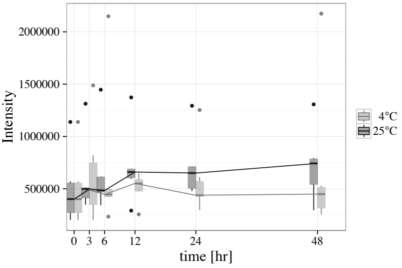

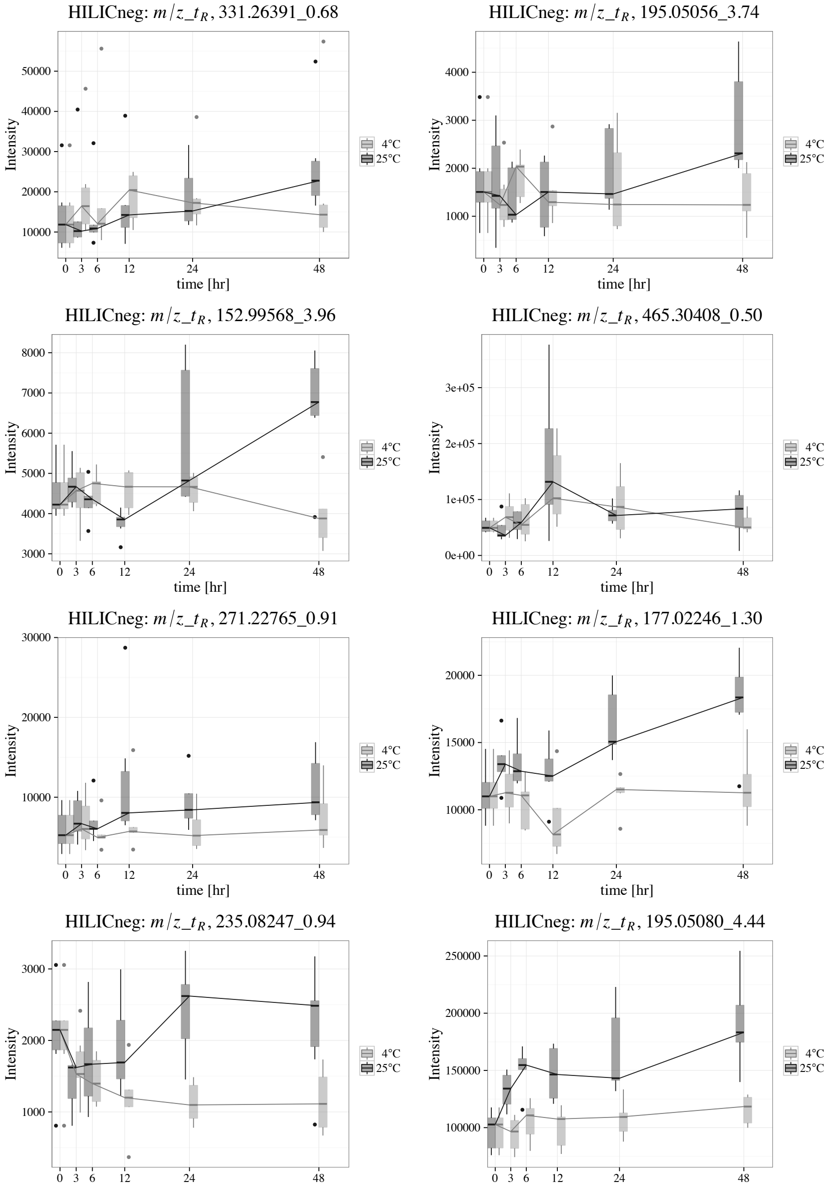

HILICneg: m/z_t_R, 331.26391_0.68
Intensity
10000
20000
30000
40000
50000
time [hr]
0
3
6
12
24
48
4°C
25°C
HILICneg: m/z_t_R, 195.05056_3.74
Intensity
1000
2000
3000
4000
time [hr]
0
3
6
12
24
48
4°C
25°C
HILICneg: m/z_t_R, 152.99568_3.96
Intensity
3000
4000
5000
6000
7000
8000
time [hr]
0
3
6
12
48
4°C
25°C
HILICneg: m/z_t_R, 465.30408_0.50
Intensity
0e+00
1e+05
2e+05
3e+05
time [hr]
0
3
6
12
24
48
4°C
25°C
HILICneg: m/z_t_R, 271.22765_0.91
Intensity
10000
20000
30000
time [hr]
0
3
6
12
24
48
4°C
25°C
HILICneg: m/z_t_R, 177.02246_1.30
Intensity
10000
15000
20000
time [hr]
0
3
6
12
24
48
4°C
25°C
HILICneg: m/z_t_R, 235.08247_0.94
Intensity
1000
2000
3000
0
3
6
12
24
48
4°C
25°C
HILICneg: m/z_t_R, 195.05080_4.44
Intensity
100000
150000
200000
250000
0
3
6
12
24
48
4°C
25°C

time [hr]

time [hr]

HILICneg: m/z_t_R, 176.09258_3.93

HILICneg: m/z_t_R, 227.20148_0.76

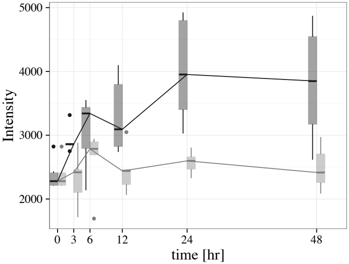

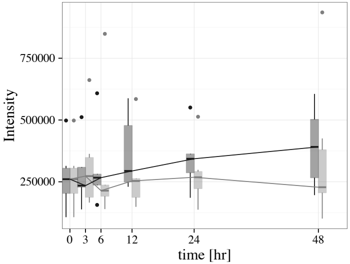

HILICneg: m/z_t_R, 173.00896_5.48

HILICneg: m/z_t_R, 217.10782_3.36

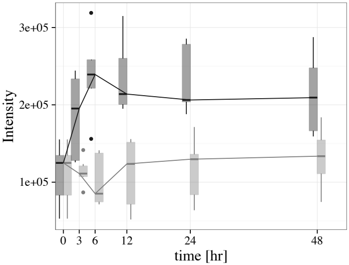

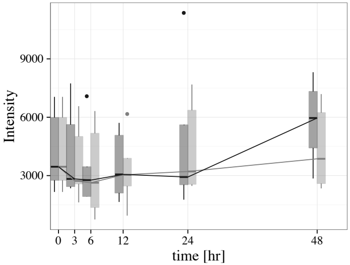

HILICneg: m/z_t_R, 343.28499_0.71

HILICneg: m/z_t_R, 209.04529_1.41

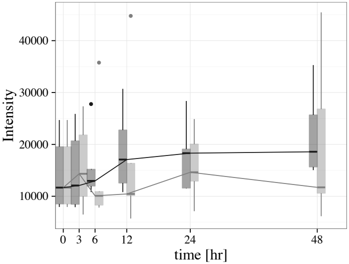

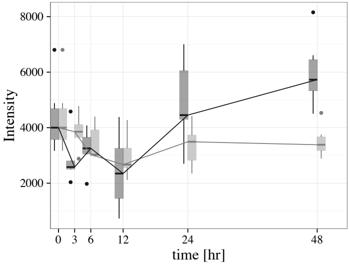

HILICneg: m/z_t_R, 202.05095_1.42

HILICneg: m/z_t_R, 255.23276_0.72

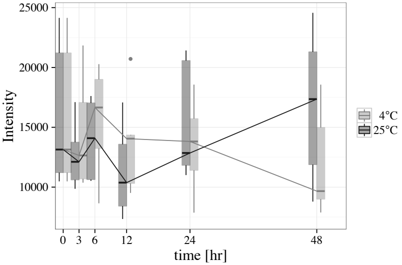

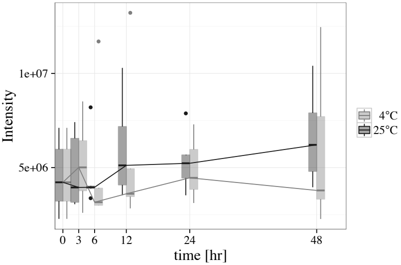

HILICneg: m/z_t_R, 199.17018_0.80

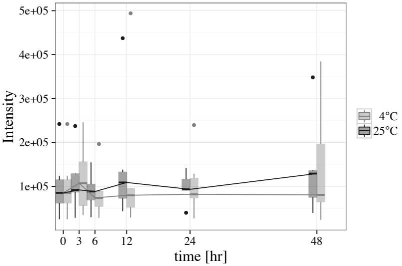

HILICneg: m/z_t_R, 327.23272_0.63

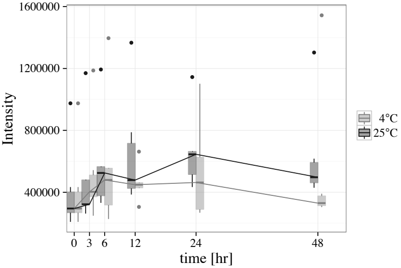

HILICneg: m/z_t_R, 146.02778_1.40

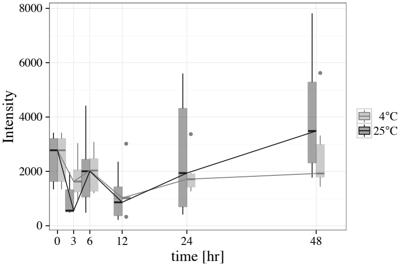

HILICneg: m/z_t_R, 279.23272_0.71

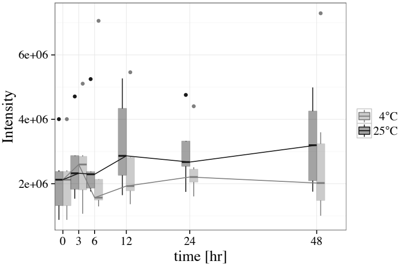

HILICneg: m/z_t_R, 460.09977_4.88

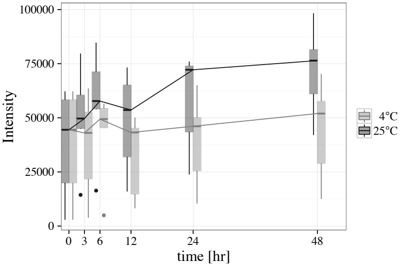

HILICneg: m/z_t_R, 176.03847_2.73

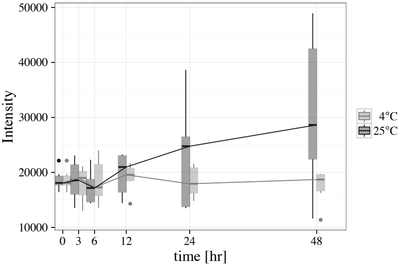

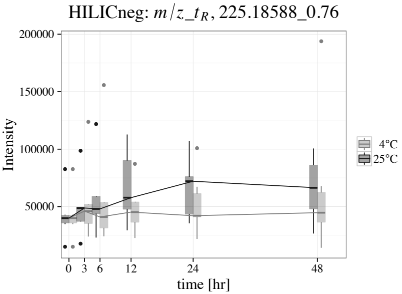
HILICneg: m/z_t_R, 225.18588_0.76
Intensity
time [hr]
4°C
25°C

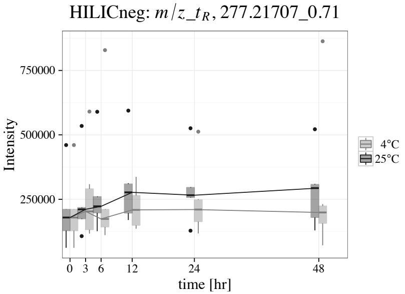
HILICneg: m/z_t_R, 277.21707_0.71
Intensity
time [hr]
4°C
25°C

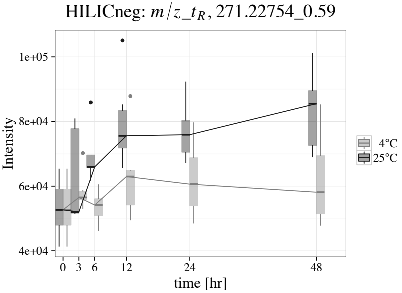
HILICneg: m/z_t_R, 271.22754_0.59
Intensity
time [hr]
4°C
25°C

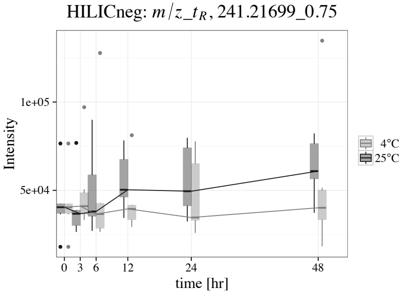
HILICneg: m/z_t_R, 241.21699_0.75
Intensity
time [hr]
4°C
25°C

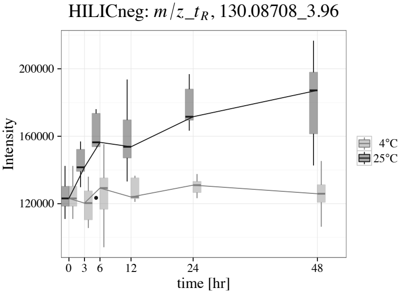
HILICneg: m/z_t_R, 130.08708_3.96
Intensity
time [hr]
4°C
25°C

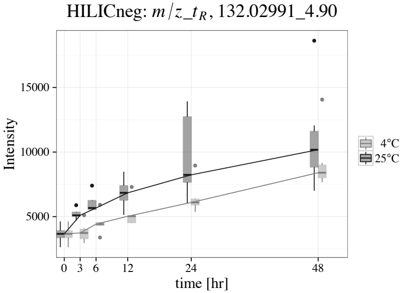
HILICneg: m/z_t_R, 132.02991_4.90
Intensity
time [hr]
4°C
25°C

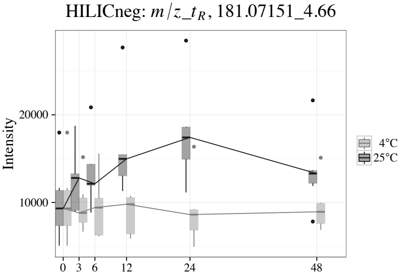
HILICneg: m/z_t_R, 181.07151_4.66
Intensity
4°C
25°C

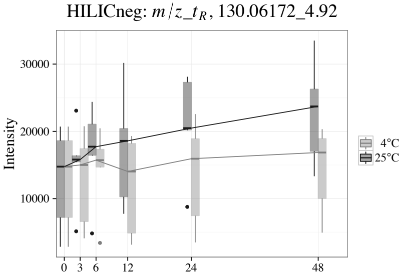
HILICneg: m/z_t_R, 130.06172_4.92
Intensity
4°C
25°C

time [hr]

time [hr]

HILICneg: m/z_t_R, 146.04566_3.82

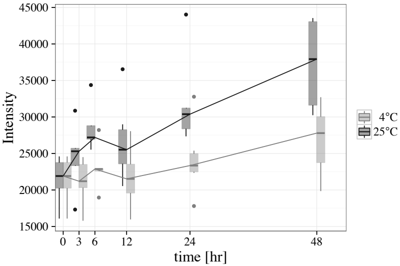

HILICneg: m/z_t_R, 290.08789_4.34

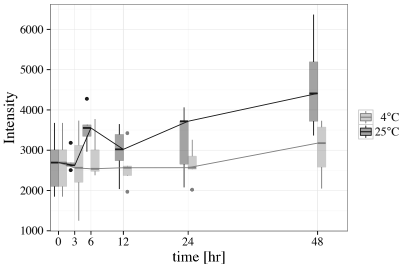

HILICneg: m/z_t_R, 480.30940_2.98

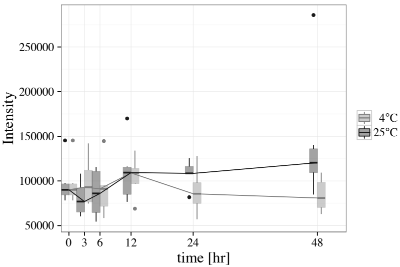

HILICneg: m/z_t_R, 115.00354_0.92

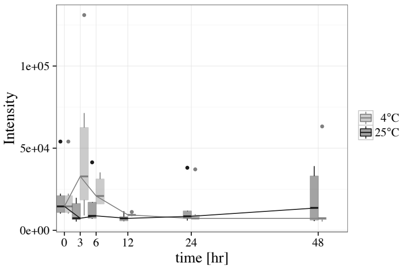

HILICneg: m/z_t_R, 857.51899_0.94

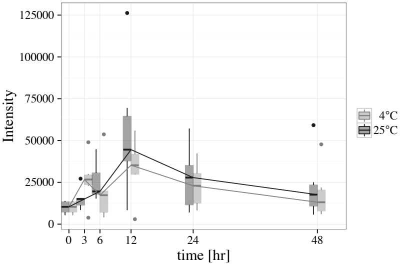

HILICneg: m/z_t_R, 523.43657_0.91

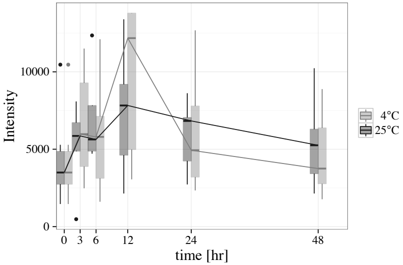

HILICneg: m/z_t_R, 267.23282_0.71

HILICneg: m/z_t_R, 105.01943_4.00

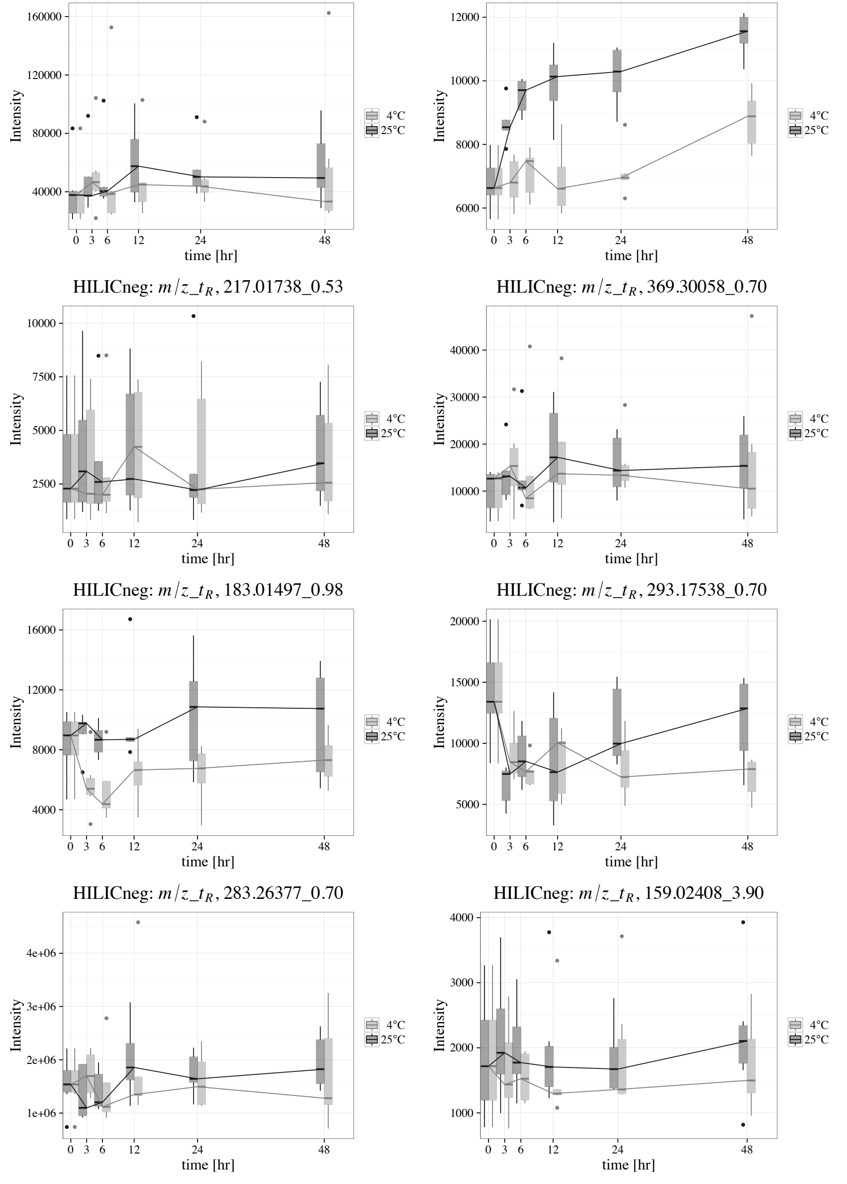
HILICneg: m/z_tR, 217.01738_0.53
HILICneg: m/z_tR, 369.30058_0.70
HILICneg: m/z_tR, 183.01497_0.98
HILICneg: m/z_tR, 293.17538_0.70
HILICneg: m/z_tR, 283.26377_0.70
HILICneg: m/z_tR, 159.02408_3.90
Intensity
time [hr]
4°C
25°C

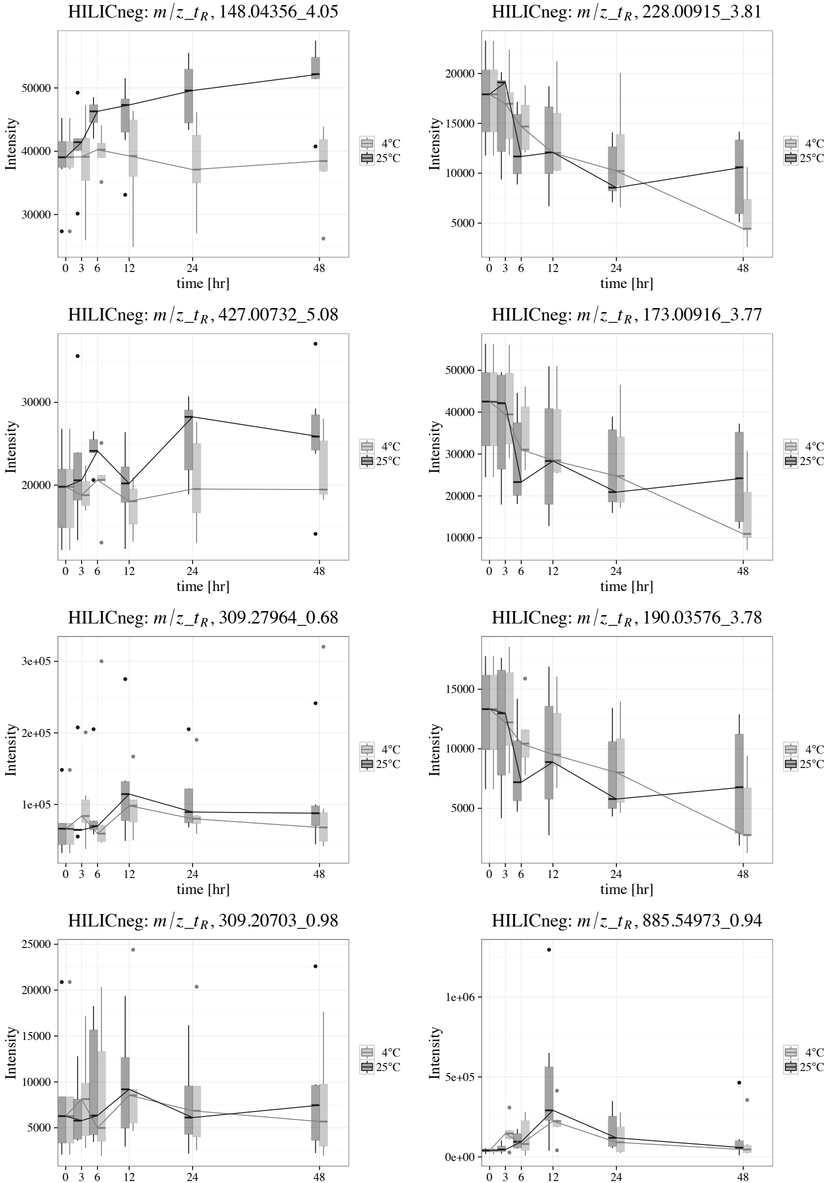
HILICneg: m/z_t_R, 148.04356_4.05
Intensity
time [hr]
4°C
25°C
HILICneg: m/z_t_R, 228.00915_3.81
Intensity
time [hr]
4°C
25°C
HILICneg: m/z_t_R, 427.00732_5.08
Intensity
time [hr]
4°C
25°C
HILICneg: m/z_t_R, 173.00916_3.77
Intensity
time [hr]
4°C
25°C
HILICneg: m/z_t_R, 309.27964_0.68
Intensity
time [hr]
4°C
25°C
HILICneg: m/z_t_R, 190.03576_3.78
Intensity
time [hr]
4°C
25°C
HILICneg: m/z_t_R, 309.20703_0.98
Intensity
4°C
25°C
HILICneg: m/z_t_R, 885.54973_0.94
Intensity
4°C
25°C

time [hr]

time [hr]

HILICneg: m/z_t_R, 391.28541_0.95

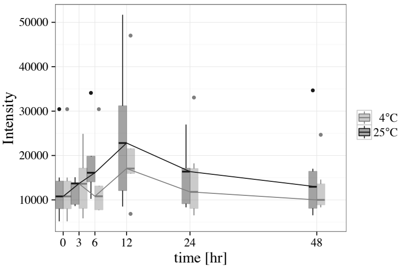

HILICneg: m/z_t_R, 205.03541_1.76

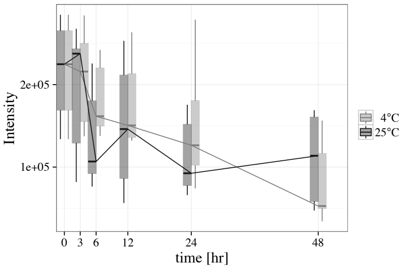

HILICneg: m/z_t_R, 173.00916_1.78

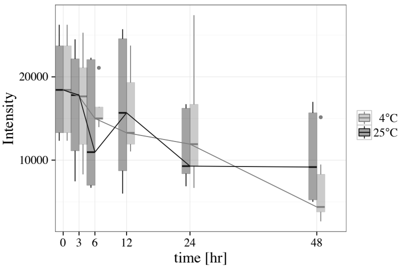

HILICneg: m/z_t_R, 173.05666_3.98

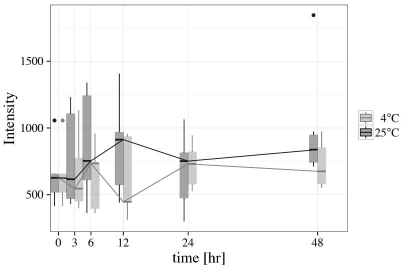

HILICneg: m/z_t_R, 269.24840_0.71

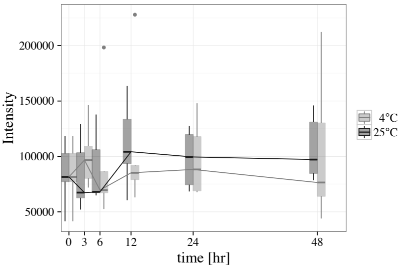

HILICneg: m/z_t_R, 378.98294_0.95

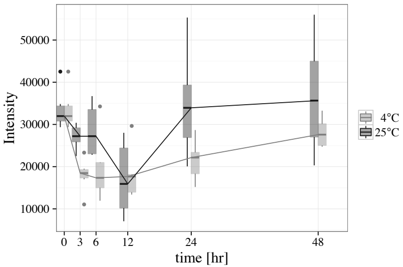

HILICneg: m/z_t_R, 861.54965_0.94

HILICneg: m/z_t_R, 451.34271_0.89

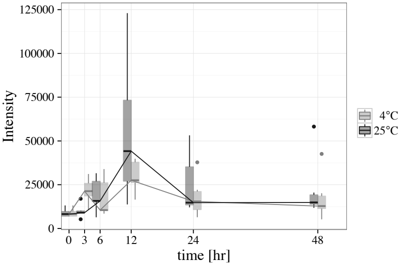

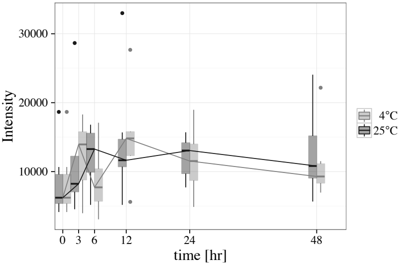

HILICneg: m/z_t_R, 104.03580_5.04

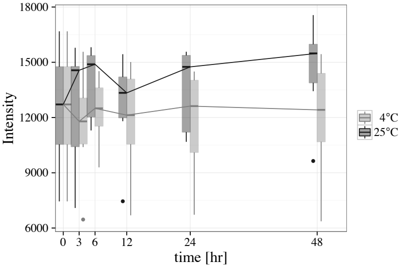

HILICneg: m/z_t_R, 130.08714_4.06

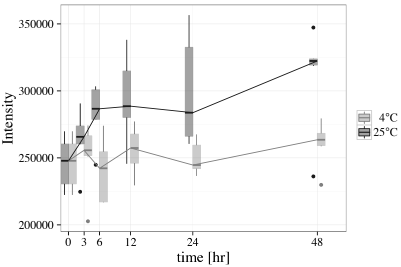

HILICneg: m/z_t_R, 226.99639_3.83

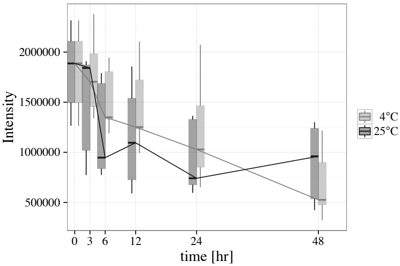

HILICneg: m/z_t_R, 281.24832_0.70

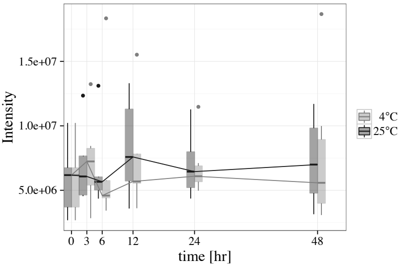

HILICneg: m/z_t_R, 307.26416_0.69

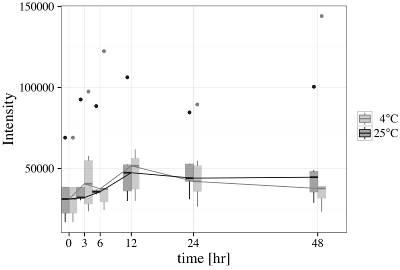

HILICneg: m/z_t_R, 329.24814_0.68

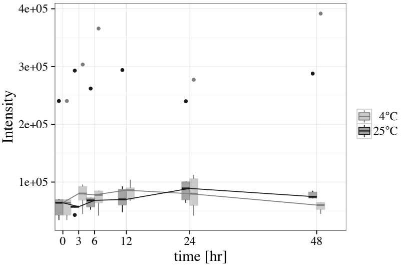

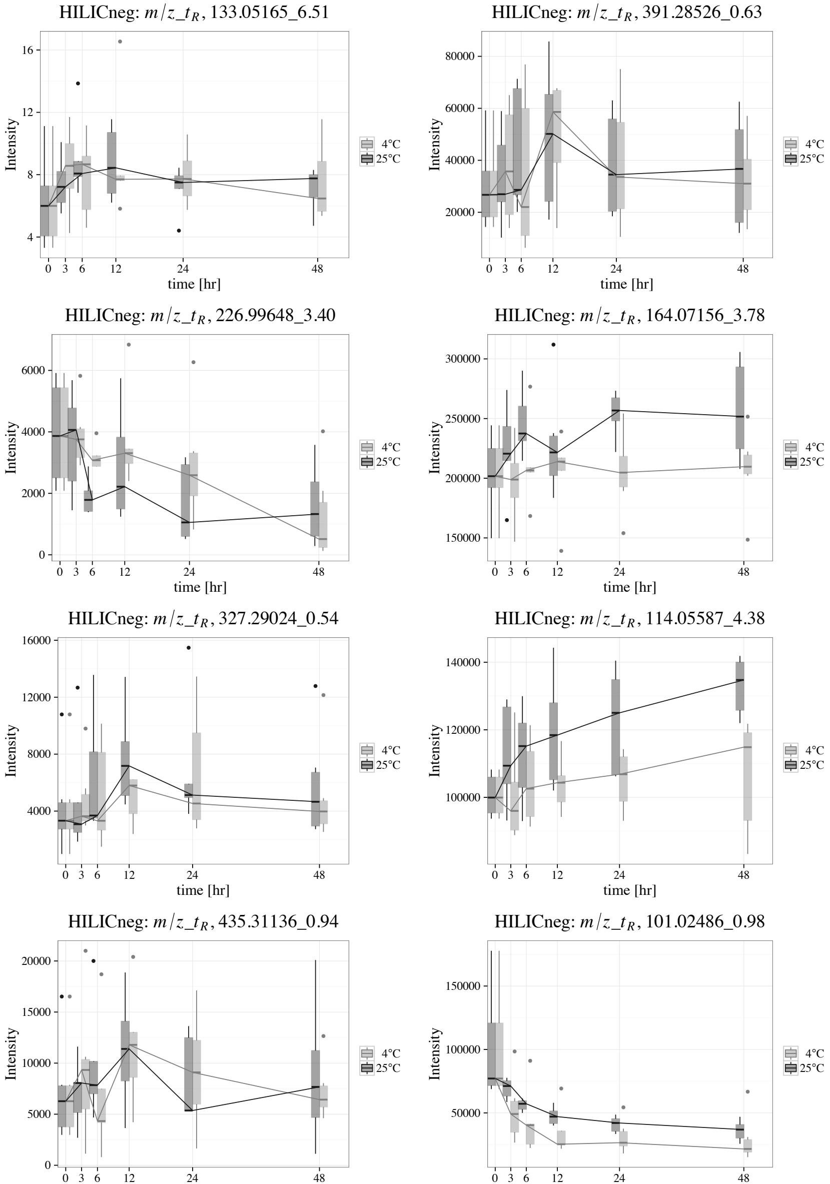

HILICneg: m/z_t_R, 133.05165_6.51
HILICneg: m/z_t_R, 391.28526_0.63
HILICneg: m/z_t_R, 226.99648_3.40
HILICneg: m/z_t_R, 164.07156_3.78
HILICneg: m/z_t_R, 327.29024_0.54
HILICneg: m/z_t_R, 114.05587_4.38
HILICneg: m/z_t_R, 435.31136_0.94
HILICneg: m/z_t_R, 101.02486_0.98
Intensity
time [hr]
0
3
6
12
24
48
4°C
25°C

time [hr]

time [hr]

HILICneg: m/z_t_R, 159.10250_3.44

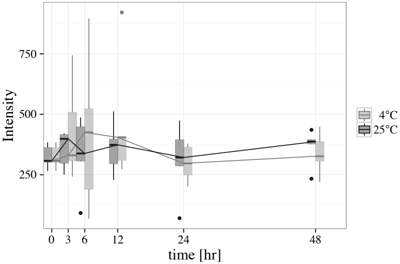

HILICneg: m/z_t_R, 434.97035_5.09

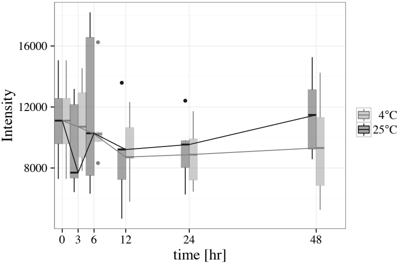

HILICneg: m/z_t_R, 238.08496_4.03

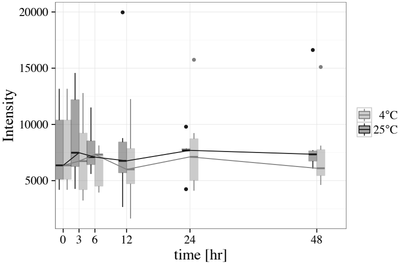

HILICneg: m/z_t_R, 165.05549_0.97

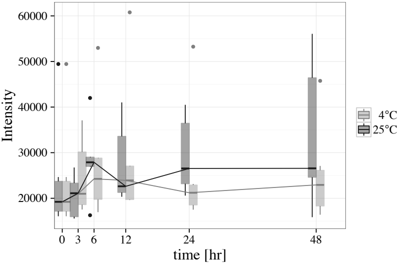

HILICneg: m/z_t_R, 479.33761_0.97

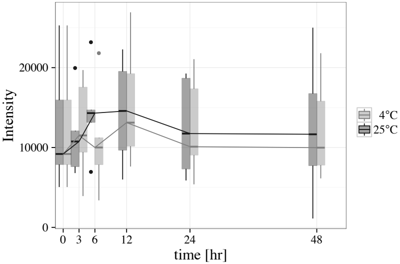

HILICneg: m/z_t_R, 129.05547_0.84

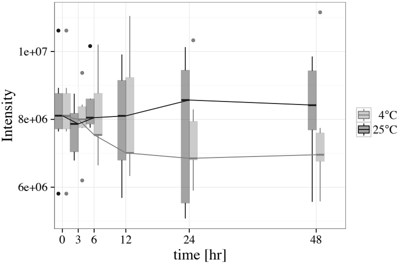

HILICneg: m/z_t_R, 169.12326_0.86

HILICneg: m/z_t_R, 213.14959_0.78

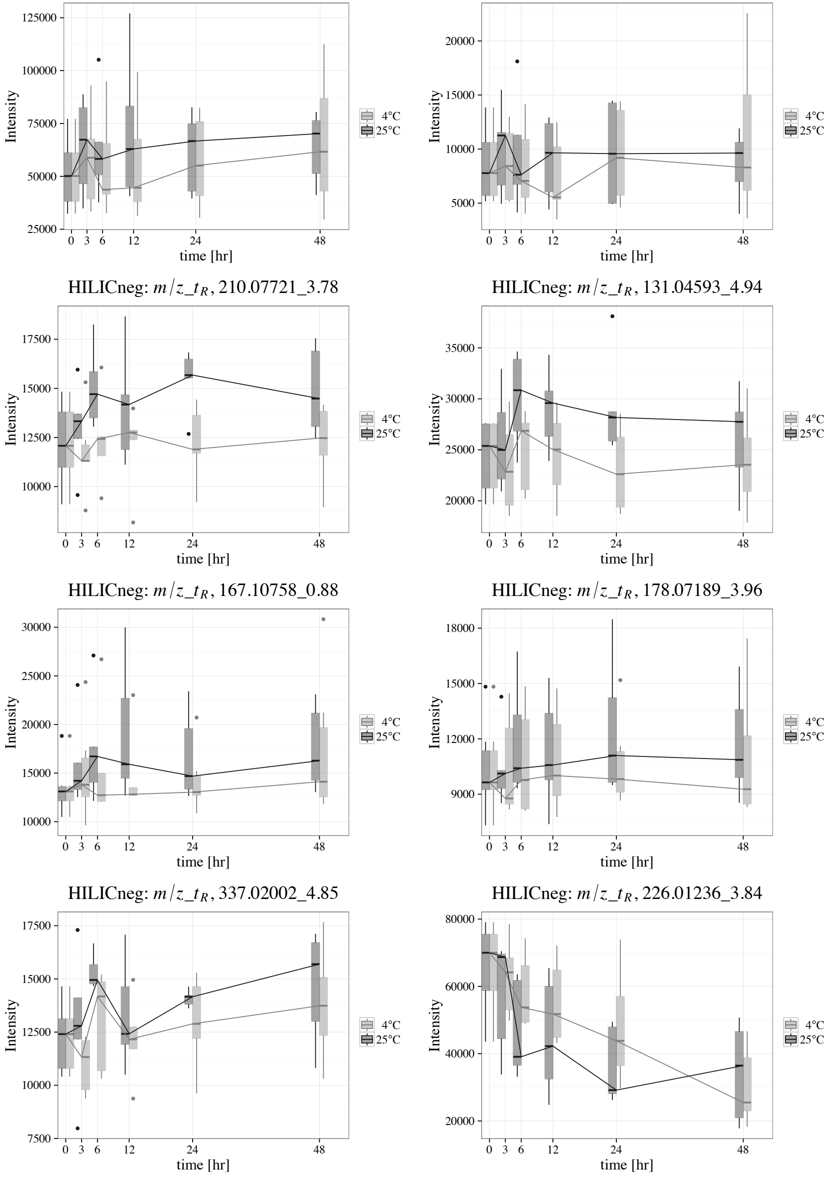
Intensity
time [hr]
4°C
25°C
HILICneg: m/z_t_R, 210.07721_3.78
HILICneg: m/z_t_R, 131.04593_4.94
HILICneg: m/z_t_R, 167.10758_0.88
HILICneg: m/z_t_R, 178.07189_3.96
HILICneg: m/z_t_R, 337.02002_4.85
HILICneg: m/z_t_R, 226.01236_3.84

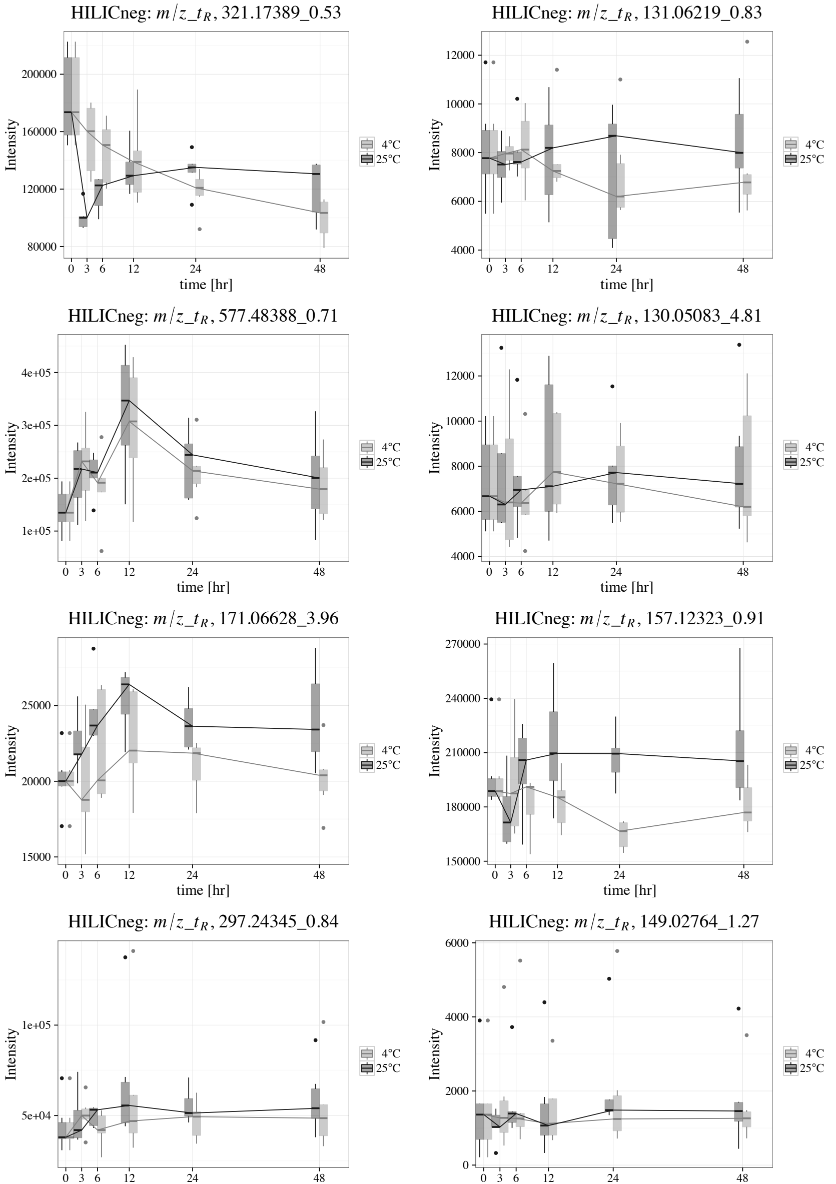
HILICneg: m/z_t_R, 321.17389_0.53
HILICneg: m/z_t_R, 131.06219_0.83
HILICneg: m/z_t_R, 577.48388_0.71
HILICneg: m/z_t_R, 130.05083_4.81
HILICneg: m/z_t_R, 171.06628_3.96
HILICneg: m/z_t_R, 157.12323_0.91
HILICneg: m/z_t_R, 297.24345_0.84
HILICneg: m/z_t_R, 149.02764_1.27
Intensity
time [hr]
4°C
25°C

time [hr]

time [hr]

HILICneg: m/z_t_R, 206.99675_0.62

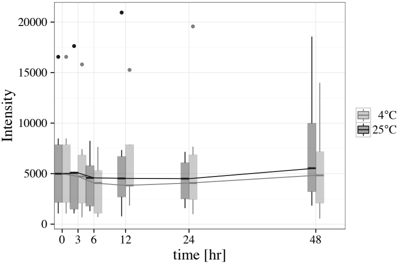

HILICneg: m/z_t_R, 331.19137_0.98

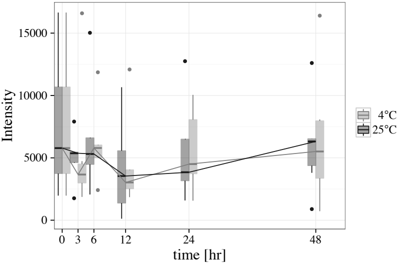

HILICneg: m/z_t_R, 183.13888_0.87

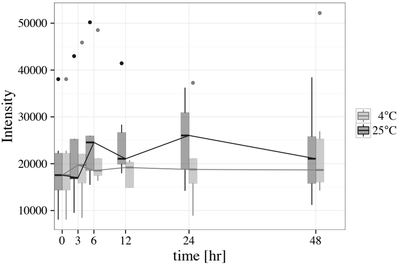

HILICneg: m/z_t_R, 311.22282_0.98

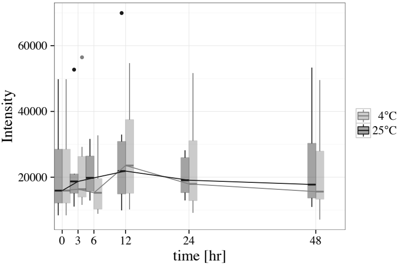

HILICneg: m/z_t_R, 197.15454_0.78

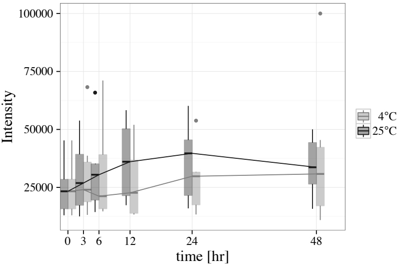

HILICneg: m/z_t_R, 154.04529_3.96

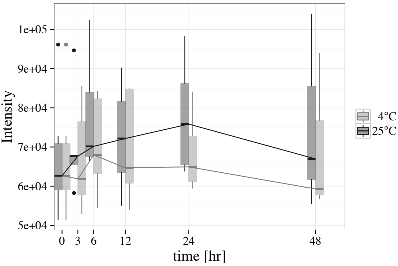

HILICneg: m/z_t_R, 180.06641_4.34

HILICneg: m/z_t_R, 137.02387_3.41

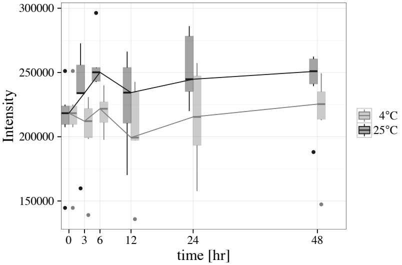

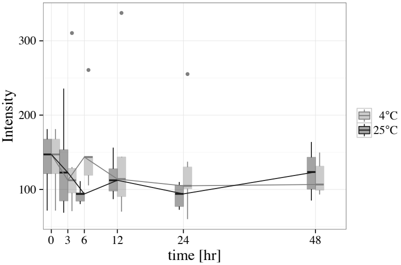

HILICneg: m/z_t_R, 162.07691_4.29

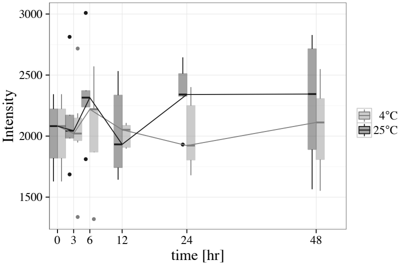

HILICneg: m/z_t_R, 115.03993_0.94

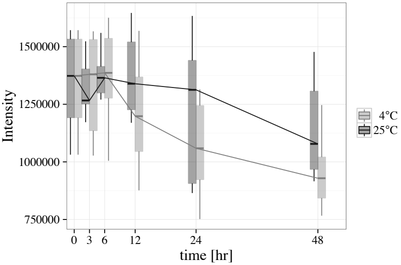

HILICneg: m/z_t_R, 201.03482_4.04

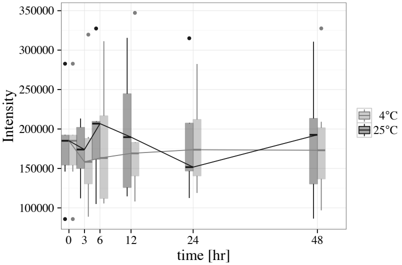

HILICneg: m/z_t_R, 116.07154_4.29

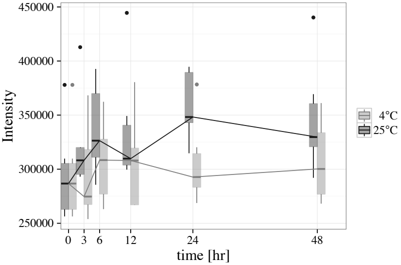

HILICneg: m/z_t_R, 293.17922_0.50

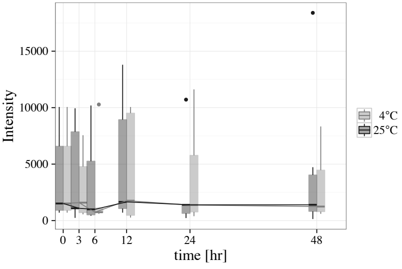

HILICneg: m/z_t_R, 191.05594_4.12

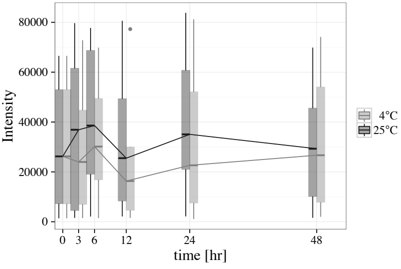

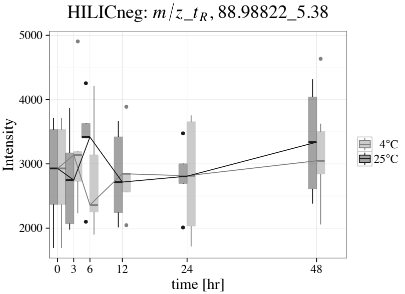
HILICneg: m/z_t_R, 88.98822_5.38
Intensity
2000
3000
4000
5000
0 3 6 12 24 48
time [hr]
4°C
25°C

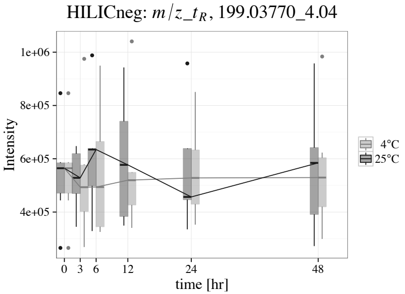
HILICneg: m/z_t_R, 199.03770_4.04
Intensity
4e+05
6e+05
8e+05
1e+06
0 3 6 12 24 48
time [hr]
4°C
25°C

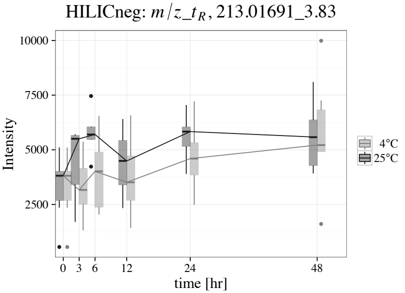
HILICneg: m/z_t_R, 213.01691_3.83
Intensity
2500
5000
7500
10000
0 3 6 12 24 48
time [hr]
4°C
25°C

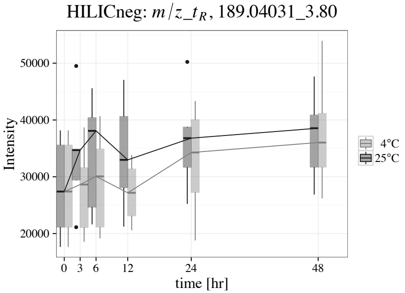
HILICneg: m/z_t_R, 189.04031_3.80
Intensity
20000
30000
40000
50000
0 3 6 12 24 48
time [hr]
4°C
25°C

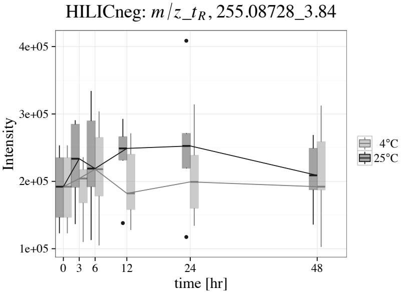
HILICneg: m/z_t_R, 255.08728_3.84
Intensity
1e+05
2e+05
3e+05
4e+05
0 3 6 12 24 48
time [hr]
4°C
25°C

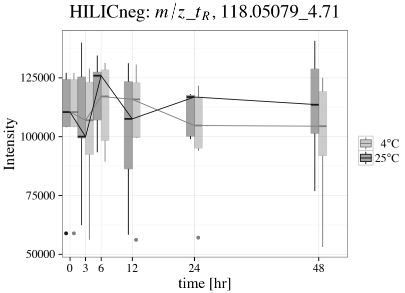
HILICneg: m/z_t_R, 118.05079_4.71
Intensity
50000
75000
100000
125000
0 3 6 12 24 48
time [hr]
4°C
25°C

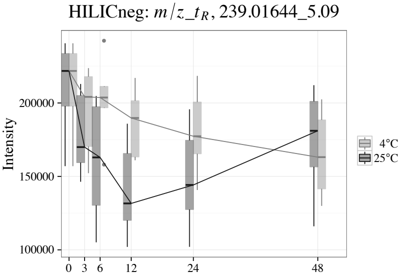
HILICneg: m/z_t_R, 239.01644_5.09
Intensity
100000
150000
200000
0 3 6 12 24 48
4°C
25°C

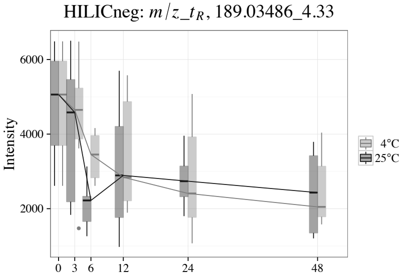
HILICneg: m/z_t_R, 189.03486_4.33
Intensity
2000
4000
6000
0 3 6 12 24 48
4°C
25°C

time [hr]

time [hr]

HILICneg: m/z_t_R, 135.04496_0.97

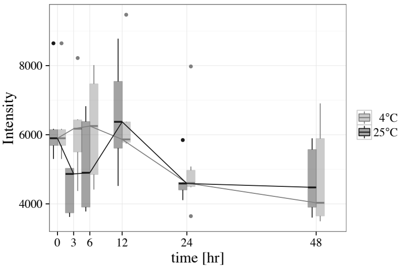

HILICneg: m/z_t_R, 349.15221_4.33

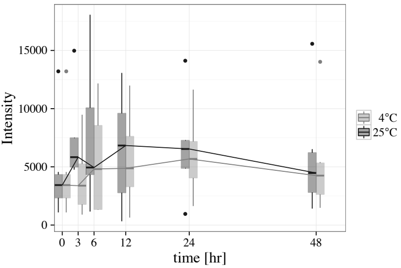

HILICneg: m/z_t_R, 507.36865_0.93

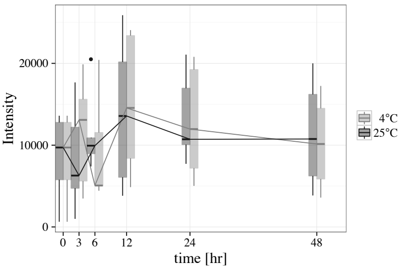

HILICneg: m/z_t_R, 171.07732_1.35

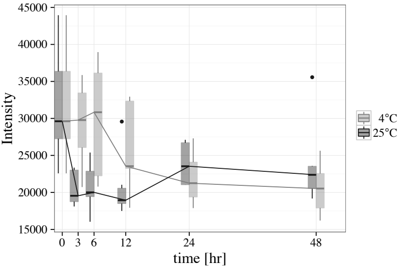

HILICneg: m/z_t_R, 826.56068_0.99

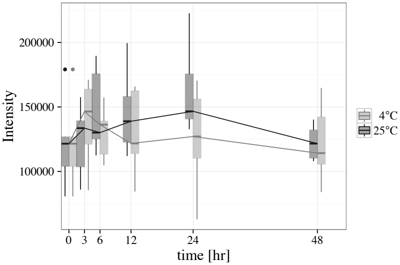

HILICneg: m/z_t_R, 134.02793_4.04

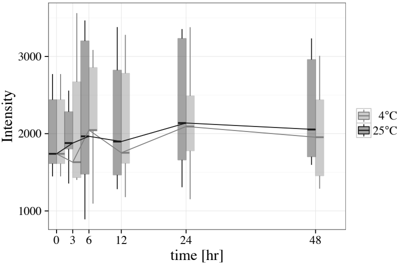

HILICneg: m/z_t_R, 180.03347_4.00

HILICneg: m/z_t_R, 854.59106_0.99

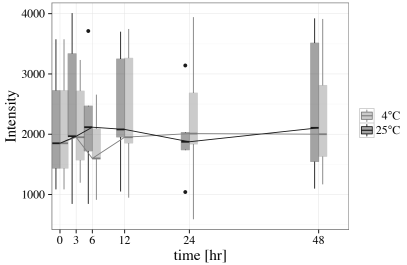

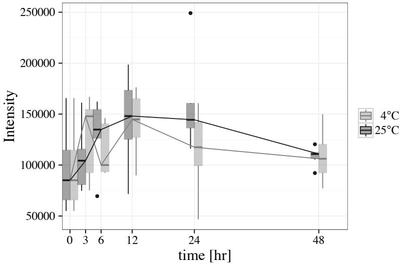

HILICneg: m/z_t_R, 141.09192_0.97

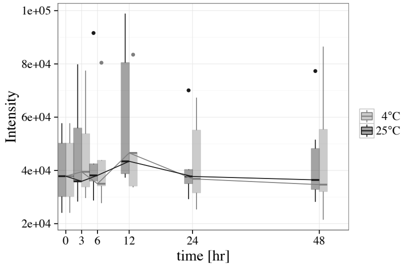

HILICneg: m/z_t_R, 149.02736_0.98

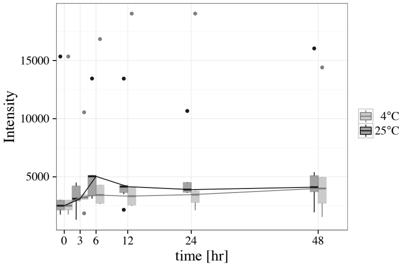

HILICneg: m/z_t_R, 595.49428_0.75

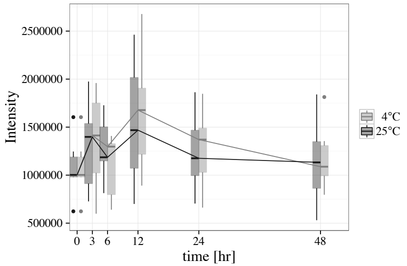

HILICneg: m/z_t_R, 397.20529_0.53

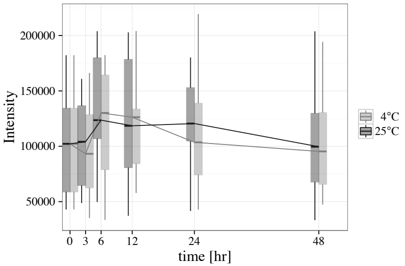

HILICneg: m/z_t_R, 223.17025_0.77

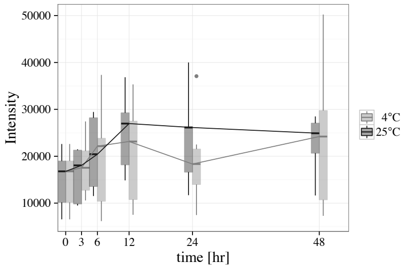

HILICneg: m/z_t_R, 195.13887_0.79

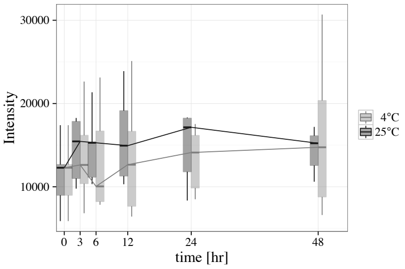

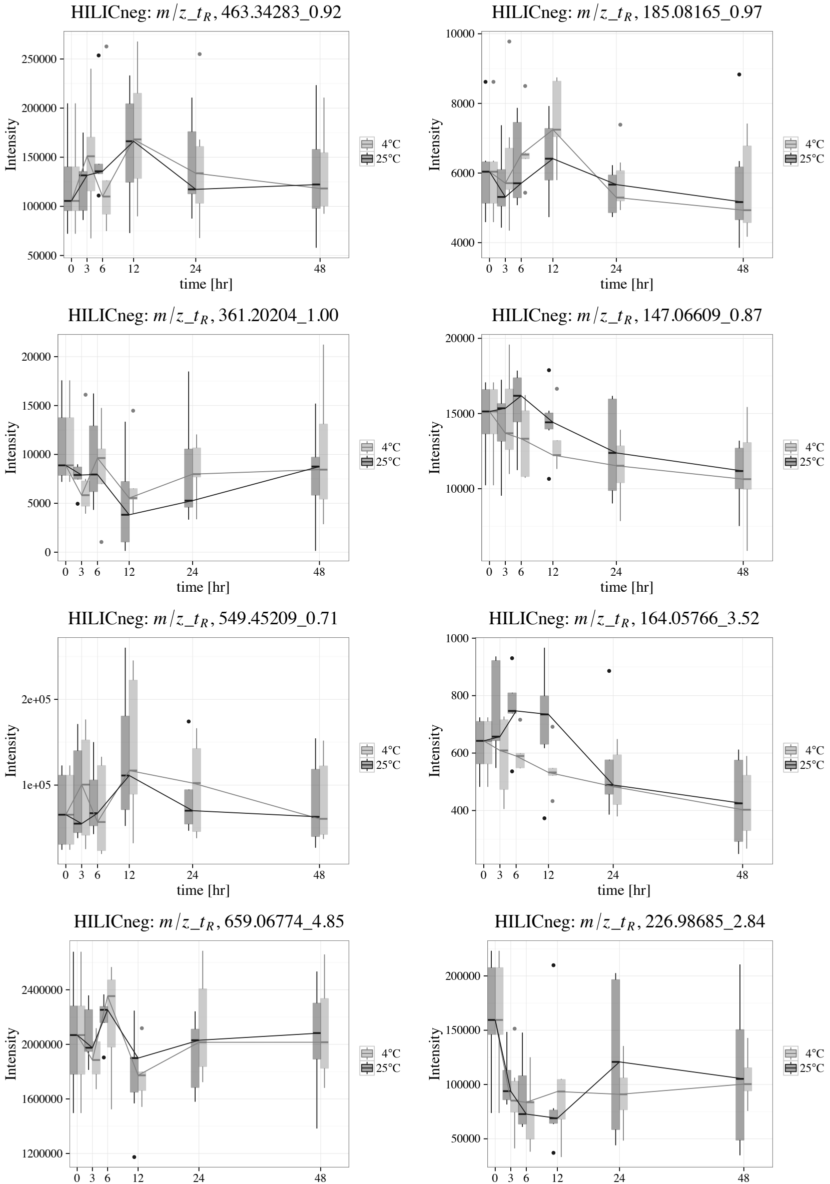
HILICneg: m/z_t_R, 463.34283_0.92
HILICneg: m/z_t_R, 185.08165_0.97
HILICneg: m/z_t_R, 361.20204_1.00
HILICneg: m/z_t_R, 147.06609_0.87
HILICneg: m/z_t_R, 549.45209_0.71
HILICneg: m/z_t_R, 164.05766_3.52
HILICneg: m/z_t_R, 659.06774_4.85
HILICneg: m/z_t_R, 226.98685_2.84
Intensity
time [hr]
0
3
6
12
24
48
4°C
25°C

time [hr]

time [hr]

HILICneg: m/z_t_R, 329.03017_4.85

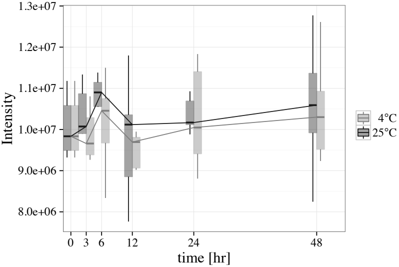

HILICneg: m/z_t_R, 295.26378_0.68

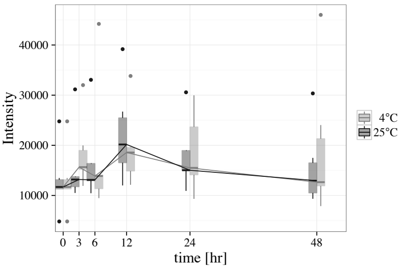

HILICneg: m/z_t_R, 267.12358_0.97

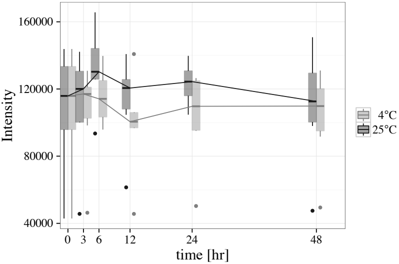

HILICneg: m/z_t_R, 154.05074_0.94

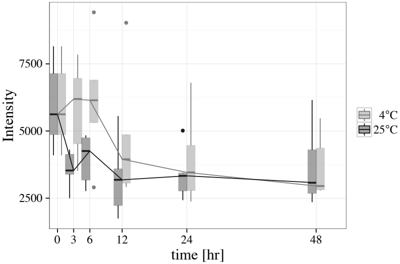

HILICneg: m/z_t_R, 145.08684_4.60

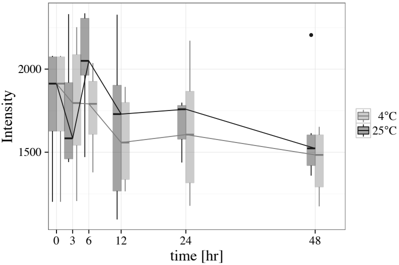

HILICneg: m/z_t_R, 255.06199_0.89

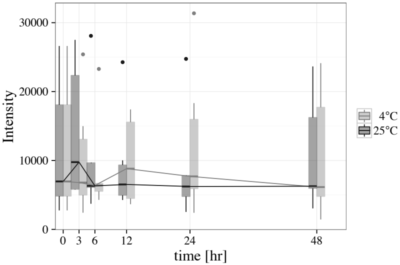

HILICneg: m/z_t_R, 397.36874_0.68

HILICneg: m/z_t_R, 349.20532_0.52

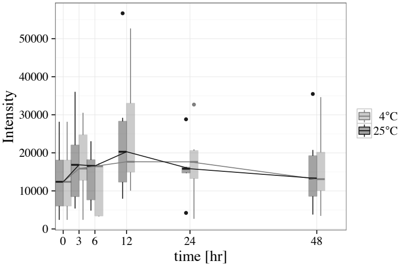

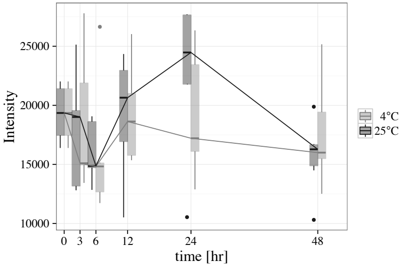

HILICneg: m/z_t_R, 187.00683_0.57

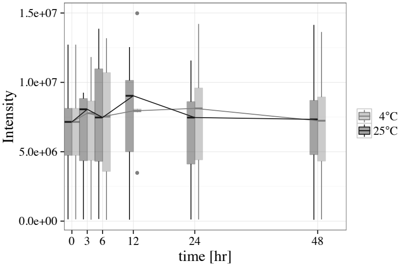

HILICneg: m/z_t_R, 241.15556_1.42

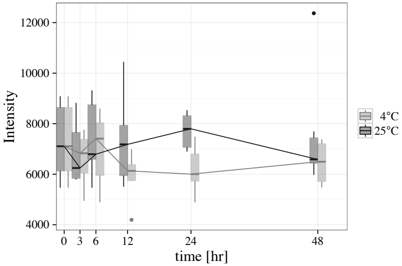

HILICneg: m/z_t_R, 266.01770_0.89

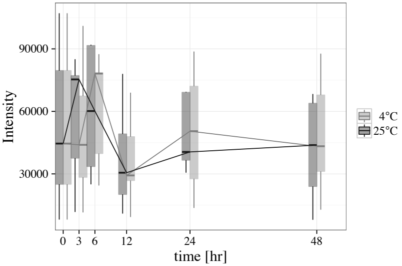

HILICneg: m/z_t_R, 241.18071_0.72

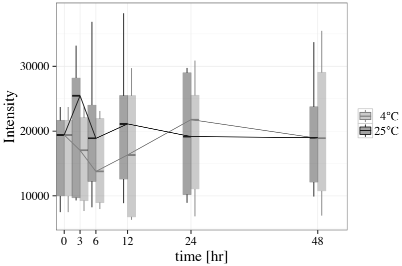

HILICneg: m/z_t_R, 265.14773_0.52

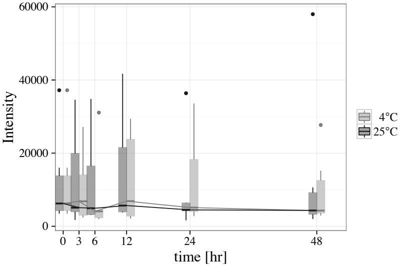

HILICneg: m/z_t_R, 199.00684_0.55

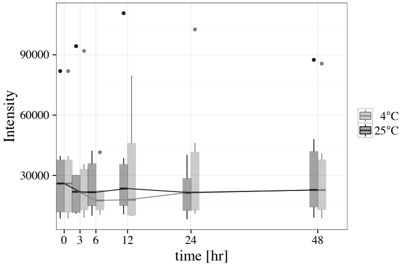

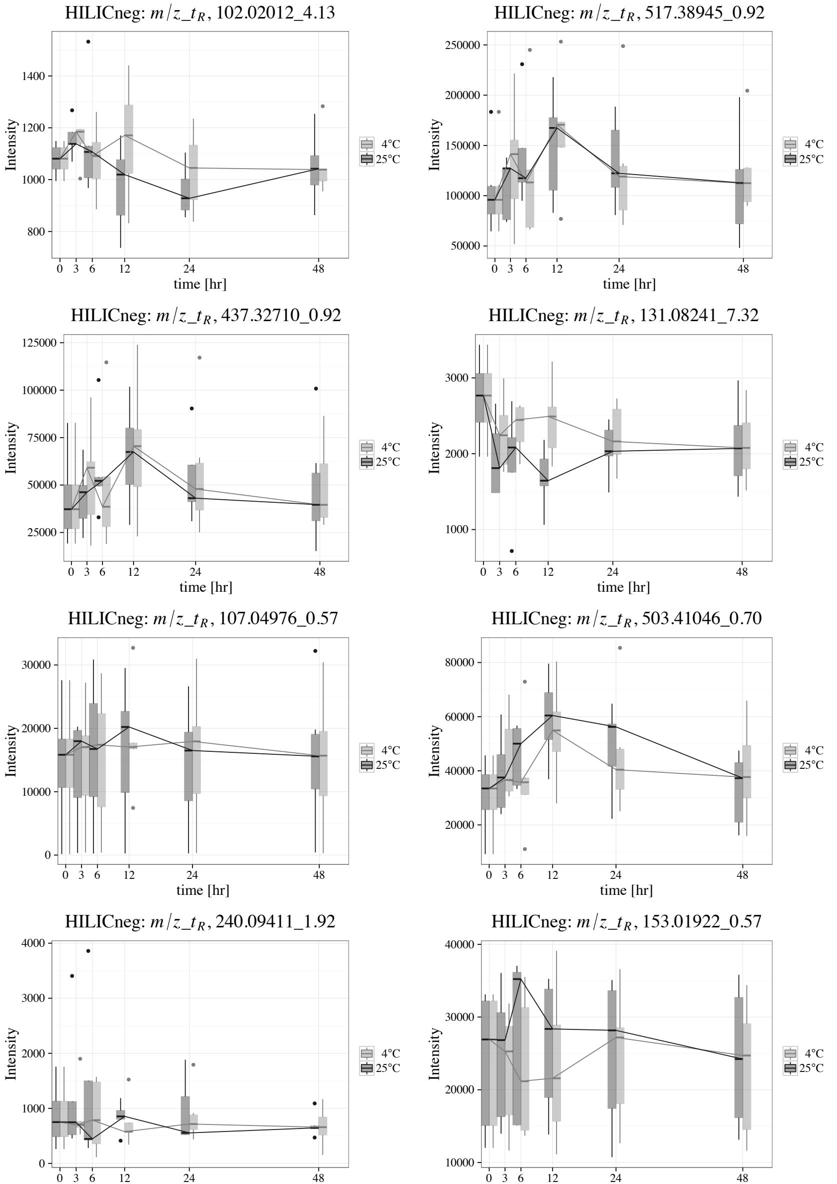
HILICneg: m/z_t_R, 102.02012_4.13
Intensity
800
1000
1200
1400
time [hr]
0 3 6 12 24 48
4°C
25°C
HILICneg: m/z_t_R, 517.38945_0.92
Intensity
50000
100000
150000
200000
250000
time [hr]
0 3 6 12 24 48
4°C
25°C
HILICneg: m/z_t_R, 437.32710_0.92
Intensity
25000
50000
75000
100000
125000
time [hr]
0 3 6 12 24 48
4°C
25°C
HILICneg: m/z_t_R, 131.08241_7.32
Intensity
1000
2000
3000
time [hr]
0 3 6 12 24 48
4°C
25°C
HILICneg: m/z_t_R, 107.04976_0.57
Intensity
0
10000
20000
30000
time [hr]
0 3 6 12 24 48
4°C
25°C
HILICneg: m/z_t_R, 503.41046_0.70
Intensity
20000
40000
60000
80000
time [hr]
0 3 6 12 24 48
4°C
25°C
HILICneg: m/z_t_R, 240.09411_1.92
Intensity
0
1000
2000
3000
4000
0 3 6 12 24 48
4°C
25°C
HILICneg: m/z_t_R, 153.01922_0.57
Intensity
10000
20000
30000
40000
0 3 6 12 24 48
4°C
25°C

time [hr]

time [hr]

HILICneg: m/z_t_R, 183.10242_0.97

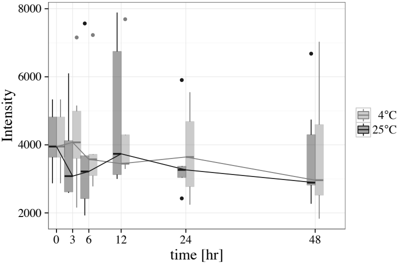

HILICneg: m/z_t_R, 311.16841_0.50

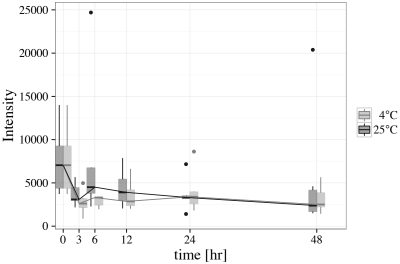

HILICneg: m/z_t_R, 117.05556_1.28

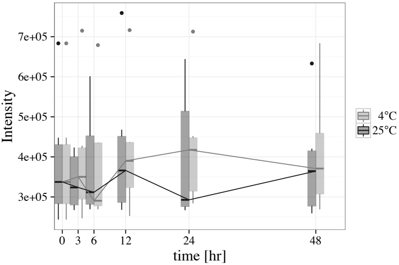

HILICneg: m/z_t_R, 163.07628_0.97

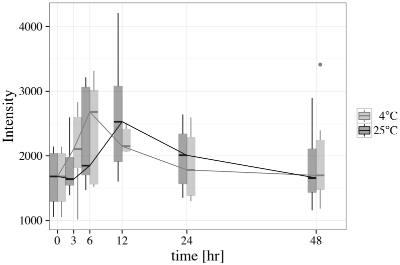

HILICneg: m/z_t_R, 431.17457_0.54

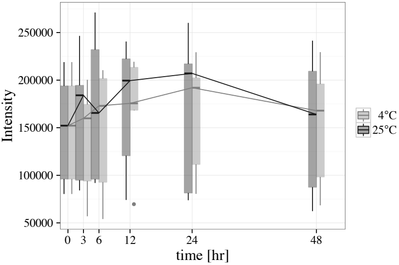

HILICneg: m/z_t_R, 239.09218_1.38

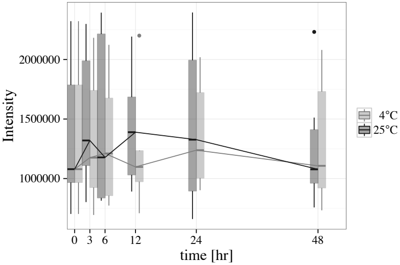

HILICneg: m/z_t_R, 201.02256_0.53

HILICneg: m/z_t_R, 429.30105_0.97

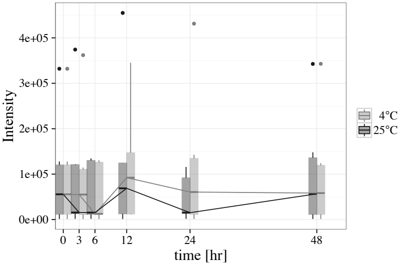

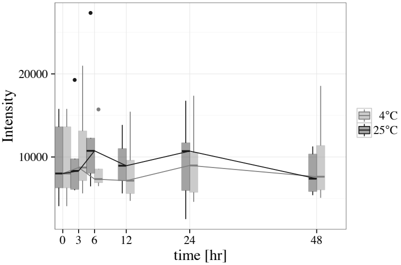

HILICneg: m/z_t_R, 173.08177_7.64

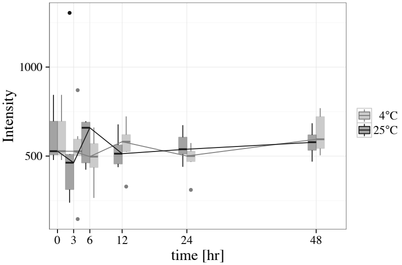

HILICneg: m/z_t_R, 102.05650_4.57

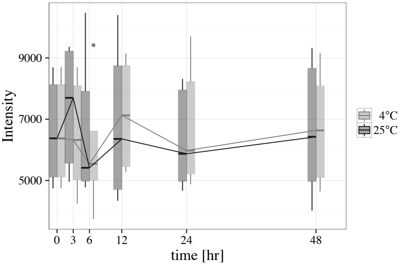

HILICneg: m/z_t_R, 295.22758_0.85

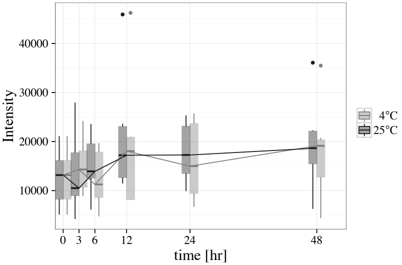

HILICneg: m/z_t_R, 367.15845_0.53

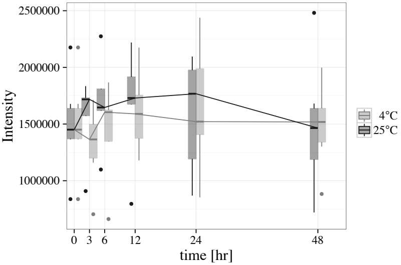

HILICneg: m/z_t_R, 213.02262_0.54

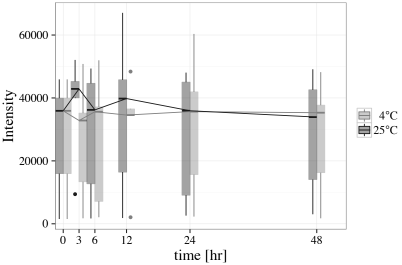

HILICneg: m/z_t_R, 103.04055_1.41

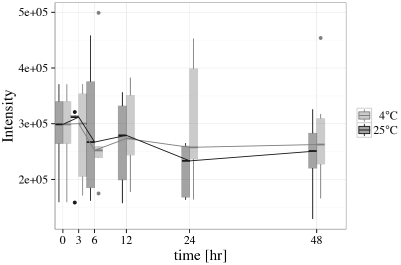

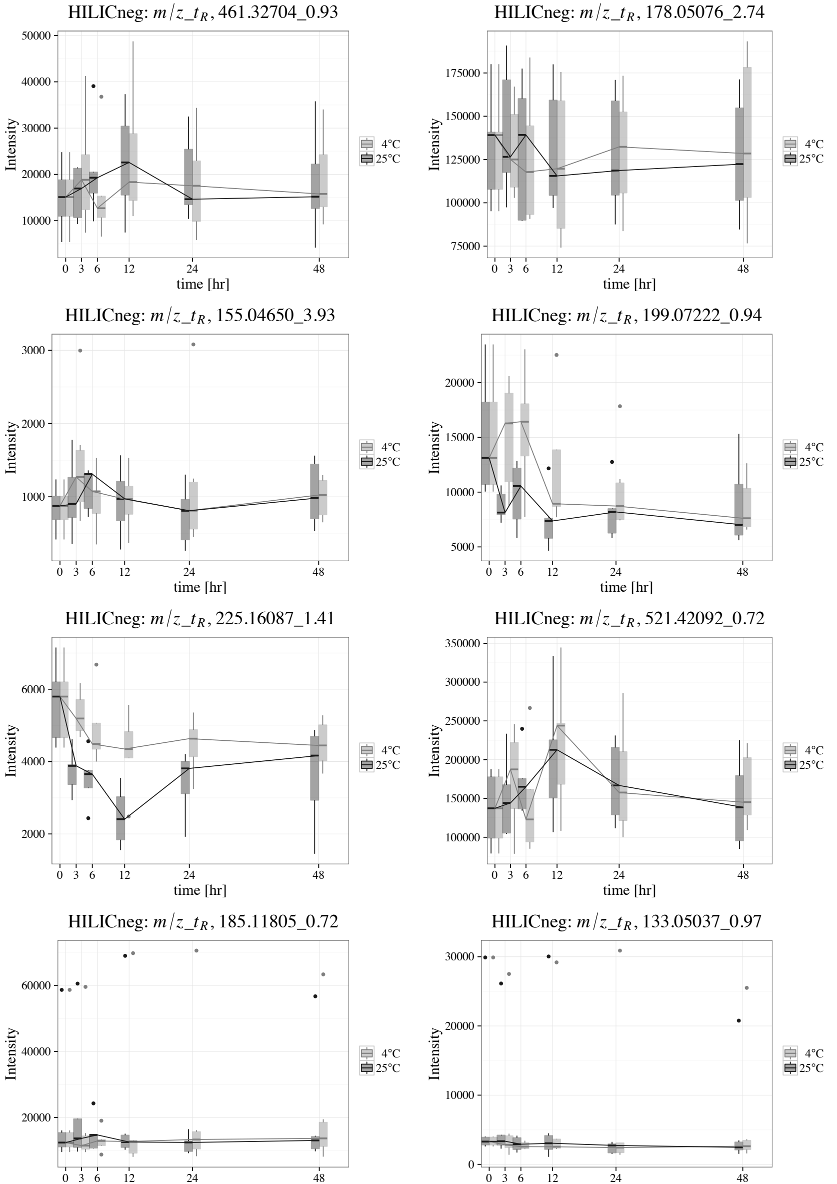

HILICneg: m/z_t_R, 461.32704_0.93
HILICneg: m/z_t_R, 178.05076_2.74
HILICneg: m/z_t_R, 155.04650_3.93
HILICneg: m/z_t_R, 199.07222_0.94
HILICneg: m/z_t_R, 225.16087_1.41
HILICneg: m/z_t_R, 521.42092_0.72
HILICneg: m/z_t_R, 185.11805_0.72
HILICneg: m/z_t_R, 133.05037_0.97
Intensity
time [hr]
0
3
6
12
24
48
4°C
25°C

time [hr]

time [hr]

HILICneg: m/z_t_R, 452.27806_3.01

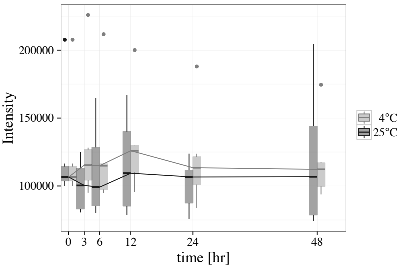

HILICneg: m/z_t_R, 159.10243_4.12

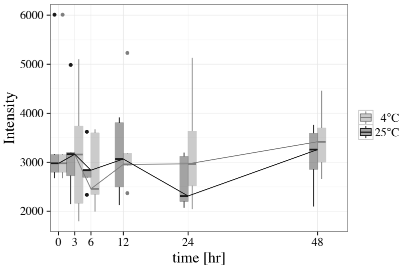

HILICneg: m/z_t_R, 763.56111_1.44

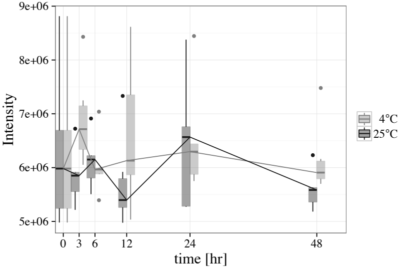

HILICneg: m/z_t_R, 239.16505_0.74

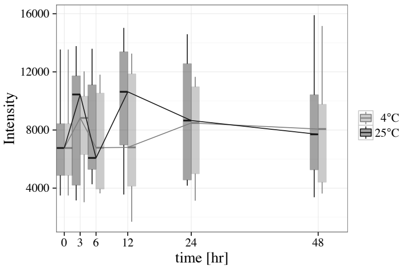

HILICneg: m/z_t_R, 187.11981_7.62

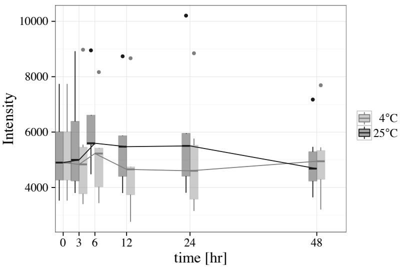

HILICneg: m/z_t_R, 129.02039_2.29

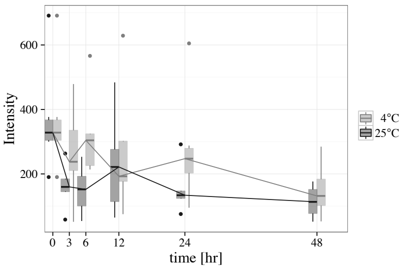

HILICneg: m/z_t_R, 395.18967_0.52

HILICneg: m/z_t_R, 237.14954_0.74

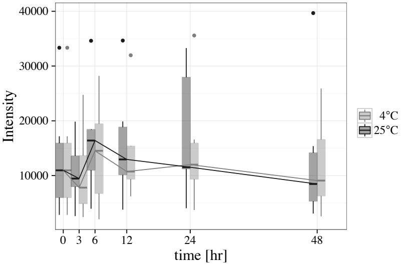

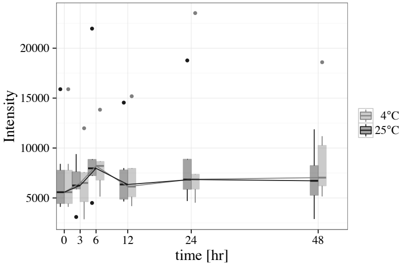

HILICneg: m/z_t_R, 157.08678_0.97

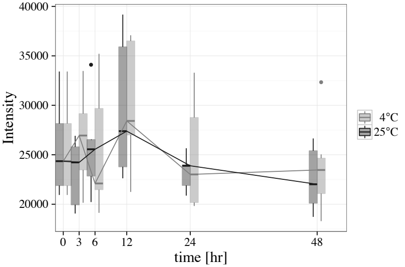

HILICneg: m/z_t_R, 445.33216_0.77

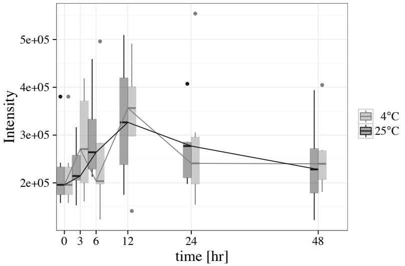

HILICneg: m/z_t_R, 575.46802_0.71

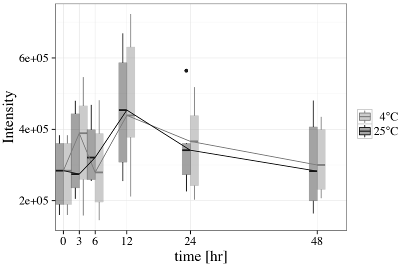

HILICneg: m/z_t_R, 383.15339_0.60

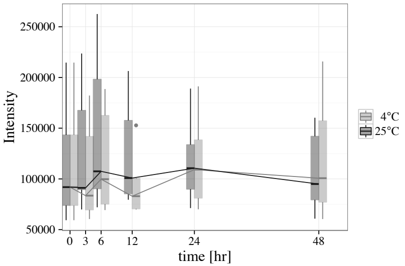

HILICneg: m/z_t_R, 187.09737_0.97

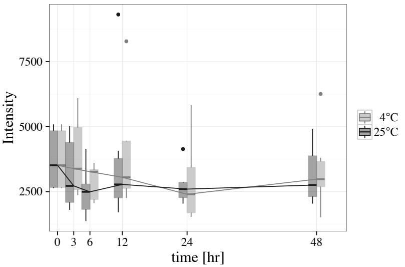

HILICneg: m/z_t_R, 155.07124_0.82

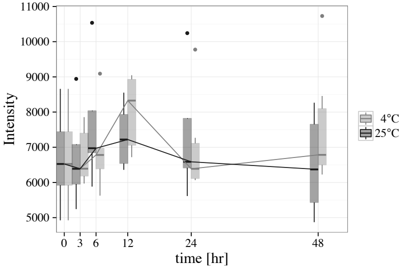

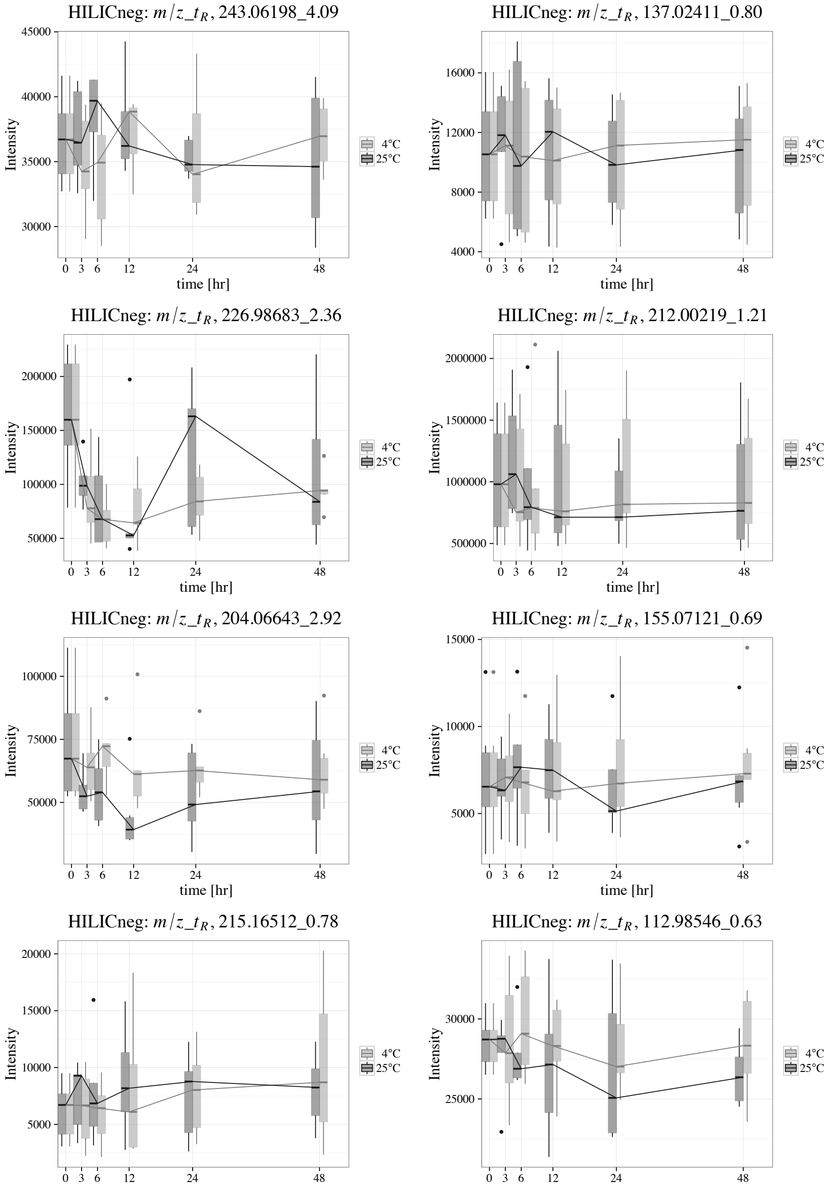
HILICneg: m/z_t_R, 243.06198_4.09
HILICneg: m/z_t_R, 137.02411_0.80
HILICneg: m/z_t_R, 226.98683_2.36
HILICneg: m/z_t_R, 212.00219_1.21
HILICneg: m/z_t_R, 204.06643_2.92
HILICneg: m/z_t_R, 155.07121_0.69
HILICneg: m/z_t_R, 215.16512_0.78
HILICneg: m/z_t_R, 112.98546_0.63
Intensity
time [hr]
0 3 6 12 24 48
4°C
25°C

time [hr]

time [hr]

HILICneg: m/z_t_R, 431.31617_0.80

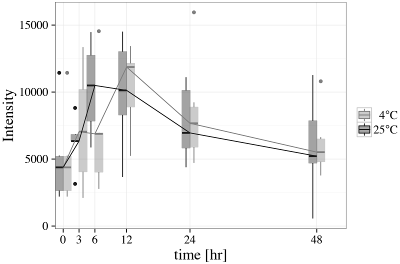

HILICneg: m/z_t_R, 129.02039_1.14

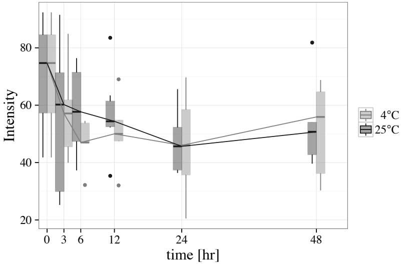

HILICneg: m/z_t_R, 391.15454_4.34

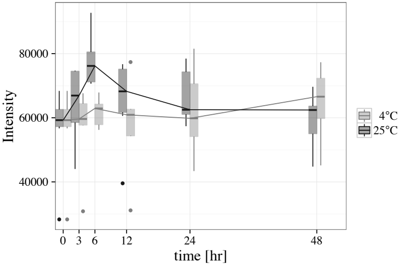

HILICneg: m/z_t_R, 369.17415_0.54

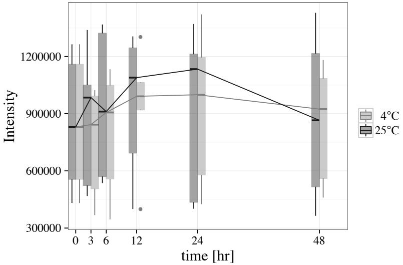

HILICneg: m/z_t_R, 149.04526_4.15

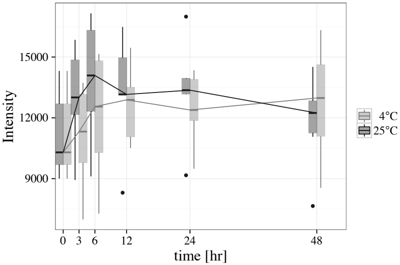

HILICneg: m/z_t_R, 145.08679_0.92

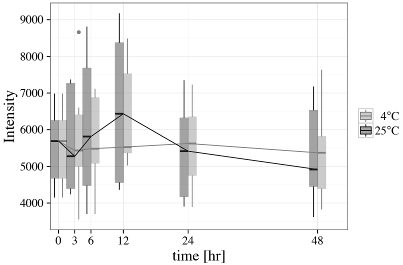

HILICneg: m/z_t_R, 165.01907_8.90

HILICneg: m/z_t_R, 289.14061_4.60

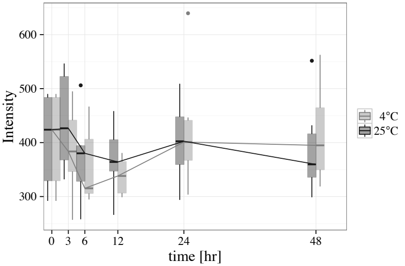

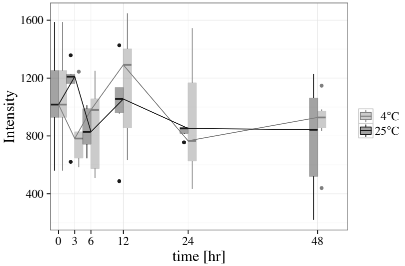

HILICneg: m/z_t_R, 194.04559_3.45

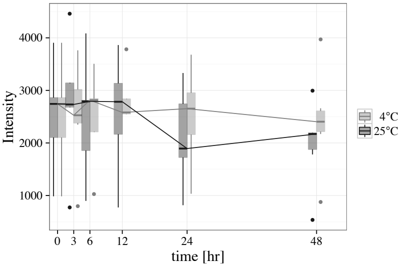

HILICneg: m/z_t_R, 291.08320_3.54

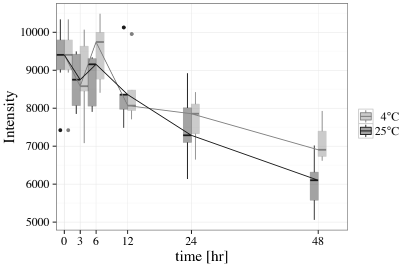

HILICneg: m/z_t_R, 182.04575_0.72

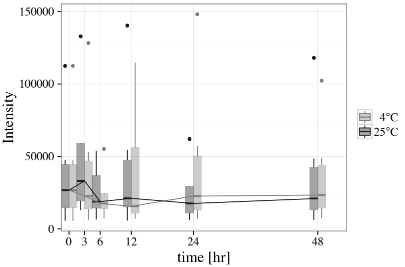

HILICneg: m/z_t_R, 369.06082_3.48

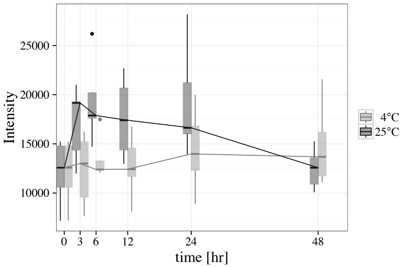

HILICneg: m/z_t_R, 103.04055_3.01

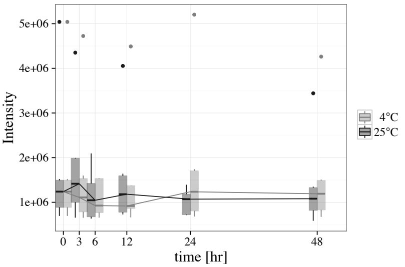

HILICneg: m/z_t_R, 189.00401_1.47

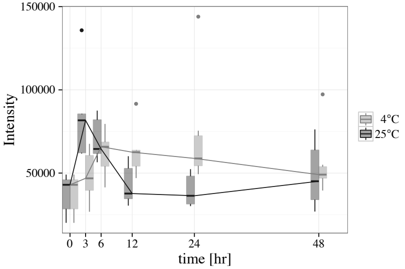

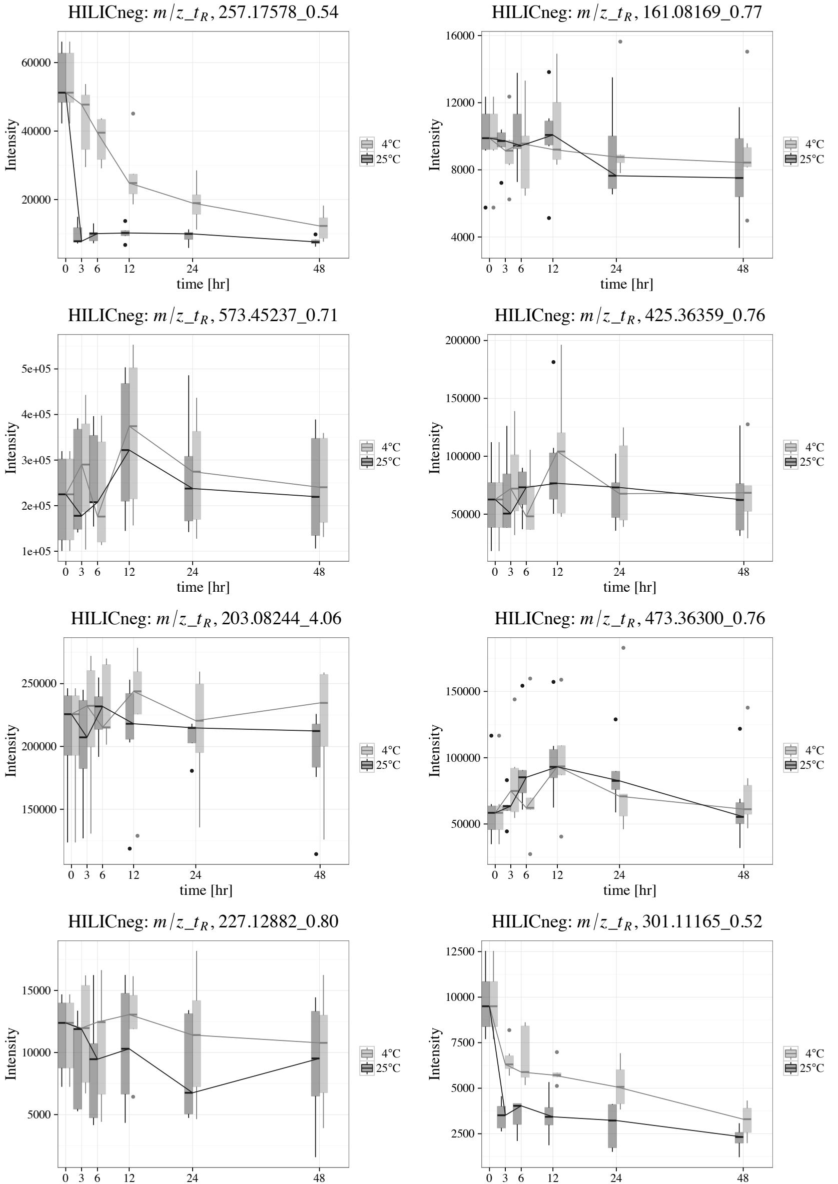

HILICneg: m/z_t_R, 257.17578_0.54
HILICneg: m/z_t_R, 161.08169_0.77
HILICneg: m/z_t_R, 573.45237_0.71
HILICneg: m/z_t_R, 425.36359_0.76
HILICneg: m/z_t_R, 203.08244_4.06
HILICneg: m/z_t_R, 473.36300_0.76
HILICneg: m/z_t_R, 227.12882_0.80
HILICneg: m/z_t_R, 301.11165_0.52
Intensity
time [hr]
0 3 6 12 24 48
4°C
25°C

time [hr]

time [hr]

HILICneg: m/z_t_R, 173.05651_3.81

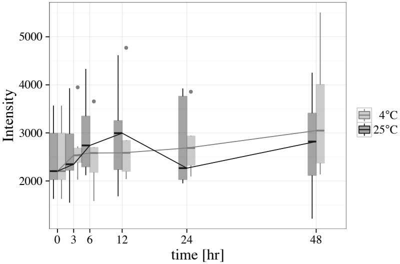

HILICneg: m/z_t_R, 551.46748_0.63

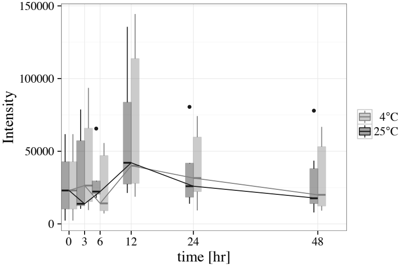

HILICneg: m/z_t_R, 200.12903_0.97

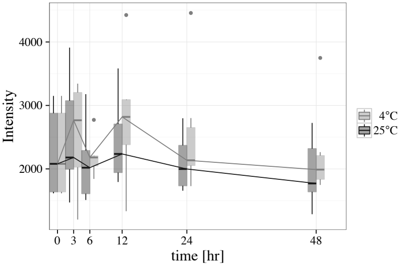

HILICneg: m/z_t_R, 157.08681_0.67

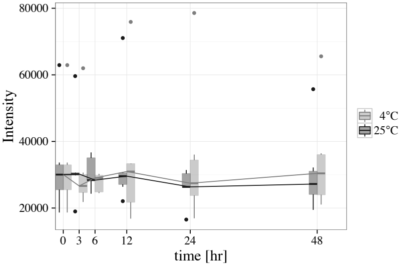

HILICneg: m/z_t_R, 145.05031_0.97

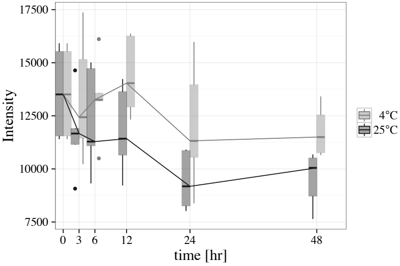

HILICneg: m/z_t_R, 788.54487_1.30

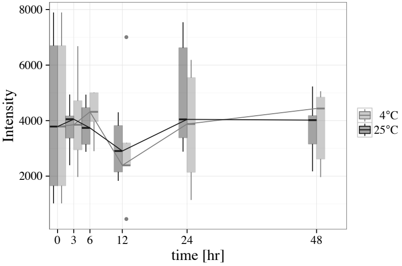

HILICneg: m/z_t_R, 126.05585_1.35

HILICneg: m/z_t_R, 149.06057_0.98

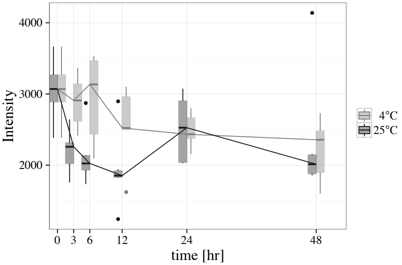

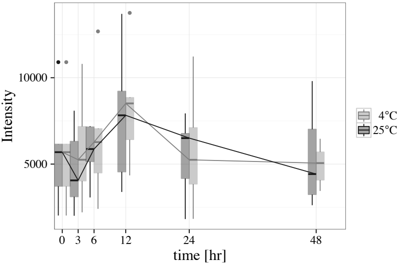

HILICneg: m/z_t_R, 411.38429_0.67

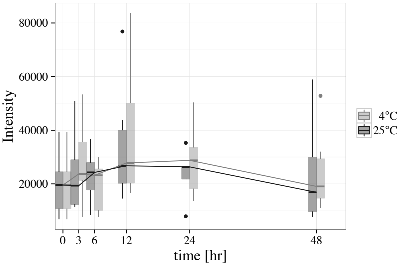

HILICneg: m/z_t_R, 117.05554_0.98

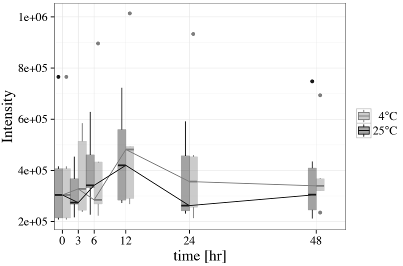

HILICneg: m/z_t_R, 433.33205_0.89

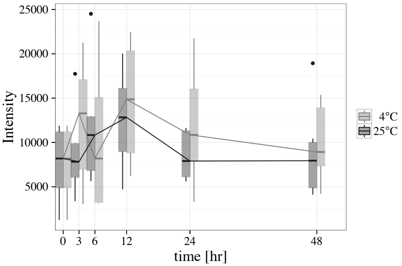

HILICneg: m/z_t_R, 417.30072_0.92

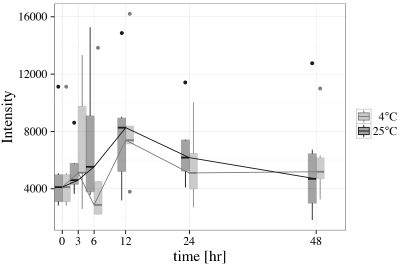

HILICneg: m/z_t_R, 489.35817_0.93

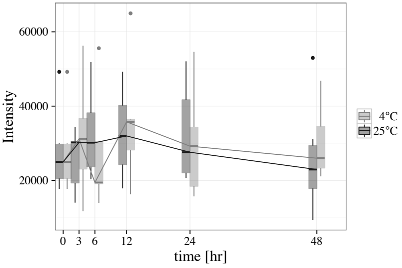

HILICneg: m/z_t_R, 112.05152_3.40

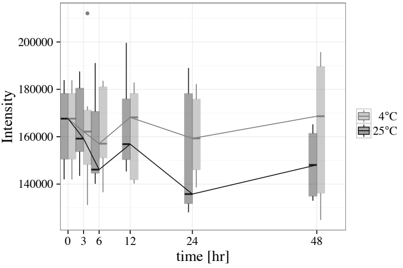

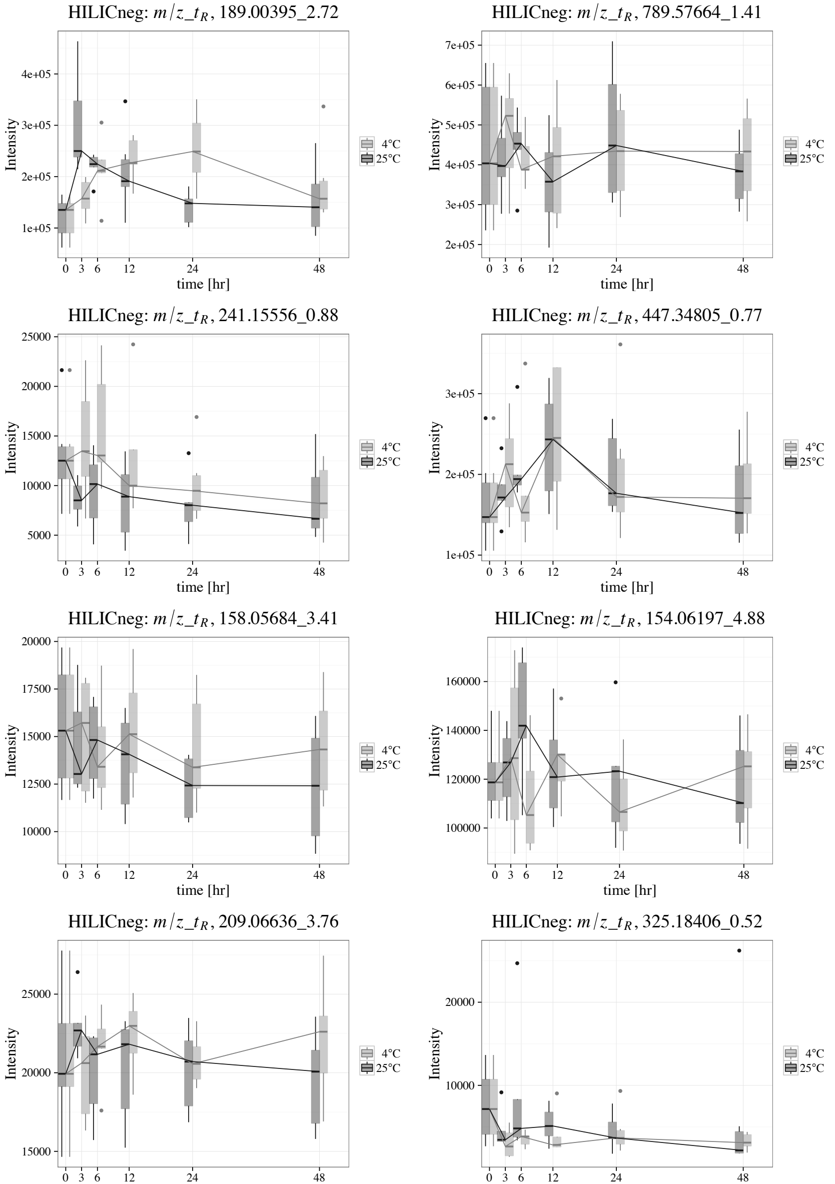
HILICneg: m/z_t_R, 189.00395_2.72
HILICneg: m/z_t_R, 789.57664_1.41
HILICneg: m/z_t_R, 241.15556_0.88
HILICneg: m/z_t_R, 447.34805_0.77
HILICneg: m/z_t_R, 158.05684_3.41
HILICneg: m/z_t_R, 154.06197_4.88
HILICneg: m/z_t_R, 209.06636_3.76
HILICneg: m/z_t_R, 325.18406_0.52
Intensity
time [hr]
0 3 6 12 24 48
4°C
25°C

time [hr]

time [hr]

HILICneg: m/z_t_R, 167.02081_4.04

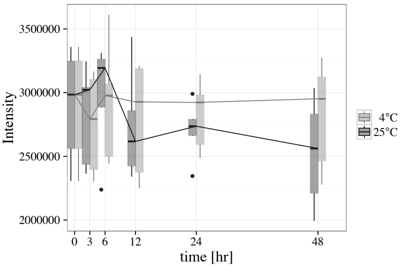

HILICneg: m/z_t_R, 249.08778_4.07

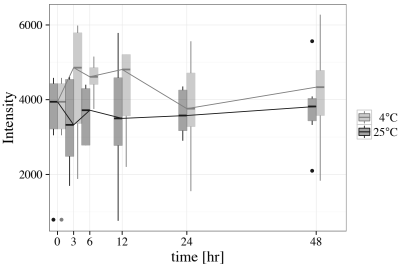

HILICneg: m/z_t_R, 193.12321_0.88

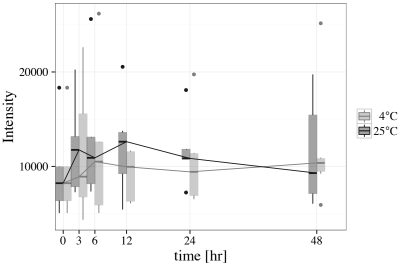

HILICneg: m/z_t_R, 245.07762_3.54

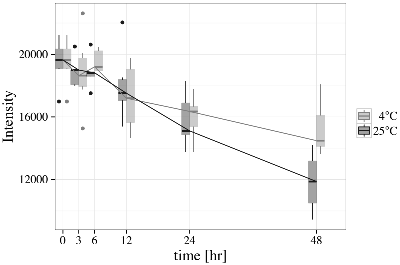

HILICneg: m/z_t_R, 207.07738_3.91

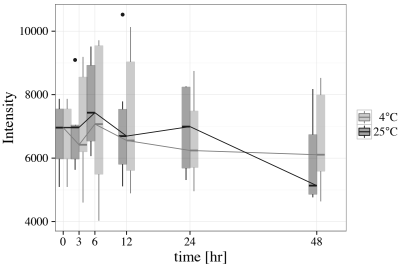

HILICneg: m/z_t_R, 352.08644_0.54

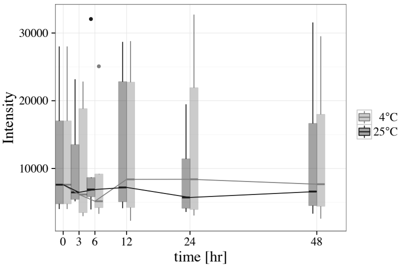

HILICneg: m/z_t_R, 111.01984_3.48

HILICneg: m/z_t_R, 146.08205_4.26

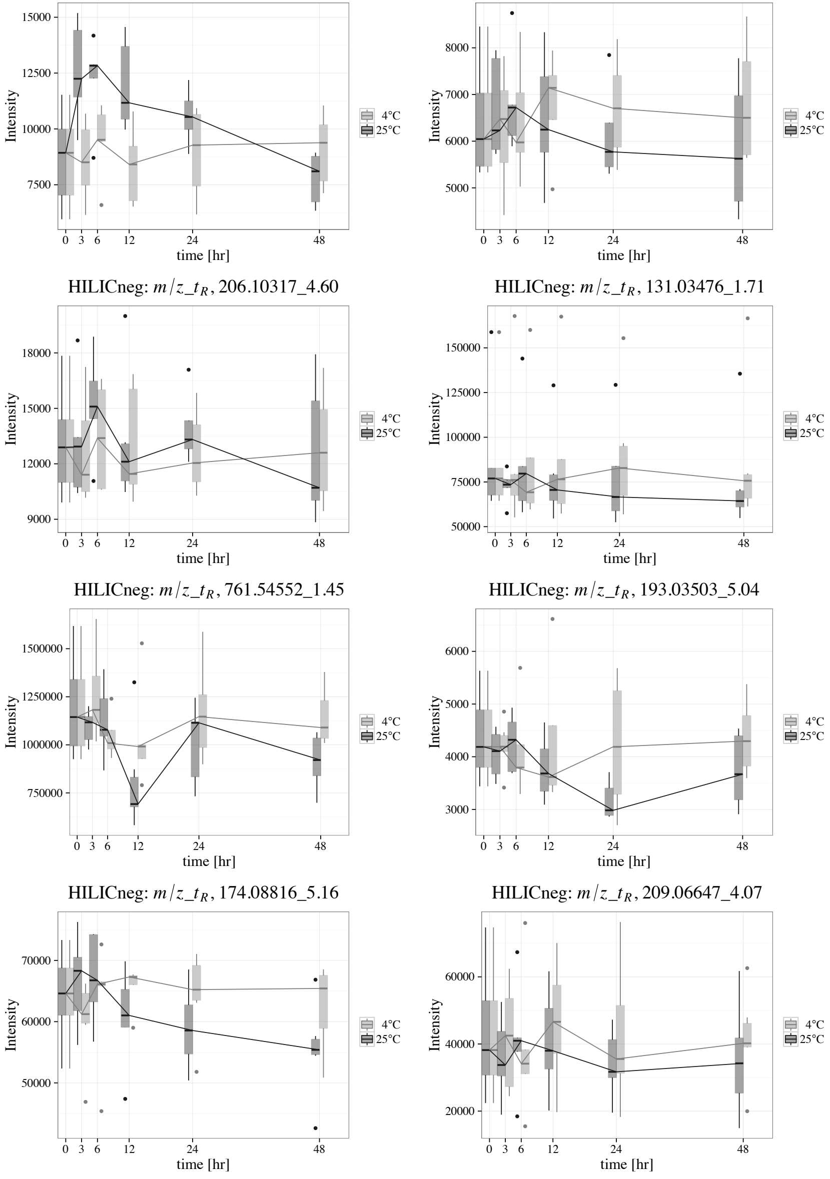
Intensity
time [hr]
4°C
25°C
HILICneg: m/z_t_R, 206.10317_4.60
HILICneg: m/z_t_R, 131.03476_1.71
HILICneg: m/z_t_R, 761.54552_1.45
HILICneg: m/z_t_R, 193.03503_5.04
HILICneg: m/z_t_R, 174.08816_5.16
HILICneg: m/z_t_R, 209.06647_4.07

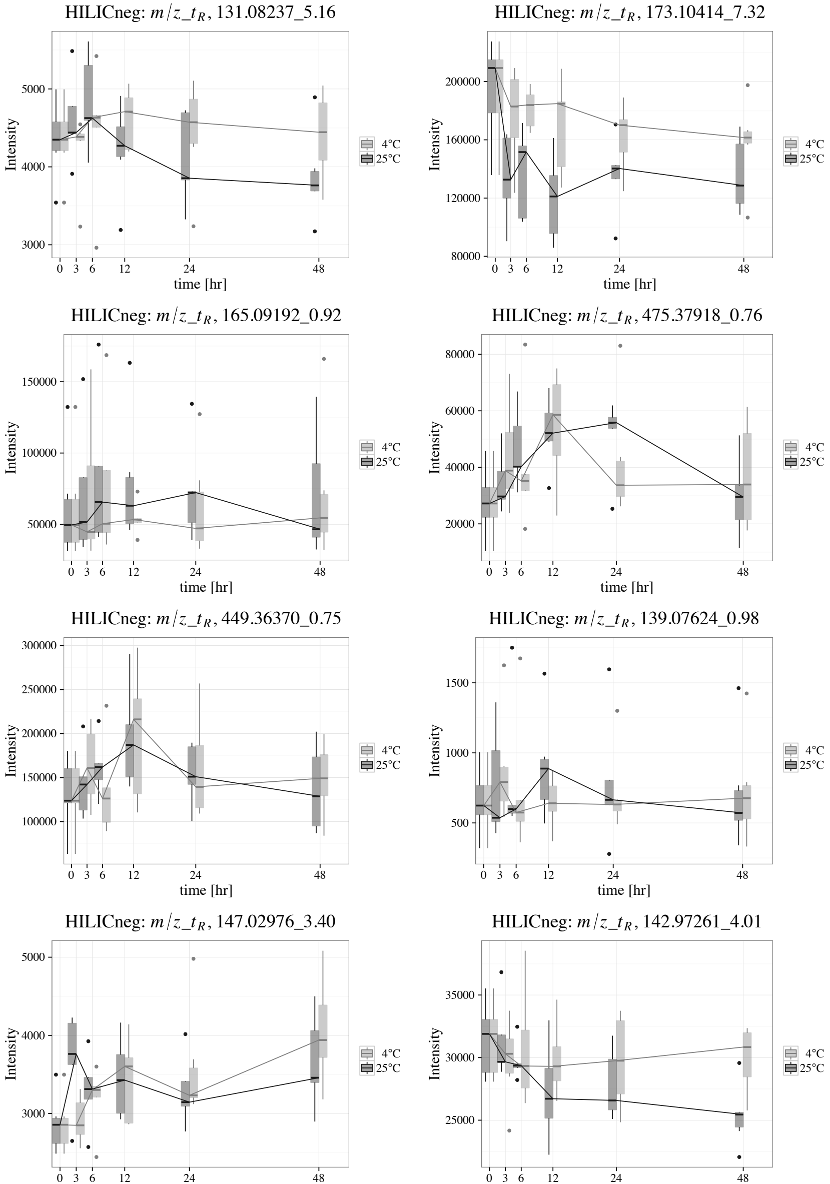
HILICneg: m/z_tR, 131.08237_5.16
HILICneg: m/z_tR, 173.10414_7.32
HILICneg: m/z_tR, 165.09192_0.92
HILICneg: m/z_tR, 475.37918_0.76
HILICneg: m/z_tR, 449.36370_0.75
HILICneg: m/z_tR, 139.07624_0.98
HILICneg: m/z_tR, 147.02976_3.40
HILICneg: m/z_tR, 142.97261_4.01
Intensity
time [hr]
4°C
25°C

time [hr]

time [hr]

HILICneg: m/z_t_R, 439.34282_0.92

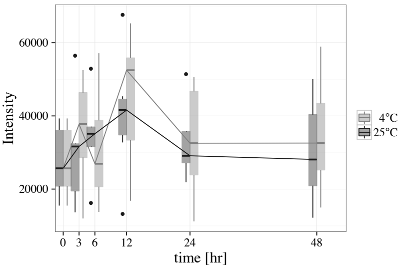

HILICneg: m/z_t_R, 804.57478_1.26

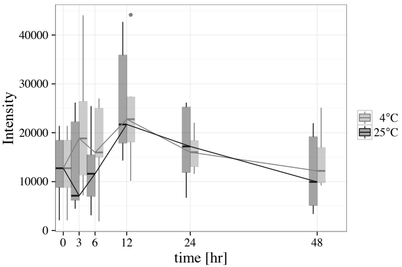

HILICneg: m/z_t_R, 179.05733_1.30

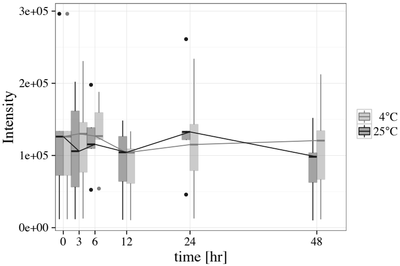

HILICneg: m/z_t_R, 178.05075_1.42

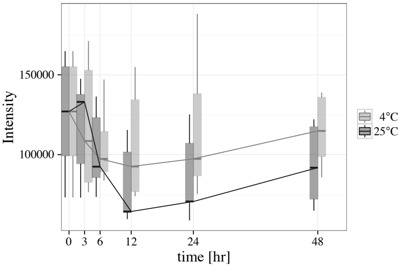

HILICneg: m/z_t_R, 327.25386_3.63

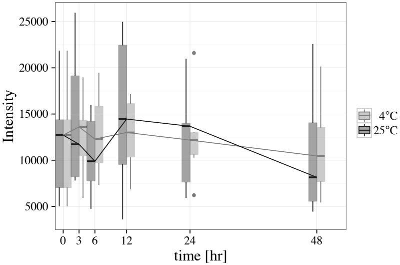

HILICneg: m/z_t_R, 199.96993_4.01

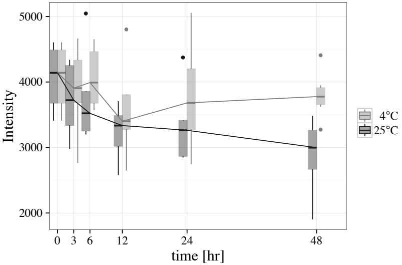

HILICneg: m/z_t_R, 149.02421_0.94

HILICneg: m/z_t_R, 168.07767_4.30

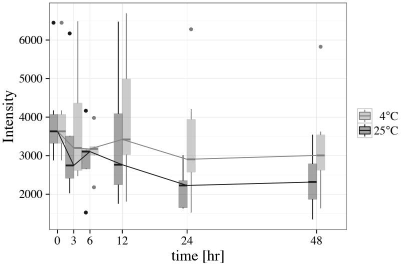

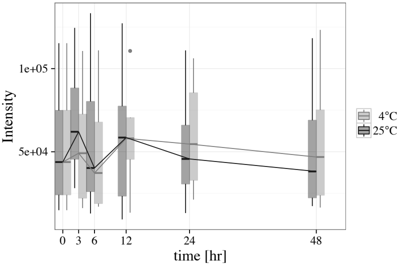

HILICneg: m/z_t_R, 131.07114_0.97

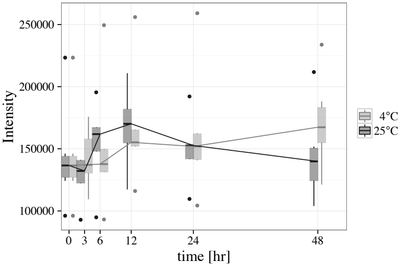

HILICneg: m/z_t_R, 223.13380_0.77

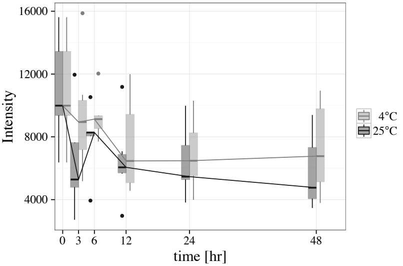

HILICneg: m/z_t_R, 144.04520_0.94

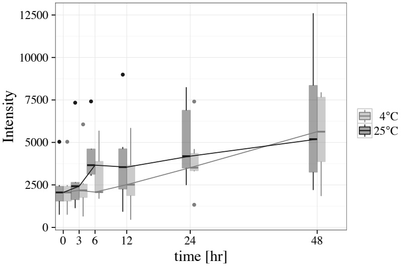

HILICneg: m/z_t_R, 205.03516_5.44

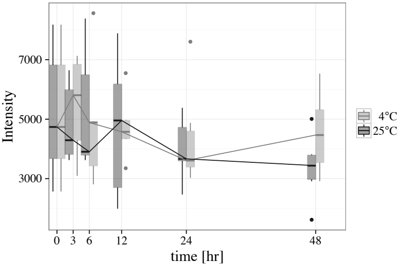

HILICneg: m/z_t_R, 202.07192_3.40

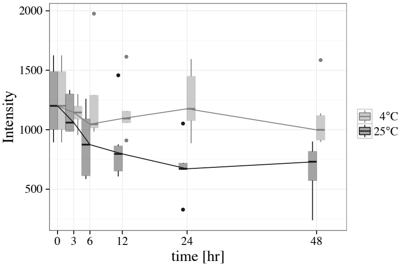

HILICneg: m/z_t_R, 274.10432_5.01

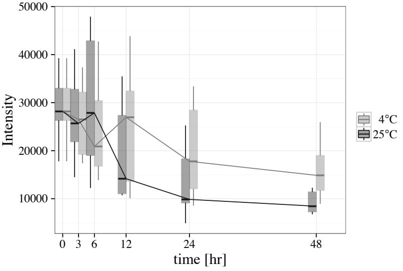

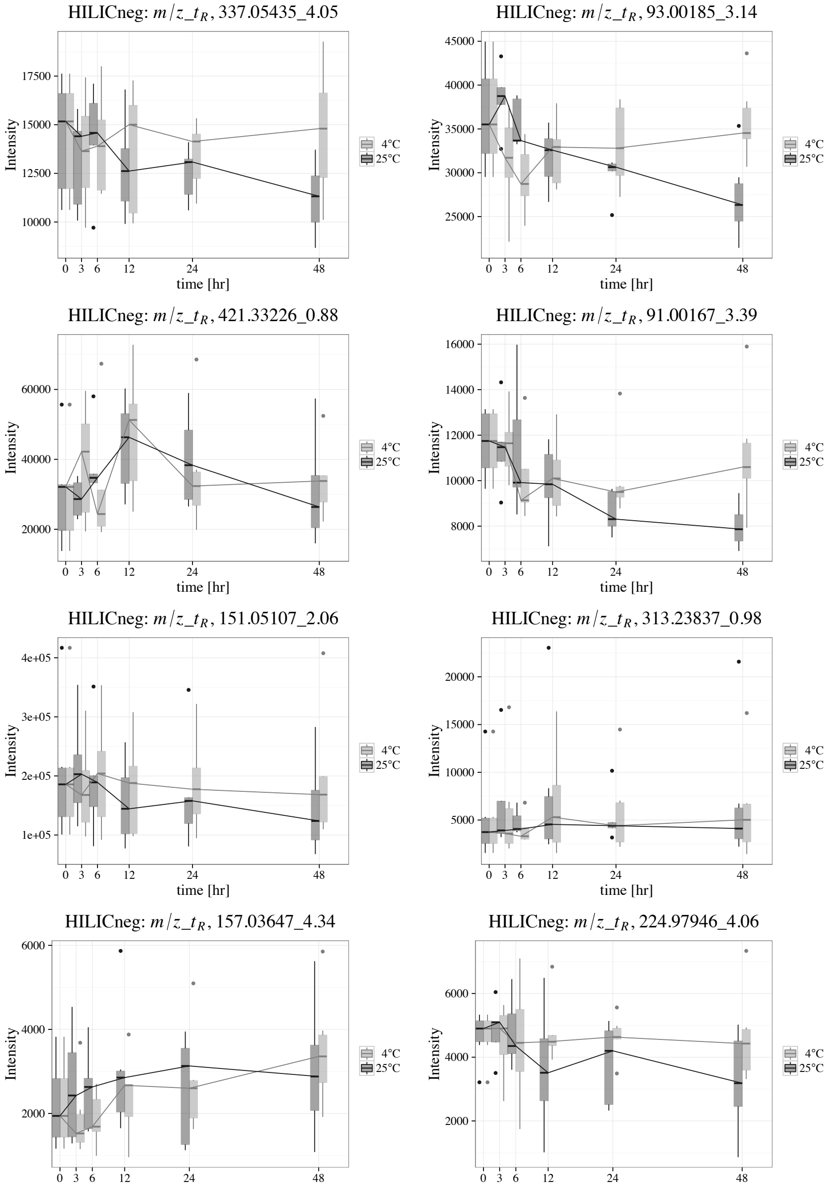

HILICneg: m/z_t_R, 337.05435_4.05
HILICneg: m/z_t_R, 93.00185_3.14
HILICneg: m/z_t_R, 421.33226_0.88
HILICneg: m/z_t_R, 91.00167_3.39
HILICneg: m/z_t_R, 151.05107_2.06
HILICneg: m/z_t_R, 313.23837_0.98
HILICneg: m/z_t_R, 157.03647_4.34
HILICneg: m/z_t_R, 224.97946_4.06
Intensity
time [hr]
4°C
25°C

time [hr]

time [hr]

HILICneg: m/z_t_R, 163.06119_4.07

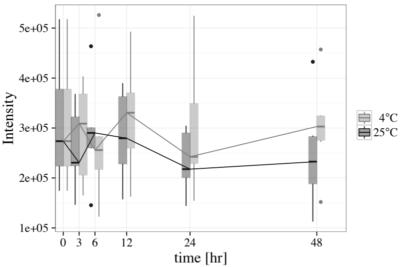

HILICneg: m/z_t_R, 144.02994_3.80

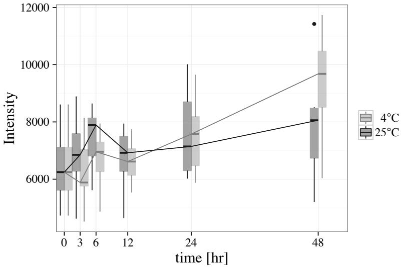

HILICneg: m/z_t_R, 91.00126_3.31

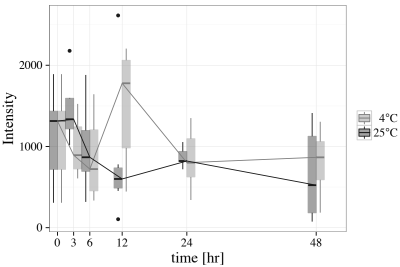

HILICneg: m/z_t_R, 119.03476_2.86

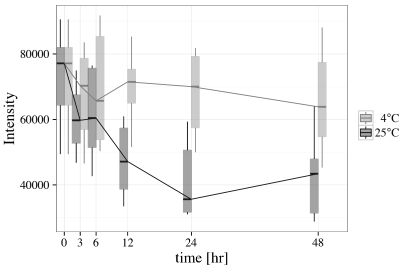

HILICneg: m/z_t_R, 89.04289_0.56

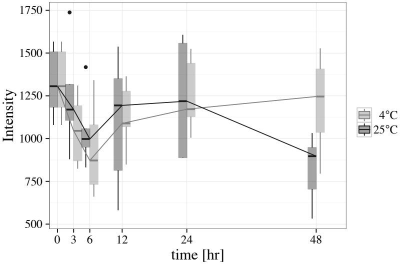

HILICneg: m/z_t_R, 591.39046_0.71

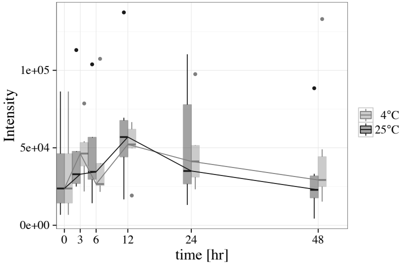

HILICneg: m/z_t_R, 443.31628_0.92

HILICneg: m/z_t_R, 160.02498_4.98

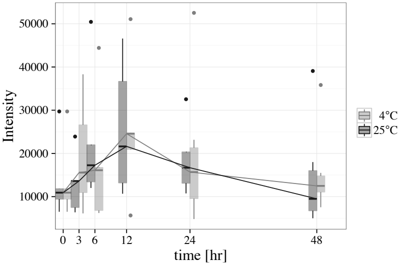

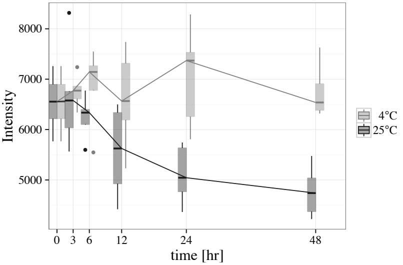

HILICneg: m/z_t_R, 71.05030_0.98

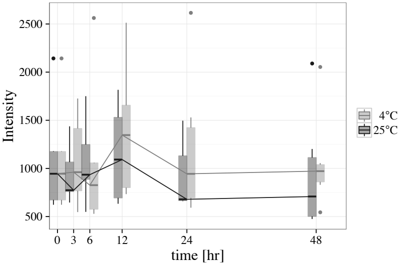

HILICneg: m/z_t_R, 329.17570_0.92

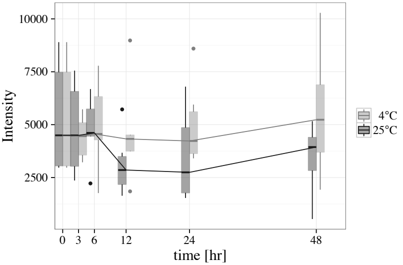

HILICneg: m/z_t_R, 151.03985_0.91

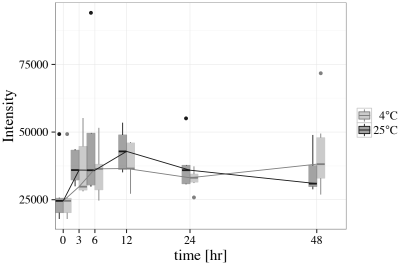

HILICneg: m/z_t_R, 129.01905_2.88

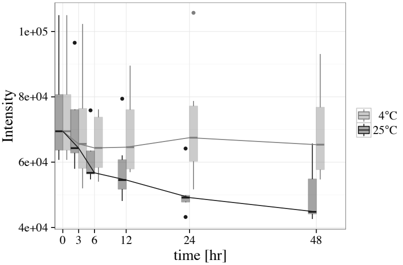

HILICneg: m/z_t_R, 274.14066_5.69

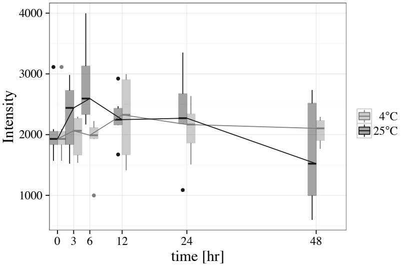

HILICneg: m/z_t_R, 176.03846_1.41

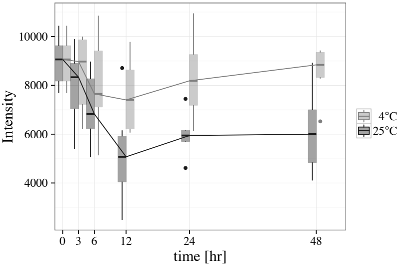

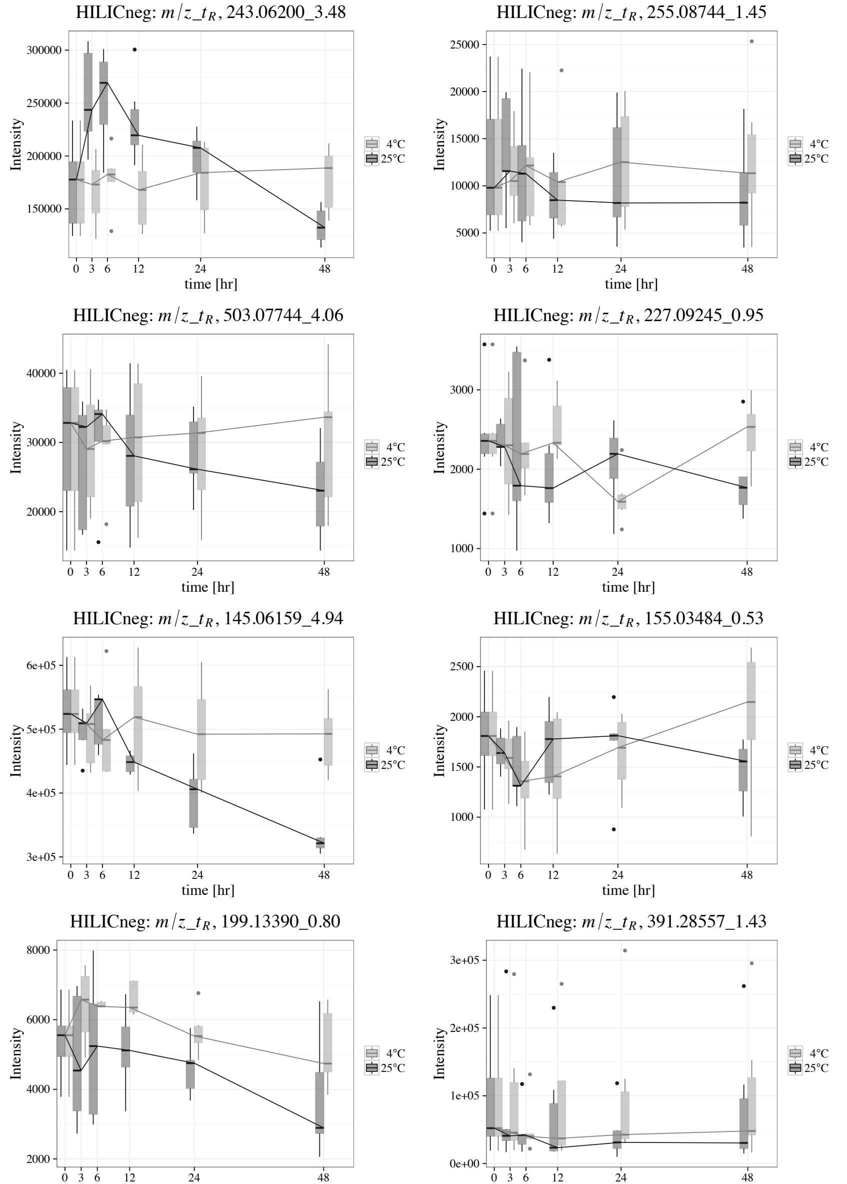
HILICneg: m/z_t_R, 243.06200_3.48
HILICneg: m/z_t_R, 255.08744_1.45
HILICneg: m/z_t_R, 503.07744_4.06
HILICneg: m/z_t_R, 227.09245_0.95
HILICneg: m/z_t_R, 145.06159_4.94
HILICneg: m/z_t_R, 155.03484_0.53
HILICneg: m/z_t_R, 199.13390_0.80
HILICneg: m/z_t_R, 391.28557_1.43
Intensity
time [hr]
0 3 6 12 24 48
4°C
25°C

time [hr]

time [hr]

HILICneg: m/z_t_R, 802.56063_0.99

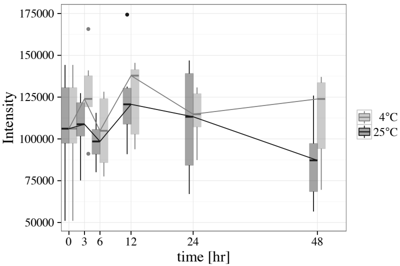

HILICneg: m/z_t_R, 173.04534_5.04

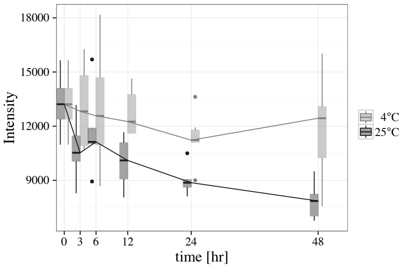

HILICneg: m/z_t_R, 291.08321_4.87

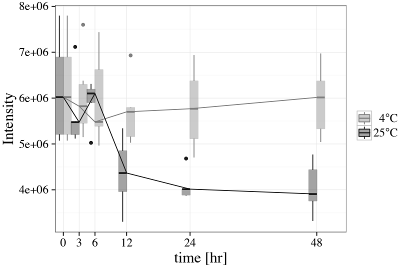

HILICneg: m/z_t_R, 821.57363_0.99

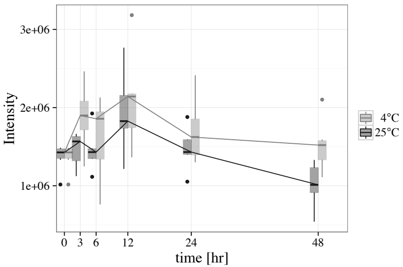

HILICneg: m/z_t_R, 137.02420_3.38

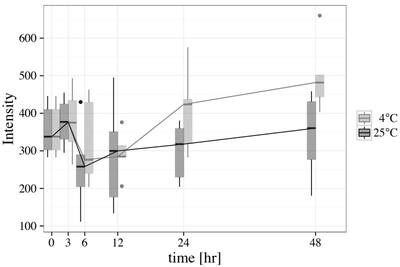

HILICneg: m/z_t_R, 343.04573_4.07

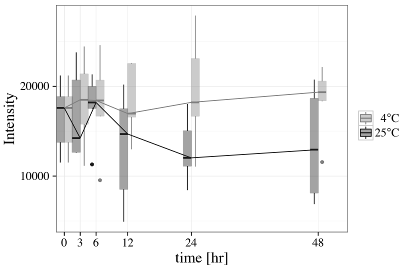

HILICneg: m/z_t_R, 263.10351_2.98

HILICneg: m/z_t_R, 541.26531_3.32

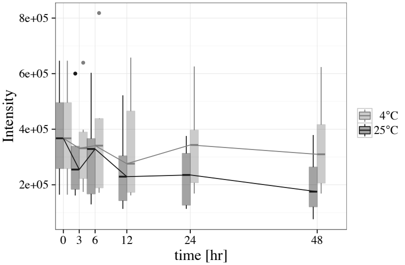

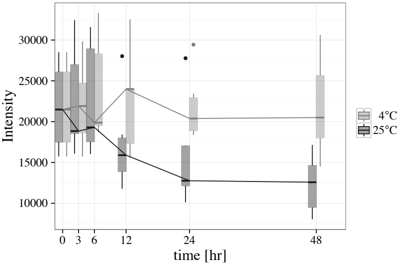

HILICneg: m/z_t_R, 454.15028_3.48

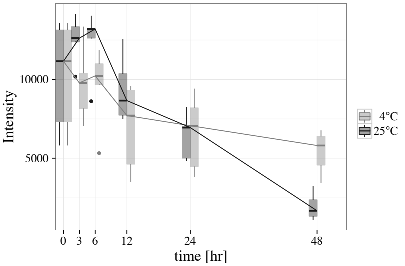

HILICneg: m/z_t_R, 448.30685_1.48

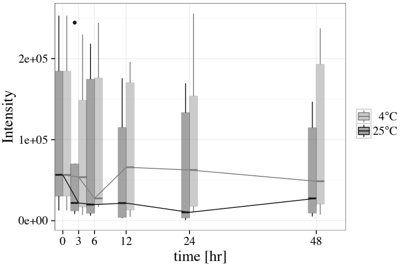

HILICneg: m/z_t_R, 749.54543_1.47

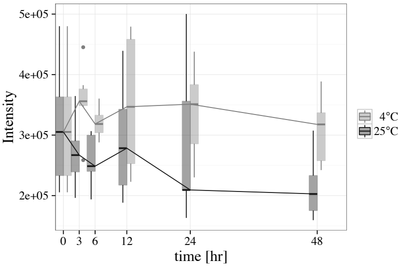

HILICneg: m/z_t_R, 130.05069_2.84

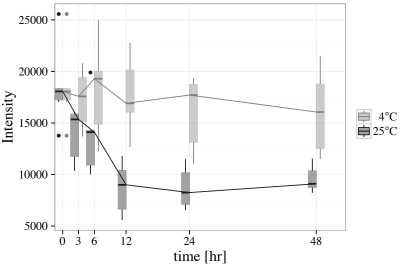

HILICneg: m/z_t_R, 175.02454_5.53

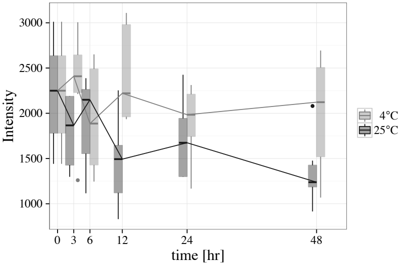

HILICneg: m/z_t_R, 437.36362_0.72

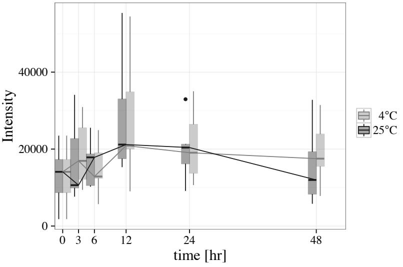

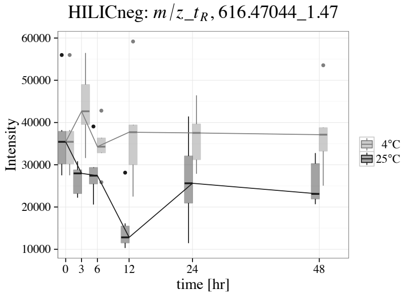
HILICneg: m/z_tR, 616.47044_1.47
Intensity
time [hr]
4°C
25°C

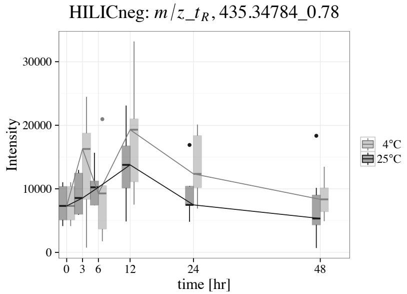
HILICneg: m/z_tR, 435.34784_0.78
Intensity
time [hr]
4°C
25°C

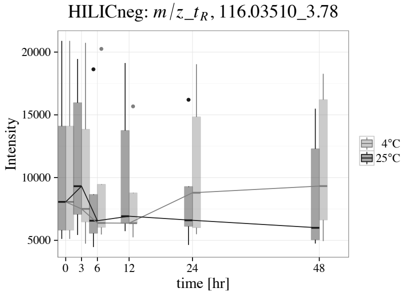
HILICneg: m/z_tR, 116.03510_3.78
Intensity
time [hr]
4°C
25°C

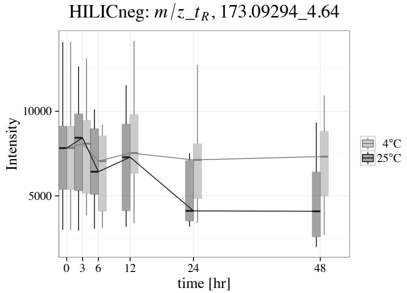
HILICneg: m/z_tR, 173.09294_4.64
Intensity
time [hr]
4°C
25°C

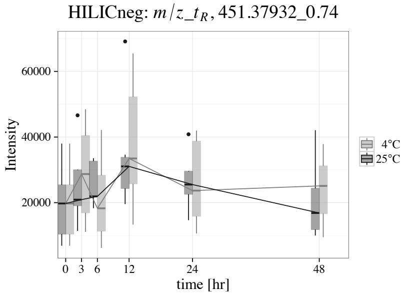
HILICneg: m/z_tR, 451.37932_0.74
Intensity
time [hr]
4°C
25°C

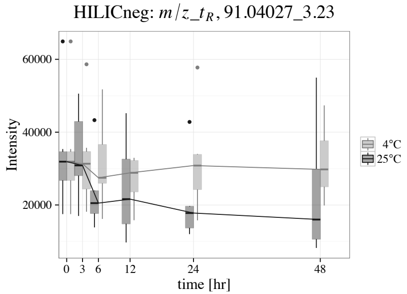
HILICneg: m/z_tR, 91.04027_3.23
Intensity
time [hr]
4°C
25°C

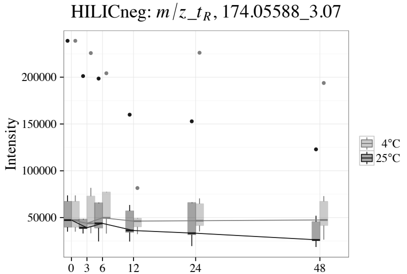
HILICneg: m/z_tR, 174.05588_3.07
Intensity
4°C
25°C

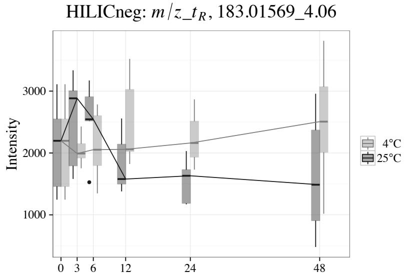
HILICneg: m/z_tR, 183.01569_4.06
Intensity
4°C
25°C

time [hr]

time [hr]

HILICneg: m/z_t_R, 187.10849_4.51

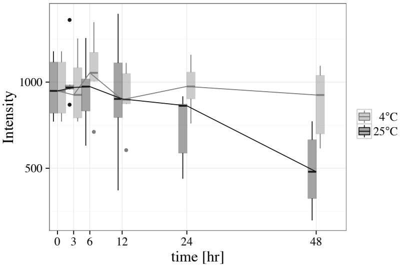

HILICneg: m/z_t_R, 180.10299_0.78

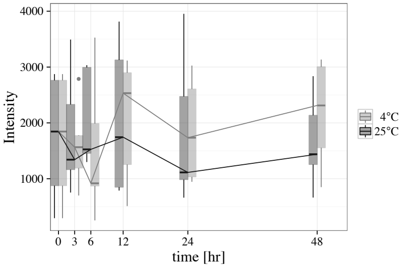

HILICneg: m/z_t_R, 133.08683_1.28

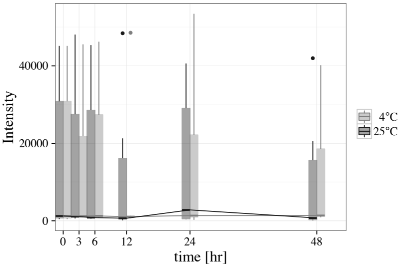

HILICneg: m/z_t_R, 207.01440_5.11

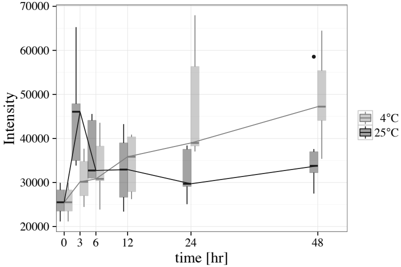

HILICneg: m/z_t_R, 763.56103_2.83

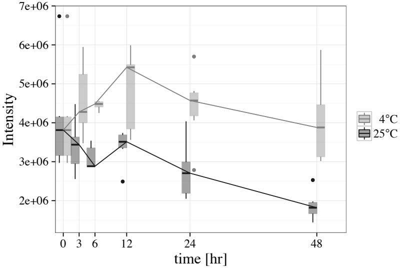

HILICneg: m/z_t_R, 376.22556_3.30

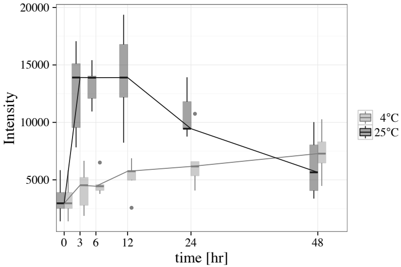

HILICneg: m/z_t_R, 761.54548_2.83

HILICneg: m/z_t_R, 267.10976_3.80

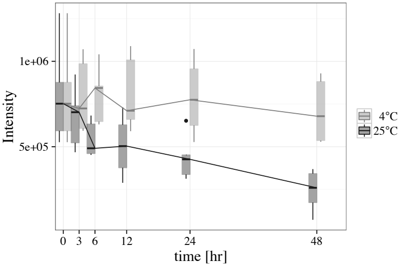

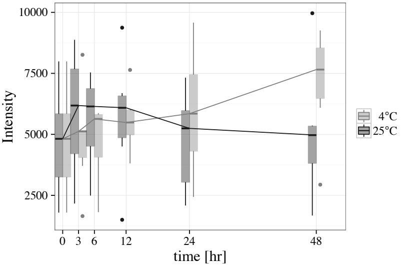

HILICneg: m/z_t_R, 478.29388_2.99

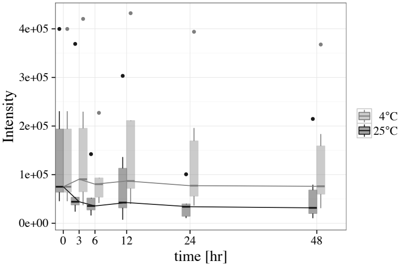

HILICneg: m/z_t_R, 143.03469_4.69

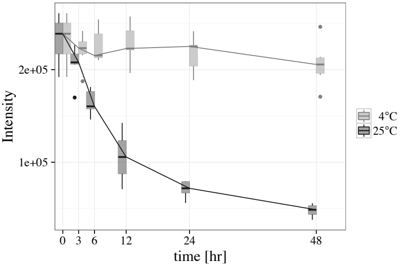

HILICneg: m/z_t_R, 524.27798_2.94

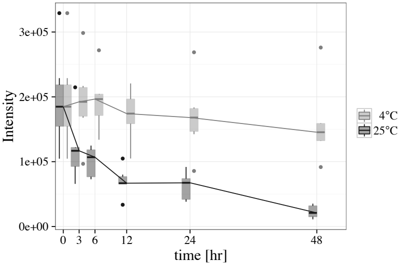

HILICneg: m/z_t_R, 101.02485_4.71

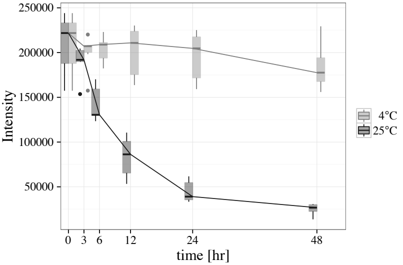

HILICneg: m/z_t_R, 113.02425_4.73

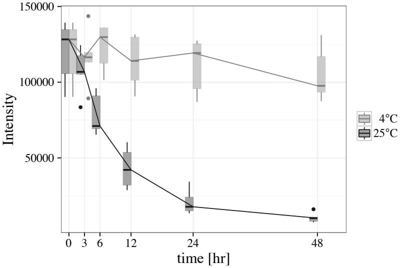

HILICneg: m/z_t_R, 131.03474_4.71

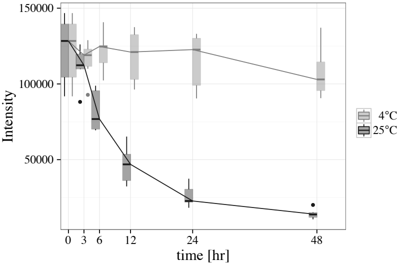

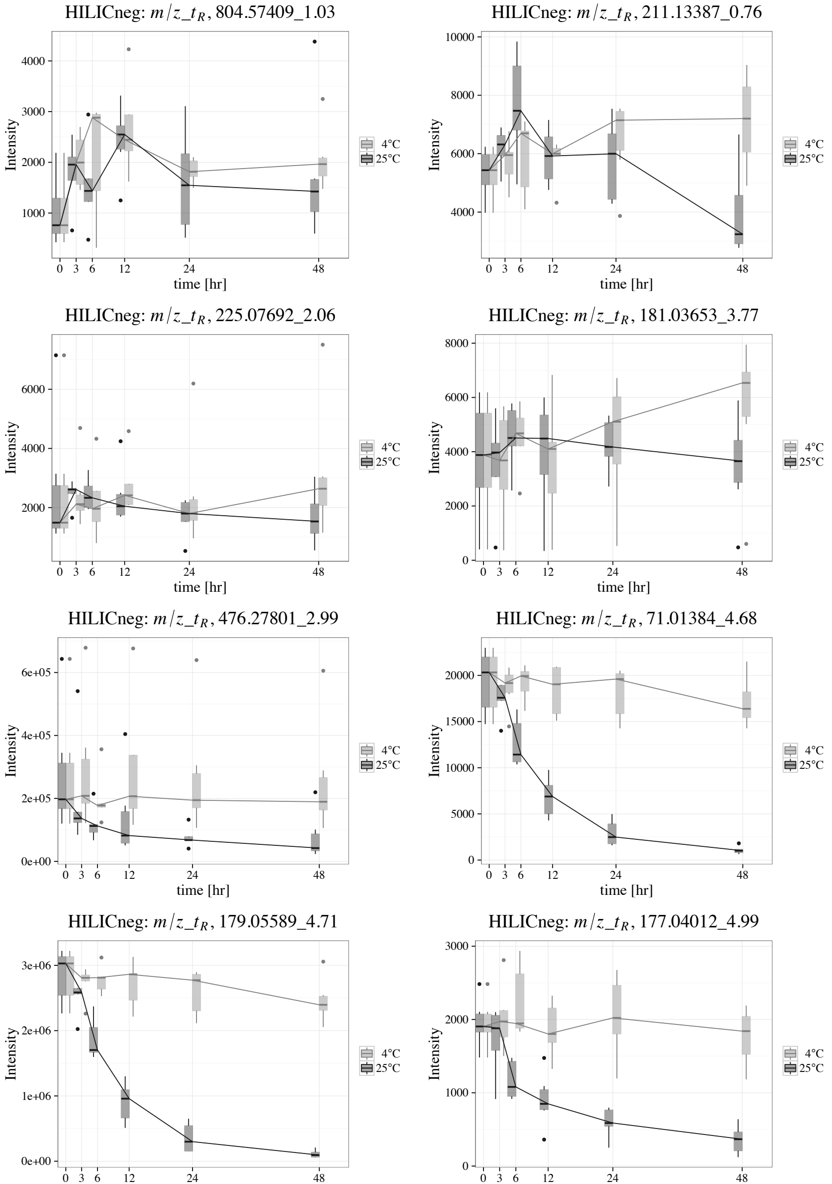

HILICneg: m/z_t_R, 804.57409_1.03
HILICneg: m/z_t_R, 211.13387_0.76
HILICneg: m/z_t_R, 225.07692_2.06
HILICneg: m/z_t_R, 181.03653_3.77
HILICneg: m/z_t_R, 476.27801_2.99
HILICneg: m/z_t_R, 71.01384_4.68
HILICneg: m/z_t_R, 179.05589_4.71
HILICneg: m/z_t_R, 177.04012_4.99
Intensity
time [hr]
4°C
25°C

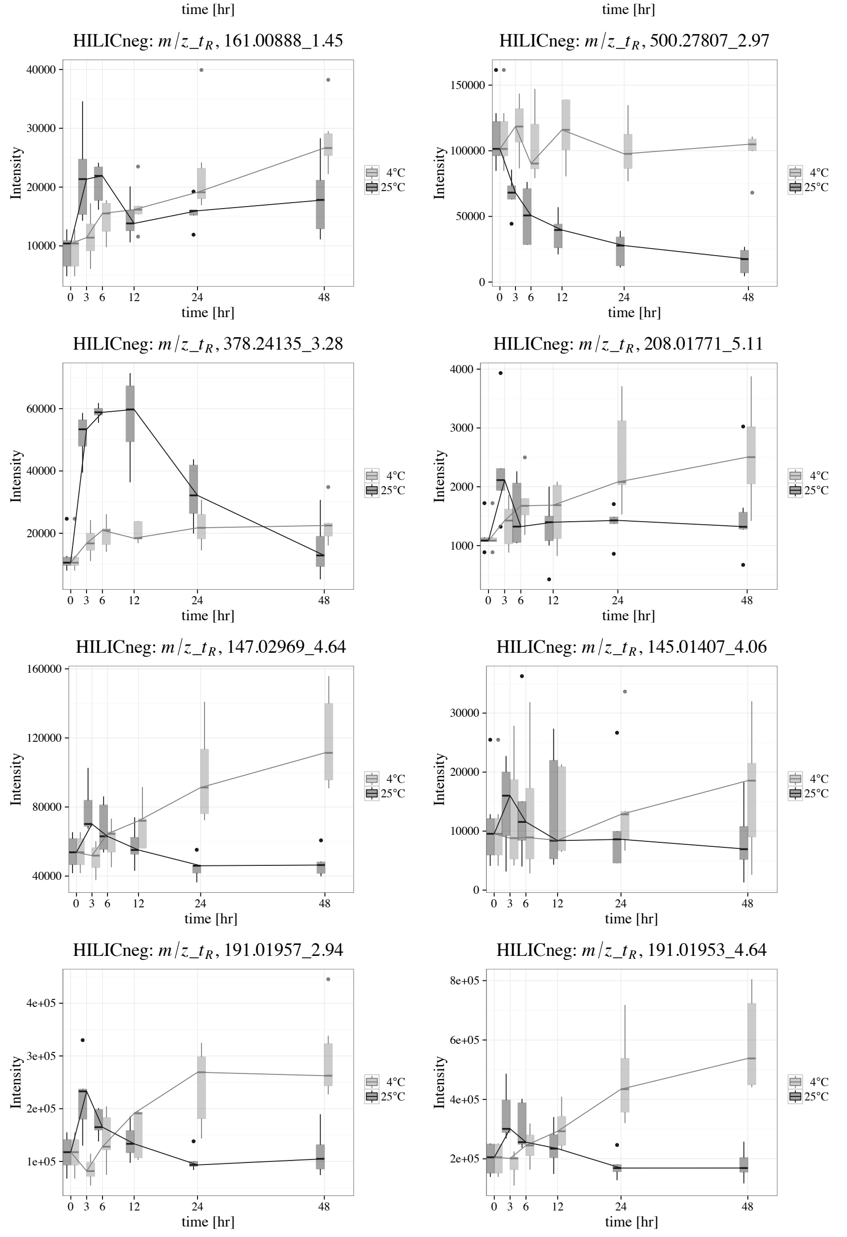

time [hr]
time [hr]
HILICneg: m/z_t_R, 161.00888_1.45
Intensity
40000
30000
20000
10000
0 3 6 12 24 48
time [hr]
4°C
25°C
HILICneg: m/z_t_R, 500.27807_2.97
Intensity
150000
100000
50000
0
0 3 6 12 24 48
time [hr]
4°C
25°C
HILICneg: m/z_t_R, 378.24135_3.28
Intensity
60000
40000
20000
0 3 6 12 24 48
time [hr]
4°C
25°C
HILICneg: m/z_t_R, 208.01771_5.11
Intensity
4000
3000
2000
1000
0 3 6 12 24 48
time [hr]
4°C
25°C
HILICneg: m/z_t_R, 147.02969_4.64
Intensity
160000
120000
80000
40000
0 3 6 12 24 48
time [hr]
4°C
25°C
HILICneg: m/z_t_R, 145.01407_4.06
Intensity
30000
20000
10000
0
0 3 6 12 24 48
time [hr]
4°C
25°C
HILICneg: m/z_t_R, 191.01957_2.94
Intensity
4e+05
3e+05
2e+05
1e+05
0 3 6 12 24 48
time [hr]
4°C
25°C
HILICneg: m/z_t_R, 191.01953_4.64
Intensity
8e+05
6e+05
4e+05
2e+05
0 3 6 12 24 48
time [hr]
4°C
25°C
